# Fatty Acid and Amino Acid Derivatives in Organocatalyzed Michael Additions

**DOI:** 10.3390/molecules31020204

**Published:** 2026-01-06

**Authors:** Aljaž Flis, Helena Brodnik, Nejc Petek, Franc Požgan, Jurij Svete, Bogdan Štefane, Luka Ciber, Uroš Grošelj

**Affiliations:** Faculty of Chemistry and Chemical Technology, University of Ljubljana, Večna pot 113, SI-1000 Ljubljana, Slovenia; af5555@student.uni-lj.si (A.F.); helena.brodnik@fkkt.uni-lj.si (H.B.); nejc.petek@fkkt.uni-lj.si (N.P.); franc.pozgan@fkkt.uni-lj.si (F.P.); jurij.svete@fkkt.uni-lj.si (J.S.); bogdan.stefane@fkkt.uni-lj.si (B.Š.)

**Keywords:** fatty acids, amino acids, β-keto esters, pyrrolones, nitroalkene acceptors, Michael addition, asymmetric organocatalysis, enantioselectivity, diastereoselectivity, tetronic acid

## Abstract

Amino acid derivatives, such as β-keto esters and pyrrolones, were used as nucleophiles in organocatalyzed Michael additions to nitroalkene acceptors, while fatty acid derivatives acted as both nucleophiles (β-keto esters) and electrophiles (nitroalkene acceptors). Bifunctional noncovalent organocatalysts were employed as asymmetric organocatalysts. Twenty compounds—including fatty acid and amino acid derivatives, as well as fatty acid–amino acid conjugates—were prepared with enantioselectivities of up to 98% *ee*. All novel products were fully characterized. This research demonstrates the ease of assembling readily available fatty acid and amino acid building blocks under ambient conditions.

## 1. Introduction

The enantioselective construction of carbon–carbon and carbon–heteroatom bonds in modern organic synthesis is crucial, as enantiomerically pure compounds play a key role in natural products and pharmaceuticals. Asymmetric organocatalysis, the third pillar of enantioselective catalysis, is a powerful tool that enables the formation of complex products from simple building blocks in an asymmetric manner, without the use of potentially toxic metals and under mild, often non-inert reaction conditions [1,2]. Among numerous organocatalyzed transformations, the asymmetric 1,4-addition to electron-deficient alkenes stands out as a key reaction, serving as a platform for organocatalyst development, the formation of advanced, functionalized, enantiomerically pure building blocks, and target-oriented asymmetric synthesis [3,4,5,6,7,8]. Even after nearly 20 years of intensive research on asymmetric organocatalyzed 1,4-additions, the reaction remains relevant and is applied to various new nucleophile–electrophile starting building blocks [9,10,11,12,13,14,15,16,17,18,19,20].

While various amino acid derivatives, such as β-keto esters [21,22], pyrrolones [22,23,24], azlactones [25,26,27], glycine Schiff bases [28,29,30,31], α,β-unsaturated amino acids [32], α-aryl-α-isocyano acetates [33], α-amino acid-derived thiazolones [34], 1*H*-imidazol-4(5*H*)-ones [35], α-imino esters [36], and others, have been used as building blocks in asymmetric organocatalyzed transformations, only a few fatty acid building blocks have been reported in asymmetric organocatalysis. Among the few reports, the enantioselective organocatalytic synthesis of 3-hydroxy fatty acids and fatty γ-lactones is notable [37,38]. Bifunctional phosphorus-based organocatalysts have been used for the atom-economical reaction of CO_2_ with epoxidized oleochemicals [39], while amines and alkanolamines as organocatalysts have been used in the amidation of fatty acid methyl esters with 3-(dimethylamino)-1-propylamine [40]. Because fatty acid derivatives exhibit various biological activities, including antitumor [41] and antiproliferative activity [42], inhibition of glioma cell growth [43], activity as inhibitors against Gram-positive *Staphylococcus aureus* [44], and serving as biomarkers of oxidative stress [45], the demand for simple and efficient enantioselective syntheses—including organocatalytic methods—of fatty acid derivatives continues to grow. On the other hand, nitro fatty acids are an emerging class of bioactive fatty acids [46,47,48,49,50,51,52,53].

We report the synthesis of readily available β-keto ester derivatives of fatty acids and amino acids [22], as well as amino acid-derived pyrrolones used as nucleophiles in the (asymmetric) organocatalyzed Michael addition to fatty acid-derived nitroalkene and *trans*-β-nitrostyrene [22]. These reactions yield a small library of fatty acid and amino acid derivatives, doubly fatty acid derivatives, and amino acid–fatty acid conjugates. The results highlight the ease of forming these potentially interesting product classes, as well as the drawbacks and challenges encountered.

## 2. Results and Discussion

**Scheme 1:**
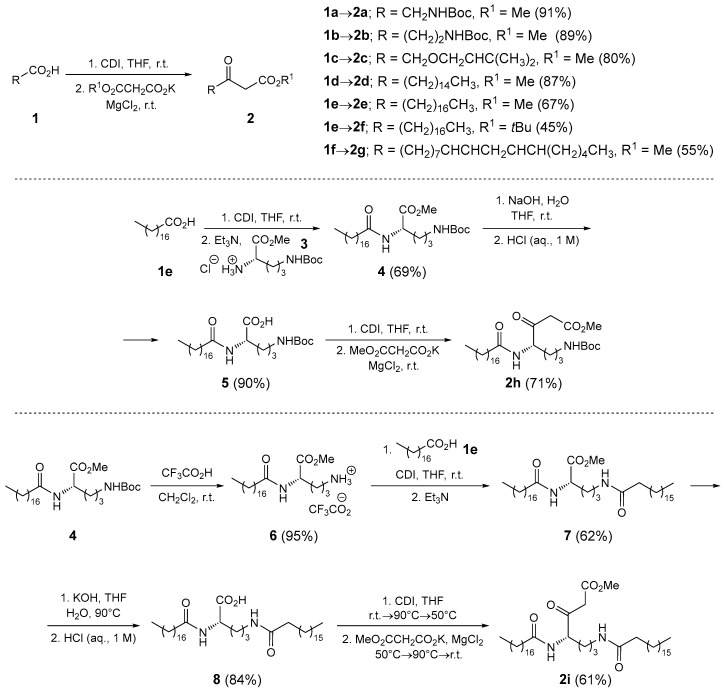
Synthesis of β-keto ester building blocks **2a**–**i**.

*Synthesis*. β-Keto ester nucleophiles were prepared by Masamune–Claisen homologation of carboxylic acids (Scheme 1) [22]. Activation of Boc-protected glycine **1a**, Boc-protected β-alanine **1b**, glycolic acid derivative **1c**, and palmitic acid (**1d**), stearic acid (**1e**), and linoleic acid (**1f**) with 1,1′-carbonyldiimidazole (CDI) in anhydrous THF, followed by addition of alkyl (methyl, *tert*-butyl) potassium malonate in the presence of magnesium chloride, afforded the corresponding β-keto esters **2a**–**g** in 45–91% yield. Except for compound **2f**, the other β-keto esters are known in the literature and are individually referenced in the Experimental section. β-Keto esters **2h** and **2i** were prepared in three and five steps, respectively, from H-Orn(Boc)-OMe·HCl (**3**). Amidation of **3** with stearic acid (**1e**) gave amido ester **4**, which was hydrolyzed under basic conditions to acid **5** and then homologated to β-keto ester **2h** in 44% yield over three steps. For the preparation of **2i**, compound **4** was Boc-deprotected using TFA to give ammonium salt **6**, followed by amidation with stearic acid (**1e**) to yield diamido ester **7**. Hydrolysis of **7** furnished acid **8**, which was transformed into β-keto ester **2i** in 20% yield over five steps (Scheme 1). Compounds **6**–**8** and **2i** have low solubility in most solvents, including chloroform and dimethyl sulfoxide. Solubility in chloroform increases significantly with the addition of small amounts of trifluoroacetic acid (700 μL CHCl_3_ and 20 μL TFA to dissolve 10–20 mg of the above-mentioned compounds).

**Scheme 2:**
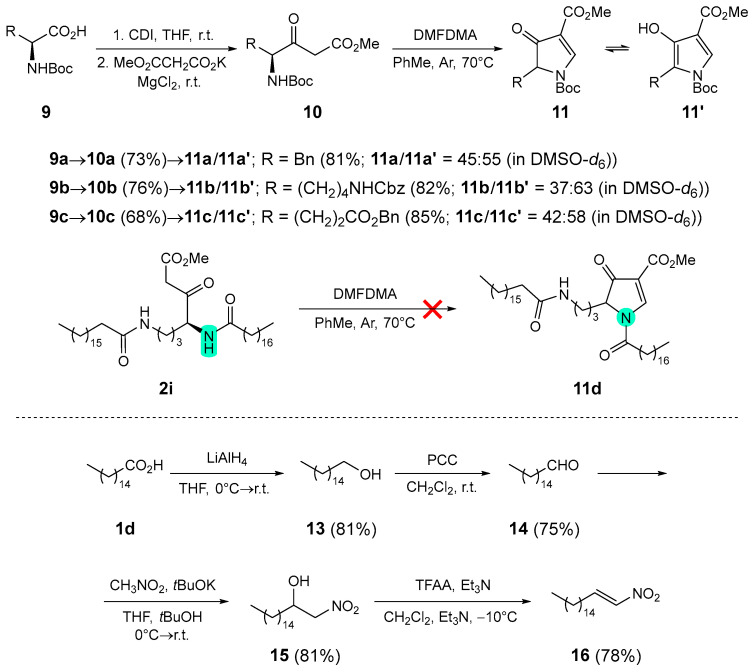
Synthesis of the starting building blocks: pyrrolones **11a**–**c** and the fatty acid-derived nitroalkene **16**.

Similarly, the pyrrolone nucleophiles **11a**–**c** were prepared from appropriately protected phenylalanine **9a**, ornithine **9b**, and glutamic acid **9c** in a two-step procedure (Scheme 2) [22,23]. Initial homologation to the corresponding β-keto esters **10a**–**c** was followed by treatment with *N*,*N*-dimethylformamide dimethyl acetal (DMFDMA) at elevated temperature, yielding the corresponding pyrrolones **11a**–**c** in 57–62% yield after two steps. All attempts to prepare the corresponding pyrrolone **11d** from amido β-keto ester **2h** resulted in complex product mixtures (Scheme 2). In addition to commercially available *trans*-β-nitrostyrene (**12**), fatty acid-derived nitroalkene electrophile **16** was prepared in four steps from palmitic acid (**1d**) following procedures described in the literature [54,55,56]. Reduction of palmitic acid (**1d**) with LiAlH_4_ gave alcohol **13**, which was oxidized with pyridinium chlorochromate (PCC) to aldehyde **14**. The Henry reaction with nitromethane in the presence of *t*BuOK afforded β-nitro alcohol **15**, which was dehydrated after treatment with trifluoroacetic anhydride (TFAA) and Et_3_N at −10 °C to nitroalkene **16** in 38% yield over four steps (Scheme 2).

**Scheme 3:**
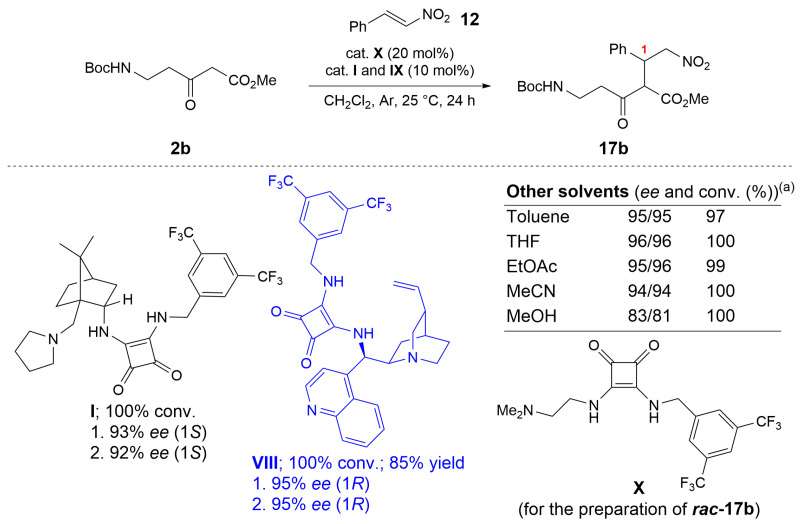
Organocatalyst and solvent optimization for the addition of Boc-β-alanine-derived β-keto ester **2b** to *trans*-β-nitrostyrene (**12**). **2b** (0.3 mmol), **12** (0.2 mmol), cat. **I** and **VIII** (10 mol%), anhydrous CH_2_Cl_2_ (1.0 mL), 25 °C, Ar, 24 h. Catalyst **X** was used to prepare ***rac*-17b**. ^(a)^ Two values for the *ee*, i.e., 95/95, refer to the enantiomeric excess of the C(1) chiral center in the two inseparable diastereomers; the diastereomer ratio of the crude mixtures could not be reliably determined from the corresponding proton spectra.

Next, the organocatalyzed 1,4-addition of Boc-β-alanine-derived β-keto ester **2b** to *trans*-β-nitrostyrene (**12**), yielding adduct **17b**, was used as a model reaction for catalyst optimization (Scheme 3) [21]. Noncovalent bifunctional organocatalysts **I**–**IX** (see ESI, Scheme S1) were tested, and achiral organocatalyst **X** was used to prepare the racemic product ***rac*-17b**. The best results for enantioselectivity, conversion, and reaction profile were obtained with the (+)-cinchonine-derived squaramide catalyst **VIII** (100% conversion, 85% yield, 95% *ee* for both diastereomers, (1*R*)-enantioselectivity), followed by the camphor-derived squaramide catalyst **I**, which showed the best complementary enantioselectivity (100% conversion, 92% and 93% *ee* for both diastereomers, (1*S*)-enantioselectivity). Screening of solvents (toluene, THF, EtOAc, MeCN) revealed broad compatibility of the model reaction catalyzed by **VIII** (*ee* between 94% and 96%), except for MeOH (81% and 83% *ee*) (Scheme 3; for details, see ESI, Scheme S2).

**Scheme 4:**
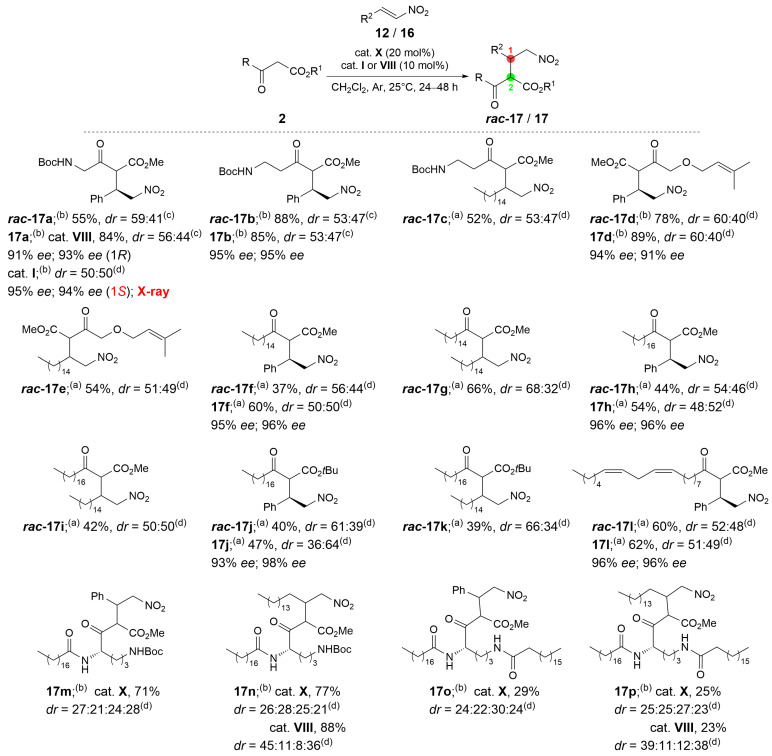
Scope for the addition of β-keto ester **2** to *trans*-β-nitrostyrene (**12**) and fatty acid-derived nitroalkene **16**; if not stated otherwise, organocatalyst **VIII** was used, yielding (1*R*)-enantioselectivity. Catalyst **X** was used to prepare the racemic products ***rac*-17a**–**l**. ^(a)^ **2** (0.3 mmol), **12** or **16** (0.2 mmol), cat. **X** (20 mol%), **I** (10 mol%), and **VIII** (10 mol%), anhydrous CH_2_Cl_2_ (1.0 mL), 25 °C, Ar, 24–48 h. ^(b)^ **2** (0.2 mmol), **12** or **16** (0.3 mmol), cat. **X** (20 mol%), **I** (10 mol%), and **VIII** (10 mmol%), anhydrous CH_2_Cl_2_ (1.0 mL), 25 °C, Ar, 24–48 h. ^(c)^ In DMSO-*d*_6_. ^(d)^ In CDCl_3_.

With optimal reaction conditions established (organocatalyst **VIII**, dichloromethane, 25 °C), the scope of the organocatalyzed 1,4-additions of β-keto esters **2** to *trans*-β-nitrostyrene (**12**) and fatty acid-derived nitroalkene **16** was explored. The achiral bifunctional noncovalent organocatalyst **X** was used efficiently to prepare all racemic adducts ***rac*-17a**–**l** in 37–88% yield. All doubly fatty acid-derived products ***rac*-17g**,**i**,**k**, as well as products ***rac*-17c** and ***rac*-17e**, could not be separated by chiral HPLC. Only the racemic products ***rac*-17a**,**b**,**d**,**f**,**h**,**j**,**l** for which all four stereoisomers (two pairs of racemic diastereomers) were separated on a chiral HPLC column, were resynthesized using chiral noncovalent bifunctional organocatalyst **VIII** (Scheme 4); the corresponding chiral nonracemic products **17a**,**b**,**d**,**f**,**h**,**j**,**l** were obtained in 47–89% yield, with high enantioselectivity (91–98% *ee*) and diastereoselectivity ranging from 50:50 to 64:36. The absolute configuration at the C-2 chiral center is unstable because of rapid keto–enol tautomerization, resulting in low observed diastereoselectivity of the products. Lastly, chiral nonracemic β-keto esters **2h** and **2i** were used for addition to *trans*-β-nitrostyrene (**12**) and fatty acid-derived nitroalkene **16**. With the achiral organocatalyst **X**, addition of **2h** to *trans*-β-nitrostyrene (**12**) and **16** gave the desired products **17m** (71% yield) and **17n** (77% yield), respectively, each as an inseparable mixture of four diastereomers in 27:21:24:28 and 26:28:25:21 ratios, respectively. Similarly, addition of **2i** to **12** and **16** gave the desired products **17o** (29% yield) and **17p** (25% yield), respectively, each as an inseparable mixture of four diastereomers in 24:22:30:24 and 25:25:27:23 ratios, respectively. Application of chiral quinuclidine catalyst **VIII** for the synthesis of products **17n** and **17p** improved the diastereomer ratio, yielding compound **17n** in 88% yield with a 45:11:8:36 diastereomer ratio and compound **17p** in 23% yield with a 39:11:12:38 diastereomer ratio (Scheme 4). No attempts have been made to determine the diastereomeric or enantiomeric ratios of compounds **17m**–**n** by HPLC. The stereochemical integrity (enantiomer ratio) of the starting β-keto esters **2h** and **2i** was not verified; nevertheless, this transformation highlights the challenges encountered—diastereoselectivity and separation of stereomers—in this simple, inherently diastereoselective reaction.

**Scheme 5:**
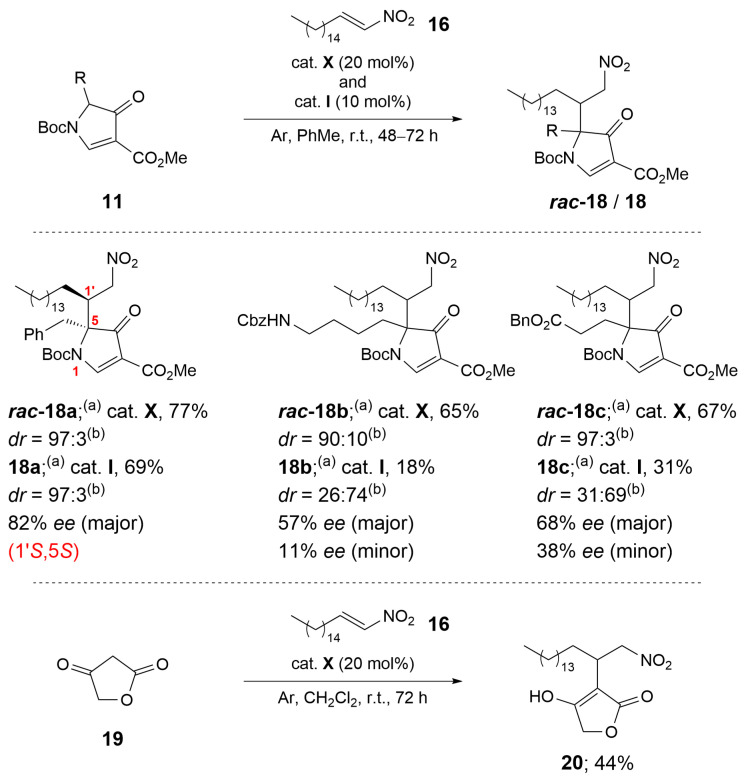
Scope for the addition of pyrrolones **11** and tetronic acid (**19**) to fatty acid-derived nitroalkene **16**. ^(a)^ **11** or **19** (0.3 mmol), **16** (0.2 mmol), cat. **X** (20 mol%) or **I** (10 mol%), anhydrous PhMe or CH_2_Cl_2_ (1.0 mL), 25 °C, Ar, 48–72 h. ^(b)^ In CDCl_3_.

Next, phenylalanine-**11a**, ornithine-**11b**, and glutamic acid-derived pyrrolones **11c** were tested as nucleophiles in the organocatalyzed addition to fatty acid nitroalkene **16** (Scheme 5). Reactions with racemic catalyst **X** proceeded smoothly, yielding the corresponding products ***rac*-18a**–**c** in 65–77% yields with high diastereoselectivity, ranging from 90:10 to 97:3. Application of the camphor-derived organocatalyst **I** in anhydrous toluene at room temperature, which were the optimal catalyst and conditions in our previous organocatalyzed additions of pyrrolone nucleophiles to nitroalkene acceptors [23], gave the corresponding chiral products **18a**–**c** in 18–69% yield. For the phenylalanine-derived product **18a** (69% yield), the diastereoselectivity remained unchanged at 97:3, with an enantioselectivity of 82% *ee* for the major diastereomer. For the ornithine-derived adduct **18b** and the glutamic acid-derived adduct **18c**, the diastereoselectivity with organocatalyst **I** not only decreased significantly but also reversed, changing from 90:10 to 26:74 for **18b** and from 97:3 to 31:69 for **18c**. For both diastereomers of product **11b**, enantioselectivity could only be tentatively determined due to partial overlap (57% *ee* for the major diastereomer and 11% *ee* for the minor diastereomer). Similarly, for product **18c**, enantioselectivity was 68% *ee* for the major diastereomer and 38% *ee* for the minor diastereomer. Finally, tetronic acid (**19**) was used as a nucleophile in the addition to nitroalkene **16**, yielding the expected product **20** in 44% yield. All attempts to separate the stereoisomers of product **20** were unsuccessful (Scheme 5).

**Figure 1:**
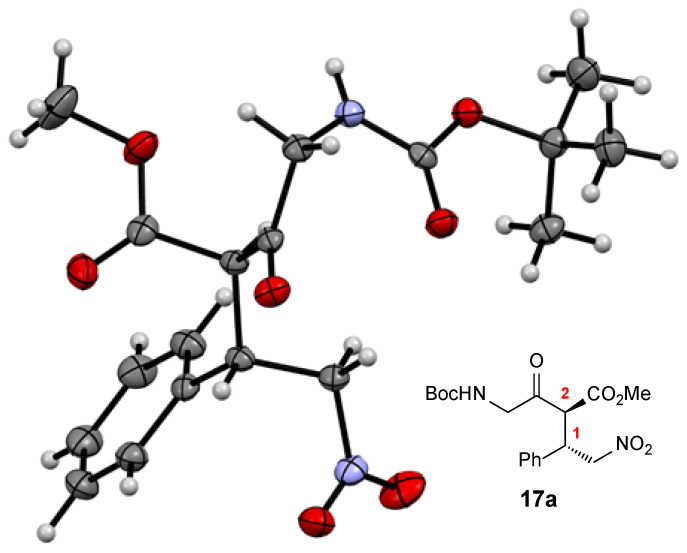
Molecular structure of the (1*S*,2*S*)-stereoisomer of product **17a**, synthesized using catalyst **I**. Thermal ellipsoids are shown at 50% probability.

*Structure determination.* The structures of novel compounds **2f**, **2h**, **2i**, **4**–**8**, **16**–**18**, and **20** were confirmed by spectroscopic methods (^1^H and ^13^C NMR, IR, and high-resolution mass spectrometry), while compound **15** was used as a crude product for further transformations; its structure was confirmed by ^1^H NMR. The diastereomers of compounds **17**/***rac*-17** and **18**/***rac*-18** could not be separated by column chromatography and were characterized as mixtures of diastereomers. The diastereomeric ratios of compounds **17**/***rac*-17** and **18**/***rac*-18** were determined using proton spectra, in which the non-overlapping signals were integrated. Similarly, the keto–enol ratios of compounds **2**, **10**, **11**, **17**/***rac*-17**, and **20** were determined from proton spectra. β-Keto esters **2**, **10**, and **17**/***rac*-17** contain up to 17% (17% for compound **2i**, see ESI) of the enol form, as indicated by the enol signal in the proton spectra (a singlet) at approximately 11.5–13.5 ppm. For details, see the ESI. In contrast, the enol form is the predominant tautomer for the pyrrolones **11a**–**c** in DMSO-*d*_6_ (ranging from 55% for **11a′** to 63% for **11b′**; see Scheme 2) [22,23]. The tetronic acid derivative **20** exists in CDCl_3_ solution exclusively in the enol form, as indicated by the enol-ester resonances in the ^13^C NMR spectra at 99.1 ppm, 177.4 ppm, and 177.9 ppm, and by the absence of a ketone signal above 200 ppm (see Scheme 5 and the ESI). The (*E*) configuration around the C=C bond of compound **16** was assigned based on the vicinal R**H**C=C**H**NO_2_ coupling constant (^3^*J* = 13.4 Hz). The structure and (1*S*,2*S*)-absolute configuration of the stereoisomer of adduct **17a** (prepared with organocatalyst **I**) were determined by single-crystal X-ray diffraction analysis (Figure 1). The (1*S*)-absolute configuration is consistent with our previous findings [21]. Based on this, we assigned the (1*R*)-absolute configuration to the major enantiomer of both diastereomers of products **17a**, **17b**, **17d**, **17f**, **17h**, **17j**, and **17l** prepared with organocatalyst **VIII**; the results are fully consistent with the stereochemistry findings of the seminal paper by Viresh H. Rawal, who first prepared and applied organocatalyst **VIII** in the Michael addition of 1,3-dicarbonyl nucleophiles to *trans*-β-nitrostyrene acceptors [57]. The absolute configuration at the C-2 chiral center is labile due to rapid keto–enol tautomerization. The (1′*S*,5*S*)-absolute configuration of the major diastereomer of product **18a** (see Scheme 5) was assigned based on our previous results in the series of 1,4-adducts of pyrrolones to nitroalkene acceptors [23].

**Figure 2:**
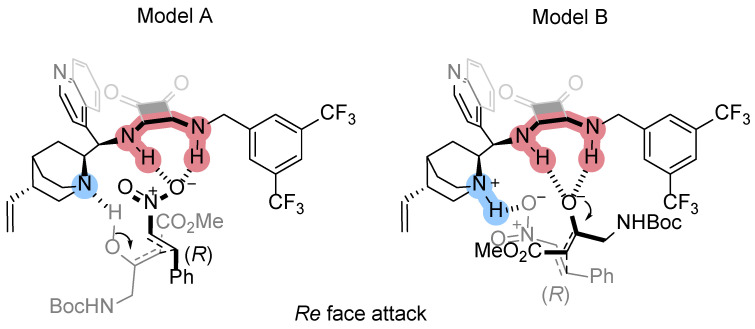
Two possible transition state models for the asymmetric Michael addition reaction **2a** + **12** → **17a**, based on the absolute configuration of product **17a**.

*Proposed mechanism for the 1,4-addition of β-keto esters.* Possible transition-state variants of the bifunctional mechanisms of cinchona-derived squaramide and thiourea organocatalysts have been studied previously. The proposed transition states are based on literature data for similar reactions and on the absolute configuration of product **17a**. Since the formation of product **17a** with organocatalyst **VIII** must occur through *Re* face attack of β-keto ester **2a** on *trans*-β-nitrostyrene (**12**), two possible transition states are proposed (Figure 2). The difference between the two transition states lies in the mode of activation. In Model A, proposed by Takemoto et al., the nucleophile is directed by the amine group, while the electrophile is activated by the squaramide NH [58]. In Model B, proposed by Pápai et al., the nucleophile is directed by the squaramide NH, and the electrophile is activated by the protonated amine group [59,60,61]. The orientation of the nucleophile is irrelevant, as it determines only the absolute configuration at the epimerizable C-2 stereocenter.

## 3. Materials and Methods

Solvents for extractions and chromatography were of technical grade and were distilled prior to use. Extracts were dried over technical grade anhydrous Na_2_SO_4_. Melting points were determined on a Kofler micro hot stage and on SRS OptiMelt MPA100—Automated Melting Point System (Stanford Research Systems, Sunnyvale, CA, USA). The NMR spectra were obtained on a Bruker UltraShield 500 plus spectrometer and on a BRUKER AVANCE NEO 600 MHz NMR spectrometer (Bruker, Billerica, MA, USA) at 500 and 600 MHz for ^1^H and 126 and 150 MHz for ^13^C nuclei, respectively, using DMSO-*d*_6_ and CDCl_3_ with TMS as the internal standard, as solvents. Mass spectra were recorded on an Agilent 6224 Accurate Mass TOF LC/MS and Agilent 6530 Q-TOF LC/MS coupled with Agilent 1260 Infinity2 HPLC (Agilent Technologies, Santa Clara, CA, USA), IR spectra on a Perkin-Elmer Spectrum BX FTIR spectrophotometer (PerkinElmer, Waltham, MA, USA). Column chromatography (CC) was performed on silica gel (Silica gel 60, particle size: 0.035–0.070 mm (Sigma-Aldrich, St. Louis, MO, USA)). HPLC analyses were performed on an Agilent 1260 Infinity LC (Agilent Technologies, Santa Clara, CA, USA) using CHIRALPAK IA-3 (0.46 cm ø × 25 cm), CHIRALPAK AD-H (0.46 cm ø × 25 cm), CHIRALCEL OD-H (0.46 cm ø × 25 cm), and CHIRALPAK AS-H (0.46 cm ø × 25 cm) as chiral column (CHIRAL TECHNOLOGIES, INC., West Chester, PA, USA). All the commercially available chemicals used were purchased from Sigma-Aldrich (St. Louis, MO, USA).

Organocatalysts **I** [21], **II** [62], **III** [63], **IV** [63], **VI** [64], **VII** [65], **VIII** [57], and **IX** [66] were prepared following the literature procedures; organocatalyst **V** was purchased from Sigma-Aldrich.

### 3.1. Synthesis of β-Keto Esters ***2*** from Carboxylic Acids ***1***—General Procedure 1 (GP1)

To a solution or suspension of carboxylic acid **1** (10 mmol) in anhydrous THF (50 mL), 1,1′-carbonyldiimidazole (CDI; 12 mmol, ω = 0.97, 1.672 g) was added under argon, and the resulting reaction mixture was stirred for 2 h at room temperature. A solid mixture of MgCl_2_ (9.8 mmol, ω = 0.98, 952 mg) and methyl potassium malonate (15 mmol, 2.343 g) or *tert*-butyl potassium malonate (15 mmol, ω = 0.95, 3.130 g) was then added. The reaction mixture was stirred for a further 24 h under argon at room temperature. The volatiles were evaporated in vacuo, the residue was dissolved in EtOAc (150 mL) and washed with NaHSO_4_ (1 M in H_2_O, 3 × 50 mL), NaHCO_3_ (aq. sat., 2 × 20 mL), and NaCl (aq. sat., 2 × 50 mL). The organic phase was dried over anhydrous Na_2_SO_4_, filtered, and the volatiles evaporated in vacuo. If necessary, the residue was purified by column chromatography (CC, Silica gel 60). The fractions containing the product were combined, and the volatiles were evaporated in vacuo.

### 3.2. Synthesis of Methyl 4-((Tert-butoxycarbonyl)amino)-3-oxobutanoate (***2a***) [22]

Following *GP1*, prepared from (*tert*-butoxycarbonyl)glycine (**1a**) (10 mmol, 1.752 g), methyl potassium malonate (15 mmol, 2.343 g); isolation by extraction. Yield: 2.10 g (9.1 mmol, 91%) of yellowish oil. ^1^H-NMR (500 MHz, DMSO-*d*_6_): *δ* 1.39 (*s*, 9H), 3.60 (*s*, 2H), 3.63 (*s*, 3H), 3.86 (*d*, *J* = 5.9 Hz, 2H), 7.13 (*t*, *J* = 5.9 Hz, 1H). ^13^C-NMR (126 MHz, DMSO-*d*_6_): *δ* 28.18, 45.91, 49.81, 51.93, 78.30, 155.81, 167.50, 200.67.

### 3.3. Synthesis of Methyl 5-((Tert-butoxycarbonyl)amino)-3-oxopentanoate (***2b***) [22]

Following *GP1*, prepared from Boc-β-alanine (**1b**) (10 mmol, 1.892 g), methyl potassium malonate (15 mmol, 2.343 g); isolation by extraction. Yield: 2.18 g (8.9 mmol, 89%) of yellowish oil. ^1^H-NMR (500 MHz, DMSO-*d*_6_): *δ* 1.36 (*s*, 9H), 2.66 (*t*, *J* = 6.9 Hz, 2H), 3.11 (*q*, *J* = 6.9 Hz, 2H), 3.61 (*s*, 2H), 3.62 (*s*, 3H), 6.77 (*t*, *J* = 5.6 Hz, 1H). ^13^C-NMR (126 MHz, DMSO-*d*_6_): *δ* 28.24, 34.88, 42.40, 48.68, 51.84, 77.72, 155.53, 167.71, 202.42.

### 3.4. Synthesis of Methyl 4-((3-Methylbut-2-en-1-yl)oxy)-3-oxobutanoate (***2c***) [67]

Following *GP1*, prepared from 2-((3-methylbut-2-en-1-yl)oxy)acetic acid (**1c**) [68] (10 mmol, 1.442 g), methyl potassium malonate (15 mmol, 2.343 g); isolation by extraction. Yield: 1.602 g (8.0 mmol, 80%) of colorless oil. ^1^H-NMR (500 MHz, CDCl_3_): *δ* 1.69 (*s*, 3H), 1.77 (*s*, 3H), 3.55 (*s*, 2H), 3.74 (*s*, 3H), 4.04 (*d*, *J* = 7.1 Hz, 2H), 4.09 (*s*, 2H), 5.29–5.36 (*m*, 1H). ^13^C-NMR (126 MHz, CDCl_3_): *δ* 18.11, 25.88, 45.77, 52.41, 67.89, 74.63, 119.97, 138.70, 167.62, 202.30.

### 3.5. Synthesis of Methyl 3-Oxooctadecanoate (***2d***) [69]

Following *GP1*, prepared from palmitic acid (**1d**) (10 mmol, 2.564 g), methyl potassium malonate (15 mmol, 2.343 g); isolation by extraction and column chromatography (EtOAc/petroleum ether = 1:10). Yield: 2717 g (8.70 mmol, 87%) of white solid; m.p. = 49.2–50.5 °C. ^1^H-NMR (500 MHz, CDCl_3_): *δ* 0.88 (*t*, *J* = 6.9 Hz, 3H), 1.19–1.35 (*m*, 24H), 1.53–1.65 (*m*, 2H), 2.53 (*t*, *J* = 7.4 Hz, 2H), 3.45 (*s*, 2H), 3.74 (*s*, 3H). ^13^C-NMR (126 MHz, CDCl_3_): *δ* 14.26, 22.83, 23.60, 29.14, 29.49, 29.50, 29.58, 29.73, 29.77, 29.79, 29.80, 29.82, 29.83, 32.06, 43.23, 49.15, 52.46, 167.85, 203.01.

### 3.6. Synthesis of Methyl 3-Oxoicosanoate (***2e***) [69]

Following *GP1*, prepared from stearic acid (**1e**) (10 mmol, 2.845 g), methyl potassium malonate (15 mmol, 2.343 g); isolation by extraction and column chromatography (EtOAc/petroleum ether = 1:10). Yield: 2.282 g (6.70 mmol, 67%) of white solid; m.p. = 49.5–51.2 °C. ^1^H-NMR (500 MHz, CDCl_3_): *δ* 0.88 (*t*, *J* = 6.9 Hz, 3H), 1.18–1.34 (*m*, 28H), 1.55–1.63 (*m*, 2H), 2.53 (*t*, *J* = 7.4 Hz, 2H), 3.45 (*s*, 2H), 3.74 (*s*, 3H). ^13^C-NMR (151 MHz, CDCl_3_): *δ* 14.25, 22.83, 23.60, 29.14, 29.49, 29.50, 29.58, 29.73, 29.78, 29.79, 29.80, 29.83, 32.06, 43.22, 49.14, 52.45, 167.84, 203.00 (3 signals missing due to overlapping).

### 3.7. Synthesis of Tert-Butyl 3-Oxoicosanoate (***2f***)

Following *GP1*, prepared from stearic acid (**1e**) (10 mmol, 2.845 g), *tert*-butyl potassium malonate (15 mmol, ω = 0.95, 3.130 g); isolation by extraction and column chromatography (EtOAc/petroleum ether = 1:15). Yield: 1.722 g (4.50 mmol, 45%) of white solid; m.p. = 36.9–38.1 °C. EI-HRMS: *m*/*z* = 327.2885 (MH^+^-*t*BuOH); C_20_H_39_O_3_ requires *m*/*z* = 327.2894 (MH^+^-*t*BuOH); *ν*_max_ 2960, 2916, 2849, 1729, 1715, 1466, 1406, 1367, 1329, 1276, 1260, 1155, 1131, 1109, 1080, 947, 920, 842, 790, 723, 647 cm^−1^. ^1^H-NMR (500 MHz, CDCl_3_): *δ* 0.88 (*t*, *J* = 6.9 Hz, 3H), 1.21–1.34 (*m*, 28H), 1.47 (*s*, 9H), 1.55–1.61 (*m*, 2H), 2.51 (*t*, *J* = 7.4 Hz, 2H), 3.34 (*s*, 2H). ^13^C-NMR (151 MHz, CDCl_3_): *δ* 14.26, 22.83, 23.62, 28.10, 29.21, 29.50, 29.52, 29.59, 29.74, 29.78, 29.80, 29.81, 29.84, 32.07, 43.09, 50.81, 81.99, 166.69, 203.68 (3 signals missing due to overlapping).

### 3.8. Synthesis of Methyl (11Z,14Z)-3-Oxoicosa-11,14-dienoate (***2g***) [69]

Following *GP1*, prepared from linoleic acid (**1f**) (10 mmol, 2.804 g), methyl potassium malonate (15 mmol, 2.343 g); isolation by extraction and column chromatography (EtOAc/petroleum ether = 1:5). Yield: 1.851 g (5.50 mmol, 55%) of colorless oil. ^1^H-NMR (500 MHz, CDCl_3_): *δ* 0.89 (*t*, *J* = 6.9 Hz, 3H), 1.23–1.40 (*m*, 14H), 1.54–1.64 (*m*, 2H), 1.97–2.09 (*m*, 4H), 2.53 (*t*, *J* = 7.4 Hz, 2H), 2.77 (*t*, *J* = 6.6 Hz, 2H), 3.45 (*s*, 2H), 3.74 (*s*, 3H), 5.28–5.43 (*m*, 4H). ^13^C-NMR (126 MHz, CDCl_3_): *δ* 14.21, 22.71, 23.57, 25.76, 27.31, 27.33, 29.10, 29.21, 29.39, 29.48, 29.72, 31.66, 43.20, 49.15, 52.46, 128.02, 128.19, 130.15, 130.35, 167.83, 202.95.

### 3.9. Synthesis of Methyl (S)-4-((Tert-butoxycarbonyl)amino)-3-oxo-5-phenylpentanoate (***10a***) [22]

Following *GP1*, prepared from Boc-L-phenylalanine (**9a**) (10 mmol, 2.653 g), methyl potassium malonate (15 mmol, 2.343 g); isolation by extraction. Yield: 2.346 g (7.30 mmol, 73%) of colorless oil. ^1^H-NMR (500 MHz, CDCl_3_): *δ* 1.40 (*s*, 9H), 2.98 (*dd*, *J* = 7.5, 14.1 Hz, 1H), 3.14 (*dd*, *J* = 6.2, 14.1 Hz, 1H), 3.46 (*d*, *J* = 16.0 Hz, 1H), 3.52 (*d*, *J* = 16.0 Hz, 1H), 3.71 (*s*, 3H), 4.56 (*q*, *J* = 7.2 Hz, 1H), 4.96–5.07 (*m*, 1H), 7.14–7.20 (*m*, 2H), 7.21–7.35 (*m*, 3H).

### 3.10. Synthesis of Methyl (S)-8-(((Benzyloxy)carbonyl)amino)-4-((tert-butoxycarbonyl)amino)-3-oxooctanoate (***10b***) [22]

Following *GP1*, prepared from Boc-Lys(Z)-OH (**9b**) (10 mmol, 3.804 g), methyl potassium malonate (15 mmol, 2.343 g); isolation by extraction. Yield: 3.317 g (7.60 mmol, 76%) of colorless oil. ^1^H-NMR (500 MHz, CDCl_3_): *δ* 1.30–1.65 (*m*, 5H), 1.43 (*s*, 9H), 1.81–1.93 (*m*, 1H), 3.12–3.27 (*m*, 2H), 3.54 (*d*, *J* = 15.7 Hz, 1H), 3.59 (*d*, *J* = 16.0 Hz, 1H), 3.73 (*s*, 3H), 4.27–4.36 (*m*, 1H), 4.93 (*t*, *J* = 6.0 Hz, 1H), 5.05–5.17 (*m*, 2H), 5.27 (br *d*, *J* = 7.7 Hz, 1H), 7.28–7.39 (*m*, 5H).

### 3.11. Synthesis of 7-Benzyl 1-Methyl (S)-4-((Tert-butoxycarbonyl)amino)-3-oxoheptanedioate (***10c***) [23]

Following *GP1*, prepared from Boc-Glu(OBzl)-OH (**9c**) (10 mmol, 3.374g), methyl potassium malonate (15 mmol, 2.343 g); isolation by extraction. Yield: 2.675 g (6.80 mmol, 68%) of colorless oil. ^1^H-NMR (500 MHz, CDCl_3_): *δ* 1.43 (*s*, 9H), 1.80–1.91 (*m*, 1H), 2.22–2.32 (*m*, 1H), 2.38–2.56 (*m*, 2H), 3.55–3.65 (*m*, 2H), 3.73 (*s*, 3H), 4.38–4.45 (*m*, 1H), 5.12 (*s*, 2H), 5.23 (br *d*, *J* = 8.1 Hz, 1H), 7.29–7.41 (*m*, 5H).

### 3.12. Synthesis of Methyl (S)-5-((Tert-butoxycarbonyl)amino)-2-stearamidopentanoate (***4***)

To a solution of stearic acid (**1e**) (20 mmol, 5.690 g) in anhydrous THF (50 mL) was added 1,1′-carbonyldiimidazole (CDI; 22 mmol, ω = 0.97, 3.678 g) under argon, and the resulting reaction mixture was stirred for 2 h at room temperature. Then, H-Orn(Boc)-OMe×HCl (**3**) (22 mmol, ω = 0.96, 6.480 g) and Et_3_N (22 mmol, 3.07 mL) were added. The reaction mixture was stirred for a further 24 h under argon at room temperature. The volatiles were evaporated in vacuo; the residue was dissolved in CH_2_Cl_2_ (150 mL) and washed with NaHSO_4_ (1 M in H_2_O, 4 × 50 mL), NaHCO_3_ (aq. sat., 3 × 20 mL) and NaCl (aq. sat., 2 × 50 mL). The organic phase was dried over anhydrous Na_2_SO_4_, filtered, and the volatiles evaporated in vacuo. Yield: 7.076 g (13.8 mmol, 69%) of white solid; m.p. = 86.0–88.0 °C. EI-HRMS: *m*/*z* = 513.4272 (MH^+^); C_29_H_57_N_2_O_5_ requires *m*/*z* = 513.4262 (MH^+^); *ν*_max_ 3345, 2915, 2847, 1762, 1684, 1648, 1526, 1473, 1462, 1369, 1285, 1252, 1212, 1171, 1143, 1047, 994, 950, 871, 754, 729, 719, 654 cm^−1^. ^1^H-NMR (500 MHz, DMSO-*d*_6_): *δ* 0.85 (*t*, *J* = 6.8 Hz, 3H), 1.14–1.31 (*m*, 30H), 1.37 (*s*, 9H), 1.42–1.57 (*m*, 3H), 1.61–1.69 (*m*, 1H), 2.09 (*t*, *J* = 7.1 Hz, 2H), 2.89 (*q*, *J* = 6.8 Hz, 2H), 3.60 (*s*, 3H), 4.14–4.23 (*m*, 1H), 6.79 (*t*, *J* = 5.7 Hz, 1H), 8.14 (*d*, *J* = 7.5 Hz, 1H). ^13^C-NMR (126 MHz, DMSO-*d*_6_): *δ* 13.98, 22.11, 25.23, 26.02, 28.20, 28.26, 28.55, 28.72, 28.78, 28.97, 29.02, 29.05, 31.31, 34.94, 51.65, 51.70, 77.40, 155.58, 172.43, 172.78 (7 signals missing due to overlapping).

### 3.13. Synthesis of (S)-5-((Tert-butoxycarbonyl)amino)-2-stearamidopentanoic Acid (***5***)

To a solution/suspension of methyl (*S*)-5-((*tert*-butoxycarbonyl)amino)-2-stearamidopentanoate (**4**) (1.70 mmol, 872 mg) in a mixture of H_2_O (3.0 mL) and THF (3.0 mL) was added NaOH (15.0 mmol, 600 mg). The reaction mixture was stirred for 3 h at room temperature. The mixture was acidified with HCl (aq. 1 M) to pH < 3 and extracted with EtOAc (3 × 30 mL). The combined organic layers were washed with brine (1 × 10 mL), dried over Na_2_SO_4_, filtered and the volatiles evaporated in vacuo. The residue was azeotropically evaporated with CHCl_3_ (3 × 30 mL) to give the anhydrous product **5**. Yield: 763 mg (1.53 mmol, 90%) of white solid; m.p. = 82.0–84.3 °C. EI-HRMS: *m*/*z* = 499.4113 (MH^+^); C_28_H_55_N_2_O_5_ requires *m*/*z* = 499.4105 (MH^+^); *ν*_max_ 3359, 2955, 2916, 2849, 1738, 1682, 1605, 1525, 1465, 1454, 1388, 1365, 1290, 1274, 1244, 1210, 1170, 1112, 1043, 1019, 957, 890, 860, 783, 727, 637 cm^−1^. ^1^H-NMR (500 MHz, CDCl_3_): *δ* 0.88 (*t*, *J* = 6.9 Hz, 3H), 1.17–1.36 (*m*, 28H), 1.44 (*s*, 9H), 1.52–1.67 (*m*, 4H), 1.68–1.79 (*m*, 1H), 1.87–1.98 (*m*, 1H), 2.25 (*t*, *J* = 7.8 Hz, 2H), 3.04–3.27 (*m*, 2H), 4.60 (*td*, *J* = 4.9, 7.5 Hz, 1H), 4.86 (*t*, *J* = 6.4 Hz, 1H), 6.73 (br *d*, *J* = 7.4 Hz, 1H), 9.64 (br *s*, 1H). ^13^C-NMR (126 MHz, CDCl_3_): *δ* 14.27, 22.84, 25.79, 26.56, 28.52, 29.06, 29.42, 29.49, 29.51, 29.67, 29.80, 29.83, 29.86, 32.07, 36.57, 39.88, 52.22, 79.99, 156.91, 174.55, 174.80 (5 signals missing due to overlapping). ^1^H-NMR (500 MHz, DMSO-*d*_6_): *δ* 0.85 (*t*, *J* = 6.9 Hz, 3H), 1.06–1.31 (*m*, 29H), 1.37 (*s*, 9H), 1.32–1.56 (*m*, 4H), 1.61–1.71 (*m*, 1H), 2.04–2.15 (*m*, 2H), 2.84–2.94 (*m*, 2H), 4.13 (*td*, *J* = 5.0, 8.5 Hz, 1H), 6.77 (*t*, *J* = 5.8 Hz, 1H), 7.98 (*d*, *J* = 7.8 Hz, 1H), 12.40 (br *s*, 1H). ^13^C-NMR (126 MHz, DMSO-*d*_6_): *δ* 13.91, 22.06, 25.24, 26.15, 28.23, 28.43, 28.57, 28.66, 28.77, 28.93, 28.97, 29.00, 31.26, 35.02, 51.54, 77.33, 155.54, 172.23, 173.71 (7 signals missing due to overlapping).

### 3.14. Synthesis of Methyl (S)-7-((Tert-butoxycarbonyl)amino)-3-oxo-4-stearamidoheptanoate (***2h***)

Following *GP1*, prepared from (*S*)-5-((*tert*-butoxycarbonyl)amino)-2-stearamidopentanoic acid (**5**) (1.5 mmol, 748 mg), CDI (1.8 mmol, ω = 0.97, 301 mg), MgCl_2_ (1.47 mmol, ω = 0.98, 143 mg), methyl potassium malonate (2.25 mmol, 351 mg); isolation by extraction and column chromatography (EtOAc/petroleum ether = 1:1). Yield: 591 mg (1.065 mmol, 71%) of white solid; m.p. = 62.3–64.9 °C. EI-HRMS: *m*/*z* = 555.4384 (MH^+^); C_31_H_59_N_2_O_6_ requires *m*/*z* = 555.4368 (MH^+^); *ν*_max_ 3341, 2915, 2848, 1747, 1711, 1681, 1638, 1524, 1438, 1390, 1365, 1316, 1251, 1168, 1016, 886, 769, 719, 643 cm^−1^. ^1^H-NMR (500 MHz, CDCl_3_): *δ* 0.88 (*t*, *J* = 6.9 Hz, 3H), 1.18–1.35 (*m*, 28H), 1.44 (*s*, 9H), 1.47–1.68 (*m*, 5H), 1.90–2.00 (*m*, 1H), 2.21–2.26 (*m*, 2H), 3.15 (*q*, *J* = 6.7 Hz, 2H), 3.58 (*s*, 2H), 3.74 (*s*, 3H), 4.66 (br *s*, 1H), 4.68–4.74 (*m*, 1H), 6.43 (br *d*, *J* = 7.4 Hz, 1H). ^13^C-NMR (126 MHz, CDCl_3_): *δ* 14.27, 22.83, 25.73, 26.44, 27.58, 28.52, 29.44, 29.47, 29.50, 29.63, 29.77, 29.80, 29.84, 32.06, 36.62, 39.87, 46.26, 52.66, 58.11, 79.56, 156.40, 167.45, 173.54, 201.86 (6 signals missing due to overlapping). ^1^H-NMR (500 MHz, DMSO-*d*_6_): *δ* 0.85 (*t*, *J* = 6.9 Hz, 3H), 1.08–1.30 (*m*, 29H), 1.37 (*s*, 9H), 1.31–1.54 (*m*, 4H), 1.62–1.75 (*m*, 1H), 2.12 (*t*, *J* = 7.4 Hz, 2H), 2.89 (*q*, *J* = 6.2 Hz, 2H), 3.58 (*s*, 2H), 3.61 (*s*, 3H), 4.23 (*ddd*, *J* = 4.5, 7.3, 9.5 Hz, 1H), 6.78 (*t*, *J* = 5.9 Hz, 1H), 8.16 (*d*, *J* = 7.4 Hz, 1H). ^13^C-NMR (126 MHz, DMSO-*d*_6_): *δ* 13.90, 22.05, 25.10, 25.88, 26.25, 28.22, 28.57, 28.65, 28.71, 28.89, 28.96, 28.99, 31.25, 34.91, 45.49, 51.76, 57.75, 77.36, 155.57, 167.46, 172.67, 202.73 (7 signals missing due to overlapping).

### 3.15. Synthesis of (S)-5-Methoxy-5-oxo-4-stearamidopentan-1-aminium 2,2,2-Trifluoroacetate (***6***)

Methyl (*S*)-5-((*tert*-butoxycarbonyl)amino)-2-stearamidopentanoate (**4**) (10 mmol, 5.128 g) was dissolved in a 1:1 mixture of CF_3_COOH and anhydrous CH_2_Cl_2_ (60 mL) under argon, and the reaction mixture was stirred for 3 h at room temperature. Volatile components were evaporated in vacuo, and the residue was azeotropically evaporated with anhydrous toluene (3 × 100 mL) to give ammonium salt **6**. Yield: 5.00 g (9.50 mmol, 95%) of white solid; m.p. = 93.0–95.7 °C. EI-HRMS: *m*/*z* = 413.3726 (MH^+^); C_24_H_49_N_2_O_3_^+^ requires *m*/*z* = 413.3738 (MH^+^); *ν*_max_ 3318, 2915, 2848, 1752, 1671, 1645, 1528, 1474, 1462, 1430, 1400, 1381, 1358, 1276, 1237, 1207, 1173, 1127, 1067, 1003, 970, 955, 893, 839, 800, 768, 747, 723, 668, 613 cm^−1^. ^1^H-NMR (500 MHz, CDCl_3_ (700 μL) + TFA (20 μL)): *δ* 0.88 (*t*, *J* = 6.9 Hz, 3H), 1.09–1.35 (*m*, 26H), 1.53–1.63 (*m*, 2H), 1.69–1.85 (*m*, 3H), 1.91–2.01 (*m*, 1H), 2.23–2.33 (*m*, 2H), 3.01–3.20 (*m*, 2H), 3.76 (*s*, 3H), 4.48–4.57 (*m*, 1H), 6.86 (*d*, *J* = 7.5 Hz, 1H), 7.61 (br *s*, 3H). ^13^C-NMR (126 MHz, CDCl_3_ (700 μL) + TFA (20 μL)): *δ* 14.25, 22.84, 23.37, 25.78, 29.22, 29.28, 29.31, 29.51, 29.59, 29.74, 29.80, 29.81, 29.86, 32.07, 36.22, 39.62, 51.65, 53.08, 115.46 (*q*, *J* = 287.8 Hz), 161.08 (*q*, *J* = 39.3 Hz), 172.10, 176.30 (3 signals missing due to overlapping).

### 3.16. Synthesis of Methyl (S)-2,5-Distearamidopentanoate (***7***)

To a solution of stearic acid (**1e**) (8.5 mmol, 2.416 g) in anhydrous THF (30 mL), 1,1′-carbonyldiimidazole (CDI; 9.35 mmol, ω = 0.97, 1.563 g) was added under argon, and the resulting reaction mixture was stirred for 2 h at room temperature. The resulting activated acid was transferred to a suspension of (*S*)-5-methoxy-5-oxo-4-stearamidopentan-1-aminium 2,2,2-trifluoroacetate (**6**) (9.35 mmol, 4.924 g) in anhydrous THF (30 mL) under argon. Et_3_N (9.35 mmol, 1.303 mL) was then added to the reaction mixture at room temperature. The reaction mixture was stirred at 40 °C for 12 h. Volatile components were evaporated in vacuo. The residue was extracted with CH_2_Cl_2_ (100 mL), EtOAc (100 mL), Et_2_O (100 mL), and *n*-hexane (100 mL) using a laboratory ultrasonic bath (5 min each), followed by decanting, respectively, to remove unreacted starting material and small portions of the product. H_2_O (150 mL) was added to the residue, followed by ultrasonic bath treatment (15 min). The resulting precipitate was collected by filtration and thoroughly washed with H_2_O (3 × 70 mL). The residue was dried under high vacuum at 40 °C for 12 h to give product **7**. Yield: 3.476 g (5.270 mmol, 62%) of white solid; m.p. = 106.7–108.4 °C. EI-HRMS: *m*/*z* = 679.6336 (MH^+^); C_42_H_83_N_2_O_4_ requires *m*/*z* = 679.6347 (MH^+^); *ν*_max_ 3305, 2914, 2848, 1742, 1639, 1542, 1470, 1420, 1385, 1277, 1259, 1239, 1205, 1173, 980, 717 cm^−1^. ^1^H-NMR (500 MHz, CDCl_3_ (700 μL) + TFA (20 μL)): *δ* 0.88 (*t*, *J* = 6.9 Hz, 6H), 1.18–1.36 (*m*, 56H), 1.54–1.77 (*m*, 7H), 1.87–1.97 (*m*, 1H), 2.26–2.38 (*m*, 4H), 3.23–3.34 (*m*, 1H), 3.34–3.47 (*m*, 1H), 3.79 (*s*, 3H), 4.57–4.64 (*m*, 1H), 6.70 (*t*, *J* = 6.1 Hz, 1H), 6.79 (*d*, *J* = 7.7 Hz, 1H). ^13^C-NMR (126 MHz, CDCl_3_ (700 μL) + TFA (20 μL)): *δ* 14.25, 22.84, 24.97, 25.87, 25.99, 29.20, 29.23, 29.28, 29.51, 29.56, 29.72, 29.78, 29.81, 29.83, 29.85, 30.00, 32.07, 36.28, 36.35, 39.58, 52.17, 53.15, 172.43, 176.50, 177.11 (17 signals missing due to overlapping).

### 3.17. Synthesis of (S)-2,5-Distearamidopentanoic Acid (***8***)

To a suspension of methyl (*S*)-2,5-distearamidopentanoate (**7**) (5 mmol, 3.393 g) in a mixture of H_2_O (20 mL) and THF (5 mL), KOH (powder for synthesis, 50 mmol, 2.810 g) was added, and the reaction mixture was stirred at 90 °C for 12 h. The mixture was cooled to room temperature, and HCl (aq., 2 M) was added under stirring until the pH reached 1–2. The precipitate was collected by filtration and thoroughly washed with H_2_O (3 × 100 mL). The residue was dried under high vacuum at 40 °C for 12 h to give acid **8**. Yield: 2.792 g (4.20 mmol, 84%) of white solid; m.p. = 108.8–110.1 °C. EI-HRMS: *m*/*z* = 665.6191 (MH^+^); C_41_H_81_N_2_O_4_ requires *m*/*z* = 665.6191 (MH^+^); *ν*_max_ 3310, 2955, 2916, 2849, 1736, 1639, 1586, 1545, 1466, 1446, 1418, 1372, 1275, 1245, 1211, 1181, 1128, 970, 829, 720, 685, 633 cm^−1^. ^1^H-NMR (500 MHz, CDCl_3_ (700 μL) + TFA (20 μL)): *δ* 0.88 (*t*, *J* = 6.8 Hz, 6H), 1.09–1.40 (*m*, 56H), 1.52–1.73 (*m*, 6H), 1.75–1.89 (*m*, 1H), 1.92–2.11 (*m*, 1H), 2.27–2.45 (*m*, 4H), 3.13–3.49 (*m*, 2H), 4.61 (*q*, *J* = 6.7, 1H), 6.87 (*s*, 1H), 6.98 (*d*, *J* = 7.3 Hz, 1H), 11.12 (br *s*, 1H). ^13^C-NMR (126 MHz, CDCl_3_ (700 μL) + TFA (20 μL)): *δ* 14.26, 22.84, 24.97, 25.88, 25.96, 29.14, 29.19, 29.21, 29.25, 29.27, 29.52, 29.55, 29.57, 29.73, 29.79, 29.82, 29.84, 29.86, 32.08, 36.09, 36.13, 39.82, 52.36, 175.92, 177.30, 177.72 (15 signals missing due to overlapping).

### 3.18. Synthesis of Methyl (S)-3-Oxo-4,7-distearamidoheptanoate (***2i***)

To a suspension of (*S*)-2,5-distearamidopentanoic acid (**8**) (2.5 mmol, 1.662 g) in anhydrous THF (25 mL), 1,1′-carbonyldiimidazole (CDI; 5 mmol, ω = 0.97, 836 mg) was added under argon, and the reaction mixture was stirred for 15 min at room temperature, 30 min at 90 °C, and 60 min at 50 °C. Then, a solid mixture of MgCl_2_ (2.5 mmol, ω = 0.98, 243 mg) and methyl potassium malonate (7.5 mmol, 1.171 g) was carefully added at 50 °C. The reaction mixture was stirred for 15 min at 50 °C, 30 min at 90 °C, and 24 h at room temperature. The volatiles were evaporated in vacuo, and NaHSO_4_ (1 M in H_2_O, 100 mL) was added to the residue. The mixture was stirred at room temperature for 30 min. The precipitate was collected by filtration and thoroughly washed with H_2_O (3 × 100 mL). The residue was dried under high vacuum at 40 °C for 12 h to give β-keto ester **2i**. Yield: 1.099 g (1.525 mmol, 61%) of white solid; m.p. = 92.1–94.7 °C. EI-HRMS: *m*/*z* = 721.6456 (MH^+^); C_44_H_85_N_2_O_5_ requires *m*/*z* = 721.6453 (MH^+^); *ν*_max_ 3306, 2916, 2849, 1748, 1717, 1638, 1539, 1463, 1378, 1328, 1258, 1239, 1223, 1204, 1147, 1013, 719 cm^−1^. ^1^H-NMR (600 MHz, CDCl_3_): *δ* 0.88 (*t*, *J* = 6.9 Hz, 6H), 1.18–1.39 (*m*, 56H), 1.51–1.67 (*m*, 7H), 1.88–1.97 (*m*, 1H), 2.17 (*t*, *J* = 7.7 Hz, 2H), 2.25 (*t*, *J* = 7.7 Hz, 2H), 3.22–3.38 (*m*, 2H), 3.58 (*d*, *J* = 1.7 Hz, 2H), 3.74 (*s*, 3H), 4.65–4.72 (*m*, 1H), 5.87 (*t*, *J* = 6.0 Hz, 1H), 6.67 (*d*, *J* = 7.5 Hz, 1H). ^13^C-NMR (151 MHz, CDCl_3_): *δ* 14.26, 22.83, 25.72, 25.94, 25.98, 27.70, 29.45, 29.50, 29.52, 29.66, 29.78, 29.80, 29.81, 29.85, 32.07, 36.56, 36.96, 38.86, 46.22, 52.66, 58.21, 167.58, 173.81, 173.95, 201.90 (19 signals missing due to overlapping).

### 3.19. Synthesis of Pyrrolones ***11*** from β-Keto Esters ***10***—General Procedure 2 (GP2)

To a solution of β-keto ester **10** (1.0 mmol) in anhydrous toluene (5 mL), DMFDMA (3 mmol, ω = 0.94, 424 μL) was added under argon and the resulting reaction mixture was stirred at 70 °C under argon until completion of the reaction, as judged by TLC analysis (1–3 h). The volatiles were evaporated in vacuo, and the residue was purified as quickly as possible by column chromatography (CC, Silica gel 60). The fractions containing the product **11** were combined, and the volatiles were evaporated in vacuo. The product was immediately used for the following transformation and/or stored under argon at −20 °C.

### 3.20. Synthesis of 1-(Tert-butyl) 3-Methyl 5-Benzyl-4-oxo-4,5-dihydro-1H-pyrrole-1,3-dicarboxylate (***11a***) and 1-(Tert-butyl) 3-Methyl 5-Benzyl-4-hydroxy-1H-pyrrole-1,3-dicarboxylate (***11a′***) [22]

Following *GP2*, prepared from methyl (*S*)-4-((*tert*-butoxycarbonyl)amino)-3-oxo-5-phenylpentanoate (**10a**) (1 mmol, 321.4 mg), 45 min; CC (EtOAc/petroleum ether = 1:1). **11a**/**11a′** = 45:55 (in DMSO-*d*_6_). Yield: 268 mg (0.81 mmol, 81%) of colorless oil. ^1^H-NMR (500 MHz, DMSO-*d*_6_) for **11a**: *δ* 1.55 (*s*, 9H), 3.22 (*dd*, *J* = 2.7, 13.8 Hz, 1H), 3.38 (*dd*, *J* = 6.4, 13.8 Hz, 1H), 3.61 (*s*, 3H), 4.58 (*dd*, *J* = 2.6, 6.3 Hz, 1H), 6.91–6.97 (*m*, 2H), 8.71 (*s*, 1H). ^1^H-NMR (500 MHz, DMSO-*d*_6_) for **11a′**: *δ* 1.34 (*s*, 9H), 3.76 (*s*, 3H), 4.14 (*s*, 2H), 6.98–7.04 (*m*, 2H), 7.11–7.28 (*m*, 3H), 7.57 (*s*, 1H), 8.26 (*s*, 1H).

### 3.21. Synthesis of 1-(Tert-butyl) 3-Methyl 5-(4-(((Benzyloxy)carbonyl)amino)butyl)-4-oxo-4,5-dihydro-1H-pyrrole-1,3-dicarboxylate (***11b***) and 1-(Tert-butyl) 3-Methyl 5-(4-(((Benzyloxy)carbonyl)amino)butyl)-4-hydroxy-1H-pyrrole-1,3-dicarboxylate (***11b′***) [22]

Following *GP2*, prepared from methyl (*S*)-8-(((benzyloxy)carbonyl)amino)-4-((*tert*-butoxycarbonyl)amino)-3-oxooctanoate (**10b**) (1 mmol, 436.5 mg), 1 h; CC (EtOAc/petroleum ether = 1:1). **11b**/**11b′** = 37:63 (in DMSO-*d*_6_). Yield: 336 mg (0.82 mmol, 82%) of colorless oil. ^1^H-NMR (500 MHz, DMSO-*d*_6_) for **11b**: *δ* 0.98–1.08 (*m*, 1H), 1.08–1.19 (*m*, 1H), 1.50 (*s*, 9H), 1.83–1.93 (*m*, 1H), 1.97–2.08 (*m*, 1H), 2.93 (*q*, *J* = 6.7 Hz, 2H), 3.68 (*s*, 3H), 4.30 (*dd*, *J* = 3.1, 6.5 Hz, 1H), 8.98 (*s*, 1H). ^1^H-NMR (500 MHz, DMSO-*d*_6_) for **11b′**: *δ* 1.28–1.46 (*m*, 4H), 1.54 (*s*, 9H), 2.70 (*t*, *J* = 6.9 Hz, 2H), 2.98 (*q*, *J* = 6.3 Hz, 2H), 3.73 (*s*, 3H), 4.99 (*s*, 2H), 7.23 (*t*, *J* = 5.8 Hz, 1H), 7.26–7.41 (*m*, 5H), 7.49 (*s*, 1H), 7.95 (*s*, 1H).

### 3.22. Synthesis of 1-(Tert-butyl) 3-Methyl 5-(3-(Benzyloxy)-3-oxopropyl)-4-oxo-4,5-dihydro-1H-pyrrole-1,3-dicarboxylate (***11c***) and 1-(Tert-butyl) 3-Methyl 5-(3-(Benzyloxy)-3-oxopropyl)-4-hydroxy-1H-pyrrole-1,3-dicarboxylate (***11c′***) [23]

Following *GP2*, prepared from 7-benzyl 1-methyl (*S*)-4-((*tert*-butoxycarbonyl)amino)-3-oxoheptanedioate (**10c**) (1 mmol, 393.4 mg), 45 min; CC (EtOAc/petroleum ether = 1:1). **11c**/**11c′** = 42:58 (in DMSO-*d*_6_). Yield: 343 mg (0.85 mmol, 85%) of colorless oil. ^1^H-NMR (500 MHz, DMSO-*d*_6_) for **11c**: *δ* 1.50 (*s*, 9H), 3.68 (*s*, 3H), 4.35–4.40 (*m*, 1H), 5.06 (*d*, *J* = 5.4 Hz, 2H), 8.92 (*s*, 1H). ^1^H-NMR (500 MHz, DMSO-*d*_6_) for **11c′**: *δ* 1.53 (*s*, 9H), 2.51–2.57 (*m*, 2H), 2.99–3.05 (*m*, 2H), 3.73 (*s*, 3H), 5.08 (*s*, 2H), 7.30–7.40 (*m*, 5H), 7.49 (*s*, 1H), 8.11 (*s*, 1H).

### 3.23. Synthesis of Hexadecan-1-ol (***13***) [54]

Palmitic acid (**11d**) (20 mmol, 5.128 g) was dissolved in anhydrous THF (80 mL) under argon, and the solution was cooled in an ice bath (0 °C). While stirring in the ice bath, LiAlH_4_ (2.4 M in THF, 80 mmol, 33.3 mL) was added, and the reaction mixture was allowed to warm to room temperature over 1 h. The reaction mixture was stirred for a further 24 h under argon at room temperature and then quenched by careful addition of NaOH (1 M in H_2_O, 60 mL). The reaction mixture was extracted with diethyl ether (2 × 70 mL). The organic phase was dried over anhydrous Na_2_SO_4_, filtered, and the volatile components evaporated in vacuo. Yield: 3.957 g (16.3 mmol, 81%) of a white solid. ^1^H-NMR (500 MHz, CDCl_3_): *δ* 0.88 (*t*, *J* = 6.9 Hz, 3H), 1.19–1.39 (*m*, 27H), 1.52–1.61 (*m*, 2H), 3.64 (*t*, *J* = 6.6 Hz, 2H).

### 3.24. Synthesis of Palmitaldehyde (***14***) [55]

To a solution of hexadecan-1-ol (**13**) (16.3 mmol, 3.951 g) in anhydrous CH_2_Cl_2_ (100 mL), pyridinium chlorochromate (PCC, 24.5 mmol, ω = 0.98, 5.389 g) was added at room temperature and the reaction mixture was stirred for 16 h at room temperature. The solution was filtered through a plaque of Celite^®^, washed with CH_2_Cl_2_ and the volatiles evaporated in vacuo. The residue was purified by column chromatography (Silica gel 60, petroleum ether/ethyl acetate = 10:1). The fractions containing the pure product **14** were combined and the volatile components were evaporated in vacuo. Yield: 2.940 g (12.23 mmol, 75%) of a colorless oil. ^1^H-NMR (500 MHz, CDCl_3_): *δ* 0.88 (*t*, *J* = 6.9 Hz, 3H), 1.26 (s, 24H), 1.58–1.67 (*m*, 2H), 2.42 (*td*, *J* = 1.9, 7.4 Hz, 2H), 9.76 (*t*, *J* = 1.9 Hz, 1H).

### 3.25. Synthesis of 1-Nitroheptadecan-2-ol (***15***)

This was prepared according to the literature procedure [56]. Palmitaldehyde (**14**) (11.44 mmol, 2.751 g) was dissolved in a mixture of anhydrous THF and anhydrous *t*-butanol in a 1:1 ratio (50 mL) under argon. Nitromethane (17.16 mmol, 930 μL) was then added at room temperature. The mixture was cooled to 0 °C, *t*BuOK (1.144 mmol, 128 mg) was added, and the reaction mixture was allowed to warm to room temperature over 1 h. After 16 h at room temperature under argon, the reaction mixture was diluted with H_2_O (300 mL) and the product was extracted with diethyl ether (2 × 100 mL). The organic phase was washed with NaCl (aq. sat., 2 × 50 mL), dried over anhydrous Na_2_SO_4_, filtered, and the volatiles evaporated *in vacuo*. The crude product **15** was used for the following transformation without further purification. Yield: 2.794 g (9.267 mmol, 81%) of a yellowish oil. ^1^H-NMR (500 MHz, CDCl_3_): *δ* 0.88 (*t*, *J* = 6.9 Hz, 3H), 1.18–1.42 (*m*, 26H), 1.44–1.57 (*m*, 2H), 2.61 (br *s*, 1H), 4.28–4.34 (*m*, 1H), 4.38 (*dd*, *J* = 8.5, 13.0 Hz, 1H), 4.43 (*dd*, *J* = 2.7, 13.0 Hz, 1H).

### 3.26. Synthesis of (E)-1-Nitroheptadec-1-ene (***16***) [70]

This was prepared according to the literature procedure [56]. 1-Nitroheptadecan-2-ol (**15**) (9.12 mmol, 2.75 g) was dissolved in anhydrous CH_2_Cl_2_ (25 mL) and cooled to −10 °C. With stirring, trifluoroacetic anhydride (TFAA, 9.12 mmol, 1.269 mL) was added dropwise and the cooled mixture (−10 °C) was stirred for another 2 min. Over the next 10 min, triethylamine (2.532 mL, 18.24 mmol) was added dropwise and the reaction mixture was stirred at −10 °C for another 30 min. The reaction mixture was then diluted with CH_2_Cl_2_ (100 mL) and washed with NaHSO_4_ (aq., 1 M, 200 mL). The aqueous phase was extracted with CH_2_Cl_2_ (2 × 40 mL). The combined organic phase was dried over anhydrous Na_2_SO_4_, filtered, and the volatiles evaporated in vacuo. The residue was purified by column chromatography (Silica gel 60; petroleum ether/EtOAc = 40:1). The fractions containing the pure product **16** were combined and the volatile components were evaporated in vacuo. Product **16** was stored under argon at 5 °C. Yield: 2.016 g (7.114 mmol, 78%) of a white solid; m.p. = 25.0–25.7 °C. EI-HRMS: *m*/*z* = 306.2395 (MNa^+^); C_17_H_34_NNaO_2_ requires *m*/*z* = 306.2404 (MNa^+^); *ν*_max_ 2922, 2853, 1650, 1526, 1465, 1350, 960, 835, 723 cm^−1^. ^1^H-NMR (500 MHz, CDCl_3_): *δ* 0.88 (*t*, *J* = 6.9 Hz, 3H), 1.19–1.37 (*m*, 24H), 1.51 (*p*, *J* = 7.3 Hz, 2H), 2.27 (*qd*, *J* = 1.5, 7.4 Hz, 2H), 6.98 (*dt*, *J* = 1.6, 13.4 Hz, 1H), 7.23–7.33 (*m*, 1H). ^13^C-NMR (126 MHz, CDCl_3_): *δ* 14.27, 22.84, 27.86, 28.61, 29.24, 29.40, 29.51, 29.59, 29.72, 29.77, 29.80, 29.82, 29.84, 32.07, 139.69, 143.01 (1 signal missing due to overlapping).

### 3.27. Organocatalyzed Michael Addition of β-Keto Esters ***2*** to Nitroalkenes—General Procedure for the Preparation of Racemic Products **rac-*17***—General Procedure 3 (GP3)

To a solution/suspension of nitroalkene **12** or **16** (0.2 mmol, 1.0 equivalent or 0.3 mmol, 1.5 equivalents) and the achiral organocatalyst **X** (0.04 mmol, 0.2 equivalents, 16.4 mg) in anhydrous CH_2_Cl_2_ (1 mL) under argon at room temperature, β-keto ester **2** (0.3 mmol, 1.5 equivalents or 0.2 mmol, 1.0 equivalent) was added, and the resulting reaction mixture was stirred at room temperature for 24–72 h. The volatiles were evaporated in vacuo, and the residue was purified by column chromatography (Silica gel 60, mobile phase). The fractions containing the pure racemic product ***rac*-17** were combined, and the volatiles were evaporated in vacuo. The product ***rac*-17** was fully characterized and analyzed by HPLC.

### 3.28. Organocatalyzed Michael Addition of β-Keto Esters ***2*** to Nitroalkenes—General Procedure for the Organocatalyzed Asymmetric Addition—General Procedure 4 (GP4)

To a solution/suspension of nitroalkene **12** or **16** (0.2 mmol, 1.0 equivalent or 0.3 mmol, 1.5 equivalents) and the chiral organocatalyst **VIII** (0.02 mmol, 0.1 equivalents, 12.3 mg) or **I** (0.02 mmol, 0.1 equivalents, 10.9 mg) in anhydrous CH_2_Cl_2_ (1 mL) under argon at room temperature, β-keto ester **2** (0.3 mmol, 1.5 equivalents or 0.2 mmol, 1.0 equivalent) was added, and the resulting reaction mixture was stirred at room temperature for 24–72 h. The volatiles were evaporated in vacuo, and the residue was purified by column chromatography (Silica gel 60, mobile phase). The fractions containing the pure chiral nonracemic product **17** were combined the volatiles were evaporated *in vacuo*. The product **17** was fully characterized and analyzed by HPLC.

### 3.29. Synthesis of Methyl 4-((Tert-butoxycarbonyl)amino)-2-((R)-2-nitro-1-phenylethyl)-3-oxobutanoate (***17a***)

Following *GP3* and *GP4*, prepared from methyl 4-((*tert*-butoxycarbonyl)amino)-3-oxobutanoate (**2a**) (0.2 mmol, 46.3 mg) and *trans*-β-nitrostyrene (**12**) (0.3 mmol, 44.7 mg), organocatalyst **VIII**, 24 h; isolation by column chromatography (EtOAc/petroleum ether = 1:4). ***rac*-17a** Yield: 41.8 mg (0.110 mmol, 55%, two diastereomers in a ratio of 59:41 in DMSO-*d*_6_) of white solid. **17a** Yield: 63.9 mg (0.168 mmol, 84%, two diastereomers in a ratio of 56:44 in DMSO-*d*_6_) of white solid; m.p. = 116–122 °C. EI-HRMS: *m*/*z* = 381.1641 (MH^+^); C_18_H_25_N_2_O_7_ requires *m*/*z* = 381.1656 (MH^+^); *ν*_max_ 3378, 2981, 1751, 1714, 1555, 1497, 1455, 1429, 1367, 1252, 1154, 1020, 974, 895, 858, 764, 699, 637 cm^−1^. ^1^H-NMR (500 MHz, DMSO-*d*_6_) for major diastereomer: *δ* 1.39 (*s*, 9H), 3.37 (s, 3H), 3.95 (*dd*, *J* = 3.5, 5.8 Hz, 2H), 4.06–4.13 (*m*, 1H), 4.40 (*d*, *J* = 10.7 Hz, 1H), 4.87–4.96 (*m*, 2H), 7.18 (*t*, *J* = 5.9 Hz, 1H), 7.22–7.34 (*m*, 5H). ^1^H-NMR (500 MHz, DMSO-*d*_6_) for minor diastereomer: *δ* 1.33 (*s*, 9H), 3.70 (*s*, 3H), 3.50 (*dd*, *J* = 5.9, 18.8 Hz, 1H), 3.80 (*dd*, *J* = 5.8, 18.8 Hz, 1H), 4.46 (*d*, *J* = 10.2 Hz, 1H), 4.75 (*dd*, *J* = 4.3, 13.2 Hz, 1H), 4.85 (*d*, *J* = 10.8 Hz, 1H), 7.06 (*t*, *J* = 5.8 Hz, 1H). ^13^C-NMR (126 MHz, DMSO-*d*_6_) for both diastereomers: *δ* 28.10, 28.14, 42.35, 42.65, 50.18, 50.25, 52.45, 52.84, 57.28, 57.93, 59.78, 77.74, 78.22, 78.24, 78.44, 127.79, 127.85, 128.18, 128.25, 128.49, 128.64, 136.85, 136.92, 155.47, 155.81, 167.33, 170.36, 199.88, 200.62 (3 signals missing due to overlapping). HPLC: Chiralpak IA-3, *n*-Hexane/*i*-PrOH = 80:20, flow rate 1.0 mL/min, λ = 210 nm, T = 20 °C. Diastereomer 1: *t*R = 9.24 min (minor); 17.62 min (major)—91% *ee*. Diastereomer 2: *t*R = 14.12 min (major); 23.56 min (minor)—93% *ee*.

### 3.30. Synthesis of Methyl 5-((Tert-butoxycarbonyl)amino)-2-((R)-2-nitro-1-phenylethyl)-3-oxopentanoate (***17b***) [22]

Following *GP3* and *GP4*, prepared from methyl 5-((*tert*-butoxycarbonyl)amino)-3-oxopentanoate (**2b**) (0.2 mmol, 49.1 mg) and *trans*-β-nitrostyrene (**12**) (0.3 mmol, 44.7 mg), organocatalyst **VIII**, 24 h; isolation by column chromatography (EtOAc/petroleum ether = 1:4). ***rac*-17b** Yield: 69.4 mg (0.176 mmol, 88%, two diastereomers in a ratio of 53:47 in DMSO-*d*_6_) of white solid. **17b** Yield: 67.1 mg (0.170 mmol, 85%, two diastereomers in a ratio of 53:47 in DMSO-*d*_6_) of white solid; m.p. = 96.1–98.4 °C. EI-HRMS: *m*/*z* = 417.1618 (MNa^+^); C_19_H_27_N_2_NaO_7_ requires *m*/*z* = 417.1632 (MNa^+^); *ν*_max_ 3424, 2978, 1743, 1707, 1553, 1506, 1455, 1434, 1366, 1246, 1164, 1082, 966, 859, 756, 701 cm^−1^. ^1^H-NMR (500 MHz, DMSO-*d*_6_) for both diastereomers: *δ* 1.34 (*s*, 4.5H), 1.37 (*s*, 4.5H), 2.24–2.34 (*m*, 0.5H), 2.58–2.68 (*m*, 0.5H), 2.74 (*t*, *J* = 6.8 Hz, 1H), 2.76–2.91 (*m*, 1H), 3.15 (*q*, *J* = 6.4 Hz, 1H), 3.35 (*s*, 1.5H), 3.70 (*s*, 1.5H), 4.00–4.09 (*m*, 1H), 4.37 (*dd*, *J* = 6.0, 10.5 Hz, 1H), 4.81 (*d*, *J* = 7.5 Hz, 1H), 4.87–4.99 (*m*, 1H), 6.58 (*t*, *J* = 5.7 Hz, 0.5H), 6.85 (*t*, *J* = 5.7 Hz, 0.5H), 7.21–7.35 (*m*, 5H). ^13^C-NMR (126 MHz, DMSO-*d*_6_) for both diastereomers: *δ* 28.19, 28.22, 34.52, 34.79, 42.30, 42.36, 42.66, 42.89, 52.43, 52.83, 60.00, 60.84, 77.69, 77.78, 78.00, 78.15, 127.79, 127.86, 128.27, 128.46, 128.63, 136.80, 136.96, 155.33, 155.53, 166.88, 167.72, 201.83 (6 signals missing due to overlapping). HPLC: Chiralpak IA-3, *n*-Hexane/*i*-PrOH = 80:20, flow rate 1.0 mL/min, λ = 210 nm, T = 20 °C. Diastereomer 1: *t*R = 7.22 min (minor); 8.98 min (major)—95% *ee*. Diastereomer 2: *t*R = 12.53 min (minor); 20.94 min (major)—95% *ee*.

### 3.31. Synthesis of Methyl 2-(3-((Tert-butoxycarbonyl)amino)propanoyl)-3-(nitromethyl)octadecanoate (**rac-*17c***)

Following *GP3*, prepared from methyl 5-((*tert*-butoxycarbonyl)amino)-3-oxopentanoate (**2b**) (0.3 mmol, 73.6 mg) and (*E*)-1-nitroheptadec-1-ene (**16**) (0.2 mmol, 56.7 mg), organocatalyst **X**, 24 h; isolation by column chromatography (EtOAc/petroleum ether = 1:5). ***rac*-17c** Yield: 55.0 mg (0.104 mmol, 52%, two diastereomers in a ratio of 53:47 in CDCl_3_) of colorless oil. EI-HRMS: *m*/*z* = 429.3313 (MH^+^-Boc); C_23_H_45_N_2_O_5_ requires *m*/*z* = 429.3323 (MH^+^-Boc); *ν*_max_ 3413, 2923, 2853, 1743, 1712, 1552, 1505, 1436, 1366, 1248, 1168, 1084, 966, 911, 863, 781, 733 cm^−1^. ^1^H-NMR (600 MHz, CDCl_3_) for the major diastereomer: *δ* 0.88 (*t*, *J* = 6.9 Hz, 3H), 1.19–1.40 (*m*, 28H), 1.43 (*s*, 9H), 2.71–2.79 (*m*, 1H), 2.82–2.96 (*m*, 2H), 3.30–3.45 (*m*, 2H), 3.76 (*s*, 3H), 3.73–3.83 (*m*, 1H), 4.60 (*dd*, *J* = 5.0, 12.9 Hz, 1H), 4.65 (*dd*, *J* = 4.5, 13.2 Hz, 1H), 4.86–4.96 (*m*, 1H). ^1^H-NMR (600 MHz, CDCl_3_) for the minor diastereomer: *δ* 3.76 (*s*, 3H), 4.52 (*dd*, *J* = 5.9, 13.1 Hz, 2H). ^13^C-NMR (151 MHz, CDCl_3_) for both diastereomers: *δ* 14.27, 22.83, 26.82, 26.88, 28.50, 29.42, 29.43, 29.49, 29.50, 29.63, 29.65, 29.74, 29.79, 29.81, 29.83, 29.84, 30.28, 32.06, 35.17, 36.64, 36.66, 43.35, 43.56, 52.94, 53.08, 59.31, 59.76, 76.02, 76.47, 79.58, 79.63, 155.95, 168.46, 203.83 (18 signals missing due to overlapping).

### 3.32. Synthesis of Methyl 4-((3-Methylbut-2-en-1-yl)oxy)-2-((R)-2-nitro-1-phenylethyl)-3-oxobutanoate (***17d***)

Following *GP3* and *GP4*, prepared from methyl 4-((3-methylbut-2-en-1-yl)oxy)-3-oxobutanoate (**2c**) (0.2 mmol, 40.0 mg) and *trans*-β-nitrostyrene (**12**) (0.3 mmol, 44.7 mg), organocatalyst **VIII**, 24 h; isolation by column chromatography (EtOAc/petroleum ether = 1:5). ***rac*-17d** Yield: 54.5 mg (0.156 mmol, 78%, two diastereomers in a ratio of 60:40 in CDCl_3_) of colorless oil. **17d** Yield: 62.2 mg (0.178 mmol, 89%, two diastereomers in a ratio of 60:40 in CDCl_3_) of colorless oil. EI-HRMS: *m*/*z* = 367.1858 (M+NH_4_^+^); C_18_H_27_N_2_O_6_ requires *m*/*z* = 367.1864 (M+NH_4_^+^); *ν*_max_ 3033, 2954, 2916, 1746, 1724, 1552, 1496, 1434, 1378, 1247, 1199, 1169, 1092, 1034, 981, 942, 893, 766, 700, 618 cm^−1^. ^1^H-NMR (500 MHz, CDCl_3_) for the major diastereomer: *δ* 1.61 (*s*, 3H), 1.74 (*s*, 3H), 3.73 (*s*, 3H), 3.76–3.90 (*m*, 2H), 4.21–4.32 (*m*, 3H), 4.79–4.95 (*m*, 3H), 5.20–5.24 (*m*, 1H), 7.18–7.24 (*m*, 2H), 7.25–7.33 (*m*, 3H). ^1^H-NMR (500 MHz, CDCl_3_) for the minor diastereomer: *δ* 1.66 (*s*, 3H), 1.75 (*s*, 3H), 3.52 (s, 3H), 3.98 (*d*, *J* = 7.0 Hz, 2H), 4.02 (*d*, *J* = 17.5 Hz, 1H), 4.10 (*d*, *J* = 17.4 Hz, 1H). ^13^C-NMR (126 MHz, CDCl_3_): *δ* 18.12, 18.16, 25.90, 25.93, 42.13, 42.35, 52.80, 52.96, 56.60, 57.33, 67.75, 67.94, 74.72, 77.22, 77.77, 119.78, 119.80, 128.05, 128.22, 128.43, 128.50, 129.10, 129.22, 136.39, 136.41, 138.70, 138.84, 167.43, 167.90, 202.15, 202.77 (1 signal missing due to overlapping). HPLC: Chiralpak AS-H, *n*-Hexane/EtOH = 90:10, flow rate 1.0 mL/min, λ = 210 nm, T = 20 °C. Minor diastereomer: enantiomers: *t*R = 14.066 min (minor); 17.773 min (major)—94% *ee*. Major diastereomer: enantiomers: *t*R = 17.164 min (major); 19.406 min (minor)—91% *ee*.

### 3.33. Synthesis of Methyl 2-(2-((3-Methylbut-2-en-1-yl)oxy)acetyl)-3-(nitromethyl)octadecanoate (**rac-*17e***)

Following *GP3*, prepared from methyl 4-((3-methylbut-2-en-1-yl)oxy)-3-oxobutanoate (**2c**) (0.3 mmol, 60.1 mg) and (*E*)-1-nitroheptadec-1-ene (**16**) (0.2 mmol, 56.7 mg), organocatalyst **X**, 24 h; isolation by column chromatography (EtOAc/petroleum ether = 1:5). ***rac*-17e** Yield: 52.2 mg (0.108 mmol, 54%, two diastereomers in a ratio of 51:49 in CDCl_3_) of colorless oil. EI-HRMS: *m*/*z* = 501.3887 (M+NH_4_^+^); C_27_H_53_N_2_O_6_ requires *m*/*z* = 501.3898 (M+NH_4_^+^); *ν*_max_ 2923, 2853, 1726, 1553, 1435, 1379, 1250, 1199, 1158, 1092, 1000, 780, 722 cm^−1^. ^1^H-NMR (600 MHz, CDCl_3_) for both diastereomers: *δ* 0.88 (*t*, *J* = 7.0 Hz, 3H), 1.18–1.51 (*m*, 28H), 1.68 (*dd*, *J* = 1.4, 4.8 Hz, 3H), 1.77 (*dd*, *J* = 1.2, 4.1 Hz, 3H), 2.85–2.93 (*m*, 1H), 3.73 (*s*, 1.5H), 3.74 (*s*, 1.5H), 3.94–4.15 (*m*, 5H), 4.46 (*dd*, *J* = 7.1, 13.3 Hz, 0.5H), 4.53 (*dd*, *J* = 5.7, 13.2 Hz, 0.5H), 4.59–4.69 (*m*, 1H), 5.27–5.34 (*m*, 1H). ^13^C-NMR (151 MHz, CDCl_3_) for both diastereomers: *δ* 14.28, 18.19, 18.21, 22.84, 25.96, 26.88, 27.05, 29.38, 29.45, 29.51, 29.66, 29.75, 29.80, 29.83, 29.85, 30.29, 32.07, 36.10, 36.30, 52.67, 52.82, 54.92, 55.32, 67.99, 68.00, 74.63, 74.73, 76.40, 76.75, 119.82, 119.92, 138.71, 138.89, 168.55, 168.56, 203.55, 203.88 (17 signals missing due to overlapping).

### 3.34. Synthesis of Methyl 2-((R)-2-Nitro-1-phenylethyl)-3-oxooctadecanoate (***17f***)

Following *GP3* and *GP4*, prepared from methyl 3-oxooctadecanoate (**2d**) (0.3 mmol, 93.7 mg) and *trans*-β-nitrostyrene (**12**) (0.2 mmol, 29.8 mg), organocatalyst **VIII**, 24 h; isolation by column chromatography (EtOAc/petroleum ether = 1:10). ***rac*-17f** Yield: 34.2 mg (0.074 mmol, 37%, two diastereomers in a ratio of 56:44 in CDCl_3_) of white solid. **17f** Yield: 55.4 mg (0.120 mmol, 60%, two diastereomers in a ratio of 50:50 in CDCl_3_) of white solid; m.p. = 60.0–61.2 °C. EI-HRMS: *m*/*z* = 462.3214 (MH^+^); C_27_H_44_NO_5_ requires *m*/*z* = 462.3214 (MH^+^); *ν*_max_ 2915, 2850, 1742, 1711, 1550, 1496, 1471, 1455, 1438, 1383, 1334, 1281, 1245, 1208, 1173, 1128, 1092, 1072, 1033, 1004, 983, 918, 892, 853, 765, 718, 700, 616 cm^−1^. ^1^H-NMR (500 MHz, CDCl_3_) for the major diastereomer: *δ* 0.88 (*t*, *J* = 6.9 Hz, 3H), 0.94–1.04 (*m*, 2H), 1.06–1.39 (*m*, 22H), 1.51–1.60 (*m*, 2H), 2.13 (*dt*, *J* = 7.2, 17.8, 1H), 2.38–2.50 (*m*, 1H), 3.76 (*s*, 3H), 4.03 (*d*, *J* = 10.0 Hz, 1H), 4.19–4.28 (*m*, 1H), 4.75–4.89 (*m*, 2H), 7.16–7.22 (*m*, 2H), 7.24–7.34 (*m*, 3H). ^1^H-NMR (500 MHz, CDCl_3_) for the minor diastereomer: *δ* 2.61 (*dt*, *J* = 7.4, 17.7 Hz, 1H), 3.52 (*s*, 3H), 4.13 (*d*, *J* = 9.5 Hz, 1H). ^13^C-NMR (126 MHz, CDCl_3_) for both diastereomers: *δ* 14.27, 22.83, 23.05, 23.39, 28.74, 29.00, 29.33, 29.45, 29.48, 29.50, 29.56, 29.68, 29.73, 29.76, 29.78, 29.80, 29.83, 32.06, 42.53, 42.86, 43.50, 43.84, 52.87, 53.05, 60.97, 61.34, 77.67, 77.93, 127.94, 128.11, 128.42, 128.50, 129.15, 129.26, 136.44, 136.63, 167.62, 168.14, 202.76, 203.81 (10 signals missing due to overlapping). HPLC: Chiralpak IA-3, *n*-Hexane/EtOH = 95:5, flow rate 1.0 mL/min, λ = 210 nm, T = 25 °C. Minor diastereomer: enantiomers: *t*R = 7.287 min (minor); 22.511 min (major)—95% *ee*. Major diastereomer: enantiomers: *t*R = 8.963 min (major); 13.215 min (minor)—96% *ee*.

### 3.35. Synthesis of Methyl 2-(1-Nitroheptadecan-2-yl)-3-oxooctadecanoate (**rac-*17g***)

Following *GP3*, prepared from methyl 3-oxooctadecanoate (**2d**) (0.3 mmol, 93.7 mg) and (*E*)-1-nitroheptadec-1-ene (**16**) (0.2 mmol, 56.7 mg), organocatalyst **X**, 24 h; isolation by column chromatography (EtOAc/petroleum ether = 1:10). ***rac*-17g** Yield: 78.7 mg (0.132 mmol, 66%, two diastereomers in a ratio of 68:32 in CDCl_3_) of white solid; m.p. = 40.0–40.9 °C. EI-HRMS: *m*/*z* = 594.5108 (M−H^+^)^−^; C_36_H_68_NO_5_ requires *m*/*z* = 594.5103 (M−H^+^)^−^; *ν*_max_ 2955, 2914, 2849, 1730, 1707, 1556, 1543, 1470, 1435, 1402, 1380, 1243, 1204, 1128, 1073, 1000, 863, 719 cm^−1^. ^1^H-NMR (600 MHz, CDCl_3_) for both diastereomers: *δ* 0.88 (*t*, *J* = 7.0, 6H), 1.11–1.46 (*m*, 52H), 1.55–1.62 (*m*, 2H), 2.47–2.55 (*m*, 1H), 2.57–2.66 (*m*, 1H), 2.79–2.90 (*m*, 1H), 3.75 (*s*, 2.04H), 3.75 (*s*, 0.96H), 3.76–3.81 (*m*, 1H), 4.49–4.57 (*m*, 1H), 4.59–4.67 (m, 1H). ^13^C-NMR (151 MHz, CDCl_3_) for both diastereomers: *δ* 14.28, 22.85, 23.46, 23.50, 26.80, 26.91, 29.08, 29.10, 29.42, 29.45, 29.48, 29.50, 29.52, 29.60, 29.65, 29.67, 29.75, 29.81, 29.83, 29.85, 30.22, 32.08, 36.72, 36.77, 43.43, 43.60, 52.79, 52.91, 59.33, 59.72, 76.16, 76.57, 168.77, 168.81, 204.33, 204.46 (36 signals missing due to overlapping).

### 3.36. Synthesis of Methyl 2-((R)-2-Nitro-1-phenylethyl)-3-oxoicosanoate (***17h***)

Following *GP3* and *GP4*, prepared from methyl 3-oxoicosanoate (**2e**) (0.3 mmol, 102.2 mg) and *trans*-β-nitrostyrene (**12**) (0.2 mmol, 29.8 mg), organocatalyst **VIII**, 24 h; isolation by column chromatography (EtOAc/petroleum ether = 1:10). ***rac*-17h** Yield: 43.1 mg (0.088 mmol, 44%, two diastereomers in a ratio of 54:46 in CDCl_3_) of white solid. **17h** Yield: 52.9 mg (0.108 mmol, 54%, two diastereomers in a ratio of 48:52 in CDCl_3_) of white solid; m.p. = 42.2–44.7 °C. EI-HRMS: *m*/*z* = 490.3524 (MH^+^); C_29_H_48_NO_5_ requires *m*/*z* = 490.3527 (MH^+^); *ν*_max_ 2914, 2849, 1737, 1712, 1555, 1496, 1471, 1455, 1433, 1404, 1378, 1271, 1198, 1168, 1113, 1082, 982, 891, 765, 716, 699 cm^−1^. ^1^H-NMR (500 MHz, CDCl_3_) for the major diastereomer: *δ* 0.88 (*t*, *J* = 6.9 Hz, 3H), 1.06–1.37 (*m*, 30H), 2.38–2.50 (*m*, 1H), 2.61 (*dt*, *J* = 7.4, 17.7 Hz, 1H), 3.52 (*s*, 3H), 4.13 (*d*, *J* = 9.4 Hz, 1H), 4.19–4.28 (*m*, 1H), 4.74–4.89 (*m*, 2H), 7.16–7.22 (*m*, 2H), 7.24–7.33 (*m*, 3H). ^1^H-NMR (500 MHz, CDCl_3_) for the minor diastereomer: *δ* 0.94–1.04 (*m*, 2H), 1.51–1.60 (*m*, 2H), 2.13 (*dt*, *J* = 7.1, 17.7 Hz, 1H), 3.75 (*s*, 3H), 4.03 (*d*, *J* = 10.0 Hz, 1H). ^13^C-NMR (126 MHz, CDCl_3_) for both diastereomers: *δ* 14.25, 22.82, 23.03, 23.37, 28.72, 28.99, 29.32, 29.43, 29.47, 29.49, 29.55, 29.67, 29.72, 29.75, 29.77, 29.79, 29.82, 32.05, 42.51, 42.86, 43.48, 43.83, 52.84, 53.02, 60.94, 61.33, 77.65, 77.92, 127.93, 128.10, 128.39, 128.47, 129.13, 129.24, 136.44, 136.63, 167.61, 168.12, 202.74, 203.79 (14 signals missing due to overlapping). HPLC: Chiralpak IA-3, *n*-Hexane/EtOH = 95:5, flow rate 1.0 mL/min, λ = 210 nm, T = 25 °C. Major diastereomer: enantiomers: *t*R = 8.838 min (minor); 26.885 min (major)—96% *ee*. Minor diastereomer: enantiomers: *t*R = 10.913 min (major); 15.371 min (minor)—96% *ee*.

### 3.37. Synthesis of Methyl 2-(1-Nitroheptadecan-2-yl)-3-oxoicosanoate (**rac-*17i***)

Following *GP3*, prepared from methyl 3-oxoicosanoate (**2e**) (0.3 mmol, 102.2 mg) and (*E*)-1-nitroheptadec-1-ene (**16**) (0.2 mmol, 56.7 mg), organocatalyst **X**, 24 h; isolation by column chromatography (EtOAc/petroleum ether = 1:10). ***rac*-17i** Yield: 52.4 mg (0.084 mmol, 42%, two diastereomers in a ratio of 50:50 in CDCl_3_) of white solid; m.p. = 42.0–43.9 °C. EI-HRMS: *m*/*z* = 622.5416 (M−H^+^)^−^; C_38_H_73_NO_5_ requires *m*/*z* = 622.5421 (M−H^+^)^−^; *ν*_max_ 2956, 2915, 2848, 1731, 1706, 1556, 1542, 1470, 1435, 1402, 1379, 1349, 1241, 1205, 1128, 1108, 1078, 1001, 719 cm^−1^. ^1^H-NMR (500 MHz, CDCl_3_) for both diastereomers: *δ* 0.87 (*t*, *J* = 6.9 Hz, 6H), 1.08–1.44 (*m*, 56H), 1.53–1.61 (*m*, 2H), 2.45–2.55 (*m*, 1H), 2.56–2.66 (*m*, 1H), 2.78–2.89 (*m*, 1H), 3.74 (*s*, 1.5H), 3.74 (*s*, 1.5H), 3.75 (*d*, *J* = 6.9 Hz, 0.5H), 3.79 (*d*, *J* = 7.6 Hz, 0.5H), 4.45–4.55 (*m*, 1H), 4.62 (*td*, *J* = 4.6, 13.5 Hz, 1H). ^13^C-NMR (126 MHz, CDCl_3_) for both diastereomers: *δ* 14.25, 22.83, 23.43, 23.48, 26.77, 26.88, 29.06, 29.08, 29.40, 29.43, 29.46, 29.48, 29.50, 29.59, 29.64, 29.71, 29.74, 29.79, 29.82, 29.84, 30.20, 32.06, 36.68, 36.74, 43.38, 43.56, 52.74, 52.86, 59.30, 59.69, 76.13, 76.54, 168.74, 168.78, 204.28, 204.41 (40 signals missing due to overlapping).

### 3.38. Synthesis of Tert-butyl 2-((R)-2-nitro-1-phenylethyl)-3-oxoicosanoate (***17j***)

Following *GP3* and *GP4*, prepared from *tert*-butyl 3-oxoicosanoate (**2f**) (0.3 mmol, 114.8 mg) and *trans*-β-nitrostyrene (**12**) (0.2 mmol, 29.8 mg), organocatalyst **VIII**, 24 h; isolation by column chromatography (EtOAc/petroleum ether = 1:10). ***rac*-17j** Yield: 42.5 mg (0.080 mmol, 40%, two diastereomers in a ratio of 61:39 in CDCl_3_) of white solid. **17j** Yield: 50.0 mg (0.094 mmol, 47%, two diastereomers in a ratio of 36:64 in CDCl_3_) of white solid; m.p. = 54.0–56.4 °C. EI-HRMS: *m*/*z* = 549.4273 (M+NH_4_^+^); C_32_H_57_N_2_O_5_ requires *m*/*z* = 549.4262 (M+NH_4_^+^); *ν*_max_ 2916, 2849, 1731, 1712, 1553, 1496, 1468, 1434, 1394, 1378, 1284, 1250, 1148, 1126, 1092, 1062, 982, 914, 838, 770, 751, 720, 700 cm^−1^. ^1^H-NMR (500 MHz, CDCl_3_) for the major diastereomer: *δ* 0.88 (*t*, *J* = 6.9 Hz, 3H), 0.98–1.07 (*m*, 2H), 1.09–1.39 (*m*, 28H), 1.46 (*s*, 9H), 1.55–1.63 (*m*, 1H), 2.14 (*dt*, *J* = 7.1, 17.5 Hz, 1H), 2.38–2.52 (*m*, 1H), 3.91 (*d*, *J* = 9.9 Hz, 1H), 4.12–4.23 (*m*, 1H), 4.65–4.76 (*m*, 1H), 7.16–7.34 (*m*, 5H). ^1^H-NMR (500 MHz, CDCl_3_) for the minor diastereomer: *δ* 2.62 (*dt*, *J* = 7.4, 17.4 Hz, 1H), 4.01 (*d*, *J* = 10.1 Hz, 1H), 4.77–4.90 (*m*, 2H). ^13^C-NMR (126 MHz, CDCl_3_) for both diastereomers: *δ* 14.26, 22.83, 23.18, 23.53, 27.52, 27.98, 28.90, 29.15, 29.38, 29.48, 29.50, 29.56, 29.69, 29.73, 29.76, 29.80, 29.83, 32.06, 42.54, 42.79, 42.87, 43.59, 62.16, 62.51, 78.06, 78.40, 82.95, 83.40, 128.18, 128.29, 128.32, 128.36, 128.95, 129.14, 136.81, 136.84, 166.04, 166.75, 203.13, 203.98 (16 signals missing due to overlapping). HPLC: Chiralpak IA-3, *n*-Hexane/EtOH = 95:5, flow rate 1.0 mL/min, λ = 210 nm, T = 25 °C. Minor diastereomer: enantiomers: *t*R = 6.051 min (minor); 8.241 min (major)—93% *ee*. Major diastereomer: enantiomers: *t*R = 6.768 min (minor); 7.403 min (major)—98% *ee*.

### 3.39. Synthesis of Tert-butyl 2-(1-nitroheptadecan-2-yl)-3-oxoicosanoate (**rac-*17k***)

Following *GP3*, prepared from *tert*-butyl 3-oxoicosanoate (**2f**) (0.3 mmol, 114.8 mg) and (*E*)-1-nitroheptadec-1-ene (**16**) (0.2 mmol, 56.7 mg), organocatalyst **X**, 24 h; isolation by column chromatography (first CC: CH_2_Cl_2_/petroleum ether = 1:3; second CC: EtOAc/petroleum ether = 1:10). ***rac*-17k** Yield: 51.9 mg (0.079 mmol, 39%, two diastereomers in a ratio of 66:34 in CDCl_3_) of white solid; m.p. = 41.2–45.0 °C. EI-HRMS: *m*/*z* = 664.5894 (M−H^+^)^−^; C_41_H_78_NO_5_ requires *m*/*z* = 664.5886 (M−H^+^)^−^; *ν*_max_ 2916, 2848, 1726, 1703, 1556, 1545, 1466, 1370, 1256, 1210, 1157, 1047, 845, 720, 618 cm^−1^. ^1^H-NMR (500 MHz, CDCl_3_) for both diastereomers: *δ* 0.88 (*t*, *J* = 7.0 Hz, 6H), 1.12–1.44 (*m*, 56H), 1.47 (*s*, 9H), 1.55–1.63 (*m*, 2H), 2.45–2.54 (*m*, 1H), 2.56–2.67 (*m*, 1H), 2.76–2.86 (*m*, 1H), 3.63 (*d*, *J* = 6.9, 0.34H), 3.66 (*d*, *J* = 7.5 Hz, 0.66H), 4.48–4.56 (*m*, 1H), 4.57–4.67 (*m*, 1H). ^13^C-NMR (126 MHz, CDCl_3_) for both diastereomers: *δ* 14.26, 22.84, 23.55, 23.60, 26.79, 26.88, 28.03, 28.07, 29.23, 29.49, 29.51, 29.54, 29.60, 29.67, 29.76, 29.81, 29.83, 29.85, 30.19, 32.08, 36.66, 36.77, 43.26, 43.47, 60.43, 60.98, 76.40, 76.77, 83.01, 83.07, 167.40, 167.43, 204.68, 204.83 (44 signals missing due to overlapping).

### 3.40. Synthesis of Methyl (11Z,14Z)-2-((R)-2-Nitro-1-phenylethyl)-3-oxoicosa-11,14-dienoate (***17l***)

Following *GP3* and *GP4*, prepared from methyl (11*Z*,14*Z*)-3-oxoicosa-11,14-dienoate (**2g**) (0.3 mmol, 101.0 mg) and *trans*-β-nitrostyrene (**12**) (0.2 mmol, 29.8 mg), organocatalyst **VIII**, 24 h; isolation by column chromatography (EtOAc/petroleum ether = 1:20). ***rac*-17l** Yield: 58 mg (0.120 mmol, 60%, two diastereomers in a ratio of 52:48 in CDCl_3_) of colorless oil. **17l** Yield: 60.2 mg (0.124 mmol, 62%, two diastereomers in a ratio of 51:49 in CDCl_3_) of colorless oil. EI-HRMS: *m*/*z* = 508.3032 (MNa^+^); C_29_H_43_NO_5_Na requires *m*/*z* = 508.3032 (MNa^+^); *ν*_max_ 3009, 2925, 2855, 1744, 1717, 1554, 1496, 1455, 1434, 1377, 1243, 1168, 981, 914, 765, 699 cm^−1^. ^1^H-NMR (600 MHz, CDCl_3_) for both diastereomers: *δ* 0.89 (*td*, *J* = 1.5, 7.0 Hz, 3H), 0.95–1.05 (*m*, 1H), 1.10–1.16 (*m*, 1H), 1.16–1.21 (*m*, 1H), 1.23–1.40 (*m*, 12H), 1.52–1.60 (*m*, 1H), 1.97–2.07 (*m*, 4H), 2.13 (*dt*, *J* = 7.2, 17.8 Hz, 0.5H), 2.39–2.49 (*m*, 1H), 2.61 (*dt*, *J* = 7.4, 17.7 Hz, 0.5H), 2.77 (*q*, *J* = 6.5 Hz, 2H), 3.52 (*s*, 1.5H), 3.76 (*s*, 1.5H), 4.03 (*d*, *J* = 10.0 Hz, 0.5H), 4.13 (*d*, *J* = 9.4 Hz, 0.5H), 4.19–4.27 (*m*, 1H), 4.74–4.88 (*m*, 2H), 5.28–5.43 (*m*, 4H), 7.17–7.21 (*m*, 2H), 7.22–7.34 (*m*, 3H). ^13^C-NMR (151 MHz, CDCl_3_) for both diastereomers: *δ* 14.22, 22.71, 23.03, 23.37, 25.76, 27.30, 27.31, 27.34, 28.70, 28.97, 29.13, 29.20, 29.24, 29.36, 29.48, 29.67, 29.72, 31.66, 42.53, 42.87, 43.48, 43.83, 52.87, 53.05, 60.98, 61.35, 77.67, 77.93, 127.94, 128.00, 128.02, 128.12, 128.20, 128.23, 128.43, 128.51, 129.16, 129.26, 130.13, 130.38, 136.45, 136.64, 168.14, 202.70, 203.76 (9 signals missing due to overlapping). HPLC: Chiralpak IA-3, *n*-Hexane/EtOH = 95:5, flow rate 1.0 mL/min, λ = 210 nm, T = 25 °C. Minor diastereomer: enantiomers: *t*R = 7.439 min (minor); 19.360 min (major)—96% *ee*. Major diastereomer: enantiomers: *t*R = 9.402 min (major); 12.992 min (minor)—96% *ee*.

### 3.41. Synthesis of Methyl (4S)-7-((Tert-butoxycarbonyl)amino)-2-(2-nitro-1-phenylethyl)-3-oxo-4-stearamidoheptanoate (***17m***)

Following *GP3*, prepared from methyl (*S*)-7-((*tert*-butoxycarbonyl)amino)-3-oxo-4-stearamidoheptanoate (**2h**) (0.2 mmol, 111.0 mg) and *trans*-β-nitrostyrene (**12**) (0.3 mmol, 44.7 mg), organocatalyst **X**, 48 h; isolation by column chromatography (1. EtOAc/petroleum ether = 1:2; 1. EtOAc/petroleum ether = 1:1). **17m** Yield: 100.0 mg (0.142 mmol, 71%, 4 diastereomers in a ratio of 27:21:24:28 in CDCl_3_) of white semisolid. EI-HRMS: *m*/*z* = 704.4827 (MH^+^); C_39_H_66_N_3_O_8_ requires *m*/*z* = 704.4844 (MH^+^); *ν*_max_ 3375, 2920, 2851, 1739, 1720, 1687, 1648, 1551, 1518, 1455, 1436, 1365, 1247, 1214, 1168, 1089, 1005, 872, 764, 720, 701, 617 cm^−1^. ^1^H-NMR (500 MHz, CDCl_3_) for 4 diastereomers: *δ* 0.88 (*t*, *J* = 6.9, 3H), 1.05–1.39 (*m*, 29H), 1.42–1.45 (*m*, 9H), 1.46–1.68 (*m*, 4H), 1.71–1.86 (*m*, 1H), 2.02–2.28 (*m*, 2H), 2.77–3.22 (*m*, 2H), 3.48, 3.49, 3.74, 3.77 (4 × *s*, 3H), 4.22–4.96 (*m*, 6H), 5.83–6.68 (*m*, 1H), 7.18–7.35 (*m*, 5H). ^13^C-NMR (126 MHz, CDCl_3_) for 4 diastereomers: *δ* 14.24, 22.80, 25.55, 25.62, 25.67, 25.90, 26.01, 26.20, 26.30, 26.90, 26.95, 27.05, 27.20, 28.48, 28.49, 29.38, 29.40, 29.43, 29.45, 29.47, 29.60, 29.61, 29.63, 29.73, 29.75, 29.77, 29.81, 32.03, 36.04, 36.40, 36.44, 36.50, 39.56, 39.71, 39.75, 42.54, 42.63, 43.10, 43.25, 52.84, 53.02, 53.11, 53.36, 57.89, 58.13, 58.32, 58.44, 58.62, 58.97, 59.18, 77.27, 77.36, 77.58, 77.70, 79.48, 79.52, 79.67, 79.70, 128.09, 128.14, 128.27, 128.36, 128.42, 128.50, 129.04, 129.18, 129.21, 136.21, 136.27, 136.50, 136.55, 156.26, 156.41, 156.55, 156.59, 166.95, 167.15, 167.30, 167.66, 173.10, 173.47, 173.85, 174.11, 200.93, 201.85, 202.18, 202.94 (69 signals missing due to overlapping).

### 3.42. Synthesis of Methyl 2-((S)-5-((Tert-butoxycarbonyl)amino)-2-stearamidopentanoyl)-3-(nitromethyl)octadecanoate (***17n***)

Following *GP3* and *GP4*, prepared from methyl (*S*)-7-((*tert*-butoxycarbonyl)amino)-3-oxo-4-stearamidoheptanoate (**2h**) (0.2 mmol, 111.0 mg) and (*E*)-1-nitroheptadec-1-ene (**16**) (0.3 mmol, 85.0 mg), organocatalyst **X**, 48 h; isolation by column chromatography (1. EtOAc/petroleum ether = 1:3; 1. EtOAc/petroleum ether = 1:2); **17n** Yield: 129.1 mg (0.154 mmol, 77%, 4 diastereomers in a ratio of 26:28:25:21 in CDCl_3_) of white solid. Organocatalyst **VIII**, 48 h; isolation by column chromatography (1. EtOAc/petroleum ether = 1:3; 1. EtOAc/petroleum ether = 1:2); **17n** Yield: 147.5 mg (0.176 mmol, 88%, 4 diastereomers in a ratio of 45:11:8:36 in CDCl_3_) of white solid. EI-HRMS: *m*/*z* = 838.6860 (MH^+^); C_48_H_92_N_3_O_8_ requires *m*/*z* = 838.6879 (MH^+^); *ν*_max_ 3361, 2917, 2850, 1741, 1718, 1687, 1644, 1553, 1524, 1467, 1366, 1250, 1222, 1171, 1039, 1011, 869, 721, 646 cm^−1^. ^1^H-NMR (500 MHz, CDCl_3_) for 4 diastereomers: *δ* 0.88 (*t*, *J* = 6.9 Hz, 6H), 1.10–1.39 (*m*, 57H), 1.44 (*s*, 9H), 1.48–1.58 (*m*, 2H), 1.58–1.67 (*m*, 2H), 1.87–1.99 (*m*, 1H), 2.20–2.27 (*m*, 2H), 2.80–2.93 (*m*, 1H), 3.05–3.24 (*m*, 2H), 3.73, 3.74, 3.76, 3.77 (4 × *s*, 3H), 3.99 (*d*, *J* = 6.3 Hz, 0.206H), 4.04 (*d*, *J* = 6.9 Hz, 0.255H), 4.09 (*d*, *J* = 5.2 Hz, 0.284H), 4.15 (*d*, *J* = 7.0 Hz, 0.255H), 4.40–4.80 (*m*, 4H), 6.42–6.58 (*m*, 1H). ^13^C-NMR (126 MHz, CDCl_3_) for 4 diastereomers: *δ* 14.23, 22.80, 25.67, 25.70, 26.36, 26.74, 26.77, 26.83, 26.87, 27.08, 27.43, 28.48, 29.42, 29.44, 29.46, 29.48, 29.64, 29.66, 29.72, 29.73, 29.75, 29.78, 29.82, 30.43, 30.52, 32.04, 36.37, 36.43, 36.53, 36.60, 37.22, 37.42, 39.76, 39.79, 39.82, 39.83, 52.77, 52.92, 53.00, 53.15, 55.92, 56.15, 56.47, 58.05, 58.26, 58.39, 58.62, 76.25, 76.43, 76.48, 77.36, 79.56, 156.45, 168.14, 168.38, 168.43, 168.46, 173.58, 173.64, 173.71, 173.80, 203.24, 203.40, 203.67 (128 signals missing due to overlapping).

### 3.43. Synthesis of Methyl (4S)-2-(2-Nitro-1-phenylethyl)-3-oxo-4,7-distearamidoheptanoate (***17o***)

Following *GP3*, prepared from methyl (*S*)-3-oxo-4,7-distearamidoheptanoate (**2i**) (0.2 mmol, 144.1 mg) and *trans*-β-nitrostyrene (**12**) (0.3 mmol, 44.7 mg), organocatalyst **X**, 48 h; isolation by column chromatography (first column chromatography: EtOAc/petroleum ether = 1:1; second column chromatography: CH_2_Cl_2_/MeOH = 75:1). **17o** Yield: 50.5 mg (0.058 mmol, 29%, 4 diastereomers in a ratio of 24:22:30:24 in CDCl_3_) of light orange solid. EI-HRMS: *m*/*z* = 870.6936 (MH^+^); C_52_H_92_N_3_O_7_ requires *m*/*z* = 870.6930 (MH^+^); *ν*_max_ 3292, 2916, 2848, 1745, 1720, 1638, 1552, 1462, 1434, 1377, 1274, 1240, 1221, 1205, 1168, 1114, 755, 719, 700 cm^−1^. ^1^H-NMR (500 MHz, CDCl_3_) for 4 diastereomers: *δ* 0.88 (*t*, *J* = 6.9 Hz, 6H), 0.99–1.42 (*m*, 56H), 1.42–1.68 (*m*, 6H), 1.69–1.86 (*m*, 1H), 2.05–2.29 (*m*, 4H), 2.91–3.35 (*m*, 3H), 3.47, 3.48, 3.74, 3.77 (4 × *s*, 3H), 4.22–4.65 (*m*, 3H), 4.72–4.95 (*m*, 2H), 5.57–5.85 (*m*, 1H), 6.14 (*d*, *J* = 7.9 Hz, 0.244H), 6.56 (*d*, *J* = 7.9 Hz, 0.296H), 6.85 (br *s*, 0.225H), 6.93 (*d*, *J* = 7.1 Hz, 0.235H), 7.18–7.34 (*m*, 5H). ^13^C-NMR (126 MHz, CDCl_3_) for 4 diastereomers: *δ* 14.24, 22.81, 25.55, 25.60, 25.63, 25.67, 25.77, 25.91, 26.08, 26.27, 26.57, 26.77, 27.03, 27.27, 29.41, 29.43, 29.48, 29.50, 29.64, 29.66, 29.78, 29.80, 29.83, 32.04, 36.02, 36.38, 36.46, 36.89, 36.91, 38.52, 38.61, 38.68, 38.71, 42.54, 42.64, 43.09, 43.31, 52.83, 52.99, 53.14, 53.32, 58.08, 58.17, 58.19, 58.25, 58.27, 58.82, 58.95, 59.10, 77.29, 77.36, 77.56, 77.65, 77.69, 128.11, 128.16, 128.26, 128.36, 128.42, 128.49, 129.03, 129.17, 136.21, 136.33, 136.52, 136.60, 167.03, 167.21, 167.40, 167.69, 173.31, 173.74, 173.80, 174.07, 174.08, 174.21, 201.14, 201.89, 202.14, 203.10 (128 signals missing due to overlapping).

### 3.44. Synthesis of Methyl 2-((S)-2,5-Distearamidopentanoyl)-3-(nitromethyl)octadecanoate (***17p***)

Following *GP3* and *GP4*, prepared from methyl (*S*)-3-oxo-4,7-distearamidoheptanoate (**2i**) (0.2 mmol, 144.1 mg) and (*E*)-1-nitroheptadec-1-ene (**16**) (0.3 mmol, 85.0 mg), organocatalyst **X**, 48 h; isolation by column chromatography (1. EtOAc/petroleum ether = 1:3; 1. EtOAc/petroleum ether = 1:1); **17p** Yield: 50.2 mg (0.05 mmol, 25%, 4 diastereomers in a ratio of 25:25:27:23 in CDCl_3_) of light orange solid. Organocatalyst **VIII**, 48 h; isolation by column chromatography (1. EtOAc/petroleum ether = 1:3; 1. EtOAc/petroleum ether = 1:1); **17p** Yield: 46.2 mg (0.046 mmol, 23%, 4 diastereomers in a ratio of 39:11:12:38 in CDCl_3_) of light orange solid. EI-HRMS: *m*/*z* = 1004.8949 (MH^+^); C_61_H_118_N_3_O_7_ requires *m*/*z* = 1004.8964 (MH^+^); *ν*_max_ 3293, 2916, 2849, 1746, 1721, 1639, 1552, 1463, 1378, 1274, 1257, 1240, 1222, 1204, 719, 615 cm^−1^. ^1^H-NMR (500 MHz, CDCl_3_) for 4 diastereomers: *δ* 0.88 (*t*, *J* = 6.9, 9H), 1.07–1.46 (*m*, 88H), 1.48–1.70 (*m*, 7H), 1.83–1.98 (*m*, 1H), 2.14–2.21 (*m*, 2H), 2.21–2.30 (*m*, 2H), 2.81–2.92 (*m*, 1H), 3.17–3.28 (*m*, 1H), 3.28–3.40 (*m*, 1H), 3.73, 3.74, 3.76, 3.77 (4 × *s*, 3H), 3.98 (*d*, *J* = 5.9 Hz, 0.235H), 4.03 (*d*, *J* = 7.0 Hz, 0.270H), 4.08 (*d*, *J* = 5.2 Hz, 0.249H), 4.14 (*d*, *J* = 7.0 Hz, 0.246H), 4.37–4.78 (*m*, 3H), 5.79–5.89 (*m*, 1H), [6.72 (*d*, *J* = 7.6 Hz), 6.76 (*d*, *J* = 7.7 Hz), 6.77 (*d*, *J* = 7.8 Hz), 6.89 (*d*, *J* = 7.6 Hz); 1H]. ^13^C-NMR (126 MHz, CDCl_3_) for 4 diastereomers: *δ* 22.81, 25.65, 25.68, 25.69, 25.91, 25.93, 26.35, 26.41, 26.45, 26.49, 26.75, 26.79, 26.85, 26.89, 27.11, 27.40, 27.57, 29.44, 29.45, 29.49, 29.52, 29.57, 29.66, 29.67, 29.69, 29.71, 29.74, 29.76, 29.79, 29.81, 29.83, 30.44, 30.59, 32.05, 36.34, 36.39, 36.46, 36.48, 36.56, 36.92, 37.19, 37.40, 38.78, 38.81, 52.80, 52.94, 52.97, 53.16, 55.83, 55.89, 56.43, 56.55, 58.28, 58.41, 58.46, 58.56, 76.25, 76.50, 76.58, 77.36, 168.25, 168.36, 168.52, 173.80, 173.90, 173.94, 173.95, 174.02, 203.12, 203.63, 203.70 (173 signals missing due to overlapping).

### 3.45. Organocatalyzed Michael Addition of Pyrrolones ***11*** to Nitroalkene ***16***—General Procedure for the Preparation of Racemic Mixtures—General Procedure 5 (GP5)

To a solution/suspension of (*E*)-1-nitroheptadec-1-ene (**16**) (0.2 mmol, 56.7 mg; 1.0 equivalent) and the achiral organocatalyst **X** (0.04 mmol, 0.2 equivalents, 16.4 mg) in anhydrous toluene (1 mL) under argon at room temperature, pyrrolone **11** (0.3 mmol, 1.5 equivalents) was added, and the resulting reaction mixture was stirred at room temperature for 48–72 h. The volatiles were evaporated in vacuo, and the residue was purified by column chromatography (Silica gel 60, mobile phase). The fractions containing the pure racemic product ***rac*-18** were combined, and the volatiles were evaporated in vacuo. The product ***rac*-18** was fully characterized and analyzed by HPLC.

### 3.46. Organocatalyzed Michael Addition of Pyrrolones ***11*** to Nitroalkene ***16***—General Procedure for the Organocatalyzed Asymmetric Addition—General Procedure 6 (GP6)

To a solution/suspension of (*E*)-1-nitroheptadec-1-ene (**16**) (0.2 mmol, 56.7 mg; 1.0 equivalent) and the chiral organocatalyst **I** (0.02 mmol, 0.1 equivalents, 10.9 mg) in anhydrous toluene (1 mL) under argon at room temperature, pyrrolone **11** (0.3 mmol, 1.5 equivalents) was added, and the resulting reaction mixture was stirred at room temperature for 48–72 h. The volatiles were evaporated in vacuo, and the residue was purified by column chromatography (Silica gel 60, mobile phase). The fractions containing the pure chiral nonracemic product **18** were combined, and the volatiles were evaporated in vacuo. The product **18** was fully characterized and analyzed by HPLC.

### 3.47. Synthesis of 1-(Tert-butyl) 3-Methyl (S)-5-benzyl-5-((S)-1-nitroheptadecan-2-yl)-4-oxo-4,5-dihydro-1H-pyrrole-1,3-dicarboxylate (***18a***)

Following *GP5* and *GP6*, prepared from 1-(*tert*-butyl) 3-methyl 5-benzyl-4-oxo-4,5-dihydro-1*H*-pyrrole-1,3-dicarboxylate (**11a**) (0.3 mmol, 99.4 mg) and (*E*)-1-nitroheptadec-1-ene (**16**) (0.2 mmol, 56.7 mg), organocatalyst **I**, 48 h; isolation by column chromatography (EtOAc/petroleum ether = 1:5). ***rac*-18a** Yield: 94.7 mg (0.154 mmol, 77%, diastereomer 1/diastereomer 2 = 97:3 in CDCl_3_) of colorless oil. **18a** Yield: 84.8 mg (0.138 mmol, 69%, diastereomer 1/diastereomer 2 = 97:3 in CDCl_3_) of colorless oil. EI-HRMS: *m*/*z* = 615.3970 (MH^+^); C_35_H_55_N_2_O_7_ requires *m*/*z* = 615.4004 (MH^+^); *ν*_max_ 2923, 2853, 1732, 1713, 1581, 1554, 1497, 1456, 1438, 1370, 1295, 1224, 1143, 1089, 1063, 992, 914, 876, 843, 760, 725, 703, 628 cm^−1^. ^1^H-NMR (500 MHz, CDCl_3_) for diastereomer 1: *δ* 0.88 (*t*, *J* = 6.9 Hz, 3H), 1.07–1.43 (*m*, 26H), 1.44–1.85 (*m*, 11H), 3.26–3.54 (*m*, 3H), 3.77 (*s*, 3H), 4.31 (*dd*, *J* = 5.1, 13.7 Hz, 1H), 4.51 (br *s*, 1H), 6.95–7.03 (*m*, 2H), 7.11–7.21 (*m*, 3H), 8.60 (br *s*, 1H). ^1^H-NMR (500 MHz, CDCl_3_) for diastereomer 2: *δ* 3.71 (*s*, 3H). ^13^C-NMR (126 MHz, CDCl_3_) for diastereomer 1: *δ* 14.26, 22.83, 27.55, 27.74, 28.10, 29.50, 29.52, 29.61, 29.63, 29.73, 29.78, 29.79, 29.82, 29.83, 32.06, 39.94, 43.20, 51.92, 76.56, 85.81, 112.39, 127.69, 128.43, 129.63, 133.25, 147.60, 161.78, 164.91, 196.43 (2 signals missing due to overlapping). HPLC: Chiralpak IA-3, *n*-Hexane/*i*PrOH = 95:5, flow rate 1.0 mL/min, λ = 210 nm, T = 25 °C. Major diastereomer: enantiomers: *t*R = 9.350 min (major); 13.217 min (minor)—82% *ee*.

### 3.48. Synthesis of 1-(Tert-butyl) 3-Methyl 5-(4-(((Benzyloxy)carbonyl)amino)butyl)-5-(1-nitroheptadecan-2-yl)-4-oxo-4,5-dihydro-1H-pyrrole-1,3-dicarboxylate (***18b***)

Following *GP5* and *GP6*, prepared from 1-(*tert*-butyl) 3-methyl 5-(4-(((benzyloxy)carbonyl)amino)butyl)-4-oxo-4,5-dihydro-1*H*-pyrrole-1,3-dicarboxylate (**11b**) (0.3 mmol, 134.0 mg) and (*E*)-1-nitroheptadec-1-ene (**16**) (0.2 mmol, 56.7 mg), organocatalyst **I**, 72 h; isolation by column chromatography (EtOAc/petroleum ether = 1:2). ***rac*-18b** Yield: 94.9 mg (0.130 mmol, 65%, diastereomer 1/diastereomer 2 = 90:10 in CDCl_3_) of colorless oil. **18b** Yield: 26.3 mg (0.036 mmol, 18%, diastereomer 1/diastereomer 2 = 26:74 in CDCl_3_) of colorless oil. EI-HRMS: *m*/*z* = 730.4644 (MH^+^); C_40_H_64_N_3_O_9_ requires *m*/*z* = 730.4637 (MH^+^); *ν*_max_ 3675, 3369, 2923, 2854, 1711, 1582, 1553, 1455, 1438, 1394, 1371, 1280, 1226, 1140, 1067, 846, 763, 697 cm^−1^. ^1^H-NMR (500 MHz, CDCl_3_) for diastereomer 1: *δ* 0.88 (*t*, *J* = 6.9 Hz, 3H), 0.91–1.03 (*m*, 2H), 1.06–1.34 (*m*, 27H), 1.35–1.49 (*m*, 4H), 1.56 (*s*, 9H), 1.96–2.07 (*m*, 1H), 2.10–2.22 (*m*, 1H), 3.03–3.17 (*m*, 3H), 3.84 (*s*, 3H), 4.22 (*dd*, *J* = 5.3, 13.8 Hz, 1H), 4.30–4.41 (*m*, 1H), 4.71 (*t*, *J* = 6.2 Hz, 1H), 5.06 (*s*, 2H), 7.27–7.39 (*m*, 5H). ^1^H-NMR (500 MHz, CDCl_3_) for diastereomer 2: *δ* 1.58 (*s*, 9H), 1.87–1.96 (*m*, 1H), 3.28–3.36 (*m*, 1H), 3.83 (*s*, 3H), 4.28 (*dd*, *J* = 5.3, 14.6 Hz, 1H), 5.18 (*dd*, *J* = 5.5, 14.7 Hz, 1H), 9.19 (*s*, 1H). ^13^C-NMR (126 MHz, CDCl_3_) for diastereomer 1: *δ* 14.26, 20.18, 22.82, 27.41, 27.49, 28.02, 29.49, 29.55, 29.62, 29.71, 29.77, 29.78, 29.81, 29.82, 32.05, 33.87, 40.61, 43.36, 5 min 2.03, 66.78, 76.35, 77.36, 86.22, 112.22, 128.24, 128.64, 136.59, 147.42, 156.40, 161.95, 165.24, 196.41 (4 signals missing due to overlapping). ^13^C-NMR (126 MHz, CDCl_3_) for diastereomer 1 and diastereomer 2 (diastereomer 1/diastereomer 2 = 26:74): *δ* 14.26, 20.19, 20.45, 22.82, 27.18, 27.50, 27.91, 28.03, 28.12, 29.44, 29.49, 29.50, 29.56, 29.63, 29.67, 29.72, 29.75, 29.79, 29.82, 29.83, 32.05, 33.89, 40.66, 41.98, 43.37, 52.03, 52.05, 66.79, 74.38, 76.11, 76.36, 77.40, 86.21, 112.23, 112.31, 128.25, 128.65, 136.59, 147.43, 156.39, 161.92, 165.29, 196.41, 197.67 (28 signals missing due to overlapping). HPLC: Chiralpak IA-3, *n*-Hexane/*i*PrOH = 90:10, flow rate 1.0 mL/min, λ = 210 nm, T = 25 °C. Major diastereomer: enantiomers: *t*R = 24.088 min (minor); 31.095 min (major)—57% *ee*. Minor diastereomer: enantiomers: *t*R = 26.634 min (minor); 30.099 min (major)—11% *ee*.

### 3.49. Synthesis of 1-(Tert-butyl) 3-Methyl 5-(3-(benzyloxy)-3-oxopropyl)-5-(1-nitroheptadecan-2-yl)-4-oxo-4,5-dihydro-1H-pyrrole-1,3-dicarboxylate (***18c***)

Following *GP5* and *GP6*, prepared from 1-(*tert*-butyl) 3-methyl 5-(3-(benzyloxy)-3-oxopropyl)-4-oxo-4,5-dihydro-1*H*-pyrrole-1,3-dicarboxylate (**11c**) (0.3 mmol, 121.0 mg) and (*E*)-1-nitroheptadec-1-ene (**16**) (0.2 mmol, 56.7 mg), organocatalyst **I**, 48 h; isolation by column chromatography (EtOAc/petroleum ether = 1:5). ***rac*-18c** Yield: 92.0 mg (0.134 mmol, 67%, diastereomer 1/diastereomer 2 = 93:7 in CDCl_3_) of colorless oil. **18c** Yield: 42.6 mg (0.062 mmol, 31%, diastereomer 1/diastereomer 2 = 31:69 in CDCl_3_) of colorless oil. EI-HRMS: *m*/*z* = 687.4224 (MH^+^); C_38_H_59_N_2_O_9_ requires *m*/*z* = 687.4215 (MH^+^); *ν*_max_ 3675, 2923, 2854, 1735, 1713, 1582, 1554, 1439, 1393, 1371, 1279, 1256, 1227, 1141, 1077, 846, 800, 752, 698 cm^−1^. ^1^H-NMR (600 MHz, CDCl_3_) for diastereomer 1: *δ* 0.88 (*t*, *J* = 6.9 Hz, 3H), 1.17–1.34 (*m*, 27H), 1.35–1.46 (*m*, 1H), 1.56 (*s*, 9H), 1.98–2.06 (*m*, 1H), 2.09–2.17 (*m*, 1H), 2.37–2.45 (*m*, 1H), 2.48–2.55 (*m*, 1H), 3.13–3.20 (*m*, 1H), 3.83 (*s*, 3H), 4.25 (*dd*, *J* = 5.6, 13.8 Hz, 1H), 4.30–4.39 (*m*, 1H), 5.07 (*s*, 2H), 7.28–7.39 (*m*, 5H), 9.08 (*s*, 1H). ^1^H-NMR (500 MHz, CDCl_3_) for diastereomer 2: *δ* 1.58 (*s*, 9H), 2.26–2.33 (*m*, 1H), 2.54–2.62 (*m*, 1H), 3.35–3.41 (*m*, 1H), 3.82 (*s*, 3H), 5.06 (*s*, 2H), 5.19 (*dd*, *J* = 5.1, 14.7 Hz, 1H), 9.17 (*s*, 1H). ^13^C-NMR (151 MHz, CDCl_3_) for diastereomer 1: *δ* 14.26, 22.83, 27.48, 27.60, 28.01, 29.13, 29.49, 29.50, 29.54, 29.62, 29.72, 29.77, 29.79, 29.82, 29.84, 32.06, 43.15, 52.06, 66.88, 75.92, 76.42, 86.62, 112.16, 128.44, 128.47, 128.56, 128.75, 135.56, 147.29, 161.76, 165.19, 171.48, 195.61 (1 signal missing due to overlapping). ^13^C-NMR (126 MHz, CDCl_3_) for diastereomer 1 and diastereomer 2 (diastereomer 1/diastereomer 2 = 31:69): *δ* 14.28, 22.84, 27.17, 27.49, 28.02, 28.07, 28.11, 28.37, 29.28, 29.44, 29.50, 29.56, 29.64, 29.68, 29.73, 29.76, 29.80, 29.83, 29.85, 32.07, 41.85, 43.16, 52.06, 52.08, 66.89, 66.93, 74.46, 75.26, 75.93, 76.42, 77.39, 86.67, 112.15, 112.36, 128.45, 128.48, 128.58, 128.77, 135.51, 135.56, 147.29, 161.72, 161.77, 165.20, 165.34, 171.18, 171.49, 195.63, 196.88 (19 signal missing due to overlapping). HPLC: Chiralpak IA-3, *n*-Hexane/*i*PrOH = 90:10, flow rate 1.0 mL/min, λ = 280 nm, T = 25 °C. Major diastereomer: enantiomers: *t*R = 10.649 min (major); 12.074 min (minor)—68% *ee*. Minor diastereomer: enantiomers: *t*R = 9.991 min (minor); 12.514 min (major)—38% *ee*.

### 3.50. Synthesis of 4-Hydroxy-3-(1-nitroheptadecan-2-yl)furan-2(5H)-one (***20***)

To a solution of (*E*)-1-nitroheptadec-1-ene (**16**) (0.2 mmol, 56.7 mg) and the achiral organocatalyst **X** (0.04 mmol, 0.2 equivalents, 16.4 mg) in anhydrous CH_2_Cl_2_ (1 mL) under argon at room temperature, tetronic acid (**19**) (0.3 mmol, 30.0 mg) was added, and the resulting reaction mixture was stirred at room temperature for 72 h. The volatiles were evaporated in vacuo, and the residue was purified by column chromatography (Silica gel 60, EtOAc/petroleum ether = 1:1). The fractions containing the pure product **20** were combined, and the volatiles were evaporated in vacuo. Yield: 33.8 mg (0.088 mmol, 44%) of white solid; m.p. = 86.8–88.2 °C. EI-HRMS: *m*/*z* = 384.2741 (MH^+^); C_21_H_38_NO_5_ requires *m*/*z* = 384.2744 (MH^+^); *ν*_max_ 2918, 2850, 1714, 1611, 1547, 1428, 1381, 1348, 1279, 1260, 1129, 1097, 1044, 971, 954, 780, 720, 682, 658 cm^−1^. ^1^H-NMR (500 MHz, CDCl_3_): *δ* 0.88 (*t*, *J* = 6.9 Hz, 3H), 1.09–1.35 (*m*, 26H), 1.50–1.60 (*m*, 1H), 1.65–1.77 (*m*, 1H), 3.37 (*tt*, *J* = 5.6, 9.6 Hz, 1H), 4.53 (*dd*, *J* = 5.6, 12.4 Hz, 1H), 4.71–4.81 (*m*, 3H), 10.97 (br *s*, 1H). ^13^C-NMR (126 MHz, CDCl_3_): *δ* 14.27, 22.84, 27.28, 29.44, 29.51, 29.62, 29.76, 29.79, 29.81, 29.84, 29.86, 29.99, 32.07, 32.87, 68.14, 76.88, 99.10, 177.35, 177.91 (2 signals missing due to overlapping).

*X-ray Crystallography.* Single-crystal X-ray diffraction data were collected on an Agilent Technologies SuperNova Dual Diffractometer with an Atlas detector using monochromated Mo-K_α_ radiation (λ = 0.71073 Å) at 150 K. The data were processed using CrysAlis PRO 1.171.43.121a (Rigaku Oxford Diffraction, 2024) [71]. Using Olex2.1.2. [72], the structure was solved by direct methods implemented in SHELXS-2014/7 [73] or SHELXT-2014/7 [74] and refined by a full-matrix least-squares procedure based on F^2^ with SHELXT-2014/7 [75]. All non-hydrogen atoms were refined anisotropically. Hydrogen atoms were placed in geometrically calculated positions and were refined using a riding model. The drawing and the analysis of bond lengths, angles, and intermolecular interactions were carried out using Mercury [76] and Platon [77]. Structural and other crystallographic details on data collection and refinement for compound **17a** have been deposited with the Cambridge Crystallographic Data Centre as supplementary publication number CCDC Deposition Number 2513139. These data can be obtained free of charge via https://www.ccdc.cam.ac.uk/structures/ (accessed on 3 January 2026) (or from the CCDC, 12 Union Road, Cambridge CB2 1EZ, UK; fax: +44-1223-336033; e-mail: deposit@ccdc.cam.ac.uk).

## 4. Conclusions

Glycine-, β-alanine-, lysine-, glycolic acid-, palmitic acid-, stearic acid-, and linoleic acid-derived β-keto esters **2a**–**i**, as well as phenylalanine-, ornithine-, and glutamic acid-derived pyrrolones **11a**–**c** and tetronic acid (**19**), were used as nucleophiles for the organocatalyzed addition to *trans*-β-nitrostyrene (**12**) and the palmitic acid-derived nitroalkene **16** electrophiles. All racemic products ***rac*-17a**–**l**, ***rac*-18a**–**c**, and **20** were prepared using the achiral organocatalyst **X**. For products with HPLC-separable stereomers, chiral nonracemic 1,4-adducts **17a**,**b**,**d**,**f**,**h**,**j**,**l** and **18a**–**c** were resynthesized using bifunctional noncovalent organocatalysts **I** and/or **VIII**. The doubly fatty acid-derived products ***rac*-17g**,**i**,**k** could not be separated on chiral HPLC columns. β-Keto ester adducts **17a**,**b**,**d**,**f**,**h**,**j**,**l** were prepared with high enantioselectivity (91–98% *ee*) but low diastereoselectivity (*dr* up to 36:64) due to the epimerizable C-2 stereocenter. The (1*S*,2*S*)-absolute configuration of the stereoisomer of adduct **17a** (prepared with organocatalyst **I**) was determined by single-crystal X-ray diffraction analysis. Based on this, we assigned the (1*R*)-absolute configuration to the major enantiomer of both diastereomers of products **17a**, **17b**, **17d**, **17f**, **17h**, **17j**, and **17l** prepared with organocatalyst **VIII**. Products **17m**–**p**, prepared from the addition of lysine-derived β-keto esters **2h** and **2i**, showed low diastereoselectivity with both achiral **X** and chiral organocatalyst **VIII**. In the pyrrolone series, the best diastereoselectivity (*dr* = 97:3) and enantioselectivity (82% *ee* for the major diastereomer) were obtained with phenylalanine-derived pyrrolone **11a**, yielding adduct **18a**. The (1′*S*,5*S*)-absolute configuration of the major diastereomer of product **18a** was assigned based on our previous results in the series of 1,4-adducts of pyrrolones to nitroalkene acceptors [22]. Despite certain drawbacks, such as low diastereoselectivity and challenges in separating stereomers on chiral HPLC columns, this study demonstrates the ease of assembling readily available amino acid and fatty acid building blocks under ambient conditions for the synthesis of interesting classes of products.

## Data Availability

No new data were created or analyzed in this study. Data sharing is not applicable to this article.

## Supplementary Materials

The following supporting information can be downloaded at: https://www.mdpi.com/article/10.3390/molecules31020204/s1, copies of ^1^H- and ^13^C-NMR spectra; HPLC data, X-ray diffraction data. Scheme S1. Evaluation of organocatalysts **I**–**IX** in Michael addition of *β*-keto ester **2b** (0.2 mmol) to *trans*-β-nitrostyrene (**12**). Scheme S2. Optimization of the reaction conditions—solvent. Scheme S3. Evaluation of organocatalysts **I** and **VIII**. Scheme S4. Scope of the organocatalyzed addition of β-keto esters **2** to *trans*-β-nitrostyrene (**12**) and fatty acid-derived nitroalkene **16**. Scheme S5. Scope of the organocatalyzed addition of pyrrolones **11** to fatty acid-derived nitroalkene **16**. Table S1. Crystal data and structure refinement for compound **17a**. Figure S1. Molecular structure of product **17a**. Thermal ellipsoids are shown at 50% probability.
