# Supplementary material for: Fatty Acid and Amino Acid Derivatives in Organocatalyzed Michael Additions
Source: Molecules. 2026 Jan 6;31(2):204. doi: 10.3390/molecules31020204 (PMC12844414; doi:10.3390/molecules31020204)
Supplement: Supplementary file 1 [file molecules-31-00204-s001.zip › molecules-4075836-supplementary.pdf]

SUPPORTING INFORMATION

Fatty Acid and Amino Acid Derivatives in Organocatalyzed Michael Additions

Aljaž Flis, Helena Brodnik, Nejc Petek, Franc Požgan, Jurij Svete, Bogdan Štefane, Luka Ciber * and Uroš Grošelj *

Faculty of Chemistry and Chemical Technology, University of Ljubljana, Večna pot 113, SI-1000 Ljubljana, Slovenia
* Correspondence: luka.ciber@fkkt.uni-lj.si (L.C.); uros.groselj@fkkt.uni-lj.si (U.G.); Tel.: +386-1-479-8565 (U.G.)

Table of contents

1. Materials and methods, syntheses, and characterization 2
2. Catalyst and solvent optimization, HPLC data 39
3. ^{1}H- and ^{13}C-NMR spectra 78
4. Structure determination by X-ray diffraction analysis 128
5. References 130

1. Materials and methods, syntheses, and characterization

Solvents for extractions and chromatography were of technical grade and were distilled prior to use. Extracts were dried over technical grade anhydrous Na_2SO_4. Melting points were determined on a Kofler micro hot stage and on SRS OptiMelt MPA100 – Automated Melting Point System (Stanford Research Systems, Sunnyvale, California, United States). The NMR spectra were obtained on a Bruker UltraShield 500 plus spektrometer and on a BRUKER AVANCE NEO 600 MHz NMR spektrometer (Bruker, Billerica, Massachusetts, United States) at 500 and 600 MHz for ^{1}H and 126 and 150 MHz for ^{13}C nucleus, respectively, using DMSO-d_6 and $CDCl_3$ with TMS as the internal standard, as solvents. Mass spectra were recorded on an Agilent 6224 Accurate Mass TOF LC/MS (Agilent Technologies, Santa Clara, California, United States), IR spectra on a Perkin-Elmer Spectrum BX FTIR spectrophotometer (PerkinElmer, Waltham, Massachusetts, United States). Column chromatography (CC) was performed on silica gel (Silica gel 60, particle size: 0.035-0.070 mm (Sigma-Aldrich, St. Louis, Missouri, United States)). HPLC analyses were performed on an Agilent 1260 Infinity LC (Agilent Technologies, Santa Clara, California, United States) using CHIRALPAK IA-3 (0.46 cm ø × 25 cm), CHIRALPAK AD-H (0.46 cm ø × 25 cm), CHIRALCEL OD-H (0.46 cm ø × 25 cm), and CHIRALPAK AS-H (0.46 cm ø × 25 cm) as chiral column (CHIRAL TECHNOLOGIES, INC., West Chester, Pennsylvania, United States). All the commercially available chemicals used were purchased from Sigma-Aldrich (St. Louis, Missouri, United States).

Organocatalysts **I**[1], **II**[2], **III**[3], **IV**[3], **VI**[4], **VII**[5], **VIII**[6], and **IX**[7] were prepared following the literature procedures; organocatalyst **V** was purchased from Sigma-Aldrich.

Synthesis of β-keto esters 2 from carboxylic acids 1 – *General procedure 1 (GP1)*

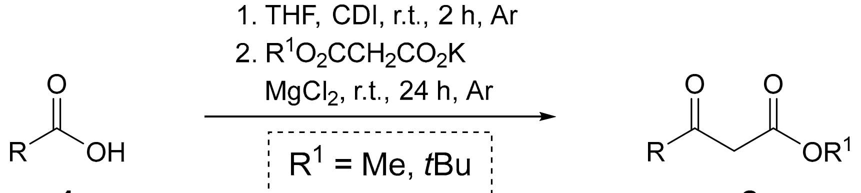

1 **2**

To a solution or suspension of carboxylic acid **1** (10 mmol) in anhydrous THF (50 mL), 1,1'-carbonyldiimidazole (CDI; 12 mmol, ω = 0.97, 1.672 g) was added under argon, and the resulting reaction mixture was stirred for 2 h at room temperature. A solid mixture of $MgCl_2$ (9.8 mmol, ω = 0.98, 952 mg) and methyl potassium malonate (15 mmol, 2.343 g) or *tert*-butyl potassium malonate (15 mmol, ω = 0.95, 3.130 g) was then added. The reaction mixture was stirred for a further 24 hours under argon at room temperature. The volatiles were evaporated *in vacuo*, the residue was dissolved in EtOAc (150 mL) and washed with $NaHSO_4$ (1 M in H_2O, 3×50 mL), $NaHCO_3$ (aq. sat., 2×20 mL), and NaCl (aq. sat., 2×50 mL). The organic phase was dried over anhydrous Na_2SO_4, filtered, and the volatiles evaporated *in vacuo*. If necessary, the residue was purified by column chromatography (CC, Silica gel 60). The fractions containing the product were combined and the volatiles were evaporated *in vacuo*.

Synthesis of methyl 4-((*tert*-butoxycarbonyl)amino)-3-oxobutanoate (2a)[8]

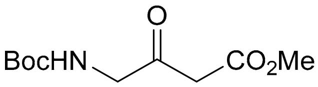

Following *GP1*. Prepared from (*tert*-butoxycarbonyl)glycine (**1a**) (10 mmol, 1.752 g), methyl potassium malonate (15 mmol, 2.343 g); isolation by extraction. Yield: 2.10 g (9.1 mmol, 91 %) of yellowish oil. ^{1}H-NMR (500 MHz, DMSO-d_6): δ 1.39 (*s*, 9H), 3.60 (*s*, 2H), 3.63 (*s*, 3H), 3.86 (*d*, J=5.9 Hz, 2H), 7.13 (*t*, J=5.9 Hz, 1H). ^{13}C-NMR (126 MHz, DMSO-d_6): δ 28.18, 45.91, 49.81, 51.93, 78.30, 155.81, 167.50, 200.67.

Synthesis of methyl 5-((*tert*-butoxycarbonyl)amino)-3-oxopentanoate (2b)[8]

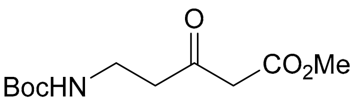

Following *GP1*, prepared from Boc-β-alanine (**1b**) (10 mmol, 1.892 g), methyl potassium malonate (15 mmol, 2.343 g); isolation by extraction. Yield: 2.18 g (8.9 mmol, 89 %) of yellowish oil. ^{1}H-NMR (500 MHz, DMSO-d_6): δ 1.36 (*s*, 9H), 2.66 (*t*, J=6.9 Hz, 2H), 3.11 (*q*,

J=6.9 Hz, 2H), 3.61 (*s*, 2H), 3.62 (*s*, 3H), 6.77 (*t*, *J*=5.6 Hz, 1H). ^{13}C-NMR (126 MHz, DMSO-d_6): *δ* 28.24, 34.88, 42.40, 48.68, 51.84, 77.72, 155.53, 167.71, 202.42.

Synthesis of methyl 4-((3-methylbut-2-en-1-yl)oxy)-3-oxobutanoate (2c)[9]

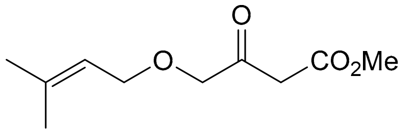

Following *GP1*. Prepared from 2-((3-methylbut-2-en-1-yl)oxy)acetic acid (**1c**)[10] (10 mmol, 1.442 g), methyl potassium malonate (15 mmol, 2.343 g); isolation by extraction. Yield: 1.602 g (8.0 mmol, 80 %) of colorless oil. ^{1}H-NMR (500 MHz, $CDCl_3$): *δ* 1.69 (*s*, 3H), 1.77 (*s*, 3H), 3.55 (*s*, 2H), 3.74 (*s*, 3H), 4.04 (*d*, *J*=7.1 Hz, 2H), 4.09 (*s*, 2H), 5.29 – 5.36 (*m*, 1H). ^{13}C-NMR (126 MHz, $CDCl_3$): *δ* 18.11, 25.88, 45.77, 52.41, 67.89, 74.63, 119.97, 138.70, 167.62, 202.30.

Synthesis of methyl 3-oxooctadecanoate (2d)[11]

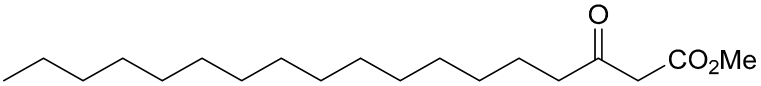

Following *GP1*. Prepared from palmitic acid (**1d**) (10 mmol, 2.564 g), methyl potassium malonate (15 mmol, 2.343 g); isolation by extraction and column chromatography (EtOAc/petroleum ether = 1:10). Yield: 2,717 g (8.70 mmol, 87 %) of white solid; m.p. = 49.2–50.5 °C. ^{1}H-NMR (500 MHz, $CDCl_3$): *δ* 0.88 (*t*, *J*=6.9 Hz, 3H), 1.19 – 1.35 (*m*, 24H), 1.53 – 1.65 (*m*, 2H), 2.53 (*t*, *J*=7.4 Hz, 2H), 3.45 (*s*, 2H), 3.74 (*s*, 3H). ^{13}C-NMR (126 MHz, $CDCl_3$): *δ* 14.26, 22.83, 23.60, 29.14, 29.49, 29.50, 29.58, 29.73, 29.77, 29.79, 29.80, 29.82, 29.83, 32.06, 43.23, 49.15, 52.46, 167.85, 203.01.

Synthesis of methyl 3-oxoicosanoate (2e)[11]

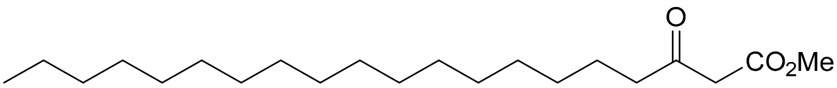

Following *GP1*. Prepared from stearic acid (**1e**) (10 mmol, 2.845 g), methyl potassium malonate (15 mmol, 2.343 g); isolation by extraction and column chromatography (EtOAc/petroleum ether = 1:10). Yield: 2.282 g (6.70 mmol, 67 %) of white solid; m.p. = 49.5 –51.2 °C. ^{1}H-NMR (500 MHz, $CDCl_3$): *δ* 0.88 (*t*, *J*=6.9 Hz, 3H), 1.18 – 1.34 (*m*, 28H), 1.55 – 1.63 (*m*, 2H), 2.53 (*t*, *J*=7.4 Hz, 2H), 3.45 (*s*, 2H), 3.74 (*s*, 3H). ^{13}C-NMR (151 MHz, $CDCl_3$): *δ* 14.25, 22.83, 23.60, 29.14, 29.49, 29.50, 29.58, 29.73, 29.78, 29.79, 29.80, 29.83, 32.06, 43.22, 49.14, 52.45, 167.84, 203.00 (3 signals missing due to overlapping).

Synthesis of *tert*-butyl 3-oxoicosanoate (2f)

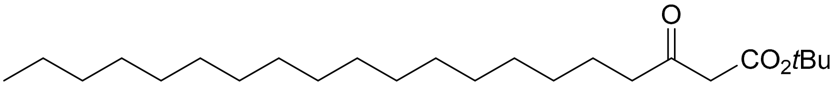

Following *GP1*. Prepared from stearic acid (**1e**) (10 mmol, 2.845 g), *tert*-butyl potassium malonate (15 mmol, ω = 0.95, 3.130 g); isolation by extraction and column chromatography (EtOAc/petroleum ether = 1:15). Yield: 1.722 g (4.50 mmol, 45 %) of white solid; m.p. = 36.9–38.1 °C. EI-HRMS: *m/z* = 327.2885 (MH^+-*t*BuOH); $C_{20}H_{39}O_3$ requires: *m/z* = 327.2894 (MH^+-*t*BuOH); ν_{max} 2960, 2916, 2849, 1729, 1715, 1466, 1406, 1367, 1329, 1276, 1260, 1155, 1131, 1109, 1080, 947, 920, 842, 790, 723, 647 cm^{-1}. ^{1}H-NMR (500 MHz, $CDCl_3$): δ 0.88 (*t*, *J*=6.9 Hz, 3H), 1.21 – 1.34 (*m*, 28H), 1.47 (*s*, 9H), 1.55 – 1.61 (*m*, 2H), 2.51 (*t*, *J*=7.4 Hz, 2H), 3.34 (*s*, 2H). ^{13}C-NMR (151 MHz, $CDCl_3$): δ 14.26, 22.83, 23.62, 28.10, 29.21, 29.50, 29.52, 29.59, 29.74, 29.78, 29.80, 29.81, 29.84, 32.07, 43.09, 50.81, 81.99, 166.69, 203.68 (3 signals missing due to overlapping).

Synthesis of methyl (11*Z*,14*Z*)-3-oxoicosa-11,14-dienoate (2g)[11]

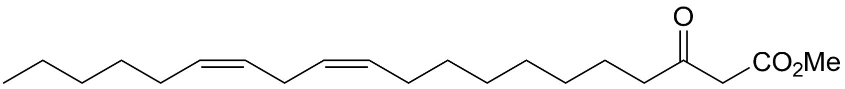

Following *GP1*. Prepared from linoleic acid (**1f**) (10 mmol, 2.804 g), methyl potassium malonate (15 mmol, 2.343 g); isolation by extraction and column chromatography (EtOAc/petroleum ether = 1:5). Yield: 1.851 g (5.50 mmol, 55 %) of colorless oil. ^{1}H-NMR (500 MHz, $CDCl_3$): δ 0.89 (*t*, *J*=6.9 Hz, 3H), 1.23 – 1.40 (*m*, 14H), 1.54 – 1.64 (*m*, 2H), 1.97 – 2.09 (*m*, 4H), 2.53 (*t*, *J*=7.4 Hz, 2H), 2.77 (*t*, *J*=6.6 Hz, 2H), 3.45 (*s*, 2H), 3.74 (*s*, 3H), 5.28 – 5.43 (*m*, 4H). ^{13}C-NMR (126 MHz, $CDCl_3$): δ 14.21, 22.71, 23.57, 25.76, 27.31, 27.33, 29.10, 29.21, 29.39, 29.48, 29.72, 31.66, 43.20, 49.15, 52.46, 128.02, 128.19, 130.15, 130.35, 167.83, 202.95.

Synthesis of methyl (*S*)-4-((*tert*-butoxycarbonyl)amino)-3-oxo-5-phenylpentanoate (10a)[8]

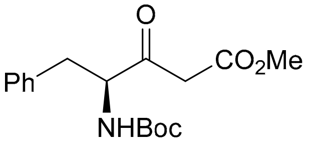

Following *GP1*. Prepared from Boc-L-phenylalanine (**9a**) (10 mmol, 2.653 g), methyl potassium malonate (15 mmol, 2.343 g); isolation by extraction. Yield: 2.346 g (7.30 mmol, 73 %) of colorless oil. ^{1}H-NMR (500 MHz, $CDCl_3$): δ 1.40 (*s*, 9H), 2.98 (*dd*, *J*=7.5, 14.1 Hz, 1H),

3.14 (*dd*, *J*=6.2, 14.1 Hz, 1H), 3.46 (*d*, *J*=16.0 Hz, 1H), 3.52 (*d*, *J*=16.0 Hz, 1H), 3.71 (*s*, 3H), 4.56 (*q*, *J*=7.2 Hz, 1H), 4.96 – 5.07 (*m*, 1H), 7.14 – 7.20 (*m*, 2H), 7.21 – 7.35 (*m*, 3H).

Synthesis of methyl (*S*)-8-(((benzyloxy)carbonyl)amino)-4-((*tert*-butoxycarbonyl)amino)-3-oxooctanoate (10b)[8]

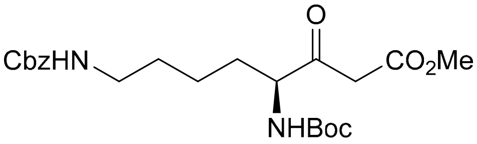

Following *GP1*. Prepared from Boc-Lys(Z)-OH (**9b**) (10 mmol, 3.804 g), methyl potassium malonate (15 mmol, 2.343 g); isolation by extraction. Yield: 3.317 g (7.60 mmol, 76 %) of colorless oil. ^{1}H-NMR (500 MHz, $CDCl_3$): *δ* 1.30 – 1.65 (*m*, 5H), 1.43 (*s*, 9H), 1.81 – 1.93 (*m,* 1H), 3.12 – 3.27 (*m*, 2H), 3.54 (*d*, *J*=15.7 Hz, 1H), 3.59 (*d*, *J*=16.0 Hz, 1H), 3.73 (*s*, 3H), 4.27 – 4.36 (*m*, 1H), 4.93 (*t*, *J*=6.0 Hz, 1H), 5.05 – 5.17 (*m*, 2H), 5.27 (br *d*, *J*=7.7 Hz, 1H), 7.28 – 7.39 (*m*, 5H).

Synthesis of 7-benzyl 1-methyl (*S*)-4-((*tert*-butoxycarbonyl)amino)-3-oxoheptanedioate (10c)[12]

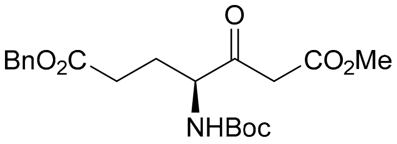

Following *GP1*. Prepared from Boc-Glu(OBzl)-OH (**9c**) (10 mmol, 3.374g), methyl potassium malonate (15 mmol, 2.343 g); isolation by extraction. Yield: 2.675 g (6.80 mmol, 68 %) of colorless oil. ^{1}H-NMR (500 MHz, $CDCl_3$): *δ* 1.43 (*s*, 9H), 1.80 – 1.91 (*m*, 1H), 2.22 – 2.32 (*m*, 1H), 2.38 – 2.56 (*m*, 2H), 3.55 – 3.65 (*m*, 2H), 3.73 (*s*, 3H), 4.38 – 4.45 (*m*, 1H), 5.12 (*s*, 2H), 5.23 (br *d*, *J*=8.1 Hz, 1H), 7.29 – 7.41 (*m*, 5H).

Synthesis of methyl (*S*)-5-((*tert*-butoxycarbonyl)amino)-2-stearamidopentanoate (4)

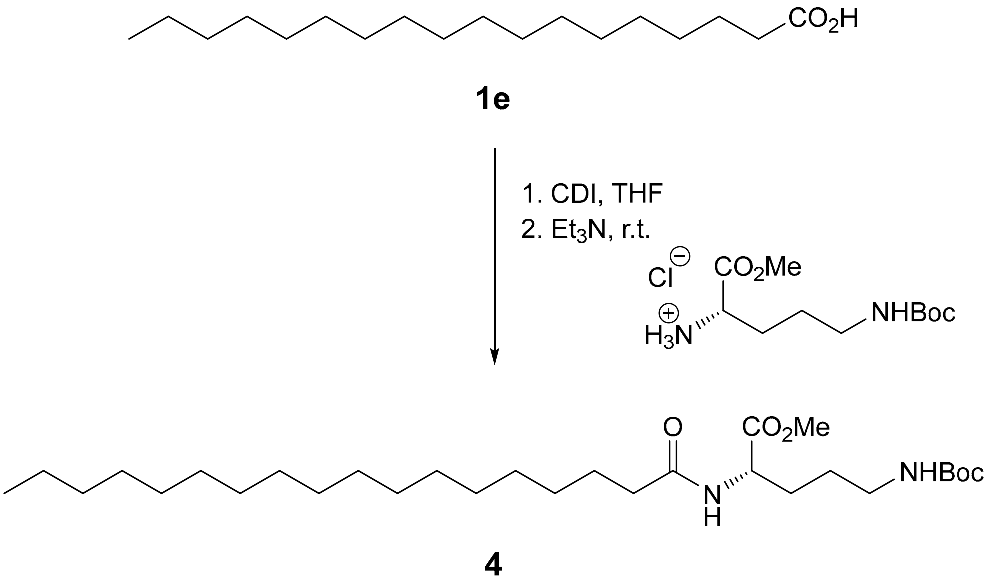

To a solution of stearic acid (**1e**) (20 mmol, 5.690 g) in anhydrous THF (50 mL) was added 1,1'-carbonyldiimidazole (CDI; 22 mmol, ω = 0.97, 3.678 g) under argon and the resulting reaction mixture was stirred for 2 h at room temperature. Then H-Orn(Boc)-OMe×HCl (**3**) (22 mmol, ω = 0.96, 6.480 g) and Et_3N (22 mmol, 3.07 mL) were added. The reaction mixture was stirred for a further 24 hours under argon at room temperature. The volatiles were evaporated *in vacuo*, the residue was dissolved in CH_2Cl_2 (150 mL) and washed with $NaHSO_4$ (1 M in H_2O, 4×50 mL), $NaHCO_3$ (aq. sat., 3×20 mL) and NaCl (aq. sat., 2×50 mL). The organic phase was dried over anhydrous Na_2SO_4, filtered and the volatiles evaporated *in vacuo*. Yield: 7.076 g (13.8 mmol, 69 %) of white solid; m.p. = 86.0–88.0 °C. EI-HRMS: *m/z* = 513.4272 (MH^+); $C_{29}H_{57}N_2O_5$ requires: *m/z* = 513.4262 (MH^+); ν_{max} 3345, 2915, 2847, 1762, 1684, 1648, 1526, 1473, 1462, 1369, 1285, 1252, 1212, 1171, 1143, 1047, 994, 950, 871, 754, 729, 719, 654 cm^{-1}. ^{1}H-NMR (500 MHz, DMSO-d_6): δ 0.85 (*t*, *J*=6.8 Hz, 3H), 1.14 – 1.31 (*m*, 30H), 1.37 (*s*, 9H), 1.42 – 1.57 (*m*, 3H), 1.61 – 1.69 (*m*, 1H), 2.09 (*t*, *J*=7.1 Hz, 2H), 2.89 (*q*, *J*=6.8 Hz, 2H), 3.60 (*s*, 3H), 4.14 – 4.23 (*m*, 1H), 6.79 (*t*, *J*=5.7 Hz, 1H), 8.14 (*d*, *J*=7.5 Hz, 1H). ^{13}C-NMR (126 MHz, DMSO-d_6): δ 13.98, 22.11, 25.23, 26.02, 28.20, 28.26, 28.55, 28.72, 28.78, 28.97, 29.02, 29.05, 31.31, 34.94, 51.65, 51.70, 77.40, 155.58, 172.43, 172.78 (7 signals missing due to overlapping).

Synthesis of (*S*)-5-((*tert*-butoxycarbonyl)amino)-2-stearamidopentanoic acid (5)

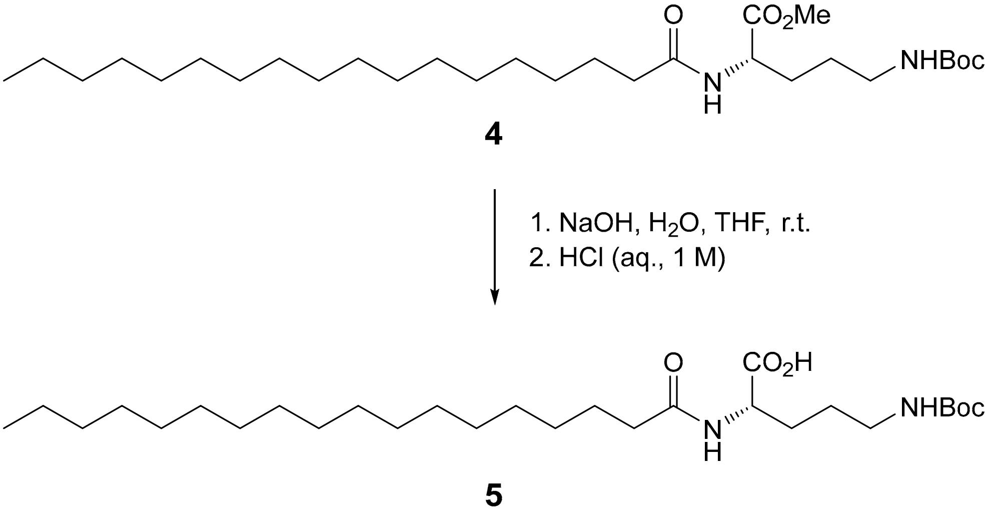

To a solution/suspension of methyl (*S*)-5-((*tert*-butoxycarbonyl)amino)-2-stearamidopentanoate (**4**) (1.70 mmol, 872 mg) in a mixture of H_2O (3.0 mL) and THF (3.0 mL) was added NaOH (15.0 mmol, 600 mg). The reaction mixture was stirred for 3 hours at room temperature. The mixture was acidified with HCl (aq. 1 M) to pH < 3 and extracted with EtOAc (3×30 mL). The combined organic layers were washed with brine (1×10 mL), dried over Na_2SO_4, filtered and the volatiles evaporated *in vacuo*. The residue was azeotropically evaporated with $CHCl_3$ (3×30 mL) to give the anhydrous product **5**. Yield: 763 mg (1.53 mmol, 90 %) of white solid; m.p. = 82.0–84.3 °C. EI-HRMS: *m/z* = 499.4113 (MH^+); $C_{28}H_{55}N_2O_5$ requires: *m/z* = 499.4105 (MH^+); ν_{max} 3359, 2955, 2916, 2849, 1738, 1682, 1605, 1525, 1465, 1454, 1388, 1365, 1290, 1274, 1244, 1210, 1170, 1112, 1043, 1019, 957, 890, 860, 783, 727, 637 cm^{-1}. ^{1}H-NMR (500 MHz, $CDCl_3$): δ 0.88 (*t*, *J*=6.9 Hz, 3H), 1.17 – 1.36 (*m*, 28H), 1.44 (*s*, 9H), 1.52 – 1.67 (*m*, 4H), 1.68 – 1.79 (*m*, 1H), 1.87 – 1.98 (*m*, 1H), 2.25 (*t*, *J*=7.8 Hz, 2H), 3.04 – 3.27 (*m*, 2H), 4.60 (*td*, *J*=4.9, 7.5 Hz, 1H), 4.86 (*t*, *J*=6.4 Hz, 1H), 6.73 (br *d*, *J*=7.4 Hz, 1H), 9.64 (br *s*, 1H). ^{13}C-NMR (126 MHz, $CDCl_3$): δ 14.27, 22.84, 25.79, 26.56, 28.52, 29.06, 29.42, 29.49, 29.51, 29.67, 29.80, 29.83, 29.86, 32.07, 36.57, 39.88, 52.22, 79.99, 156.91, 174.55, 174.80 (5 signals missing due to overlapping). ^{1}H-NMR (500 MHz, DMSO-d_6): δ 0.85 (*t*, *J*=6.9 Hz, 3H), 1.06 – 1.31 (*m*, 29H), 1.37 (*s*, 9H), 1.32 – 1.56 (*m*, 4H), 1.61 – 1.71 (*m*, 1H), 2.04 – 2.15 (*m*, 2H), 2.84 – 2.94 (*m*, 2H), 4.13 (*td*, *J*=5.0, 8.5 Hz, 1H), 6.77 (*t*, *J*=5.8 Hz, 1H), 7.98 (*d*, *J*=7.8 Hz, 1H), 12.40 (br *s*, 1H). ^{13}C-NMR (126 MHz, DMSO-d_6): δ 13.91, 22.06, 25.24, 26.15, 28.23, 28.43, 28.57, 28.66, 28.77, 28.93, 28.97, 29.00, 31.26, 35.02, 51.54, 77.33, 155.54, 172.23, 173.71 (7 signals missing due to overlapping).

Synthesis of methyl (*S*)-7-((*tert*-butoxycarbonyl)amino)-3-oxo-4-stearamidoheptanoate (2h)

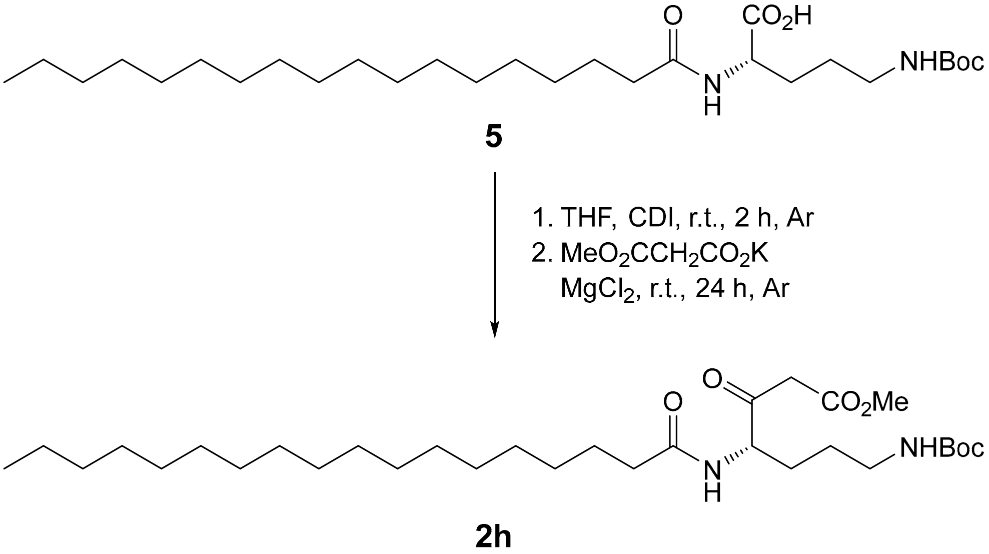

Following *GP1*, prepared from (*S*)-5-((*tert*-butoxycarbonyl)amino)-2-stearamidopentanoic acid (**5**) (1.5 mmol, 748 mg), CDI (1.8 mmol, ω = 0.97, 301 mg), $MgCl_2$ (1.47 mmol, ω = 0.98, 143 mg), methyl potassium malonate (2.25 mmol, 351 mg); isolation by extraction and column chromatography (EtOAc/petroleum ether = 1:1). Yield: 591 mg (1.065 mmol, 71 %) of white solid; m.p. = 62.3–64.9 °C. EI-HRMS: *m/z* = 555.4384 (MH^+); $C_{31}H_{59}N_2O_6$ requires: *m/z* = 555.4368 (MH^+); ν_{max} 3341, 2915, 2848, 1747, 1711, 1681, 1638, 1524, 1438, 1390, 1365, 1316, 1251, 1168, 1016, 886, 769, 719, 643 cm^{-1}. ^{1}H-NMR (500 MHz, $CDCl_3$): δ 0.88 (*t*, *J*=6.9 Hz, 3H), 1.18 – 1.35 (*m*, 28H), 1.44 (*s*, 9H), 1.47 – 1.68 (*m*, 5H), 1.90 – 2.00 (*m*, 1H), 2.21 – 2.26 (*m*, 2H), 3.15 (*q*, *J*=6.7 Hz, 2H), 3.58 (*s*, 2H), 3.74 (*s*, 3H), 4.66 (br *s*, 1H), 4.68 – 4.74 (*m*, 1H), 6.43 (br *d*, *J*=7.4 Hz, 1H). ^{13}C-NMR (126 MHz, $CDCl_3$): δ 14.27, 22.83, 25.73, 26.44, 27.58, 28.52, 29.44, 29.47, 29.50, 29.63, 29.77, 29.80, 29.84, 32.06, 36.62, 39.87, 46.26, 52.66, 58.11, 79.56, 156.40, 167.45, 173.54, 201.86 (6 signals missing due to overlapping). ^{1}H-NMR (500 MHz, DMSO-d_6): δ 0.85 (*t*, *J*=6.9 Hz, 3H), 1.08 – 1.30 (*m*, 29H), 1.37 (*s*, 9H), 1.31 – 1.54 (*m*, 4H), 1.62 – 1.75 (*m*, 1H), 2.12 (*t*, *J*=7.4 Hz, 2H), 2.89 (*q*, *J*=6.2 Hz, 2H), 3.58 (*s*, 2H), 3.61 (*s*, 3H), 4.23 (*ddd*, *J*=4.5, 7.3, 9.5 Hz, 1H), 6.78 (*t*, *J*=5.9 Hz, 1H), 8.16 (*d*, *J*=7.4 Hz, 1H). ^{13}C-NMR (126 MHz, DMSO-d_6): δ 13.90, 22.05, 25.10, 25.88, 26.25, 28.22, 28.57, 28.65, 28.71, 28.89, 28.96, 28.99, 31.25, 34.91, 45.49, 51.76, 57.75, 77.36, 155.57, 167.46, 172.67, 202.73 (7 signals missing due to overlapping).

Synthesis of (*S*)-5-methoxy-5-oxo-4-stearamidopentan-1-aminium 2,2,2-trifluoroacetate (6)

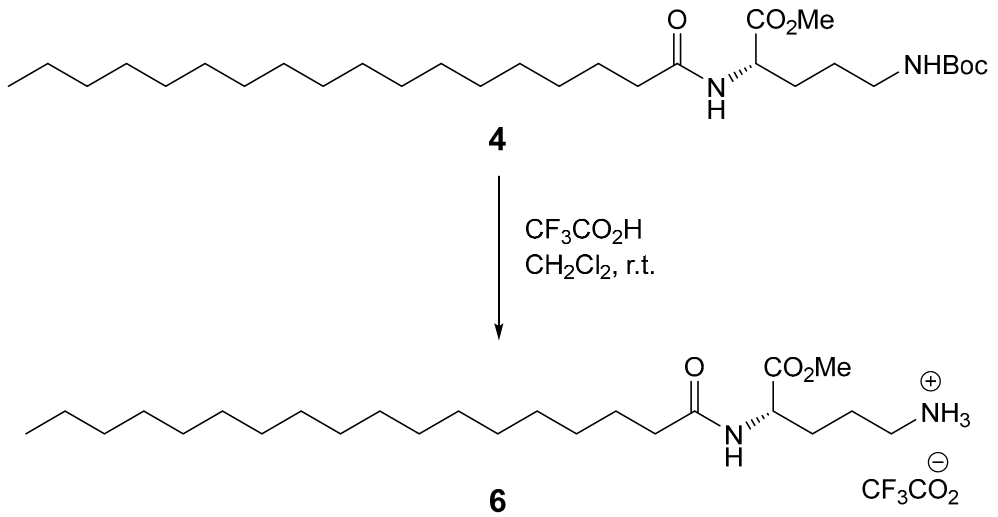

Methyl (*S*)-5-((*tert*-butoxycarbonyl)amino)-2-stearamidopentanoate (**4**) (10 mmol, 5.128 g) was dissolved in a 1:1 mixture of CF_3COOH and anhydrous CH_2Cl_2 (60 mL) under argon, and the reaction mixture was stirred for 3 hours at room temperature. Volatile components were evaporated *in vacuo*, and the residue was azeotropically evaporated with anhydrous toluene (3×100 mL) to give ammonium salt **6**. Yield: 5.00 g (9.50 mmol, 95%) of white solid; m.p. = 93.0–95.7 °C. EI-HRMS: m/z = 413.3726 (MH^+); $C_{24}H_{49}N_2O_3^+$ requires: m/z = 413.3738 (MH^+); ν_{max} 3318, 2915, 2848, 1752, 1671, 1645, 1528, 1474, 1462, 1430, 1400, 1381, 1358, 1276, 1237, 1207, 1173, 1127, 1067, 1003, 970, 955, 893, 839, 800, 768, 747, 723, 668, 613 cm^{-1}. ^{1}H-NMR (500 MHz, $CDCl_3$ (700 μL) + TFA (20 μL)): δ 0.88 (*t*, *J*=6.9 Hz, 3H), 1.09 – 1.35 (*m*, 26H), 1.53 – 1.63 (*m*, 2H), 1.69 – 1.85 (*m*, 3H), 1.91 – 2.01 (*m*, 1H), 2.23 – 2.33 (*m*, 2H), 3.01 – 3.20 (*m*, 2H), 3.76 (*s*, 3H), 4.48 – 4.57 (*m*, 1H), 6.86 (*d*, *J*=7.5 Hz, 1H), 7.61 (br *s*, 3H). ^{13}C-NMR (126 MHz, $CDCl_3$ (700 μL) + TFA (20 μL)): δ 14.25, 22.84, 23.37, 25.78, 29.22, 29.28, 29.31, 29.51, 29.59, 29.74, 29.80, 29.81, 29.86, 32.07, 36.22, 39.62, 51.65, 53.08, 115.46 (*q*, *J*=287.8 Hz), 161.08 (*q*, *J*=39.3 Hz), 172.10, 176.30 (3 signals missing due to overlapping).

Synthesis of methyl (*S*)-2,5-distearamidopentanoate (7)

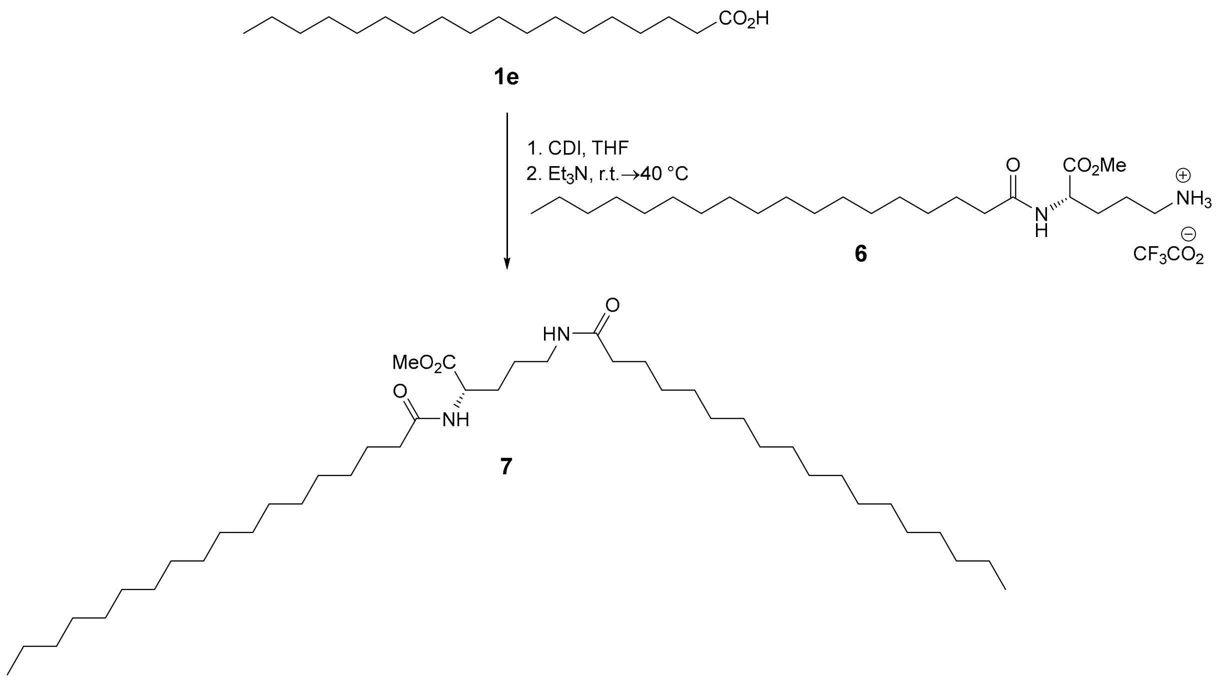

To a solution of stearic acid (**1e**) (8.5 mmol, 2.416 g) in anhydrous THF (30 mL), 1,1'-carbonyldiimidazole (CDI; 9.35 mmol, ω = 0.97, 1.563 g) was added under argon, and the resulting reaction mixture was stirred for 2 h at room temperature. The resulting activated acid was transferred to a suspension of (*S*)-5-methoxy-5-oxo-4-stearamidopentan-1-aminium 2,2,2-trifluoroacetate (**6**) (9.35 mmol, 4.924 g) in anhydrous THF (30 mL) under argon. Et_3N (9.35 mmol, 1.303 mL) was then added to the reaction mixture at room temperature. The reaction mixture was stirred at 40 °C for 12 hours. Volatile components were evaporated *in vacuo*. The residue was extracted with CH_2Cl_2 (100 mL), EtOAc (100 mL), Et_2O (100 mL), and *n*-hexane (100 mL) using a laboratory ultrasonic bath (5 minutes each), followed by decanting, respectively, to remove unreacted starting material and small portions of the product. H_2O (150 mL) was added to the residue, followed by ultrasonic bath treatment (15 minutes). The resulting precipitate was collected by filtration and thoroughly washed with H_2O (3×70 mL). The residue was dried under high vacuum at 40 °C for 12 hours to give product **7**. Yield: 3.476 g (5.270 mmol, 62 %) of white solid; m.p. = 106.7–108.4 °C. EI-HRMS: *m/z* = 679.6336 (MH^+); $C_{42}H_{83}N_2O_4$ requires: *m/z* = 679.6347 (MH^+); ν_{max} 3305, 2914, 2848, 1742, 1639, 1542, 1470, 1420, 1385, 1277, 1259, 1239, 1205, 1173, 980, 717 cm^{-1}. ^{1}H-NMR (500 MHz, $CDCl_3$ (700 μL) + TFA (20 μL)): δ 0.88 (*t*, *J*=6.9 Hz, 6H), 1.18 – 1.36 (*m*, 56H), 1.54 – 1.77 (*m*, 7H), 1.87 – 1.97 (*m*, 1H), 2.26 – 2.38 (*m*, 4H), 3.23 – 3.34 (m, 1H), 3.34 – 3.47 (*m*, 1H), 3.79 (*s*, 3H), 4.57 – 4.64 (*m*, 1H), 6.70 (*t*, *J*=6.1 Hz, 1H), 6.79 (*d*, *J*=7.7 Hz, 1H). ^{13}C-NMR (126 MHz, $CDCl_3$ (700 μL) + TFA (20 μL)): δ 14.25, 22.84, 24.97, 25.87, 25.99, 29.20, 29.23, 29.28, 29.51, 29.56,

29.72, 29.78, 29.81, 29.83, 29.85, 30.00, 32.07, 36.28, 36.35, 39.58, 52.17, 53.15, 172.43, 176.50, 177.11 (17 signals missing due to overlapping).

Synthesis of (*S*)-2,5-distearamidopentanoic acid (8)

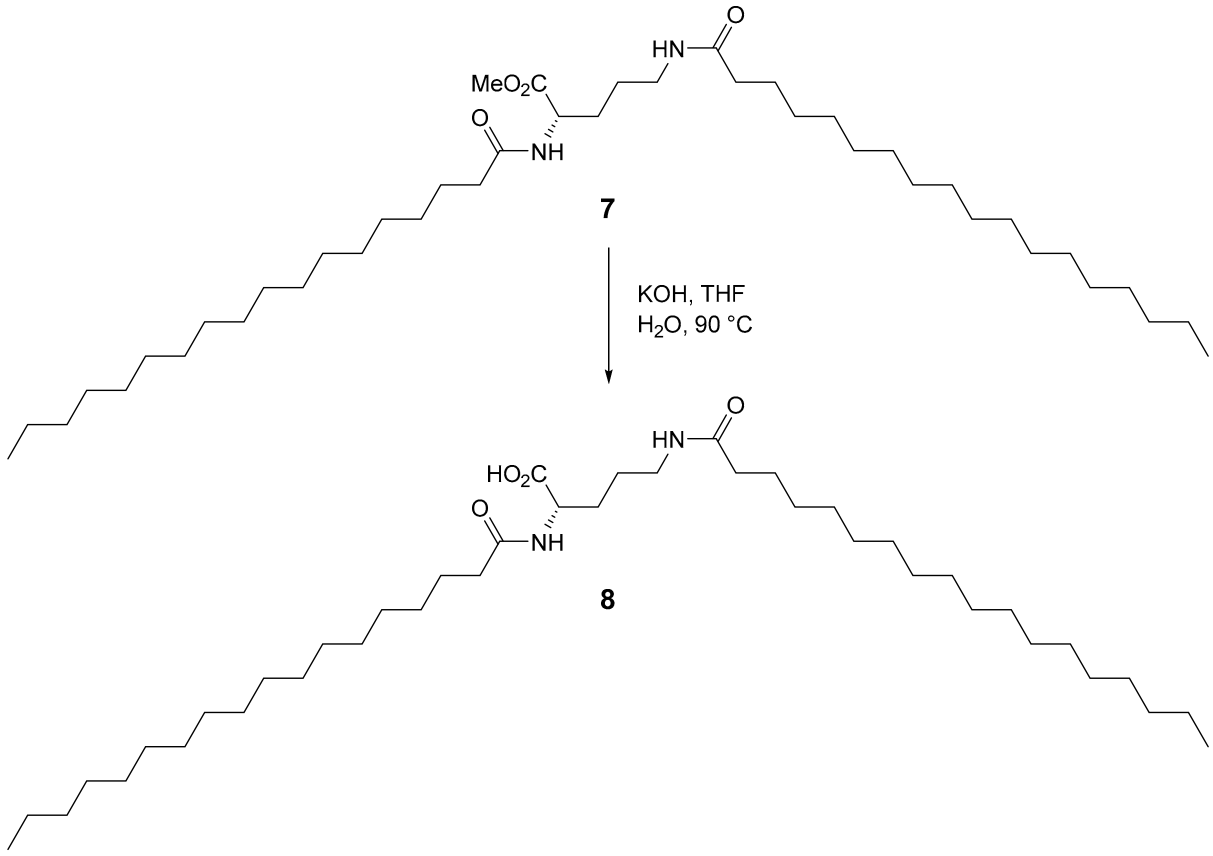

To a suspension of methyl (*S*)-2,5-distearamidopentanoate (**7**) (5 mmol, 3.393 g) in a mixture of H_2O (20 mL) and THF (5 mL), KOH (powder for synthesis, 50 mmol, 2.810 g) was added, and the reaction mixture was stirred at 90 °C for 12 hours. The mixture was cooled to room temperature, and HCl (aq., 2 M) was added under stirring until the pH reached 1–2. The precipitate was collected by filtration and thoroughly washed with H_2O (3×100 mL). The residue was dried under high vacuum at 40 °C for 12 hours to give acid **8**. Yield: 2.792 g (4.20 mmol, 84 %) of white solid; m.p. = 108.8–110.1 °C. EI-HRMS: *m/z* = 665.6191 (MH^+); $C_{41}H_{81}N_2O_4$ requires: *m/z* = 665.6191 (MH^+); ν_{max} 3310, 2955, 2916, 2849, 1736, 1639, 1586, 1545, 1466, 1446, 1418, 1372, 1275, 1245, 1211, 1181, 1128, 970, 829, 720, 685, 633 cm^{-1}. ^{1}H-NMR (500 MHz, $CDCl_3$ (700 µL) + TFA (20 µL)): δ 0.88 (*t*, *J*=6.8 Hz, 6H), 1.09 – 1.40 (*m*, 56H), 1.52 – 1.73 (*m*, 6H), 1.75 – 1.89 (*m*, 1H), 1.92 – 2.11 (*m*, 1H), 2.27 – 2.45 (*m*, 4H), 3.13 – 3.49 (*m*, 2H), 4.61 (*q*, *J*=6.7, 1H), 6.87 (*s*, 1H), 6.98 (*d*, *J*=7.3 Hz, 1H), 11.12 (br *s*, 1H). ^{13}C-NMR (126 MHz, $CDCl_3$ (700 µL) + TFA (20 µL)): δ 14.26, 22.84, 24.97, 25.88, 25.96, 29.14,

29.19, 29.21, 29.25, 29.27, 29.52, 29.55, 29.57, 29.73, 29.79, 29.82, 29.84, 29.86, 32.08, 36.09, 36.13, 39.82, 52.36, 175.92, 177.30, 177.72 (15 signals missing due to overlapping).

Synthesis of methyl (*S*)-3-oxo-4,7-distearamidoheptanoate (2i)

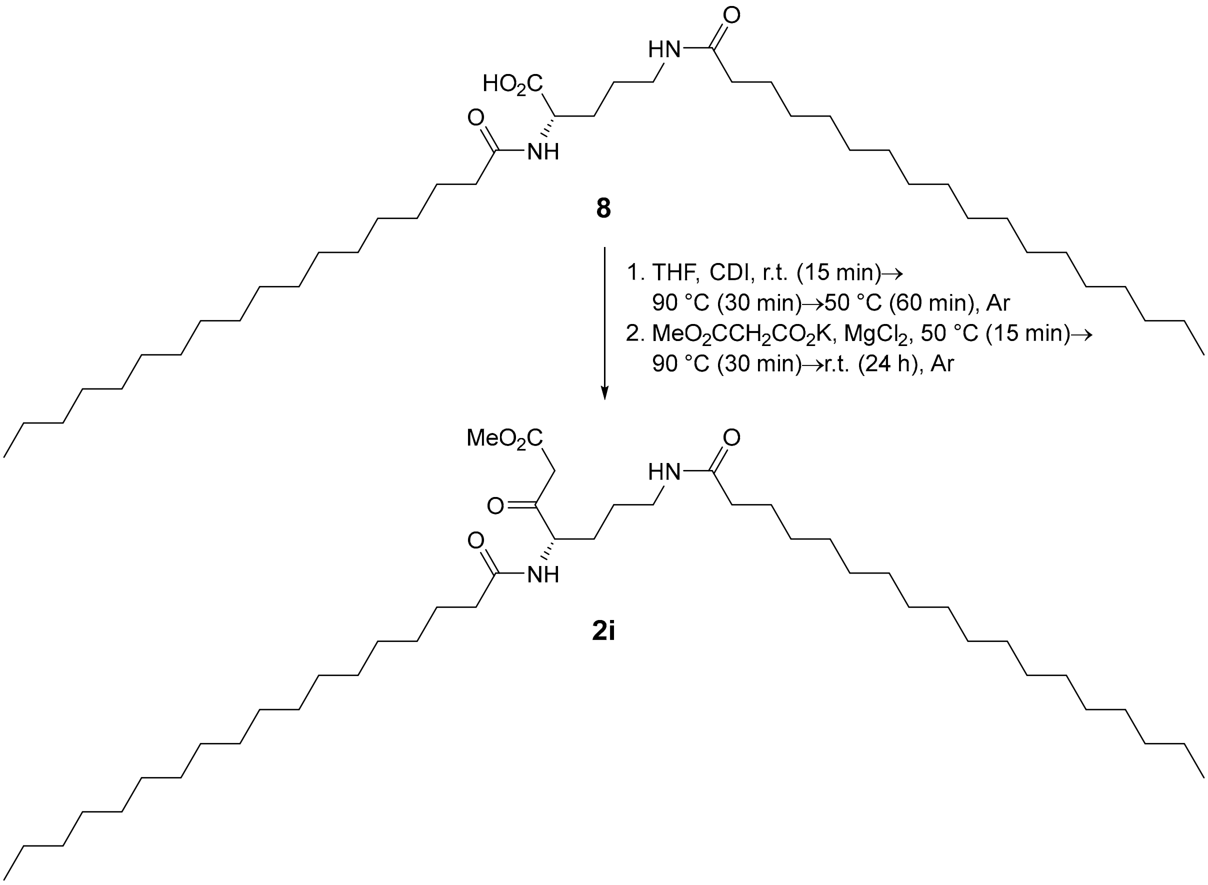

To a suspension of (*S*)-2,5-distearamidopentanoic acid (**8**) (2.5 mmol, 1.662 g) in anhydrous THF (25 mL), 1,1'-carbonyldiimidazole (CDI; 5 mmol, ω = 0.97, 836 mg) was added under argon, and the reaction mixture was stirred for 15 minutes at room temperature, 30 minutes at 90 °C, and 60 minutes at 50 °C. Then, a solid mixture of $MgCl_2$ (2.5 mmol, ω = 0.98, 243 mg) and methyl potassium malonate (7.5 mmol, 1.171 g) was carefully added at 50 °C. The reaction mixture was stirred for 15 minutes at 50 °C, 30 minutes at 90 °C, and 24 hours at room temperature. The volatiles were evaporated *in vacuo*, and $NaHSO_4$ (1 M in H_2O, 100 mL) was added to the residue. The mixture was stirred at room temperature for 30 minutes. The precipitate was collected by filtration and thoroughly washed with H_2O (3×100 mL). The residue was dried under high vacuum at 40 °C for 12 hours to give β-keto ester **2i**. Yield: 1.099 g (1.525 mmol, 61 %) of white solid; m.p. = 92.1–94.7 °C. EI-HRMS: *m/z* = 721.6456 (MH^+); $C_{44}H_{85}N_2O_5$ requires: *m/z* = 721.6453 (MH^+); ν_{max} 3306, 2916, 2849, 1748, 1717, 1638, 1539, 1463, 1378, 1328, 1258, 1239, 1223, 1204, 1147, 1013, 719 cm^{-1}. ^{1}H-NMR (600 MHz, $CDCl_3$):

δ 0.88 (*t*, *J*=6.9 Hz, 6H), 1.18 – 1.39 (*m*, 56H), 1.51 – 1.67 (*m*, 7H), 1.88 – 1.97 (*m*, 1H), 2.17 (*t*, *J*=7.7 Hz, 2H), 2.25 (*t*, *J*=7.7 Hz, 2H), 3.22 – 3.38 (*m*, 2H), 3.58 (*d*, *J*=1.7 Hz, 2H), 3.74 (*s*, 3H), 4.65 – 4.72 (*m*, 1H), 5.87 (*t*, *J*=6.0 Hz, 1H), 6.67 (*d*, *J*=7.5 Hz, 1H). ^{13}C-NMR (151 MHz, $CDCl_3$): δ 14.26, 22.83, 25.72, 25.94, 25.98, 27.70, 29.45, 29.50, 29.52, 29.66, 29.78, 29.80, 29.81, 29.85, 32.07, 36.56, 36.96, 38.86, 46.22, 52.66, 58.21, 167.58, 173.81, 173.95, 201.90 (19 signals missing due to overlapping).

Synthesis of pyrrolones 11 from β-keto esters 10 – ***General procedure 2 (GP2)***

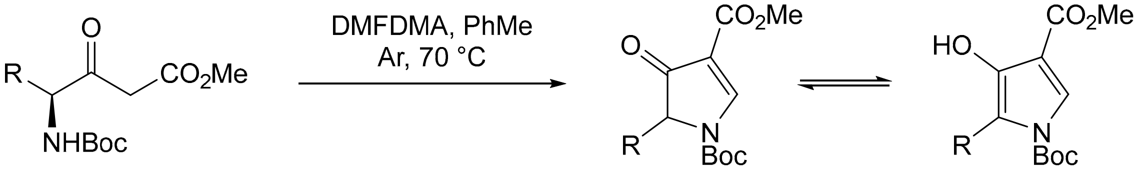

10 **11** **11'**

To a solution of β-keto ester **10** (1.0 mmol) in anhydrous toluene (5 mL), DMFDMA (3 mmol, ω = 0.94, 424 μL) was added under argon and the resulting reaction mixture was stirred at 70 °C under argon until completion of the reaction, as judged by TLC analysis (1–3 hours). The volatiles were evaporated *in vacuo* and the residue was purified as quickly as possible by column chromatography (CC, Silica gel 60). The fractions containing the product **11** were combined and the volatiles were evaporated *in vacuo*. The product was immediately used for the following transformation or/and stored under argon at –20 °C.

Synthesis of 1-(*tert*-butyl) 3-methyl 5-benzyl-4-oxo-4,5-dihydro-1*H*-pyrrole-1,3-dicarboxylate (11a) and 1-(*tert*-butyl) 3-methyl 5-benzyl-4-hydroxy-1*H*-pyrrole-1,3-dicarboxylate (11a')[8]

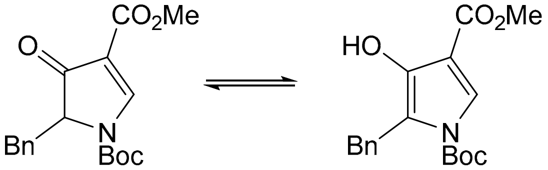

11a **11a'**

Following *GP2*. Prepared from methyl (*S*)-4-((*tert*-butoxycarbonyl)amino)-3-oxo-5-phenylpentanoate (**10a**) (1 mmol, 321.4 mg), 45 minutes; CC (EtOAc/petroleum ether = 1:1). **11a**/**11a'** = 45:55 (in DMSO-d_6). Yield: 268 mg (0.81 mmol, 81 %) of colorless oil. ^{1}H-NMR (500 MHz, DMSO-d_6) for **11a**: δ 1.55 (*s*, 9H), 3.22 (*dd*, *J*=2.7, 13.8 Hz, 1H), 3.38 (*dd*, *J*=6.4, 13.8 Hz, 1H), 3.61 (*s*, 3H), 4.58 (*dd*, *J*=2.6, 6.3 Hz, 1H), 6.91 – 6.97 (*m*, 2H), 8.71 (*s*, 1H). ^{1}H-

NMR (500 MHz, DMSO-d_6) for **11a'**: *δ* 1.34 (*s*, 9H), 3.76 (*s*, 3H), 4.14 (*s*, 2H), 6.98 – 7.04 (*m*, 2H), 7.11 – 7.28 (*m*, 3H), 7.57 (*s*, 1H), 8.26 (*s*, 1H).

Synthesis of 1-(*tert*-butyl) 3-methyl 5-(4-(((benzyloxy)carbonyl)amino)butyl)-4-oxo-4,5-dihydro-1*H*-pyrrole-1,3-dicarboxylate (11b) and 1-(*tert*-butyl) 3-methyl 5-(4-(((benzyloxy)carbonyl)amino)butyl)-4-hydroxy-1*H*-pyrrole-1,3-dicarboxylate (11b')[8]

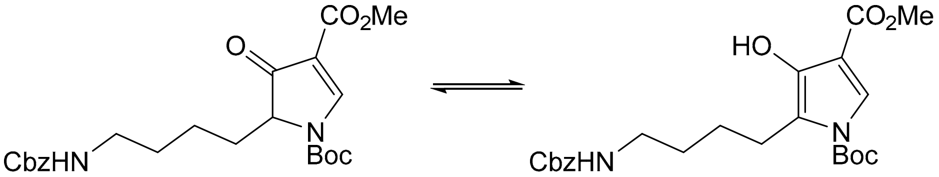

11b **11b'**

Following *GP2*. Prepared from methyl (*S*)-8-(((benzyloxy)carbonyl)amino)-4-((*tert*-butoxycarbonyl)amino)-3-oxooctanoate (**10b**) (1 mmol, 436.5 mg), 1 hour; CC (EtOAc/petroleum ether = 1:1). **11b**/**11b'** = 37:63 (in DMSO-d_6). Yield: 336 mg (0.82 mmol, 82 %) of colorless oil. ^{1}H-NMR (500 MHz, DMSO-d_6) for **11b**: *δ* 0.98 – 1.08 (*m*, 1H), 1.08 – 1.19 (*m*, 1H), 1.50 (*s*, 9H), 1.83 – 1.93 (*m*, 1H), 1.97 – 2.08 (*m*, 1H), 2.93 (*q*, *J*=6.7 Hz, 2H), 3.68 (*s*, 3H), 4.30 (*dd*, *J*=3.1, 6.5 Hz, 1H), 8.98 (*s*, 1H). ^{1}H-NMR (500 MHz, DMSO-d_6) for **11b'**: *δ* 1.28 – 1.46 (*m*, 4H), 1.54 (*s*, 9H), 2.70 (*t*, *J*=6.9 Hz, 2H), 2.98 (*q*, *J*=6.3 Hz, 2H), 3.73 (*s*, 3H), 4.99 (*s*, 2H), 7.23 (*t*, *J*=5.8 Hz, 1H), 7.26 – 7.41 (*m*, 5H), 7.49 (*s*, 1H), 7.95 (*s*, 1H).

Synthesis of 1-(*tert*-butyl) 3-methyl 5-(3-(benzyloxy)-3-oxopropyl)-4-oxo-4,5-dihydro-1*H*-pyrrole-1,3-dicarboxylate (11c) and 1-(*tert*-butyl) 3-methyl 5-(3-(benzyloxy)-3-oxopropyl)-4-hydroxy-1*H*-pyrrole-1,3-dicarboxylate (11c')[12]

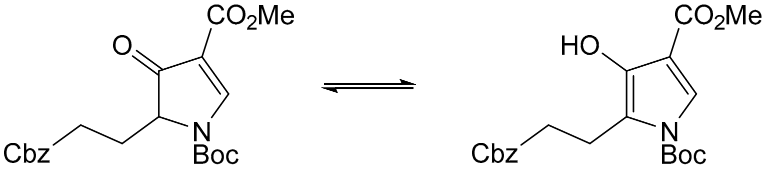

11c **11c'**

Following *GP2*. Prepared from 7-benzyl 1-methyl (*S*)-4-((*tert*-butoxycarbonyl)amino)-3-oxoheptanedioate (**10c**) (1 mmol, 393.4 mg), 45 minutes; CC (EtOAc/petroleum ether = 1:1). **11c**/**11c'** = 42:58 (in DMSO-d_6). Yield: 343 mg (0.85 mmol, 85 %) of colorless oil. ^{1}H-NMR (500 MHz, DMSO-d_6) for **11c**: *δ* 1.50 (*s*, 9H), 3.68 (*s*, 3H), 4.35 – 4.40 (*m*, 1H), 5.06 (*d*, *J*=5.4 Hz, 2H), 8.92 (*s*, 1H). ^{1}H-NMR (500 MHz, DMSO-d_6) for **11c'**: *δ* 1.53 (*s*, 9H), 2.51 – 2.57 (*m*,

2H), 2.99 – 3.05 (*m*, 2H), 3.73 (*s*, 3H), 5.08 (*s*, 2H), 7.30 – 7.40 (*m*, 5H), 7.49 (*s*, 1H), 8.11 (*s*, 1H).

Synthesis of hexadecan-1-ol (13)[13]

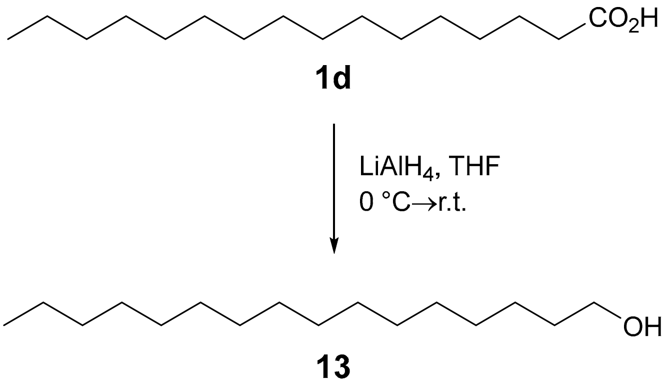

Palmitic acid (**11d**) (20 mmol, 5.128 g) was dissolved in anhydrous THF (80 mL) under argon and the solution was cooled in an ice bath (0 ºC). While stirring in the ice bath, $LiAlH_4$ (2.4 M in THF, 80 mmol, 33.3 mL) was added and the reaction mixture was allowed to warm to room temperature over 1 hour. The reaction mixture was stirred for a further 24 hours under argon at room temperature, and then quenched by careful addition of NaOH (1 M in H_2O, 60 mL). The reaction mixture was extracted with diethyl ether (2×70 mL). The organic phase was dried over anhydrous Na_2SO_4, filtered, and the volatile components evaporated *in vacuo*. Yield: 3.957 g (16.3 mmol, 81 %) of a white solid. ^{1}H-NMR (500 MHz, $CDCl_3$): δ 0.88 (*t*, *J*=6.9 Hz, 3H), 1.19 – 1.39 (*m*, 27H), 1.52 – 1.61 (*m*, 2H), 3.64 (*t*, *J*=6.6 Hz, 2H).

Synthesis of palmitaldehyde (14)[14]

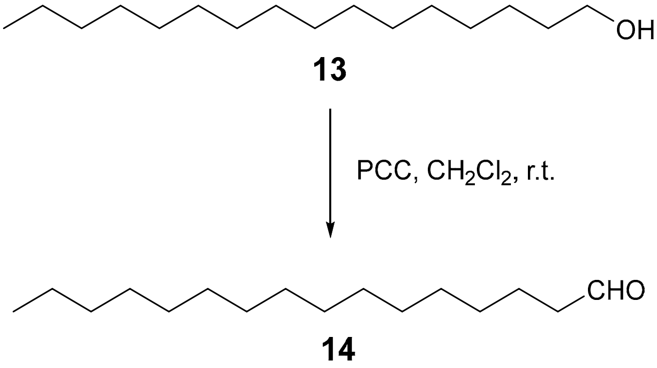

To a solution of hexadecan-1-ol (**13**) (16.3 mmol, 3.951 g) in anhydrous CH_2Cl_2 (100 mL), pyridinium chlorochromate (PCC, 24.5 mmol, ω = 0.98, 5.389 g) was added at room temperature and the reaction mixture was stirred for 16 h at room temperature. The solution was filtered through a plaque of Celite®, washed with CH_2Cl_2 and the volatiles evaporated *in*

vacuo. The residue was purified by column chromatography (Silica gel 60, petroleum ether/ethyl acetate = 10:1). The fractions containing the pure product **14** were combined and the volatile components were evaporated *in vacuo*. Yield: 2.940 g (12.23 mmol, 75 %) of a colorless oil. ^{1}H-NMR (500 MHz, $CDCl_3$): δ 0.88 (*t*, *J*=6.9 Hz, 3H), 1.26 (s, 24H), 1.58 – 1.67 (*m*, 2H), 2.42 (*td*, *J*=1.9, 7.4 Hz, 2H), 9.76 (*t*, *J*=1.9 Hz, 1H).

Synthesis of 1-nitroheptadecan-2-ol (15)

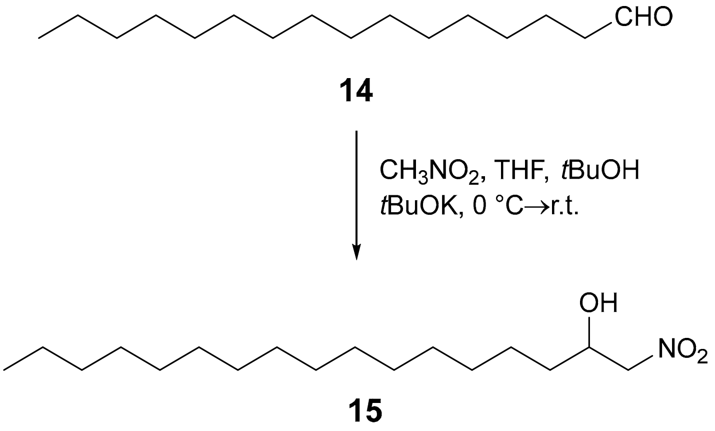

Prepared according to the literature procedure.[15] Palmitaldehyde (**14**) (11.44 mmol, 2.751 g) was dissolved in a mixture of anhydrous THF and anhydrous *t*-butanol in a 1:1 ratio (50 mL) under argon. Nitromethane (17.16 mmol, 930 µL) was then added at room temperature. The mixture was cooled to 0 °C, *t*BuOK (1.144 mmol, 128 mg) was added, and the reaction mixture was allowed to warm to room temperature over 1 hour. After 16 h at room temperature under argon, the reaction mixture was diluted with H_2O (300 mL) and the product was extracted with diethyl ether (2×100 mL). The organic phase was washed with NaCl (aq. sat., 2×50 mL), dried over anhydrous Na_2SO_4, filtered, and the volatiles evaporated *in vacuo*. The crude product **15** was used for the following transformation without further purification. Yield: 2.794 g (9.267 mmol, 81 %) of a yellowish oil. ^{1}H-NMR (500 MHz, $CDCl_3$): δ 0.88 (*t*, *J*=6.9 Hz, 3H), 1.18 – 1.42 (*m*, 26H), 1.44 – 1.57 (*m*, 2H), 2.61 (br *s*, 1H), 4.28 – 4.34 (*m*, 1H), 4.38 (*dd*, *J*=8.5, 13.0 Hz, 1H), 4.43 (*dd*, *J*=2.7, 13.0 Hz, 1H).

Synthesis of (*E*)-1-nitroheptadec-1-ene (16)[16]

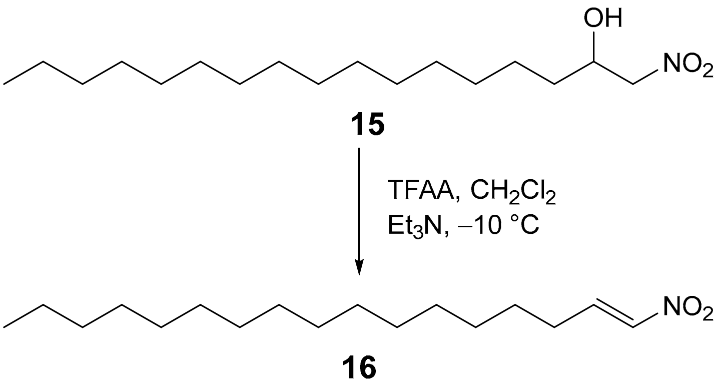

Prepared according to the literature procedure.[15] 1-Nitroheptadecan-2-ol (**15**) (9.12 mmol, 2.75 g) was dissolved in anhydrous CH_2Cl_2 (25 mL) and cooled to –10 °C. With stirring, trifluoroacetic anhydride (TFAA, 9.12 mmol, 1.269 mL) was added dropwise and the cooled mixture (–10 °C) was stirred for another 2 minutes. Over the next 10 minutes, triethylamine (2.532 mL, 18.24 mmol) was added dropwise and the reaction mixture was stirred at –10 °C for another 30 minutes. The reaction mixture was then diluted with CH_2Cl_2 (100 mL) and washed with $NaHSO_4$ (aq., 1 M, 200 mL). The aqueous phase was extracted with CH_2Cl_2 (2×40 mL). The combined organic phase was dried over anhydrous Na_2SO_4, filtered, and the volatiles evaporated *in vacuo*. The residue was purified by column chromatography (Silica gel 60; petroleum ether/EtOAc = 40:1). The fractions containing the pure product **16** were combined and the volatile components were evaporated *in vacuo*. Product **16** was stored under argon at 5 °C. Yield: 2.016 g (7.114 mmol, 78 %) of a white solid; m.p. = 25.0–25.7 °C. EI-HRMS: *m/z* = 306.2395 (MNa^+); $C_{17}H_{34}NNaO_2$ requires: *m/z* = 306.2404 (MNa^+); ν_{max} 2922, 2853, 1650, 1526, 1465, 1350, 960, 835, 723 cm^{-1}. ^{1}H-NMR (500 MHz, $CDCl_3$): δ 0.88 (*t*, *J*=6.9 Hz, 3H), 1.19 – 1.37 (*m*, 24H), 1.51 (*p*, *J*=7.3 Hz, 2H), 2.27 (*qd*, *J*=1.5, 7.4 Hz, 2H), 6.98 (*dt*, *J*=1.6, 13.4 Hz, 1H), 7.23 – 7.33 (*m*, 1H). ^{13}C-NMR (126 MHz, $CDCl_3$): δ 14.27, 22.84, 27.86, 28.61, 29.24, 29.40, 29.51, 29.59, 29.72, 29.77, 29.80, 29.82, 29.84, 32.07, 139.69, 143.01 (1 signal missing due to overlapping).

Organocatalyzed Michael addition of β-keto esters 2 to nitroalkenes – General procedure for the preparation of racemic products *rac*-17 – *General procedure 3 (GP3)*

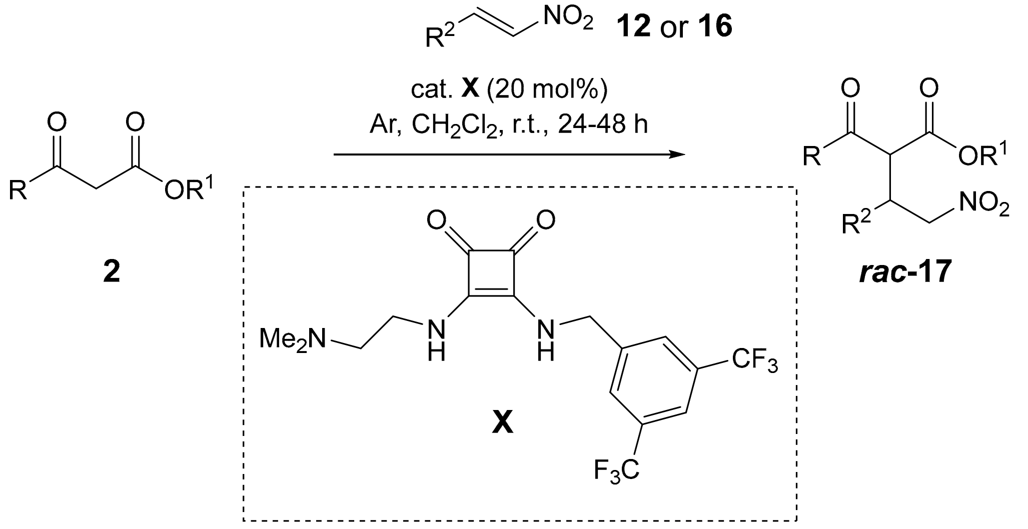

To a solution/suspension of nitroalkene **12** or **16** (0.2 mmol, 1.0 equivalent or 0.3 mmol, 1.5 equivalents) and the achiral organocatalyst **X** (0.04 mmol, 0.2 equivalents, 16.4 mg) in anhydrous CH_2Cl_2 (1 mL) under argon at room temperature, β-keto ester **2** (0.3 mmol, 1.5 equivalents or 0.2 mmol, 1.0 equivalent) was added and the resulting reaction mixture was stirred at room temperature for 24–72 hours. The volatiles were evaporated *in vacuo* and the residue was purified by column chromatography (Silica gel 60, mobile phase). The fractions containing the pure racemic product ***rac*-17** were combined and the volatiles were evaporated *in vacuo*. The product ***rac*-17** was fully characterized and analyzed by HPLC.

Organocatalyzed Michael addition of β-keto esters 2 to nitroalkenes – General procedure for the organocatalyzed asymmetric addition – ***General procedure 4 (GP4)***

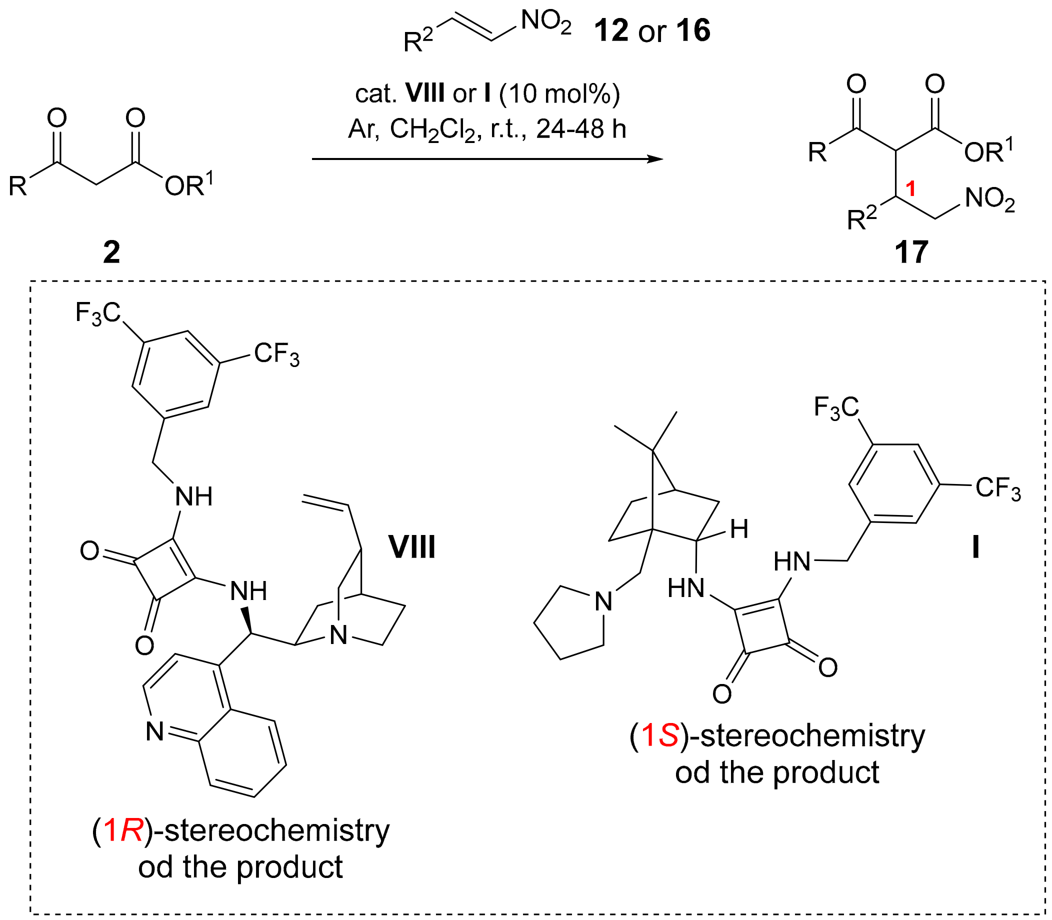

To a solution/suspension of nitroalkene **12** or **16** (0.2 mmol, 1.0 equivalent or 0.3 mmol, 1.5 equivalents) and the chiral organocatalyst **VIII** (0.02 mmol, 0.1 equivalents, 12.3 mg) or **I** (0.02 mmol, 0.1 equivalents, 10.9 mg) in anhydrous CH_2Cl_2 (1 mL) under argon at room temperature, β-keto ester **2** (0.3 mmol, 1.5 equivalents or 0.2 mmol, 1.0 equivalent) was added and the resulting reaction mixture was stirred at room temperature for 24–72 hours. The volatiles were evaporated *in vacuo* and the residue was purified by column chromatography (Silica gel 60, mobile phase). The fractions containing the pure chiral nonracemic product **17** were combined the volatiles were evaporated *in vacuo*. The product **17** was fully characterized and analyzed by HPLC.

Synthesis of methyl 4-((*tert*-butoxycarbonyl)amino)-2-((*R*)-2-nitro-1-phenylethyl)-3-oxobutanoate (17a)

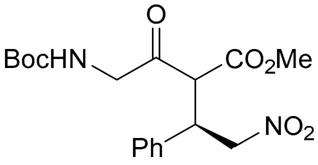

Following *GP3* and *GP4*. Prepared from methyl 4-((*tert*-butoxycarbonyl)amino)-3-oxobutanoate (**2a**) (0.2 mmol, 46.3 mg) and *trans*-β-nitrostyrene (**12**) (0.3 mmol, 44.7 mg), organocatalyst **VIII**, 24 h; isolation by column chromatography (EtOAc/petroleum ether = 1:4). ***rac*-17a** Yield: 41.8 mg (0.110 mmol, 55%, two diastereomers in a ratio of 59:41 in DMSO-d_6) of white solid. **17a** Yield: 63.9 mg (0.168 mmol, 84%, two diastereomers in a ratio of 56:44 in DMSO-d_6) of white solid; m.p. = 116–122 °C. EI-HRMS: *m/z* = 381.1641 (MH^+); $C_{18}H_{25}N_2O_7$ requires: *m/z* = 381.1656 (MH^+); ν_{max} 3378, 2981, 1751, 1714, 1555, 1497, 1455, 1429, 1367, 1252, 1154, 1020, 974, 895, 858, 764, 699, 637 cm^{-1}. ^{1}H-NMR (500 MHz, DMSO-d_6) for major diastereomer: δ 1.39 (*s*, 9H), 3.37 (s, 3H), 3.95 (*dd*, *J*=3.5, 5.8 Hz, 2H), 4.06 – 4.13 (*m*, 1H), 4.40 (*d*, *J*=10.7 Hz, 1H), 4.87 – 4.96 (*m*, 2H), 7.18 (*t*, *J*=5.9 Hz, 1H), 7.22 – 7.34 (*m*, 5H). ^{1}H-NMR (500 MHz, DMSO-d_6) for minor diastereomer: δ 1.33 (*s*, 9H), 3.70 (*s*, 3H), 3.50 (*dd*, *J*=5.9, 18.8 Hz, 1H), 3.80 (*dd*, *J*=5.8, 18.8 Hz, 1H), 4.46 (*d*, *J*=10.2 Hz, 1H), 4.75 (*dd*, *J*=4.3, 13.2 Hz, 1H), 4.85 (*d*, *J*=10.8 Hz, 1H), 7.06 (*t*, *J*=5.8 Hz, 1H). ^{13}C-NMR (126 MHz, DMSO-d_6) for both diastereomers: δ 28.10, 28.14, 42.35, 42.65, 50.18, 50.25, 52.45, 52.84, 57.28, 57.93, 59.78, 77.74, 78.22, 78.24, 78.44, 127.79, 127.85, 128.18, 128.25, 128.49, 128.64, 136.85, 136.92, 155.47, 155.81, 167.33, 170.36, 199.88, 200.62 (3 signals missing due to overlapping). HPLC: Chiralpak IA-3, *n*-Hexane/*i*-PrOH = 80:20, flow rate 1.0 mL/min, λ = 210 nm, T = 20°C. Diastereomer 1: *t*R = 9.24 minutes (minor); 17.62 minutes (major) – 91% *ee*. Diastereomer 2: *t*R = 14.12 minutes (major); 23.56 minutes (minor) – 93% *ee*.

Synthesis of methyl 5-((*tert*-butoxycarbonyl)amino)-2-((*R*)-2-nitro-1-phenylethyl)-3-oxopentanoate (17b)[1]

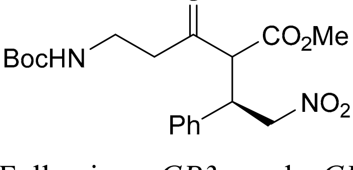

Following *GP3* and *GP4*. Prepared from methyl 5-((*tert*-butoxycarbonyl)amino)-3-oxopentanoate (**2b**) (0.2 mmol, 49.1 mg) and *trans*-β-nitrostyrene (**12**) (0.3 mmol, 44.7 mg), organocatalyst **VIII**, 24 h; isolation by column chromatography (EtOAc/petroleum ether = 1:4).

***rac*-17b** Yield: 69.4 mg (0.176 mmol, 88%, two diastereomers in a ratio of 53:47 in DMSO-d_6) of white solid. **17b** Yield: 67.1 mg (0.170 mmol, 85%, two diastereomers in a ratio of 53:47 in DMSO-d_6) of white solid; m.p. = 96.1–98.4 °C. EI-HRMS: *m/z* = 417.1618 (MNa^+); $C_{19}H_{27}N_2NaO_7$ requires: *m/z* = 417.1632 (MNa^+); ν_{max} 3424, 2978, 1743, 1707, 1553, 1506, 1455, 1434, 1366, 1246, 1164, 1082, 966, 859, 756, 701 cm^{-1}. ^{1}H-NMR (500 MHz, DMSO-d_6) for both diastereomers: δ 1.34 (*s*, 4.5H), 1.37 (*s*, 4.5H), 2.24 – 2.34 (*m*, 0.5H), 2.58 – 2.68 (*m*, 0.5H), 2.74 (*t*, *J*=6.8 Hz, 1H), 2.76 – 2.91 (*m*, 1H), 3.15 (*q*, *J*=6.4 Hz, 1H), 3.35 (*s*, 1.5H), 3.70 (*s*, 1.5H), 4.00 – 4.09 (*m*, 1H), 4.37 (*dd*, *J*=6.0, 10.5 Hz, 1H), 4.81 (*d*, *J*=7.5 Hz, 1H), 4.87 – 4.99 (*m*, 1H), 6.58 (*t*, *J*=5.7 Hz, 0.5H), 6.85 (*t*, *J*=5.7 Hz, 0.5H), 7.21 – 7.35 (*m*, 5H). ^{13}C-NMR (126 MHz, DMSO-d_6) for both diastereomers: δ 28.19, 28.22, 34.52, 34.79, 42.30, 42.36, 42.66, 42.89, 52.43, 52.83, 60.00, 60.84, 77.69, 77.78, 78.00, 78.15, 127.79, 127.86, 128.27, 128.46, 128.63, 136.80, 136.96, 155.33, 155.53, 166.88, 167.72, 201.83 (6 signals missing due to overlapping). HPLC: Chiralpak IA-3, *n*-Hexane/*i*-PrOH = 80:20, flow rate 1.0 mL/min, λ = 210 nm, T = 20°C. Diastereomer 1: *t*R = 7.22 minutes (minor); 8.98 minutes (major) – 95% *ee*. Diastereomer 2: *t*R = 12.53 minutes (minor); 20.94 minutes (major) – 95% *ee*.

Synthesis of methyl 2-(3-((*tert*-butoxycarbonyl)amino)propanoyl)-3-(nitromethyl)octadecanoate (*rac*-17c)

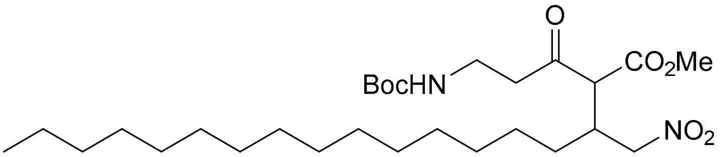

Following *GP3*. Prepared from methyl 5-((*tert*-butoxycarbonyl)amino)-3-oxopentanoate (**2b**) (0.3 mmol, 73.6 mg) and (*E*)-1-nitroheptadec-1-ene (**16**) (0.2 mmol, 56.7 mg), organocatalyst **X**, 24 h; isolation by column chromatography (EtOAc/petroleum ether = 1:5). ***rac*-17c** Yield: 55.0 mg (0.104 mmol, 52%, two diastereomers in a ratio of 53:47 in $CDCl_3$) of colorless oil. EI-HRMS: *m/z* = 429.3313 (MH^+-Boc); $C_{23}H_{45}N_2O_5$ requires: *m/z* = 429.3323 (MH^+-Boc); ν_{max} 3413, 2923, 2853, 1743, 1712, 1552, 1505, 1436, 1366, 1248, 1168, 1084, 966, 911, 863, 781, 733 cm^{-1}. ^{1}H-NMR (600 MHz, $CDCl_3$) for the major diastereomer: δ 0.88 (*t*, *J*=6.9 Hz, 3H), 1.19 – 1.40 (*m*, 28H), 1.43 (*s*, 9H), 2.71 – 2.79 (*m*, 1H), 2.82 – 2.96 (*m*, 2H), 3.30 – 3.45 (*m*, 2H), 3.76 (*s*, 3H), 3.73 – 3.83 (*m*, 1H), 4.60 (*dd*, *J*=5.0, 12.9 Hz, 1H), 4.65 (*dd*, *J*=4.5, 13.2 Hz, 1H), 4.86 – 4.96 (*m*, 1H). ^{1}H-NMR (600 MHz, $CDCl_3$) for the minor diastereomer: δ 3.76 (*s*, 3H), 4.52 (*dd*, *J*=5.9, 13.1 Hz, 2H). ^{13}C-NMR (151 MHz, $CDCl_3$) for both diastereomers: δ 14.27, 22.83, 26.82, 26.88, 28.50, 29.42, 29.43, 29.49, 29.50, 29.63, 29.65, 29.74, 29.79, 29.81,

29.83, 29.84, 30.28, 32.06, 35.17, 36.64, 36.66, 43.35, 43.56, 52.94, 53.08, 59.31, 59.76, 76.02, 76.47, 79.58, 79.63, 155.95, 168.46, 203.83 (18 signals missing due to overlapping).

Synthesis of methyl 4-((3-methylbut-2-en-1-yl)oxy)-2-((*R*)-2-nitro-1-phenylethyl)-3-oxobutanoate (17d)

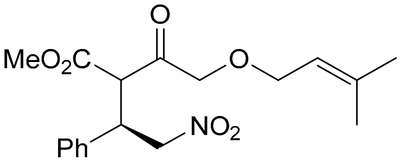

Following *GP3* and *GP4*. Prepared from methyl 4-((3-methylbut-2-en-1-yl)oxy)-3-oxobutanoate (**2c**) (0.2 mmol, 40.0 mg) and *trans*-β-nitrostyrene (**12**) (0.3 mmol, 44.7 mg), organocatalyst **VIII**, 24 h; isolation by column chromatography (EtOAc/petroleum ether = 1:5). ***rac*-17d** Yield: 54.5 mg (0.156 mmol, 78%, two diastereomers in a ratio of 60:40 in $CDCl_3$) of colorless oil. **17d** Yield: 62.2 mg (0.178 mmol, 89%, two diastereomers in a ratio of 60:40 in $CDCl_3$) of colorless oil. EI-HRMS: *m/z* = 367.1858 ($M+NH_4^+$); $C_{18}H_{27}N_2O_6$ requires: *m/z* = 367.1864 ($M+NH_4^+$); ν_{max} 3033, 2954, 2916, 1746, 1724, 1552, 1496, 1434, 1378, 1247, 1199, 1169, 1092, 1034, 981, 942, 893, 766, 700, 618 cm^{-1}. ^{1}H-NMR (500 MHz, $CDCl_3$) for the major diastereomer: δ 1.61 (*s*, 3H), 1.74 (*s*, 3H), 3.73 (*s*, 3H), 3.76 – 3.90 (*m*, 2H), 4.21 – 4.32 (*m*, 3H), 4.79 – 4.95 (*m*, 3H), 5.20 – 5.24 (*m*, 1H), 7.18 – 7.24 (*m*, 2H), 7.25 – 7.33 (*m*, 3H). ^{1}H-NMR (500 MHz, $CDCl_3$) for the minor diastereomer: δ 1.66 (*s*, 3H), 1.75 (*s*, 3H), 3.52 (s, 3H), 3.98 (*d*, *J*=7.0 Hz, 2H), 4.02 (*d*, *J*=17.5 Hz, 1H), 4.10 (*d*, *J*=17.4 Hz, 1H). ^{13}C-NMR (126 MHz, $CDCl_3$): δ 18.12, 18.16, 25.90, 25.93, 42.13, 42.35, 52.80, 52.96, 56.60, 57.33, 67.75, 67.94, 74.72, 77.22, 77.77, 119.78, 119.80, 128.05, 128.22, 128.43, 128.50, 129.10, 129.22, 136.39, 136.41, 138.70, 138.84, 167.43, 167.90, 202.15, 202.77 (1 signal missing due to overlapping). HPLC: Chiralpak AS-H, *n*-Hexane/EtOH = 90:10, flow rate 1.0 mL/min, λ = 210 nm, T = 20 °C. Minor diastereomer: enantiomers: *t*R = 14.066 minutes (minor); 17.773 minutes (major) – 94% *ee*. Major diastereomer: enantiomers: *t*R = 17.164 minutes (major); 19.406 minutes (minor) – 91% *ee*.

Synthesis of methyl 2-(2-((3-methylbut-2-en-1-yl)oxy)acetyl)-3-(nitromethyl)octadecanoate (*rac*-17e)

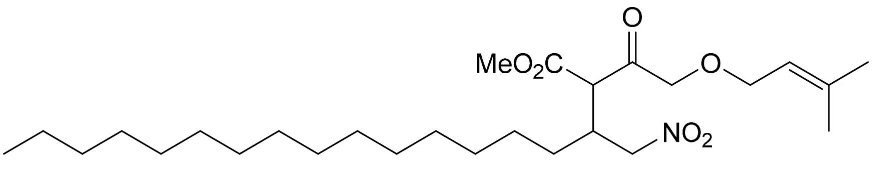

Following *GP3*. Prepared from methyl 4-((3-methylbut-2-en-1-yl)oxy)-3-oxobutanoate (**2c**) (0.3 mmol, 60.1 mg) and (*E*)-1-nitroheptadec-1-ene (**16**) (0.2 mmol, 56.7 mg), organocatalyst **X**, 24 h; isolation by column chromatography (EtOAc/petroleum ether = 1:5). ***rac*-17e** Yield: 52.2 mg (0.108 mmol, 54%, two diastereomers in a ratio of 51:49 in $CDCl_3$) of colorless oil. EI-HRMS: *m/z* = 501.3887 ($M+NH_4^+$); $C_{27}H_{53}N_2O_6$ requires: *m/z* = 501.3898 ($M+NH_4^+$); ν_{max} 2923, 2853, 1726, 1553, 1435, 1379, 1250, 1199, 1158, 1092, 1000, 780, 722 cm^{-1}. ^{1}H-NMR (600 MHz, $CDCl_3$) for both diastereomers: δ 0.88 (*t*, *J*=7.0 Hz, 3H), 1.18 – 1.51 (*m*, 28H), 1.68 (*dd*, *J*=1.4, 4.8 Hz, 3H), 1.77 (*dd*, *J*=1.2, 4.1 Hz, 3H), 2.85 – 2.93 (*m*, 1H), 3.73 (*s*, 1.5H), 3.74 (*s*, 1.5H), 3.94 – 4.15 (*m*, 5H), 4.46 (*dd*, *J*=7.1, 13.3 Hz, 0.5H), 4.53 (*dd*, *J*=5.7, 13.2 Hz, 0.5H), 4.59 – 4.69 (*m*, 1H), 5.27 – 5.34 (*m*, 1H). ^{13}C-NMR (151 MHz, $CDCl_3$) for both diastereomers: δ 14.28, 18.19, 18.21, 22.84, 25.96, 26.88, 27.05, 29.38, 29.45, 29.51, 29.66, 29.75, 29.80, 29.83, 29.85, 30.29, 32.07, 36.10, 36.30, 52.67, 52.82, 54.92, 55.32, 67.99, 68.00, 74.63, 74.73, 76.40, 76.75, 119.82, 119.92, 138.71, 138.89, 168.55, 168.56, 203.55, 203.88 (17 signals missing due to overlapping).

Synthesis of methyl 2-((*R*)-2-nitro-1-phenylethyl)-3-oxooctadecanoate (17f)

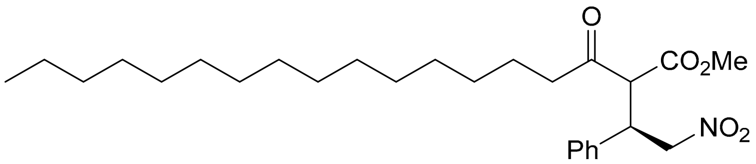

Following *GP3* and *GP4*. Prepared from methyl 3-oxooctadecanoate (**2d**) (0.3 mmol, 93.7 mg) and *trans*-β-nitrostyrene (**12**) (0.2 mmol, 29.8 mg), organocatalyst **VIII**, 24 h; isolation by column chromatography (EtOAc/petroleum ether = 1:10). ***rac*-17f** Yield: 34.2 mg (0.074 mmol, 37%, two diastereomers in a ratio of 56:44 in $CDCl_3$) of white solid. **17f** Yield: 55.4 mg (0.120 mmol, 60%, two diastereomers in a ratio of 50:50 in $CDCl_3$) of white solid; m.p. = 60.0–61.2 °C. EI-HRMS: *m/z* = 462.3214 (MH^+); $C_{27}H_{44}NO_5$ requires: *m/z* = 462.3214 (MH^+); ν_{max} 2915, 2850, 1742, 1711, 1550, 1496, 1471, 1455, 1438, 1383, 1334, 1281, 1245, 1208, 1173, 1128, 1092, 1072, 1033, 1004, 983, 918, 892, 853, 765, 718, 700, 616 cm^{-1}. ^{1}H-NMR (500 MHz, $CDCl_3$) for the major diastereomer: δ 0.88 (*t*, *J*=6.9 Hz, 3H), 0.94 – 1.04 (*m*, 2H), 1.06 – 1.39 (*m*, 22H), 1.51 – 1.60 (*m*, 2H), 2.13 (*dt*, *J*=7.2, 17.8, 1H), 2.38 – 2.50 (*m*, 1H), 3.76 (*s*, 3H), 4.03 (*d*, *J*=10.0 Hz, 1H), 4.19 – 4.28 (*m*, 1H), 4.75 – 4.89 (*m*, 2H), 7.16 – 7.22 (*m*, 2H), 7.24 – 7.34 (*m*, 3H). ^{1}H-NMR (500 MHz, $CDCl_3$) for the minor diastereomer: δ 2.61 (*dt*, *J*=7.4, 17.7 Hz, 1H), 3.52 (*s*, 3H), 4.13 (*d*, *J*=9.5 Hz, 1H). ^{13}C-NMR (126 MHz, $CDCl_3$) for both diastereomers: δ 14.27, 22.83, 23.05, 23.39, 28.74, 29.00, 29.33, 29.45, 29.48, 29.50, 29.56,

29.68, 29.73, 29.76, 29.78, 29.80, 29.83, 32.06, 42.53, 42.86, 43.50, 43.84, 52.87, 53.05, 60.97, 61.34, 77.67, 77.93, 127.94, 128.11, 128.42, 128.50, 129.15, 129.26, 136.44, 136.63, 167.62, 168.14, 202.76, 203.81 (10 signals missing due to overlapping). HPLC: Chiralpak IA-3, *n*-Hexane/EtOH = 95:5, flow rate 1.0 mL/min, λ = 210 nm, T = 25 °C. Minor diastereomer: enantiomers: *t*R = 7.287 minutes (minor); 22.511 minutes (major) – 95% *ee*. Major diastereomer: enantiomers: *t*R = 8.963 minutes (major); 13.215 minutes (minor) – 96% *ee*.

Synthesis of methyl 2-(1-nitroheptadecan-2-yl)-3-oxooctadecanoate (*rac*-17g)

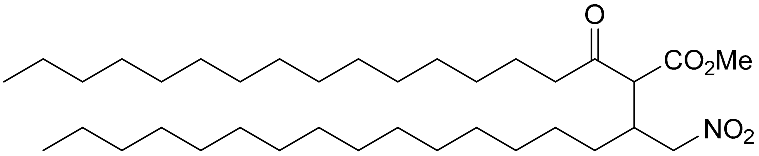

Following *GP3*. Prepared from methyl 3-oxooctadecanoate (**2d**) (0.3 mmol, 93.7 mg) and (*E*)-1-nitroheptadec-1-ene (**16**) (0.2 mmol, 56.7 mg), organocatalyst **X**, 24 h; isolation by column chromatography (EtOAc/petroleum ether = 1:10). ***rac*-17g** Yield: 78.7 mg (0.132 mmol, 66%, two diastereomers in a ratio of 68:32 in $CDCl_3$) of white solid; m.p. = 40.0–40.9 °C. EI-HRMS: *m/z* = 594.5108 $(M\text{-}H^+)^-$; $C_{36}H_{68}NO_5$ requires: *m/z* = 594.5103 $(M\text{-}H^+)^-$; ν_{max} 2955, 2914, 2849, 1730, 1707, 1556, 1543, 1470, 1435, 1402, 1380, 1243, 1204, 1128, 1073, 1000, 863, 719 cm^{-1}. ^{1}H-NMR (600 MHz, $CDCl_3$) for both diastereomers: δ 0.88 (*t*, *J*=7.0, 6H), 1.11 – 1.46 (*m*, 52H), 1.55 – 1.62 (*m*, 2H), 2.47 – 2.55 (*m*, 1H), 2.57 – 2.66 (*m*, 1H), 2.79 – 2.90 (*m*, 1H), 3.75 (*s*, 2.04H), 3.75 (*s*, 0.96H), 3.76 – 3.81 (*m*, 1H), 4.49 – 4.57 (*m*, 1H), 4.59 – 4.67 (m, 1H). ^{13}C-NMR (151 MHz, $CDCl_3$) for both diastereomers: δ 14.28, 22.85, 23.46, 23.50, 26.80, 26.91, 29.08, 29.10, 29.42, 29.45, 29.48, 29.50, 29.52, 29.60, 29.65, 29.67, 29.75, 29.81, 29.83, 29.85, 30.22, 32.08, 36.72, 36.77, 43.43, 43.60, 52.79, 52.91, 59.33, 59.72, 76.16, 76.57, 168.77, 168.81, 204.33, 204.46 (36 signals missing due to overlapping).

Synthesis of methyl 2-((*R*)-2-nitro-1-phenylethyl)-3-oxoicosanoate (17h)

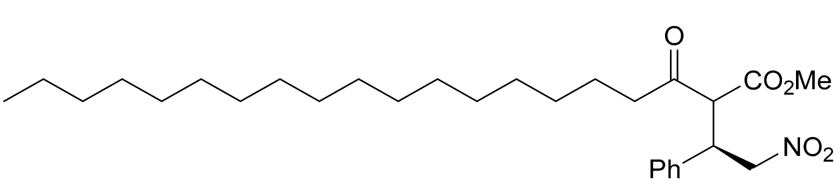

Following *GP3* and *GP4*. Prepared from methyl 3-oxoicosanoate (**2e**) (0.3 mmol, 102.2 mg) and *trans*-β-nitrostyrene (**12**) (0.2 mmol, 29.8 mg), organocatalyst **VIII**, 24 h; isolation by column chromatography (EtOAc/petroleum ether = 1:10). ***rac*-17h** Yield: 43.1 mg (0.088 mmol, 44%, two diastereomers in a ratio of 54:46 in $CDCl_3$) of white solid. **17h** Yield: 52.9 mg

(0.108 mmol, 54%, two diastereomers in a ratio of 48:52 in $CDCl_3$) of white solid; m.p. = 42.2–44.7 °C. EI-HRMS: *m/z* = 490.3524 (MH^+); $C_{29}H_{48}NO_5$ requires: *m/z* = 490.3527 (MH^+); ν_{max} 2914, 2849, 1737, 1712, 1555, 1496, 1471, 1455, 1433, 1404, 1378, 1271, 1198, 1168, 1113, 1082, 982, 891, 765, 716, 699 cm^{-1}. ^{1}H-NMR (500 MHz, $CDCl_3$) for the major diastereomer: δ 0.88 (*t*, *J*=6.9 Hz, 3H), 1.06 – 1.37 (*m*, 30H), 2.38 – 2.50 (*m*, 1H), 2.61 (*dt*, *J*=7.4, 17.7 Hz, 1H), 3.52 (*s*, 3H), 4.13 (*d*, *J*=9.4 Hz, 1H), 4.19 – 4.28 (*m*, 1H), 4.74 – 4.89 (*m*, 2H), 7.16 – 7.22 (*m*, 2H), 7.24 – 7.33 (*m*, 3H). ^{1}H-NMR (500 MHz, $CDCl_3$) for the minor diastereomer: δ 0.94 – 1.04 (*m*, 2H), 1.51 – 1.60 (*m*, 2H), 2.13 (*dt*, *J*=7.1, 17.7 Hz, 1H), 3.75 (*s*, 3H), 4.03 (*d*, *J*=10.0 Hz, 1H). ^{13}C-NMR (126 MHz, $CDCl_3$) for both diastereomers: δ 14.25, 22.82, 23.03, 23.37, 28.72, 28.99, 29.32, 29.43, 29.47, 29.49, 29.55, 29.67, 29.72, 29.75, 29.77, 29.79, 29.82, 32.05, 42.51, 42.86, 43.48, 43.83, 52.84, 53.02, 60.94, 61.33, 77.65, 77.92, 127.93, 128.10, 128.39, 128.47, 129.13, 129.24, 136.44, 136.63, 167.61, 168.12, 202.74, 203.79 (14 signals missing due to overlapping). HPLC: Chiralpak IA-3, *n*-Hexane/EtOH = 95:5, flow rate 1.0 mL/min, λ = 210 nm, T = 25 °C. Major diastereomer: enantiomers: *t*R = 8.838 minutes (minor); 26.885 minutes (major) – 96% *ee*. Minor diastereomer: enantiomers: *t*R = 10.913 minutes (major); 15.371 minutes (minor) – 96% *ee*.

Synthesis of methyl 2-(1-nitroheptadecan-2-yl)-3-oxoicosanoate (*rac*-17i)

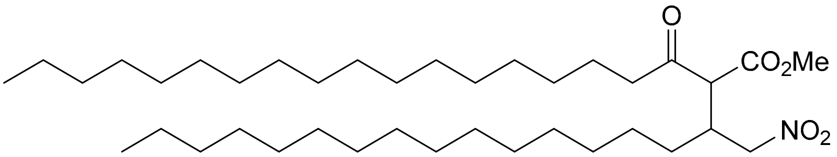

Following *GP3*. Prepared from methyl 3-oxoicosanoate (**2e**) (0.3 mmol, 102.2 mg) and (*E*)-1-nitroheptadec-1-ene (**16**) (0.2 mmol, 56.7 mg), organocatalyst **X**, 24 h; isolation by column chromatography (EtOAc/petroleum ether = 1:10). ***rac*-17i** Yield: 52.4 mg (0.084 mmol, 42%, two diastereomers in a ratio of 50:50 in $CDCl_3$) of white solid; m.p. = 42.0–43.9 °C. EI-HRMS: *m/z* = 622.5416 ($(M\text{-}H^+)^-$); $C_{38}H_{73}NO_5$ requires: *m/z* = 622.5421 ($(M\text{-}H^+)^-$); ν_{max} 2956, 2915, 2848, 1731, 1706, 1556, 1542, 1470, 1435, 1402, 1379, 1349, 1241, 1205, 1128, 1108, 1078, 1001, 719 cm^{-1}. ^{1}H-NMR (500 MHz, $CDCl_3$) for both diastereomers: δ 0.87 (*t*, *J*=6.9 Hz, 6H), 1.08 – 1.44 (*m*, 56H), 1.53 – 1.61 (*m*, 2H), 2.45 – 2.55 (*m*, 1H), 2.56 – 2.66 (*m*, 1H), 2.78 – 2.89 (*m*, 1H), 3.74 (*s*, 1.5H), 3.74 (*s*, 1.5H), 3.75 (*d*, *J*=6.9 Hz, 0.5H), 3.79 (*d*, *J*=7.6 Hz, 0.5H), 4.45 – 4.55 (*m*, 1H), 4.62 (*td*, *J*=4.6, 13.5 Hz, 1H). ^{13}C-NMR (126 MHz, $CDCl_3$) for both diastereomers: δ 14.25, 22.83, 23.43, 23.48, 26.77, 26.88, 29.06, 29.08, 29.40, 29.43, 29.46, 29.48, 29.50, 29.59, 29.64, 29.71, 29.74, 29.79, 29.82, 29.84, 30.20, 32.06, 36.68, 36.74, 43.38,

43.56, 52.74, 52.86, 59.30, 59.69, 76.13, 76.54, 168.74, 168.78, 204.28, 204.41 (40 signals missing due to overlapping).

Synthesis of *tert*-butyl 2-((*R*)-2-nitro-1-phenylethyl)-3-oxoicosanoate (17j)

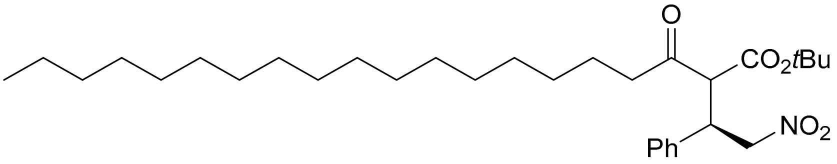

Following *GP3* and *GP4*. Prepared from *tert*-butyl 3-oxoicosanoate (**2f**) (0.3 mmol, 114.8 mg) and *trans*-β-nitrostyrene (**12**) (0.2 mmol, 29.8 mg), organocatalyst **VIII**, 24 h; isolation by column chromatography (EtOAc/petroleum ether = 1:10). ***rac*-17j** Yield: 42.5 mg (0.080 mmol, 40%, two diastereomers in a ratio of 61:39 in $CDCl_3$) of white solid. **17j** Yield: 50.0 mg (0.094 mmol, 47%, two diastereomers in a ratio of 36:64 in $CDCl_3$) of white solid; m.p. = 54.0–56.4 °C. EI-HRMS: *m/z* = 549.4273 ($M+NH_4^+$); $C_{32}H_{57}N_2O_5$ requires: *m/z* = 549.4262 ($M+NH_4^+$); ν_{max} 2916, 2849, 1731, 1712, 1553, 1496, 1468, 1434, 1394, 1378, 1284, 1250, 1148, 1126, 1092, 1062, 982, 914, 838, 770, 751, 720, 700 cm^{-1}. ^1H-NMR (500 MHz, $CDCl_3$) for the major diastereomer: δ 0.88 (*t*, *J*=6.9 Hz, 3H), 0.98 – 1.07 (*m*, 2H), 1.09 – 1.39 (*m*, 28H), 1.46 (*s*, 9H), 1.55 – 1.63 (*m*, 1H), 2.14 (*dt*, *J*=7.1, 17.5 Hz, 1H), 2.38 – 2.52 (*m*, 1H), 3.91 (*d*, *J*=9.9 Hz, 1H), 4.12 – 4.23 (*m*, 1H), 4.65 – 4.76 (*m*, 1H), 7.16 – 7.34 (*m*, 5H). ^1H-NMR (500 MHz, $CDCl_3$) for the minor diastereomer: δ 2.62 (*dt*, *J*=7.4, 17.4 Hz, 1H), 4.01 (*d*, *J*=10.1 Hz, 1H), 4.77 – 4.90 (*m*, 2H). ^{13}C-NMR (126 MHz, $CDCl_3$) for both diastereomers: δ 14.26, 22.83, 23.18, 23.53, 27.52, 27.98, 28.90, 29.15, 29.38, 29.48, 29.50, 29.56, 29.69, 29.73, 29.76, 29.80, 29.83, 32.06, 42.54, 42.79, 42.87, 43.59, 62.16, 62.51, 78.06, 78.40, 82.95, 83.40, 128.18, 128.29, 128.32, 128.36, 128.95, 129.14, 136.81, 136.84, 166.04, 166.75, 203.13, 203.98 (16 signals missing due to overlapping). HPLC: Chiralpak IA-3, *n*-Hexane/EtOH = 95:5, flow rate 1.0 mL/min, λ = 210 nm, T = 25 °C. Minor diastereomer: enantiomers: *t*R = 6.051 minutes (minor); 8.241 minutes (major) – 93% *ee*. Major diastereomer: enantiomers: *t*R = 6.768 minutes (minor); 7.403 minutes (major) – 98% *ee*.

Synthesis of *tert*-butyl 2-(1-nitroheptadecan-2-yl)-3-oxoicosanoate (*rac*-17k)

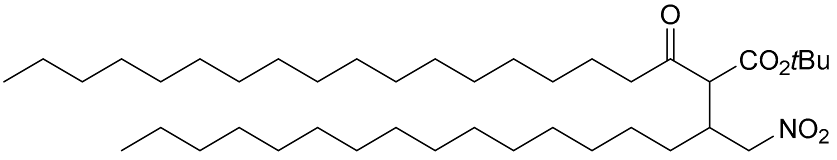

Following *GP3*. Prepared from *tert*-butyl 3-oxoicosanoate (**2f**) (0.3 mmol, 114.8 mg) and (*E*)-1-nitroheptadec-1-ene (**16**) (0.2 mmol, 56.7 mg), organocatalyst **X**, 24 h; isolation by column chromatography (first CC: CH_2Cl_2/petroleum ether = 1:3; second CC: EtOAc/petroleum ether = 1:10). ***rac*-17k** Yield: 51.9 mg (0.079 mmol, 39%, two diastereomers in a ratio of 66:34 in $CDCl_3$) of white solid; m.p. = 41.2–45.0 °C. EI-HRMS: *m/z* = 664.5894 $(M-H^+)^-$; $C_{41}H_{78}NO_5$ requires: *m/z* = 664.5886 $(M-H^+)^-$; ν_{max} 2916, 2848, 1726, 1703, 1556, 1545, 1466, 1370, 1256, 1210, 1157, 1047, 845, 720, 618 cm^{-1}. ^{1}H-NMR (500 MHz, $CDCl_3$) for both diastereomers: δ 0.88 (*t*, *J*=7.0 Hz, 6H), 1.12 – 1.44 (*m*, 56H), 1.47 (*s*, 9H), 1.55 – 1.63 (*m*, 2H), 2.45 – 2.54 (*m*, 1H), 2.56 – 2.67 (*m*, 1H), 2.76 – 2.86 (*m*, 1H), 3.63 (*d*, *J*=6.9, 0.34H), 3.66 (*d*, *J*=7.5 Hz, 0.66H), 4.48 – 4.56 (*m*, 1H), 4.57 – 4.67 (*m*, 1H). ^{13}C-NMR (126 MHz, $CDCl_3$) for both diastereomers: δ 14.26, 22.84, 23.55, 23.60, 26.79, 26.88, 28.03, 28.07, 29.23, 29.49, 29.51, 29.54, 29.60, 29.67, 29.76, 29.81, 29.83, 29.85, 30.19, 32.08, 36.66, 36.77, 43.26, 43.47, 60.43, 60.98, 76.40, 76.77, 83.01, 83.07, 167.40, 167.43, 204.68, 204.83 (44 signals missing due to overlapping).

Synthesis of methyl (11*Z*,14*Z*)-2-((*R*)-2-nitro-1-phenylethyl)-3-oxoicosa-11,14-dienoate (17l)

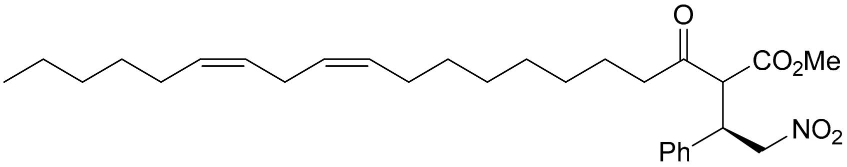

Following *GP3* and *GP4*. Prepared from methyl (11*Z*,14*Z*)-3-oxoicosa-11,14-dienoate (**2g**) (0.3 mmol, 101.0 mg) and *trans*-β-nitrostyrene (**12**) (0.2 mmol, 29.8 mg), organocatalyst **VIII**, 24 h; isolation by column chromatography (EtOAc/petroleum ether = 1:20). ***rac*-17l** Yield: 58 mg (0.120 mmol, 60%, two diastereomers in a ratio of 52:48 in $CDCl_3$) of colorless oil. **17l** Yield: 60.2 mg (0.124 mmol, 62%, two diastereomers in a ratio of 51:49 in $CDCl_3$) of colorless oil. EI-HRMS: *m/z* = 508.3032 (MNa^+); $C_{29}H_{43}NO_5Na$ requires: *m/z* = 508.3032 (MNa^+); ν_{max} 3009, 2925, 2855, 1744, 1717, 1554, 1496, 1455, 1434, 1377, 1243, 1168, 981, 914, 765, 699 cm^{-1}. ^{1}H-NMR (600 MHz, $CDCl_3$) for both diastereomers: δ 0.89 (*td*, *J*=1.5, 7.0 Hz, 3H), 0.95 – 1.05 (*m*, 1H), 1.10 – 1.16 (*m*, 1H), 1.16 – 1.21 (*m*, 1H), 1.23 – 1.40 (*m*, 12H), 1.52 – 1.60 (*m*, 1H), 1.97 – 2.07 (*m*, 4H), 2.13 (*dt*, *J*=7.2, 17.8 Hz, 0.5H), 2.39 – 2.49 (*m*, 1H), 2.61 (*dt*, *J*=7.4, 17.7 Hz, 0.5H), 2.77 (*q*, *J*=6.5 Hz, 2H), 3.52 (*s*, 1.5H), 3.76 (*s*, 1.5H), 4.03 (*d*, *J*=10.0 Hz, 0.5H), 4.13 (*d*, *J*=9.4 Hz, 0.5H), 4.19 – 4.27 (*m*, 1H), 4.74 – 4.88 (*m*, 2H), 5.28 – 5.43 (*m*, 4H), 7.17 – 7.21 (*m*, 2H), 7.22 – 7.34 (*m*, 3H). ^{13}C-NMR (151 MHz, $CDCl_3$) for both diastereomers: δ 14.22, 22.71, 23.03, 23.37, 25.76, 27.30, 27.31, 27.34, 28.70, 28.97, 29.13, 29.20, 29.24, 29.36,

29.48, 29.67, 29.72, 31.66, 42.53, 42.87, 43.48, 43.83, 52.87, 53.05, 60.98, 61.35, 77.67, 77.93, 127.94, 128.00, 128.02, 128.12, 128.20, 128.23, 128.43, 128.51, 129.16, 129.26, 130.13, 130.38, 136.45, 136.64, 168.14, 202.70, 203.76 (9 signals missing due to overlapping). HPLC: Chiralpak IA-3, *n*-Hexane/EtOH = 95:5, flow rate 1.0 mL/min, λ = 210 nm, T = 25 °C. Minor diastereomer: enantiomers: *t*R = 7.439 minutes (minor); 19.360 minutes (major) – 96% *ee*. Major diastereomer: enantiomers: *t*R = 9.402 minutes (major); 12.992 minutes (minor) – 96% *ee*.

Synthesis of methyl (4*S*)-7-((*tert*-butoxycarbonyl)amino)-2-(2-nitro-1-phenylethyl)-3-oxo-4-stearamidoheptanoate (17m)

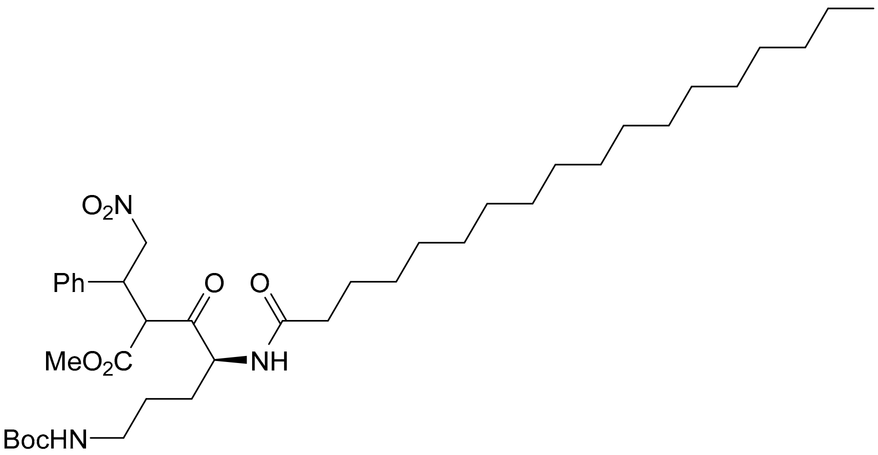

Following *GP3*. Prepared from methyl (*S*)-7-((*tert*-butoxycarbonyl)amino)-3-oxo-4-stearamidoheptanoate (**2h**) (0.2 mmol, 111.0 mg) and *trans*-β-nitrostyrene (**12**) (0.3 mmol, 44.7 mg), organocatalyst **X**, 48 h; isolation by column chromatography (1. EtOAc/petroleum ether = 1:2; 1. EtOAc/petroleum ether = 1:1). **17m** Yield: 100.0 mg (0.142 mmol, 71%, 4 diastereomers in a ratio of 27:21:24:28 in $CDCl_3$) of white semisolid. EI-HRMS: *m/z* = 704.4827 (MH^+); $C_{39}H_{66}N_3O_8$ requires: *m/z* = 704.4844 (MH^+); ν_{max} 3375, 2920, 2851, 1739, 1720, 1687, 1648, 1551, 1518, 1455, 1436, 1365, 1247, 1214, 1168, 1089, 1005, 872, 764, 720, 701, 617 cm^{-1}. ^{1}H-NMR (500 MHz, $CDCl_3$) for 4 diastereomers: δ 0.88 (*t*, *J*=6.9, 3H), 1.05 – 1.39 (*m*, 29H), 1.42 – 1.45 (*m*, 9H), 1.46 – 1.68 (*m*, 4H), 1.71 – 1.86 (*m*, 1H), 2.02 – 2.28 (*m*, 2H), 2.77 – 3.22 (*m*, 2H), 3.48, 3.49, 3.74, 3.77 (4 × *s*, 3H), 4.22 – 4.96 (*m*, 6H), 5.83 – 6.68 (m, 1H), 7.18 – 7.35 (*m*, 5H). ^{13}C-NMR (126 MHz, $CDCl_3$) for 4 diastereomers: δ 14.24, 22.80, 25.55, 25.62, 25.67, 25.90, 26.01, 26.20, 26.30, 26.90, 26.95, 27.05, 27.20, 28.48, 28.49, 29.38, 29.40, 29.43, 29.45, 29.47, 29.60, 29.61, 29.63, 29.73, 29.75, 29.77, 29.81, 32.03, 36.04, 36.40, 36.44, 36.50, 39.56, 39.71, 39.75, 42.54, 42.63, 43.10, 43.25, 52.84, 53.02, 53.11, 53.36, 57.89, 58.13, 58.32, 58.44, 58.62, 58.97, 59.18, 77.27, 77.36, 77.58, 77.70, 79.48, 79.52, 79.67, 79.70,

128.09, 128.14, 128.27, 128.36, 128.42, 128.50, 129.04, 129.18, 129.21, 136.21, 136.27, 136.50, 136.55, 156.26, 156.41, 156.55, 156.59, 166.95, 167.15, 167.30, 167.66, 173.10, 173.47, 173.85, 174.11, 200.93, 201.85, 202.18, 202.94 (69 signals missing due to overlapping).

Synthesis of methyl 2-((*S*)-5-((*tert*-butoxycarbonyl)amino)-2-stearamidopentanoyl)-3-(nitromethyl)octadecanoate (17n)

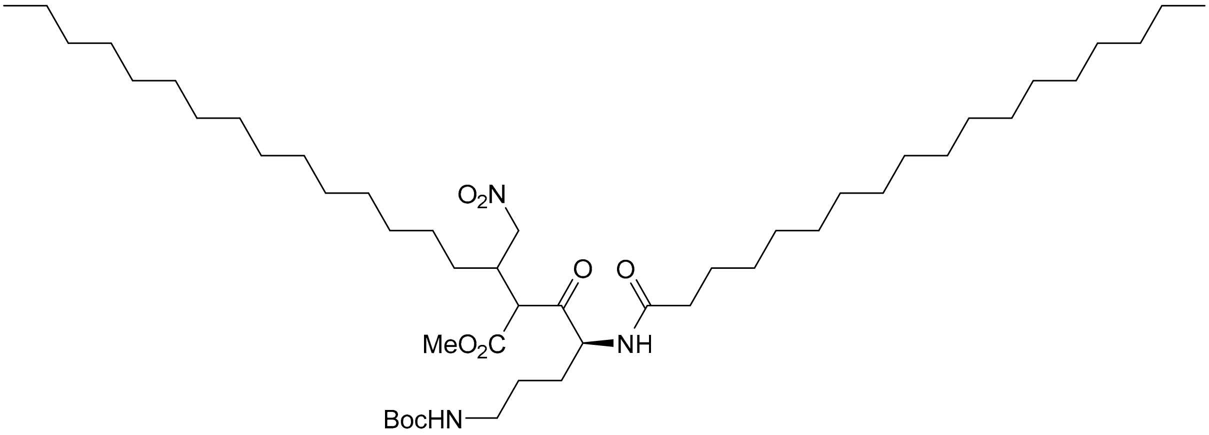

Following *GP3* and *GP4*. Prepared from methyl (*S*)-7-((*tert*-butoxycarbonyl)amino)-3-oxo-4-stearamidoheptanoate (**2h**) (0.2 mmol, 111.0 mg) and (*E*)-1-nitroheptadec-1-ene (**16**) (0.3 mmol, 85.0 mg), organocatalyst **X**, 48 h; isolation by column chromatography (1. EtOAc/petroleum ether = 1:3; 1. EtOAc/petroleum ether = 1:2); **17n** Yield: 129.1 mg (0.154 mmol, 77%, 4 diastereomers in a ratio of 26:28:25:21 in $CDCl_3$) of white solid. Organocatalyst **VIII**, 48 h; isolation by column chromatography (1. EtOAc/petroleum ether = 1:3; 1. EtOAc/petroleum ether = 1:2); **17n** Yield: 147.5 mg (0.176 mmol, 88%, 4 diastereomers in a ratio of 45:11:8:36 in $CDCl_3$) of white solid. EI-HRMS: *m/z* = 838.6860 (MH^+); $C_{48}H_{92}N_3O_8$ requires: *m/z* = 838.6879 (MH^+); ν_{max} 3361, 2917, 2850, 1741, 1718, 1687, 1644, 1553, 1524, 1467, 1366, 1250, 1222, 1171, 1039, 1011, 869, 721, 646 cm^{-1}. ^{1}H-NMR (500 MHz, $CDCl_3$) for 4 diastereomers: δ 0.88 (*t*, *J*=6.9 Hz, 6H), 1.10 – 1.39 (*m*, 57H), 1.44 (*s*, 9H), 1.48 – 1.58 (*m*, 2H), 1.58 – 1.67 (*m*, 2H), 1.87 – 1.99 (*m*, 1H), 2.20 – 2.27 (*m*, 2H), 2.80 – 2.93 (*m*, 1H), 3.05 – 3.24 (*m*, 2H), 3.73, 3.74, 3.76, 3.77 (4 × *s*, 3H), 3.99 (*d*, *J*=6.3 Hz, 0.206H), 4.04 (*d*, *J*=6.9 Hz, 0.255H), 4.09 (*d*, *J*=5.2 Hz, 0.284H), 4.15 (*d*, *J*=7.0 Hz, 0.255H), 4.40 – 4.80 (*m*, 4H), 6.42 – 6.58 (m, 1H). ^{13}C-NMR (126 MHz, $CDCl_3$) for 4 diastereomers: δ 14.23, 22.80, 25.67, 25.70, 26.36, 26.74, 26.77, 26.83, 26.87, 27.08, 27.43, 28.48, 29.42, 29.44, 29.46, 29.48, 29.64, 29.66, 29.72, 29.73, 29.75, 29.78, 29.82, 30.43, 30.52, 32.04, 36.37, 36.43, 36.53, 36.60, 37.22, 37.42, 39.76, 39.79, 39.82, 39.83, 52.77, 52.92, 53.00, 53.15, 55.92, 56.15, 56.47, 58.05, 58.26, 58.39, 58.62, 76.25, 76.43, 76.48, 77.36, 79.56, 156.45, 168.14, 168.38, 168.43, 168.46,

173.58, 173.64, 173.71, 173.80, 203.24, 203.40, 203.67 (128 signals missing due to overlapping).

Synthesis of methyl (4*S*)-2-(2-nitro-1-phenylethyl)-3-oxo-4,7-distearamidoheptanoate (17o)

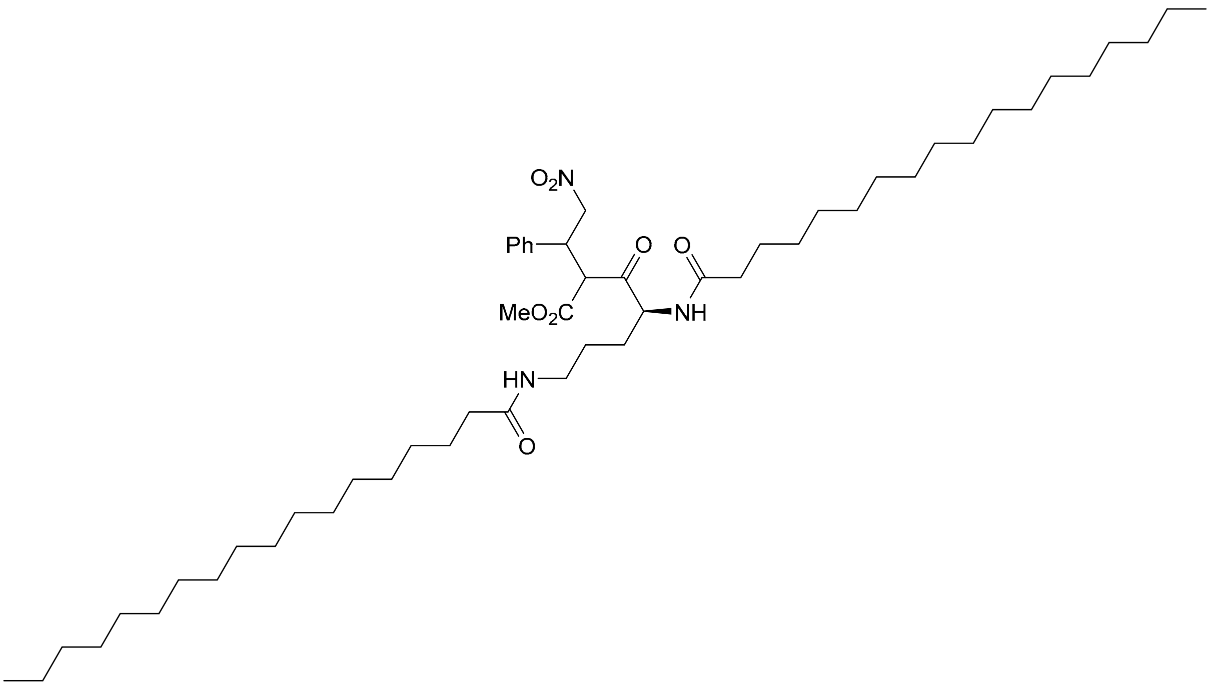

Following *GP3*. Prepared from methyl (*S*)-3-oxo-4,7-distearamidoheptanoate (**2i**) (0.2 mmol, 144.1 mg) and *trans*-β-nitrostyrene (**12**) (0.3 mmol, 44.7 mg), organocatalyst **X**, 48 h; isolation by column chromatography (first column chromatography: EtOAc/petroleum ether = 1:1; second column chromatography: CH_2Cl_2/MeOH = 75:1). **17o** Yield: 50.5 mg (0.058 mmol, 29%, 4 diastereomers in a ratio of 24:22:30:24 in $CDCl_3$) of light orange solid. EI-HRMS: *m/z* = 870.6936 (MH^+); $C_{52}H_{92}N_3O_7$ requires: *m/z* = 870.6930 (MH^+); ν_{max} 3292, 2916, 2848, 1745, 1720, 1638, 1552, 1462, 1434, 1377, 1274, 1240, 1221, 1205, 1168, 1114, 755, 719, 700 cm^{-1}. ^{1}H-NMR (500 MHz, $CDCl_3$) for 4 diastereomers: δ 0.88 (*t*, *J*=6.9 Hz, 6H), 0.99 – 1.42 (*m*, 56H), 1.42 – 1.68 (*m*, 6H), 1.69 – 1.86 (*m*, 1H), 2.05 – 2.29 (*m*, 4H), 2.91 – 3.35 (*m*, 3H), 3.47, 3.48, 3.74, 3.77 (4 × *s*, 3H), 4.22 – 4.65 (*m*, 3H), 4.72 – 4.95 (*m*, 2H), 5.57 – 5.85 (m, 1H), 6.14 (*d*, *J*=7.9 Hz, 0.244H), 6.56 (*d*, *J*=7.9 Hz, 0.296H), 6.85 (br *s*, 0.225H), 6.93 (*d*, *J*=7.1 Hz, 0.235H), 7.18 – 7.34 (*m*, 5H). ^{13}C-NMR (126 MHz, $CDCl_3$) for 4 diastereomers: δ 14.24, 22.81, 25.55, 25.60, 25.63, 25.67, 25.77, 25.91, 26.08, 26.27, 26.57, 26.77, 27.03, 27.27, 29.41, 29.43, 29.48, 29.50, 29.64, 29.66, 29.78, 29.80, 29.83, 32.04, 36.02, 36.38, 36.46, 36.89, 36.91, 38.52, 38.61, 38.68, 38.71, 42.54, 42.64, 43.09, 43.31, 52.83, 52.99, 53.14, 53.32, 58.08, 58.17, 58.19, 58.25, 58.27, 58.82, 58.95, 59.10, 77.29, 77.36, 77.56, 77.65, 77.69, 128.11, 128.16, 128.26, 128.36, 128.42, 128.49, 129.03, 129.17, 136.21, 136.33, 136.52, 136.60, 167.03, 167.21,

167.40, 167.69, 173.31, 173.74, 173.80, 174.07, 174.08, 174.21, 201.14, 201.89, 202.14, 203.10 (128 signals missing due to overlapping).

Synthesis of methyl 2-((*S*)-2,5-distearamidopentanoyl)-3-(nitromethyl)octadecanoate (17p)

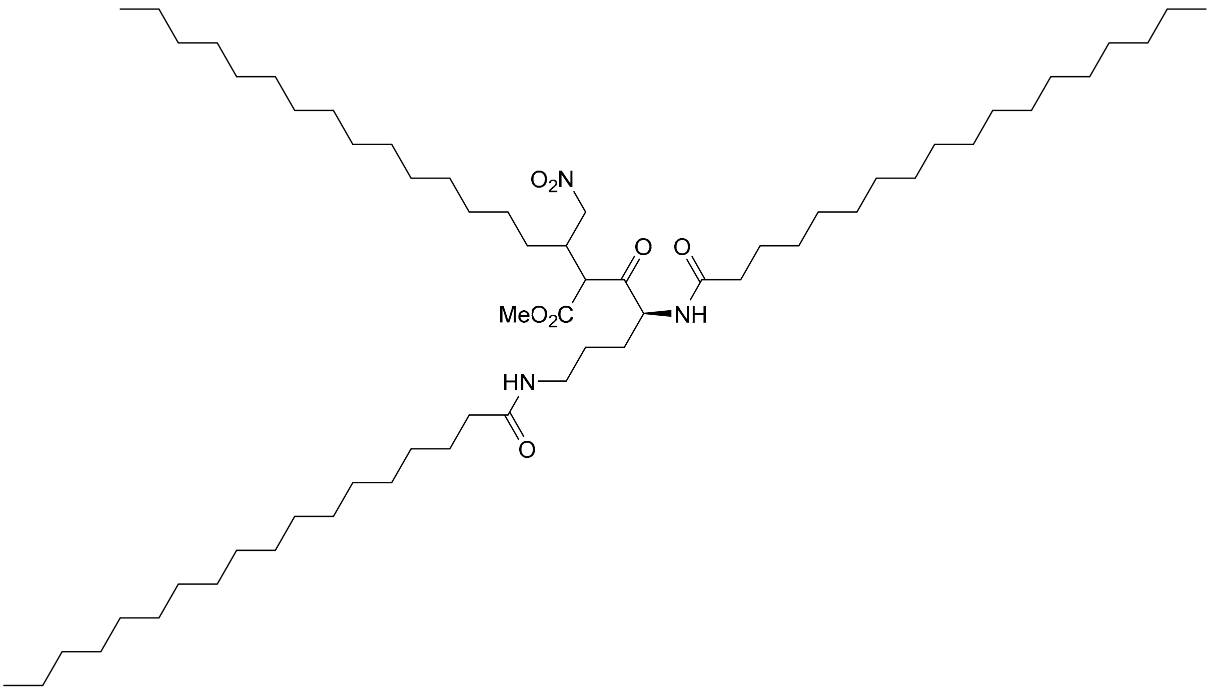

Following *GP3* and *GP4*. Prepared from methyl (*S*)-3-oxo-4,7-distearamidoheptanoate (**2i**) (0.2 mmol, 144.1 mg) and (*E*)-1-nitroheptadec-1-ene (**16**) (0.3 mmol, 85.0 mg), organocatalyst **X**, 48 h; isolation by column chromatography (1. EtOAc/petroleum ether = 1:3; 1. EtOAc/petroleum ether = 1:1); **17p** Yield: 50.2 mg (0.05 mmol, 25%, 4 diastereomers in a ratio of 25:25:27:23 in $CDCl_3$) of light orange solid. Organocatalyst **VIII**, 48 h; isolation by column chromatography (1. EtOAc/petroleum ether = 1:3; 1. EtOAc/petroleum ether = 1:1); **17p** Yield: 46.2 mg (0.046 mmol, 23%, 4 diastereomers in a ratio of 39:11:12:38 in $CDCl_3$) of light orange solid. EI-HRMS: *m/z* = 1004.8949 (MH^+); $C_{61}H_{118}N_3O_7$ requires: *m/z* = 1004.8964 (MH^+); ν_{max} 3293, 2916, 2849, 1746, 1721, 1639, 1552, 1463, 1378, 1274, 1257, 1240, 1222, 1204, 719, 615 cm^{-1}. ^{1}H-NMR (500 MHz, $CDCl_3$) for 4 diastereomers: δ 0.88 (*t*, *J*=6.9, 9H), 1.07 – 1.46 (m, 88H), 1.48 – 1.70 (*m*, 7H), 1.83 – 1.98 (*m*, 1H), 2.14 – 2.21 (*m*, 2H), 2.21 – 2.30 (*m*, 2H), 2.81 – 2.92 (*m*, 1H), 3.17 – 3.28 (*m*, 1H), 3.28 – 3.40 (*m*, 1H), 3.73, 3.74, 3.76, 3.77 (4 × *s*, 3H), 3.98 (*d*, *J*=5.9 Hz, 0.235H), 4.03 (*d*, *J*=7.0 Hz, 0.270H), 4.08 (*d*, *J*=5.2 Hz, 0.249H), 4.14 (*d*, *J*=7.0 Hz, 0.246H), 4.37 – 4.78 (*m*, 3H), 5.79 – 5.89 (*m*, 1H), [6.72 (*d*, *J*=7.6 Hz), 6.76 (*d*, *J*=7.7 Hz), 6.77 (*d*, *J*=7.8 Hz), 6.89 (*d*, *J*=7.6 Hz); 1H]. ^{13}C-NMR (126 MHz, $CDCl_3$) for 4 diastereomers: δ 22.81, 25.65, 25.68, 25.69, 25.91, 25.93, 26.35, 26.41, 26.45, 26.49, 26.75, 26.79, 26.85, 26.89, 27.11, 27.40, 27.57, 29.44, 29.45, 29.49, 29.52, 29.57, 29.66, 29.67, 29.69,

29.71, 29.74, 29.76, 29.79, 29.81, 29.83, 30.44, 30.59, 32.05, 36.34, 36.39, 36.46, 36.48, 36.56, 36.92, 37.19, 37.40, 38.78, 38.81, 52.80, 52.94, 52.97, 53.16, 55.83, 55.89, 56.43, 56.55, 58.28, 58.41, 58.46, 58.56, 76.25, 76.50, 76.58, 77.36, 168.25, 168.36, 168.52, 173.80, 173.90, 173.94, 173.95, 174.02, 203.12, 203.63, 203.70 (173 signals missing due to overlapping).

Organocatalyzed Michael addition of pyrrolones 11 to nitroalkene 16 – General procedure for the preparation of racemic mixtures – ***General procedure 5 (GP5)***

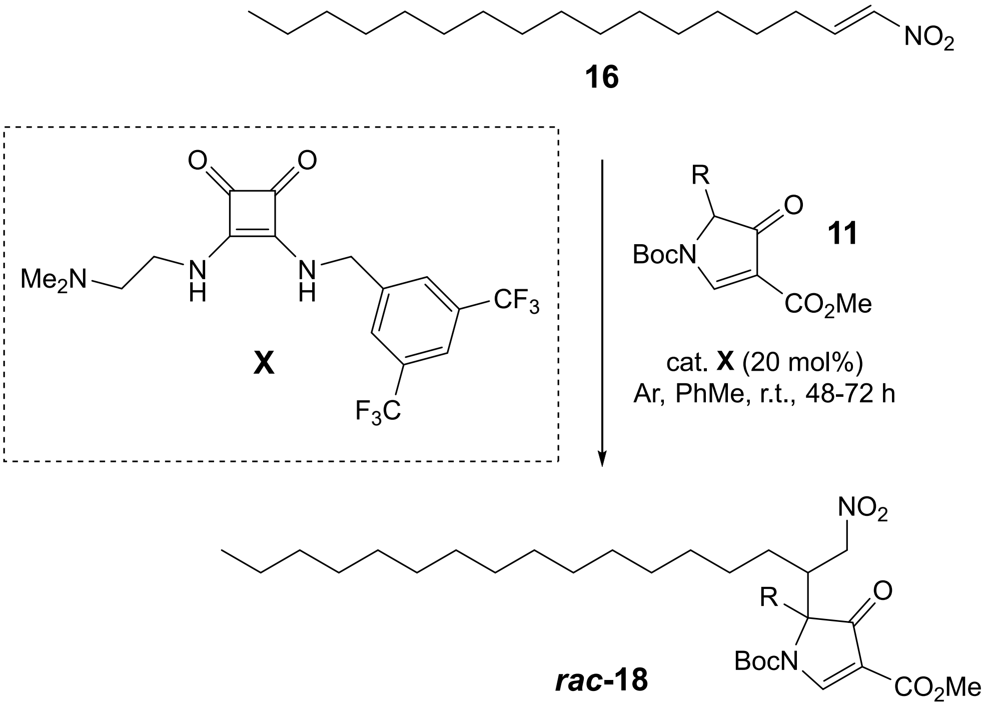

To a solution/suspension of (*E*)-1-nitroheptadec-1-ene (**16**) (0.2 mmol, 56.7 mg; 1.0 equivalent) and the achiral organocatalyst **X** (0.04 mmol, 0.2 equivalents, 16.4 mg) in anhydrous toluene (1 mL) under argon at room temperature, pyrrolone **11** (0.3 mmol, 1.5 equivalents) was added and the resulting reaction mixture was stirred at room temperature for 48–72 hours. The volatiles were evaporated *in vacuo* and the residue was purified by column chromatography (Silica gel 60, mobile phase). The fractions containing the pure racemic product ***rac*-18** were combined and the volatiles were evaporated *in vacuo*. The product ***rac*-18** was fully characterized and analyzed by HPLC.

Organocatalyzed Michael addition of pyrrolones 11 to nitroalkene 16 – General procedure for the organocatalyzed asymmetric addition – *General procedure 6 (GP6)*

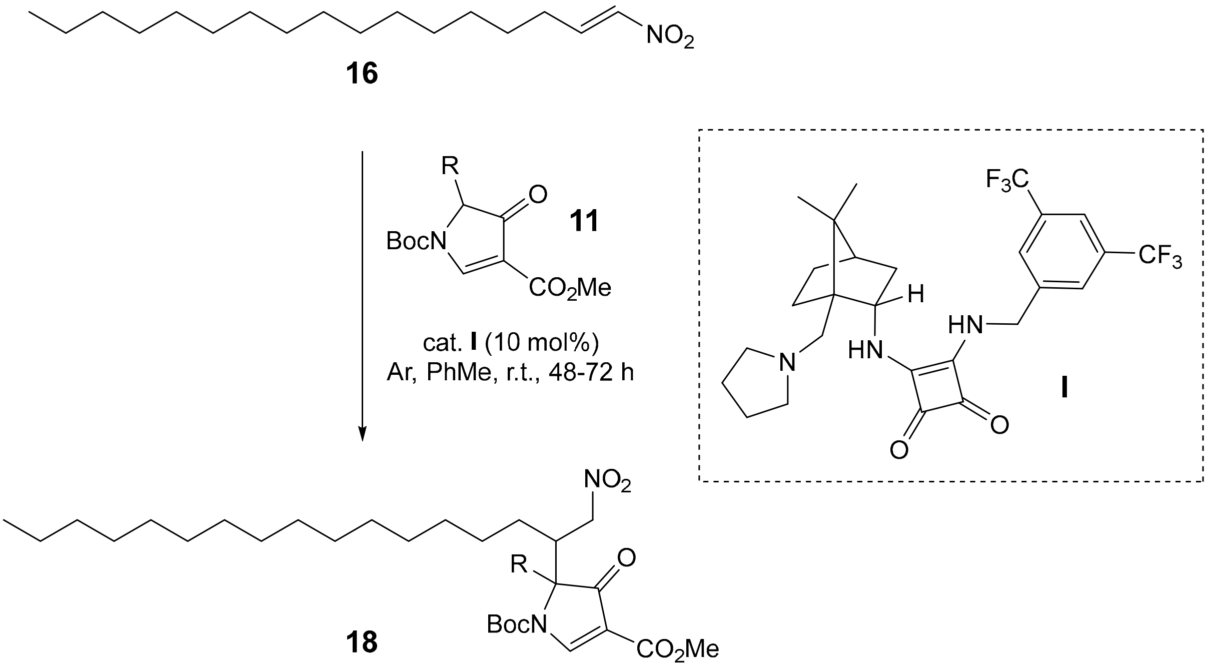

To a solution/suspension of (*E*)-1-nitroheptadec-1-ene (**16**) (0.2 mmol, 56.7 mg; 1.0 equivalent) and the chiral organocatalyst **I** (0.02 mmol, 0.1 equivalents, 10.9 mg) in anhydrous toluene (1 mL) under argon at room temperature, pyrrolone **11** (0.3 mmol, 1.5 equivalents) was added and the resulting reaction mixture was stirred at room temperature for 48–72 hours. The volatiles were evaporated *in vacuo* and the residue was purified by column chromatography (Silica gel 60, mobile phase). The fractions containing the pure chiral nonracemic product **18** were combined the volatiles were evaporated *in vacuo*. The product **18** was fully characterized and analyzed by HPLC.

Synthesis of 1-(*tert*-butyl) 3-methyl (*S*)-5-benzyl-5-((*S*)-1-nitroheptadecan-2-yl)-4-oxo-4,5-dihydro-1*H*-pyrrole-1,3-dicarboxylate (18a)

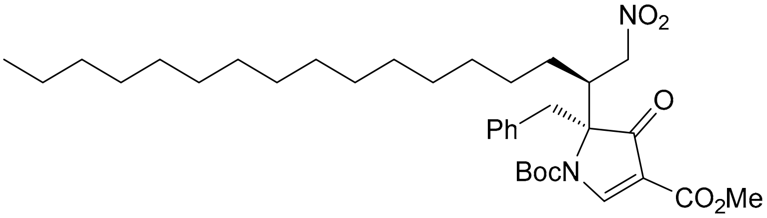

Following *GP5* and *GP6*. Prepared from 1-(*tert*-butyl) 3-methyl 5-benzyl-4-oxo-4,5-dihydro-1*H*-pyrrole-1,3-dicarboxylate (**11a**) (0.3 mmol, 99.4 mg) and (*E*)-1-nitroheptadec-1-ene (**16**) (0.2 mmol, 56.7 mg), organocatalyst **I**, 48 h; isolation by column chromatography

(EtOAc/petroleum ether = 1:5). ***rac*-18a** Yield: 94.7 mg (0.154 mmol, 77%, diastereomer 1/diastereomer 2 = 97:3 in $CDCl_3$) of colorless oil. **18a** Yield: 84.8 mg (0.138 mmol, 69%, diastereomer 1 (*S*,*S*-diastereomer)/diastereomer 2 = 97:3 in $CDCl_3$) of colorless oil. EI-HRMS: *m/z* = 615.3970 (MH^+); $C_{35}H_{55}N_2O_7$ requires: *m/z* = 615.4004 (MH^+); ν_{max} 2923, 2853, 1732, 1713, 1581, 1554, 1497, 1456, 1438, 1370, 1295, 1224, 1143, 1089, 1063, 992, 914, 876, 843, 760, 725, 703, 628 cm^{-1}. ^{1}H-NMR (500 MHz, $CDCl_3$) for diastereomer 1 (*S*,*S*-diastereomer): δ 0.88 (*t*, *J*=6.9 Hz, 3H), 1.07 – 1.43 (*m*, 26H), 1.44 – 1.85 (*m*, 11H), 3.26 – 3.54 (*m*, 3H), 3.77 (*s*, 3H), 4.31 (*dd*, *J*=5.1, 13.7 Hz, 1H), 4.51 (br *s*, 1H), 6.95 – 7.03 (*m*, 2H), 7.11 – 7.21 (*m*, 3H), 8.60 (br *s*, 1H). ^{1}H-NMR (500 MHz, $CDCl_3$) for diastereomer 2: δ 3.71 (*s*, 3H). ^{13}C-NMR (126 MHz, $CDCl_3$) for diastereomer 1 (*S*,*S*-diastereomer): δ 14.26, 22.83, 27.55, 27.74, 28.10, 29.50, 29.52, 29.61, 29.63, 29.73, 29.78, 29.79, 29.82, 29.83, 32.06, 39.94, 43.20, 51.92, 76.56, 85.81, 112.39, 127.69, 128.43, 129.63, 133.25, 147.60, 161.78, 164.91, 196.43 (2 signals missing due to overlapping). HPLC: Chiralpak IA-3, *n*-Hexane/*i*PrOH = 95:5, flow rate 1.0 mL/min, λ = 210 nm, T = 25 °C. Major diastereomer: enantiomers: *t*R = 9.350 minutes (major); 13.217 minutes (minor) – 82% *ee*.

Synthesis of 1-(*tert*-butyl) 3-methyl 5-(4-(((benzyloxy)carbonyl)amino)butyl)-5-(1-nitroheptadecan-2-yl)-4-oxo-4,5-dihydro-1*H*-pyrrole-1,3-dicarboxylate (18b)

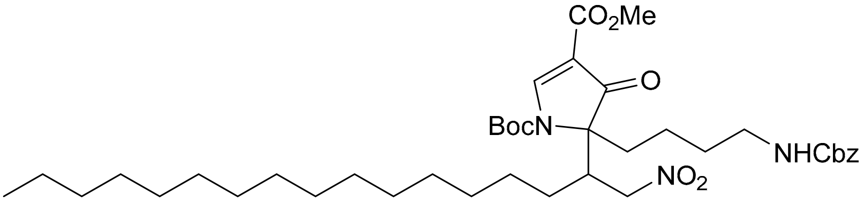

Following *GP5* and *GP6*. Prepared from 1-(*tert*-butyl) 3-methyl 5-(4-(((benzyloxy)carbonyl)amino)butyl)-4-oxo-4,5-dihydro-1*H*-pyrrole-1,3-dicarboxylate (**11b**) (0.3 mmol, 134.0 mg) and (*E*)-1-nitroheptadec-1-ene (**16**) (0.2 mmol, 56.7 mg), organocatalyst **I**, 72 h; isolation by column chromatography (EtOAc/petroleum ether = 1:2). ***rac*-18b** Yield: 94.9 mg (0.130 mmol, 65%, diastereomer 1/diastereomer 2 = 90:10 in $CDCl_3$) of colorless oil. **18b** Yield: 26.3 mg (0.036 mmol, 18%, diastereomer 1/diastereomer 2 = 26:74 in $CDCl_3$) of colorless oil. EI-HRMS: *m/z* = 730.4644 (MH^+); $C_{40}H_{64}N_3O_9$ requires: *m/z* = 730.4637 (MH^+); ν_{max} 3675, 3369, 2923, 2854, 1711, 1582, 1553, 1455, 1438, 1394, 1371, 1280, 1226, 1140, 1067, 846, 763, 697 cm^{-1}. ^{1}H-NMR (500 MHz, $CDCl_3$) for diastereomer 1: δ 0.88 (*t*, *J*=6.9 Hz, 3H), 0.91 – 1.03 (*m*, 2H), 1.06 – 1.34 (*m*, 27H), 1.35 – 1.49 (*m*, 4H), 1.56 (*s*, 9H), 1.96 – 2.07 (*m*, 1H), 2.10 – 2.22 (*m*, 1H), 3.03 – 3.17 (*m*, 3H), 3.84 (*s*, 3H), 4.22 (*dd*, *J*=5.3, 13.8 Hz, 1H), 4.30 – 4.41 (*m*, 1H), 4.71 (*t*, *J*=6.2 Hz, 1H), 5.06 (*s*, 2H), 7.27 – 7.39 (*m*, 5H). ^{1}H-NMR (500

MHz, $CDCl_3$) for diastereomer 2: δ 1.58 (*s*, 9H), 1.87 – 1.96 (*m*, 1H), 3.28 – 3.36 (*m*, 1H), 3.83 (*s*, 3H), 4.28 (*dd*, *J*=5.3, 14.6 Hz, 1H), 5.18 (*dd*, *J*=5.5, 14.7 Hz, 1H), 9.19 (*s*, 1H). ^{13}C-NMR (126 MHz, $CDCl_3$) for diastereomer 1: δ 14.26, 20.18, 22.82, 27.41, 27.49, 28.02, 29.49, 29.55, 29.62, 29.71, 29.77, 29.78, 29.81, 29.82, 32.05, 33.87, 40.61, 43.36, 52.03, 66.78, 76.35, 77.36, 86.22, 112.22, 128.24, 128.64, 136.59, 147.42, 156.40, 161.95, 165.24, 196.41 (4 signals missing due to overlapping). ^{13}C-NMR (126 MHz, $CDCl_3$) for diastereomer 1 and diastereomer 2 (diastereomer 1/diastereomer 2 = 26:74): δ 14.26, 20.19, 20.45, 22.82, 27.18, 27.50, 27.91, 28.03, 28.12, 29.44, 29.49, 29.50, 29.56, 29.63, 29.67, 29.72, 29.75, 29.79, 29.82, 29.83, 32.05, 33.89, 40.66, 41.98, 43.37, 52.03, 52.05, 66.79, 74.38, 76.11, 76.36, 77.40, 86.21, 112.23, 112.31, 128.25, 128.65, 136.59, 147.43, 156.39, 161.92, 165.29, 196.41, 197.67 (28 signals missing due to overlapping). HPLC: Chiralpak IA-3, *n*-Hexane/*i*PrOH = 90:10, flow rate 1.0 mL/min, λ = 210 nm, T = 25 °C. Major diastereomer: enantiomers: *t*R = 24.088 minutes (minor); 31.095 minutes (major) – 57% *ee*. Minor diastereomer: enantiomers: *t*R = 26.634 minutes (minor); 30.099 minutes (major) – 11% *ee*.

Synthesis of 1-(*tert*-butyl) 3-methyl 5-(3-(benzyloxy)-3-oxopropyl)-5-(1-nitroheptadecan-2-yl)-4-oxo-4,5-dihydro-1*H*-pyrrole-1,3-dicarboxylate (18c)

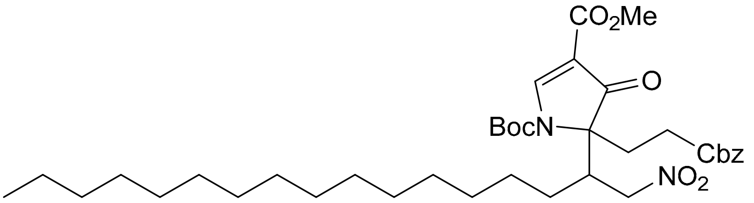

Following *GP5* and *GP6*. Prepared from 1-(*tert*-butyl) 3-methyl 5-(3-(benzyloxy)-3-oxopropyl)-4-oxo-4,5-dihydro-1*H*-pyrrole-1,3-dicarboxylate (**11c**) (0.3 mmol, 121.0 mg) and (*E*)-1-nitroheptadec-1-ene (**16**) (0.2 mmol, 56.7 mg), organocatalyst **I**, 48 h; isolation by column chromatography (EtOAc/petroleum ether = 1:5). ***rac*-18c** Yield: 92.0 mg (0.134 mmol, 67%, diastereomer 1/diastereomer 2 = 93:7 in $CDCl_3$) of colorless oil. **18c** Yield: 42.6 mg (0.062 mmol, 31%, diastereomer 1/diastereomer 2 = 31:69 in $CDCl_3$) of colorless oil. EI-HRMS: *m/z* = 687.4224 (MH^+); $C_{38}H_{59}N_2O_9$ requires: *m/z* = 687.4215 (MH^+); ν_{max} 3675, 2923, 2854, 1735, 1713, 1582, 1554, 1439, 1393, 1371, 1279, 1256, 1227, 1141, 1077, 846, 800, 752, 698 cm^{-1}. ^{1}H-NMR (600 MHz, $CDCl_3$) for diastereomer 1: δ 0.88 (*t*, *J*=6.9 Hz, 3H), 1.17 – 1.34 (*m*, 27H), 1.35 – 1.46 (*m*, 1H), 1.56 (*s*, 9H), 1.98 – 2.06 (*m*, 1H), 2.09 – 2.17 (*m*, 1H), 2.37 – 2.45 (*m*, 1H), 2.48 – 2.55 (*m*, 1H), 3.13 – 3.20 (*m*, 1H), 3.83 (*s*, 3H), 4.25 (*dd*, *J*=5.6, 13.8 Hz, 1H), 4.30 – 4.39 (*m*, 1H), 5.07 (*s*, 2H), 7.28 – 7.39 (*m*, 5H), 9.08 (*s*, 1H). ^{1}H-NMR (500 MHz, $CDCl_3$) for diastereomer 2: δ 1.58 (*s*, 9H), 2.26 – 2.33 (*m*, 1H), 2.54 – 2.62 (*m*, 1H), 3.35 – 3.41

(*m*, 1H), 3.82 (*s*, 3H), 5.06 (*s*, 2H), 5.19 (*dd*, *J*=5.1, 14.7 Hz, 1H), 9.17 (*s*, 1H). ^{13}C-NMR (151 MHz, $CDCl_3$) for diastereomer 1: δ 14.26, 22.83, 27.48, 27.60, 28.01, 29.13, 29.49, 29.50, 29.54, 29.62, 29.72, 29.77, 29.79, 29.82, 29.84, 32.06, 43.15, 52.06, 66.88, 75.92, 76.42, 86.62, 112.16, 128.44, 128.47, 128.56, 128.75, 135.56, 147.29, 161.76, 165.19, 171.48, 195.61 (1 signal missing due to overlapping). ^{13}C-NMR (126 MHz, $CDCl_3$) for diastereomer 1 and diastereomer 2 (diastereomer 1/diastereomer 2 = 31:69): δ 14.28, 22.84, 27.17, 27.49, 28.02, 28.07, 28.11, 28.37, 29.28, 29.44, 29.50, 29.56, 29.64, 29.68, 29.73, 29.76, 29.80, 29.83, 29.85, 32.07, 41.85, 43.16, 52.06, 52.08, 66.89, 66.93, 74.46, 75.26, 75.93, 76.42, 77.39, 86.67, 112.15, 112.36, 128.45, 128.48, 128.58, 128.77, 135.51, 135.56, 147.29, 161.72, 161.77, 165.20, 165.34, 171.18, 171.49, 195.63, 196.88 (19 signal missing due to overlapping). HPLC: Chiralpak IA-3, *n*-Hexane/*i*PrOH = 90:10, flow rate 1.0 mL/min, λ = 280 nm, T = 25 °C. Major diastereomer: enantiomers: *t*R = 10.649 minutes (major); 12.074 minutes (minor) – 68% *ee*. Minor diastereomer: enantiomers: *t*R = 9.991 minutes (minor); 12.514 minutes (major) – 38% *ee*.

Synthesis of 4-hydroxy-3-(1-nitroheptadecan-2-yl)furan-2(5*H*)-one (20)

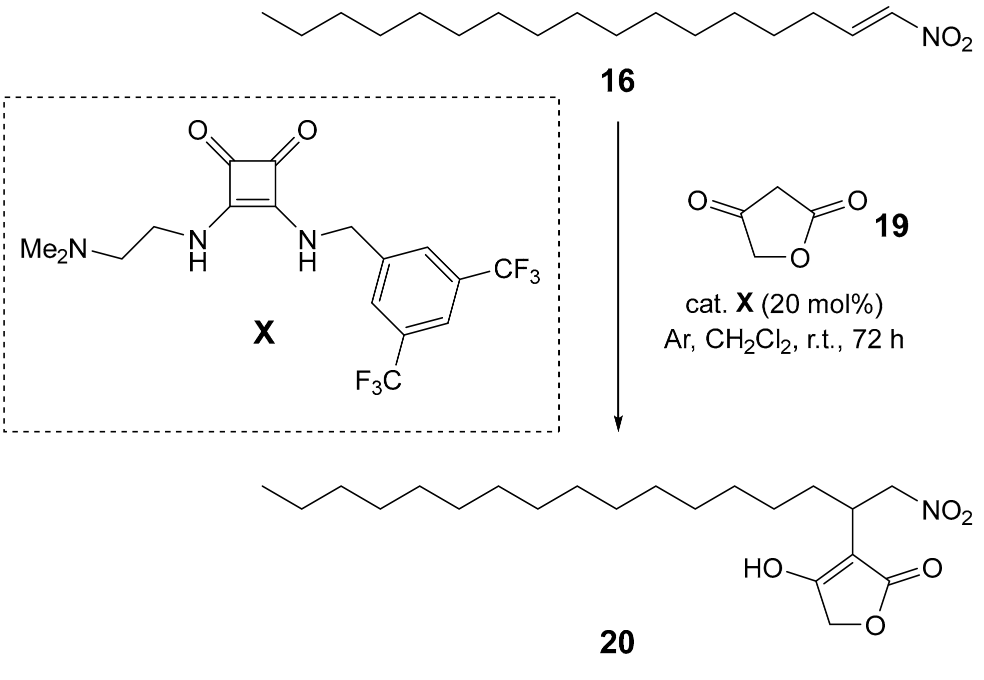

To a solution of (*E*)-1-nitroheptadec-1-ene (**16**) (0.2 mmol, 56.7 mg) and the achiral organocatalyst **X** (0.04 mmol, 0.2 equivalents, 16.4 mg) in anhydrous CH_2Cl_2 (1 mL) under argon at room temperature, tetronic acid (**19**) (0.3 mmol, 30.0 mg) was added and the resulting reaction mixture was stirred at room temperature for 72 hours. The volatiles were evaporated *in vacuo* and the residue was purified by column chromatography (Silica gel 60,

EtOAc/petroleum ether = 1:1). The fractions containing the pure product **20** were combined and the volatiles were evaporated *in vacuo*. Yield: 33.8 mg (0.088 mmol, 44%) of white solid; m.p. = 86.8-88.2 °C. EI-HRMS: *m/z* = 384.2741 (MH^+); $C_{21}H_{38}NO_5$ requires: *m/z* = 384.2744 (MH^+); ν_{max} 2918, 2850, 1714, 1611, 1547, 1428, 1381, 1348, 1279, 1260, 1129, 1097, 1044, 971, 954, 780, 720, 682, 658 cm^{-1}. ^{1}H-NMR (500 MHz, $CDCl_3$): δ 0.88 (*t*, *J*=6.9 Hz, 3H), 1.09 – 1.35 (*m*, 26H), 1.50 – 1.60 (*m*, 1H), 1.65 – 1.77 (*m*, 1H), 3.37 (*tt*, *J*=5.6, 9.6 Hz, 1H), 4.53 (*dd*, *J*=5.6, 12.4 Hz, 1H), 4.71 – 4.81 (*m*, 3H), 10.97 (br *s*, 1H). ^{13}C-NMR (126 MHz, $CDCl_3$): δ 14.27, 22.84, 27.28, 29.44, 29.51, 29.62, 29.76, 29.79, 29.81, 29.84, 29.86, 29.99, 32.07, 32.87, 68.14, 76.88, 99.10, 177.35, 177.91 (2 signals missing due to overlapping).

2. Catalyst and solvent optimization, HPLC data

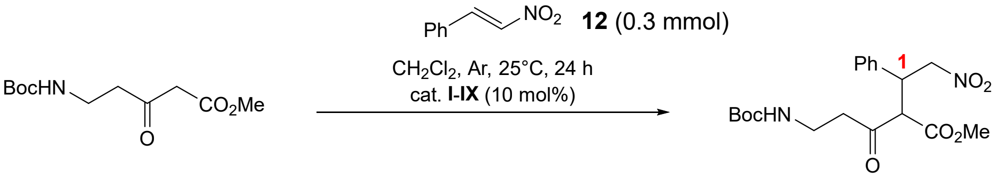

2b (0.2 mmol) **17b**

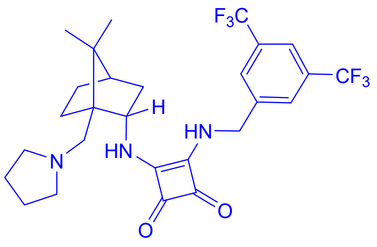

I; 100% conv.
1. 93% *ee* (1*S*)
2. 92% *ee* (1*S*)

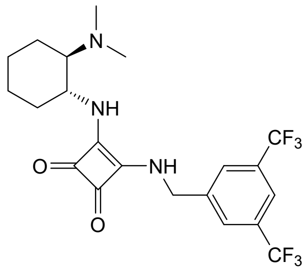

II; 100% conv.
1. 83% *ee* (1*R*)
2. 84% *ee* (1*R*)

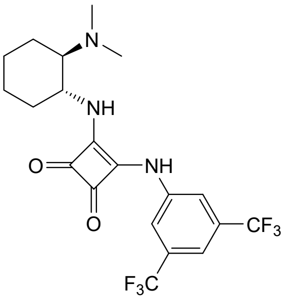

III; 100% conv.
1. 80% *ee* (1*R*)
2. 77% *ee* (1*R*)

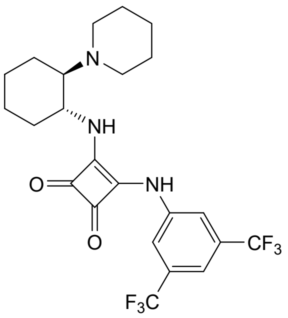

IV; 100% conv.
1. 92% *ee* (1*R*)
2. 95% *ee* (1*R*)

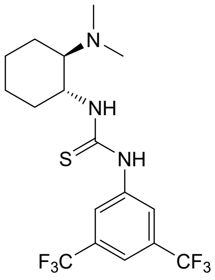

V; 100% conv.
1. 92% *ee* (1*R*)
2. 91% *ee* (1*R*)

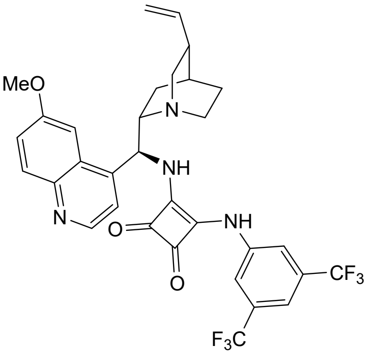

VI; 100% conv.
1. 90% *ee* (1*S*)
2. 91% *ee* (1*S*)

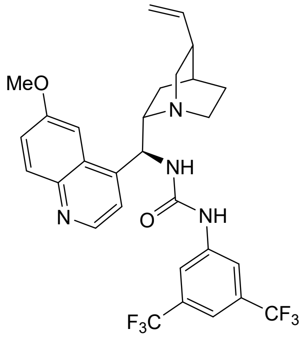

VII; 100% conv.
1. 90% *ee* (1*S*)
2. 91% *ee* (1*S*)

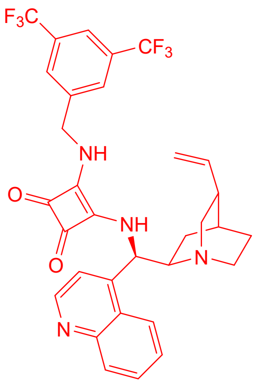

VIII; 100% conv.; 85% yield
1. 95% *ee* (1*R*)
2. 95% *ee* (1*R*)

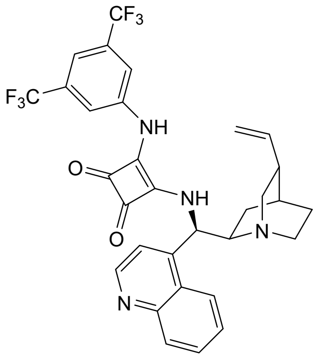

IX; 100% conv.
1. 85% *ee* (1*R*)
2. 85% *ee* (1*R*)

Scheme S1. Evaluation of organocatalysts **I–IX** in Michael addition of *β*-keto ester **2b** (0.2 mmol) to *trans*-β-nitrostyrene (**12**) (0.3 mmol).

Evaluation of organocatalysts **I–IX** in Michael addition of *β*-keto ester **2b** (0.2 mmol) to *trans*-β-nitrostyrene (**12**) (0.3 mmol) (**Scheme S1**).

HPLC: Chiralpak IA-3, *n*-Hexane/*i*-PrOH = 80:20, flow rate 1.0 mL/min, λ = 210 nm, T = 20°C.

Diastereomer 1: *t*R = 7.22 minutes; 8.97 minutes.

Diastereomer 2: *t*R = 12.50 minutes; 20.99 minutes.

Racemate:

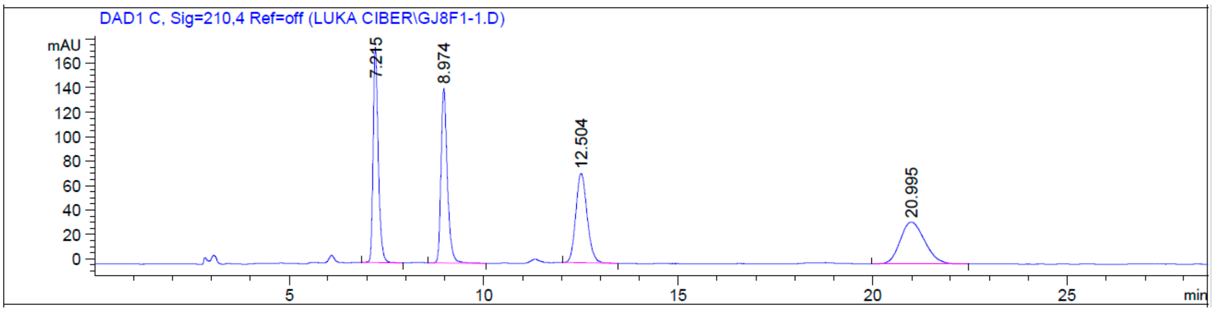

Scheme S1, Entry 1

	Product	Catalyst	Conversion (%)	*ee* (%)
1	**17b**	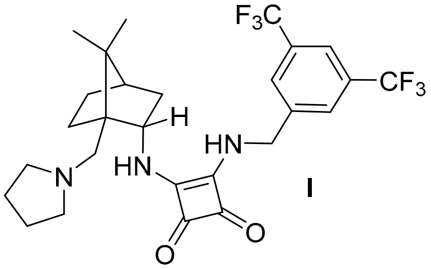 **I**	100	93 (1*S*)<br>92 (1*S*)

HPLC: Chiralpak IA-3, *n*-Hexane/*i*-PrOH = 80:20, flow rate 1.0 mL/min, λ = 210 nm, T = 20°C.

Diastereomer 1: *t*R = 7.22 minutes (major); 8.99 minutes (minor) – 93% *ee*.

Diastereomer 2: *t*R = 12.51 minutes (major); 21.14 minutes (minor) – 92% *ee*.

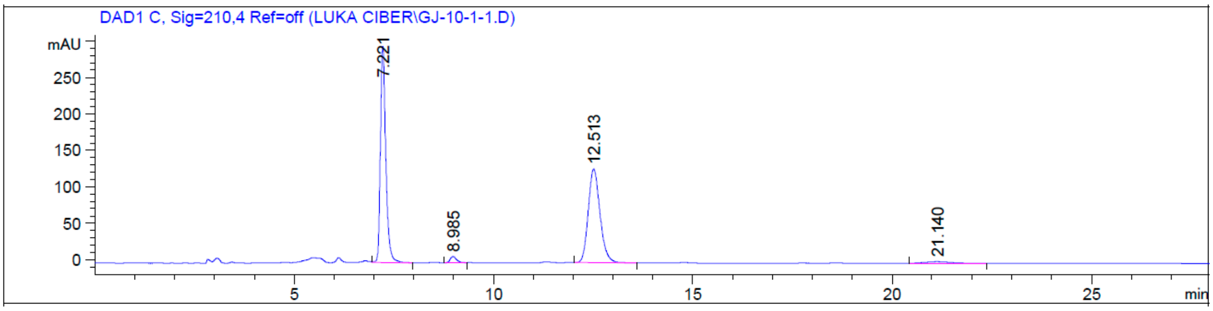

Signal 3: DAD1 C, Sig=210,4 Ref=off

Peak #	RetTime [min]	Type	Width [min]	Area [mAU*s]	Height [mAU]	Area %
1	7.221	VB	0.1413	2774.60645	296.09885	49.4052
2	8.985	BB	0.1649	94.62581	8.69651	1.6849
3	12.513	BB	0.3176	2637.12158	128.38416	46.9572
4	21.140	BB	0.5419	109.66273	2.40518	1.9527
Totals :				5616.01656	435.58468	

Scheme S1, Entry 2

	Product	Catalyst	Conversion (%)	*ee* (%)
2	**17b**	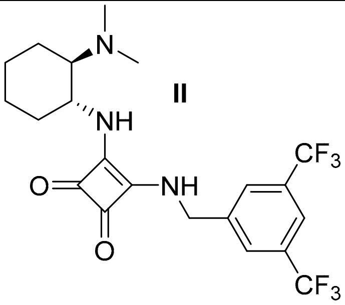 **II**	100	83 (1*R*)<br>84 (1*R*)

HPLC: Chiralpak IA-3, *n*-Hexane/*i*-PrOH = 80:20, flow rate 1.0 mL/min, λ = 210 nm, T = 20°C.

Diastereomer 1: *t*R = 7.29 minutes (minor); 9.06 minutes (major) – 83% *ee*.

Diastereomer 2: *t*R = 12.65 minutes (minor); 21.15 minutes (major) – 84% *ee*.

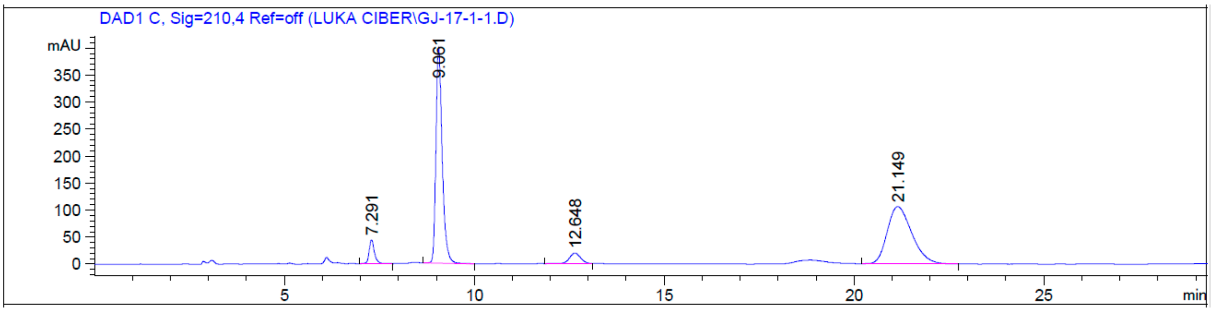

Signal 3: DAD1 C, Sig=210,4 Ref=off

Peak #	RetTime [min]	Type	Width [min]	Area [mAU*s]	Height [mAU]	Area %
1	7.291	BB	0.1448	427.27057	44.18998	4.2069
2	9.061	BB	0.1713	4559.09229	399.13351	44.8888
3	12.648	BV	0.3250	421.49448	19.91055	4.1500
4	21.149	BB	0.6828	4748.56445	105.96630	46.7543

Totals : 1.01564e4 569.20034

Scheme S1, Entry 3

	Product	Catalyst	Conversion (%)	*ee* (%)
3	**17b**	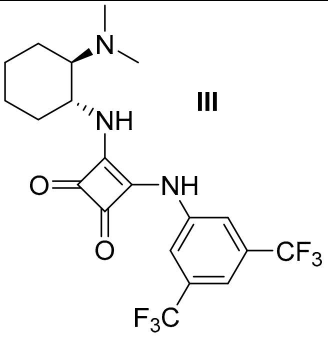 **III**	100	80 (1*R*)<br>77 (1*R*)

HPLC: Chiralpak IA-3, *n*-Hexane/*i*-PrOH = 80:20, flow rate 1.0 mL/min, λ = 210 nm, T = 20°C.

Diastereomer 1: *t*R = 7.29 minutes (minor); 9.07 minutes (major) – 80% *ee*.

Diastereomer 2: *t*R = 12.65 minutes (minor); 21.24 minutes (major) – 77% *ee*.

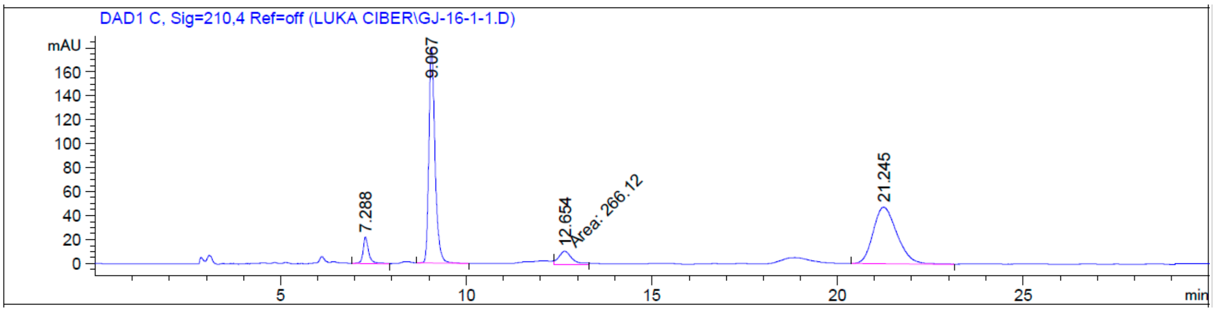

Signal 3: DAD1 C, Sig=210,4 Ref=off

Peak #	RetTime [min]	Type	Width [min]	Area [mAU*s]	Height [mAU]	Area %
1	7.288	BB	0.1541	230.18684	21.99939	4.9282
2	9.067	BB	0.1726	2076.41895	180.04294	44.4548
3	12.654	MM	0.4089	266.11963	10.84706	5.6974
4	21.245	BB	0.6625	2098.13062	47.21210	44.9196

Totals : 4670.85603 260.10149

Scheme S1, Entry 4

	Product	Catalyst	Conversion (%)	*ee* (%)
4	**17b**	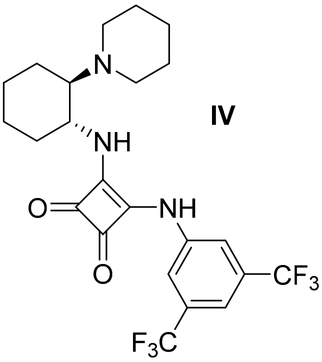 **IV**	100	92 (1*R*)<br>95 (1*R*)

HPLC: Chiralpak IA-3, *n*-Hexane/*i*-PrOH = 80:20, flow rate 1.0 mL/min, λ = 210 nm, T = 20°C.

Diastereomer 1: *t*R = 7.29 minutes (minor); 9.07 minutes (major) – 92% *ee*.

Diastereomer 2: *t*R = 12.65 minutes (minor); 21.21 minutes (major) – 95% *ee*.

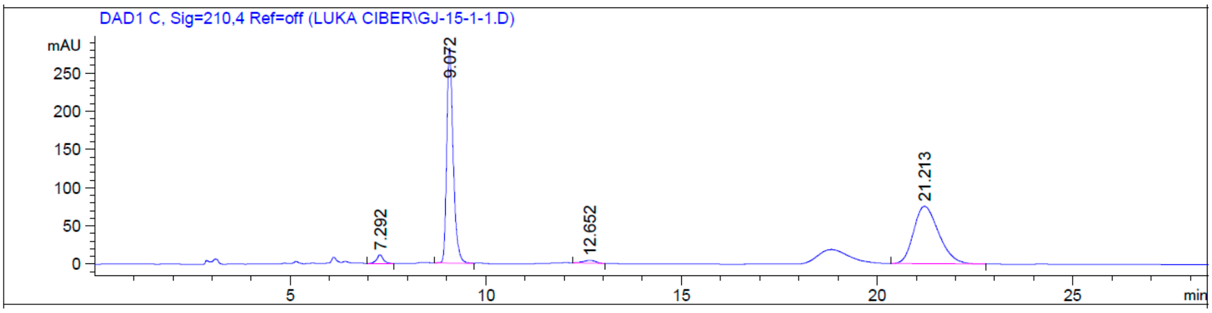


```
Signal 3: DAD1 C, Sig=210,4 Ref=off

Peak RetTime Type  Width     Area      Height     Area
  #   [min]        [min]   [mAU*s]     [mAU]        %
----|-------|----|-------|----------|----------|--------|
   1   7.292 BB    0.1668  130.48752   11.46616   1.9296
   2   9.072 BV    0.1730 3210.81372  281.63260  47.4812
   3  12.652 BB    0.2953   80.72482    3.80778   1.1937
   4  21.213 BB    0.6752 3340.26440   75.06170  49.3955

Totals :                  6762.29046  371.96823
```

Scheme S1, Entry 5

	**Product**	**Catalyst**	**Conversion (%)**	***ee* (%)**
5	**17b**	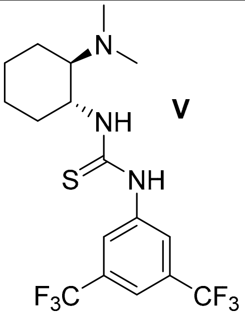 **V**	100	92 (1*R*)<br>91 (1*R*)

HPLC: Chiralpak IA-3, *n*-Hexane/*i*-PrOH = 80:20, flow rate 1.0 mL/min, λ = 210 nm, T = 20°C.

Diastereomer 1: *t*R = 7.23 minutes (minor); 8.98 minutes (major) – 92% *ee*.

Diastereomer 2: *t*R = 12.52 minutes (minor); 20.92 minutes (major) – 91% *ee*.

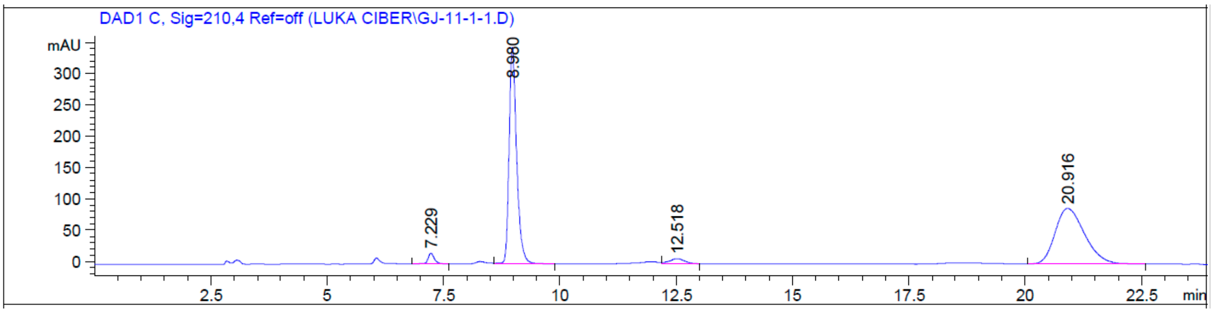

Signal 3: DAD1 C, Sig=210,4 Ref=off

Peak #	RetTime [min]	Type	Width [min]	Area [mAU*s]	Height [mAU]	Area %
1	7.229	BB	0.1452	167.69049	17.27947	2.0353
2	8.980	VB	0.1725	3930.28296	345.99649	47.7017
3	12.518	VB	0.3325	190.24126	8.26593	2.3090
4	20.916	VB	0.6845	3951.07739	88.90484	47.9541

Totals : 8239.29210 460.44673

Scheme S1, Entry 6

	Product	Catalyst	Conversion (%)	*ee* (%)
6	**17b**	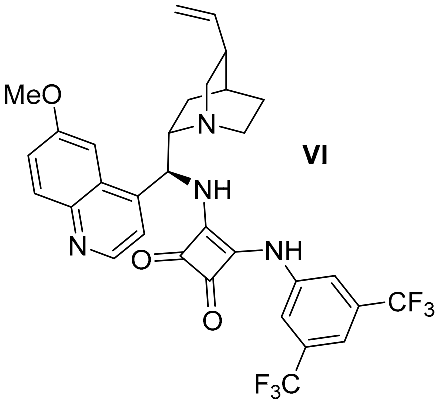 **VI**	100	90 (1*S*)<br>91 (1*S*)

HPLC: Chiralpak IA-3, *n*-Hexane/*i*-PrOH = 80:20, flow rate 1.0 mL/min, λ = 210 nm, T = 20°C.

Diastereomer 1: *t*R = 7.29 minutes (major); 9.08 minutes (minor) – 90% *ee*.

Diastereomer 2: *t*R = 12.64 minutes (major); 21.37 minutes (minor) – 91% *ee*.

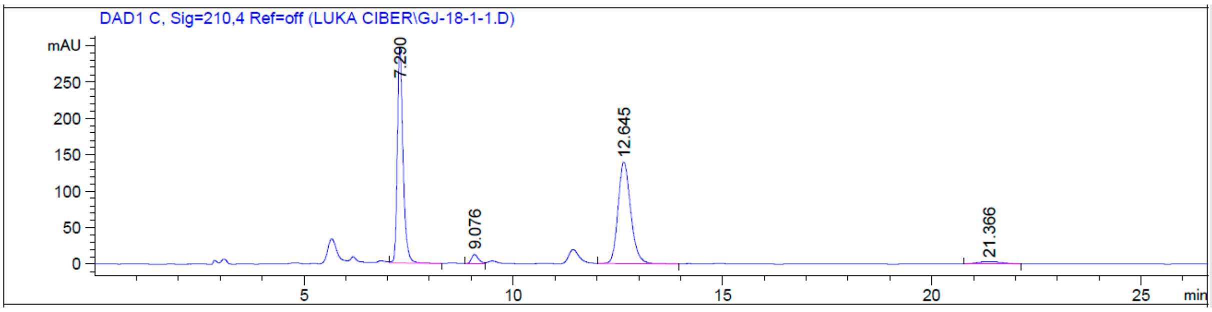


```
Signal 3: DAD1 C, Sig=210,4 Ref=off

Peak RetTime Type  Width     Area      Height     Area
  #   [min]        [min]   [mAU*s]     [mAU]        %
----|-------|----|-------|----------|----------|--------|
   1   7.290 VB    0.1422 2793.28076  295.81036 46.6793
   2   9.076 BV    0.1764  146.35773   12.52099  2.4458
   3  12.645 BB    0.3222 2914.94629  139.24802 48.7125
   4  21.366 BB    0.4942  129.39319    3.15678  2.1623

Totals :                  5983.97797  450.73615
```

Scheme S1, Entry 7

	Product	Catalyst		Conversion (%)	*ee* (%)
7	**17b**	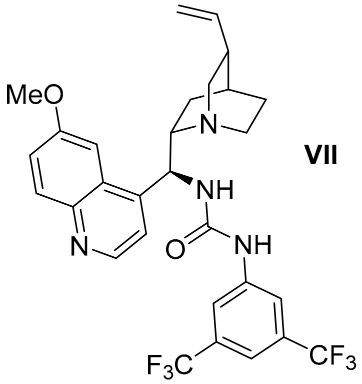	**VII**	100	90 (1*S*)<br>91 (1*S*)

HPLC: Chiralpak IA-3, *n*-Hexane/*i*-PrOH = 80:20, flow rate 1.0 mL/min, λ = 210 nm, T = 20°C.

Diastereomer 1: *t*R = 7.29 minutes (major); 9.08 minutes (minor) – 90% *ee*.

Diastereomer 2: *t*R = 12.66 minutes (major); 21.40 minutes (minor) – 91% *ee*.

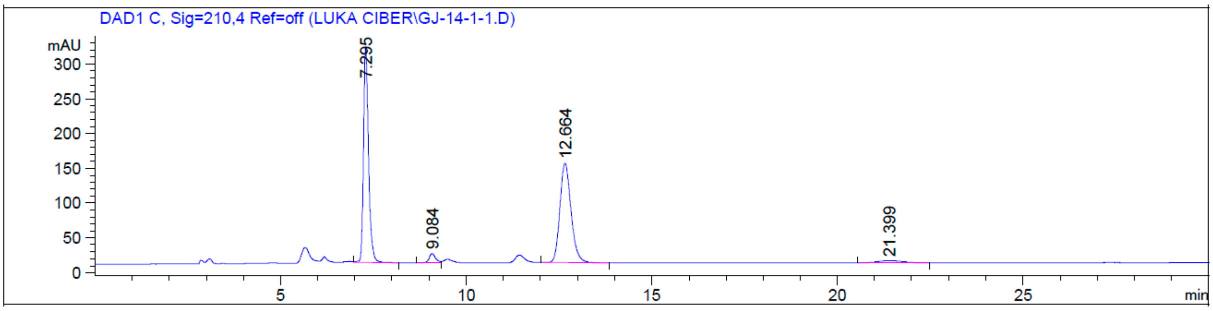

Signal 3: DAD1 C, Sig=210,4 Ref=off

Peak #	RetTime [min]	Type	Width [min]	Area [mAU*s]	Height [mAU]	Area %
1	7.295	VB	0.1421	2917.29712	309.24655	47.1321
2	9.084	BV	0.1769	157.09970	13.19718	2.5381
3	12.664	BB	0.3207	2979.21924	143.22690	48.1326
4	21.399	BB	0.5296	135.99629	3.10112	2.1972

Totals : 6189.61235 468.77176

Scheme S1, Entry 8

	Product	Catalyst	Conversion (%)	*ee* (%)
8	**17b**	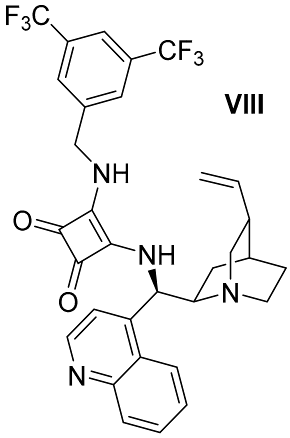 **VIII**	100<br>85% yield	95 (1*R*)<br>95 (1*R*)

HPLC: Chiralpak IA-3, *n*-Hexane/*i*-PrOH = 80:20, flow rate 1.0 mL/min, λ = 210 nm, T = 20°C.

Diastereomer 1: *t*R = 7.22 minutes (minor); 8.98 minutes (major) – 95% *ee*.

Diastereomer 2: *t*R = 12.53 minutes (minor); 20.94 minutes (major) – 95% *ee*.

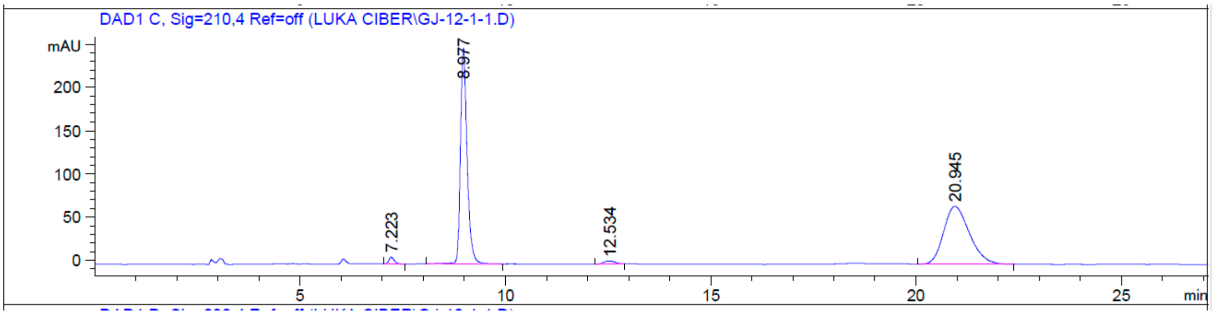

Signal 3: DAD1 C, Sig=210,4 Ref=off

Peak #	RetTime [min]	Type	Width [min]	Area [mAU*s]	Height [mAU]	Area %
1	7.223	BB	0.1374	70.62483	7.81546	1.2010
2	8.977	BB	0.1723	2828.10278	249.42270	48.0930
3	12.534	BB	0.2508	69.47431	3.60606	1.1814
4	20.945	BB	0.6480	2912.28662	66.88858	49.5246

Totals : 5880.48855 327.73280

Scheme S1, Entry 9

	Product	Catalyst	Conversion (%)	*ee* (%)
9	**17b**	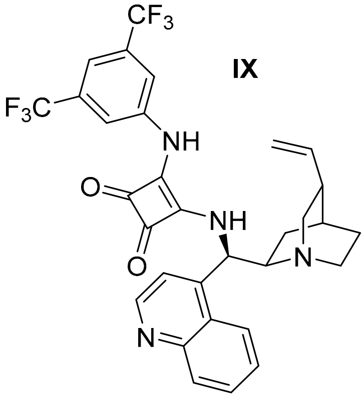 **IX**	100	85 (1*R*)<br>85 (1*R*)

HPLC: Chiralpak IA-3, *n*-Hexane/*i*-PrOH = 80:20, flow rate 1.0 mL/min, λ = 210 nm, T = 20°C.

Diastereomer 1: *t*R = 7.23 minutes (minor); 8.99 minutes (major) – 85% *ee*.

Diastereomer 2: *t*R = 12.52 minutes (minor); 20.97 minutes (major) – 85% *ee*.

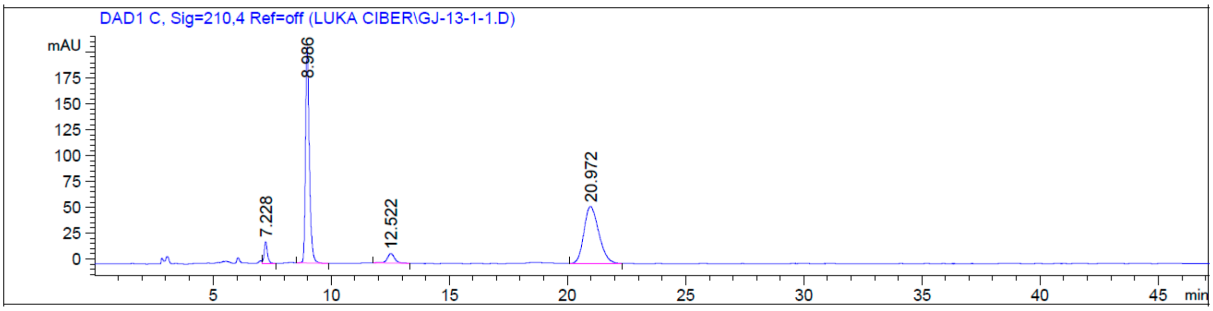

Signal 3: DAD1 C, Sig=210,4 Ref=off

Peak #	RetTime [min]	Type	Width [min]	Area [mAU*s]	Height [mAU]	Area %
1	7.228	VB	0.1437	196.51450	20.89279	3.8094
2	8.986	BB	0.1722	2360.12573	208.35469	45.7508
3	12.522	BB	0.3220	198.60716	9.12408	3.8500
4	20.972	BB	0.6512	2403.40845	55.06463	46.5898

Totals : 5158.65584 293.43619

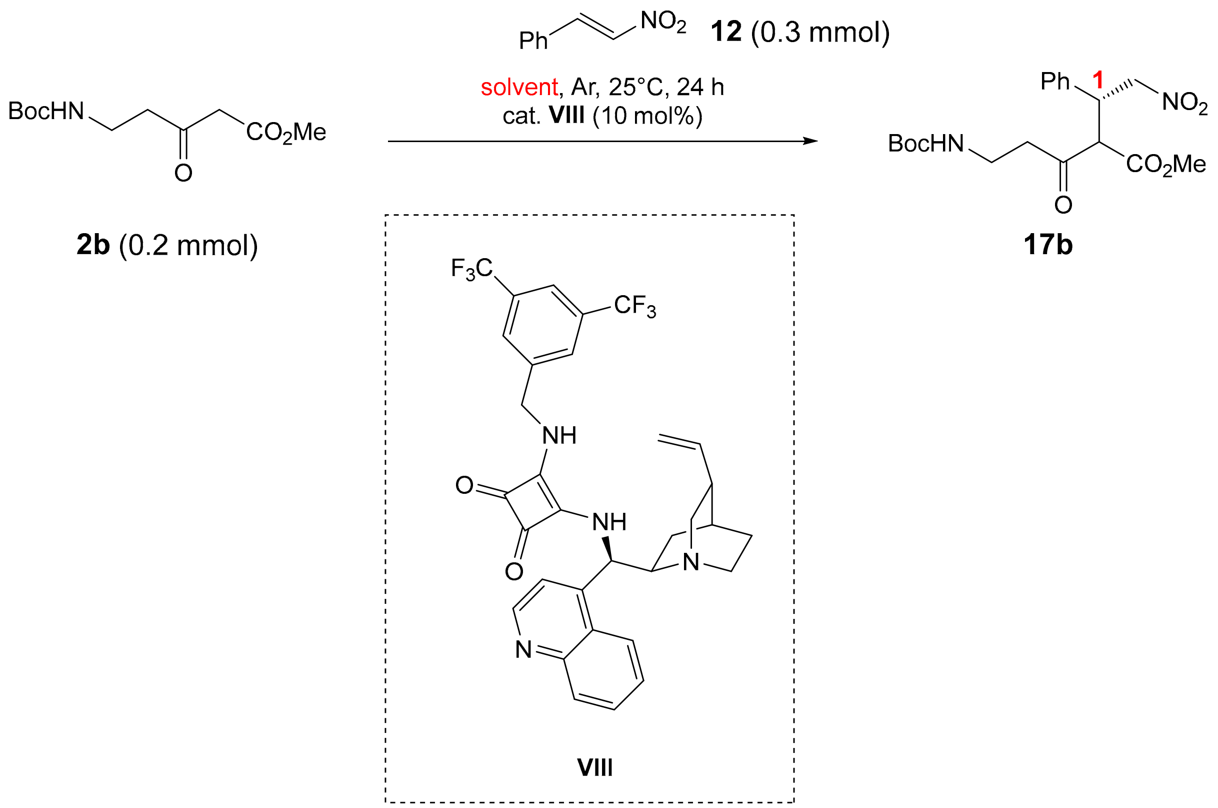

Scheme S2. Optimization of the reaction conditions – solvent.

Scheme S2, Entry 1

	Product	Catalyst/Solvent	Conversion (%)	*ee* (%)
1	**17b**	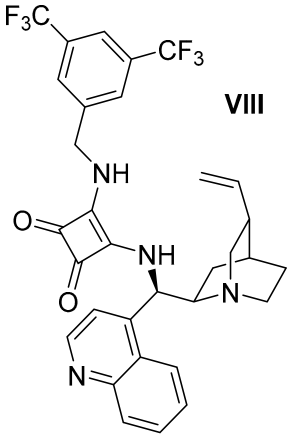 **VIII**<br>Toluene	97	95 (1*R*)<br>95 (1*R*)

HPLC: Chiralpak IA-3, *n*-Hexane/*i*-PrOH = 80:20, flow rate 1.0 mL/min, λ = 210 nm, T = 20°C.

Diastereomer 1: *t*R = 7.19 minutes (minor); 8.94 minutes (major) – 95% *ee*.

Diastereomer 2: *t*R = 12.46 minutes (minor); 20.90 minutes (major) – 95% *ee*.

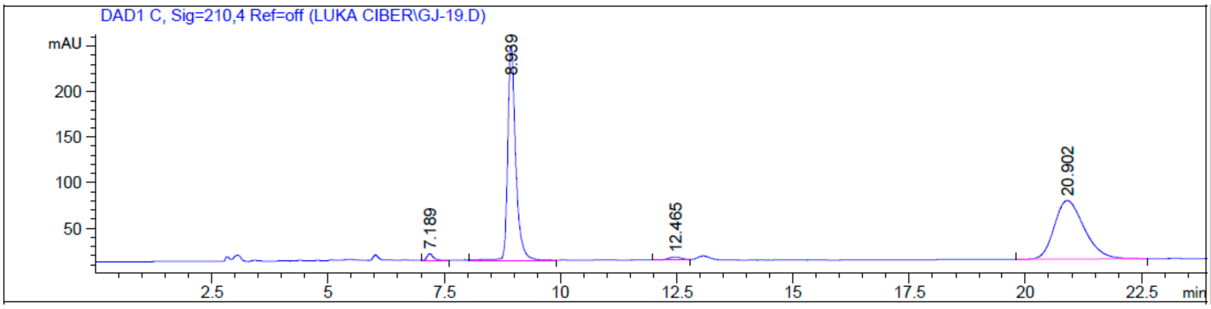

Signal 3: DAD1 C, Sig=210,4 Ref=off

Peak #	RetTime [min]	Type	Width [min]	Area [mAU*s]	Height [mAU]	Area %
1	7.189	BB	0.1386	64.37454	7.04663	1.1139
2	8.939	BB	0.1773	2778.42285	236.14130	48.0754
3	12.465	BV	0.2857	64.68170	3.20171	1.1192
4	20.902	BB	0.6850	2871.81763	64.30836	49.6915

Totals :				5779.29672	310.69800	

Scheme S2, Entry 2

	Product	Catalyst/Solvent	Conversion (%)	*ee* (%)
2	**17b**	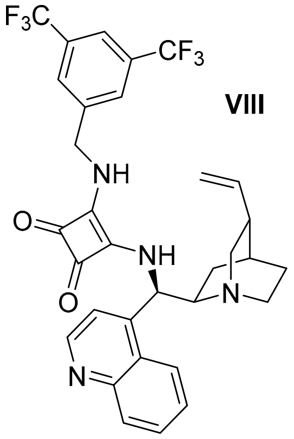**VIII**<br>THF	100	96 (1*R*)<br>96 (1*R*)

HPLC: Chiralpak IA-3, *n*-Hexane/*i*-PrOH = 80:20, flow rate 1.0 mL/min, λ = 210 nm, T = 20°C.

Diastereomer 1: *t*R = 7.19 minutes (minor); 8.94 minutes (major) – 96% *ee*.

Diastereomer 2: *t*R = 12.46 minutes (minor); 20.90 minutes (major) – 96% *ee*.

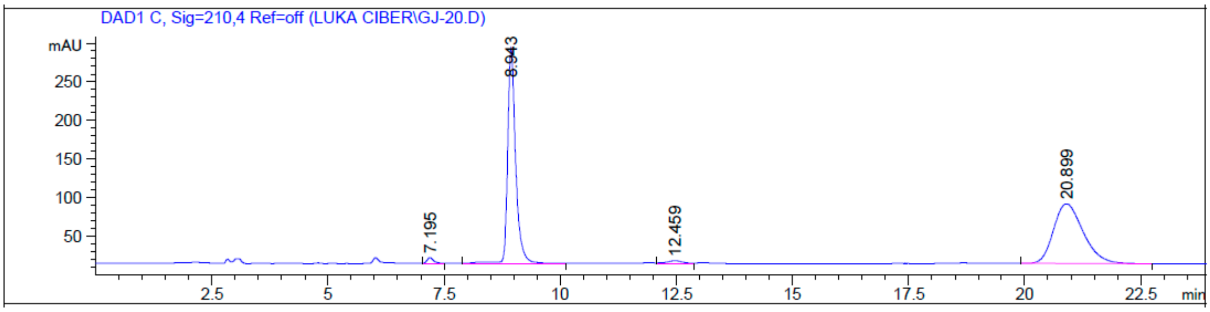

Signal 3: DAD1 C, Sig=210,4 Ref=off

Peak #	RetTime [min]	Type	Width [min]	Area [mAU*s]	Height [mAU]	Area %
1	7.195	VB	0.1385	67.69527	7.41675	0.9817
2	8.943	BB	0.1786	3318.40430	279.36002	48.1234
3	12.459	BV	0.2444	64.02470	3.29994	0.9285
4	20.899	BB	0.6912	3445.48511	77.42260	49.9664

Totals : 6895.60938 367.49931

Scheme S2, Entry 3

	Product	Catalyst/Solvent	Conversion (%)	*ee* (%)
4	**17b**	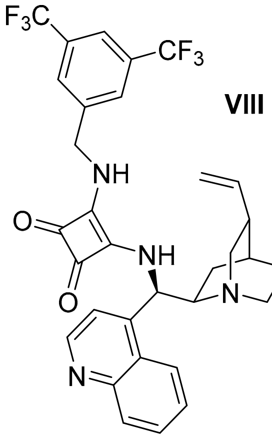 EtOAc	99	95 (1*R*)<br>96 (1*R*)

HPLC: Chiralpak IA-3, *n*-Hexane/*i*-PrOH = 80:20, flow rate 1.0 mL/min, λ = 210 nm, T = 20°C.

Diastereomer 1: *t*R = 7.22 minutes (minor); 8.97 minutes (major) – 95% *ee*.

Diastereomer 2: *t*R = 12.50 minutes (minor); 20.95 minutes (major) – 96% *ee*.

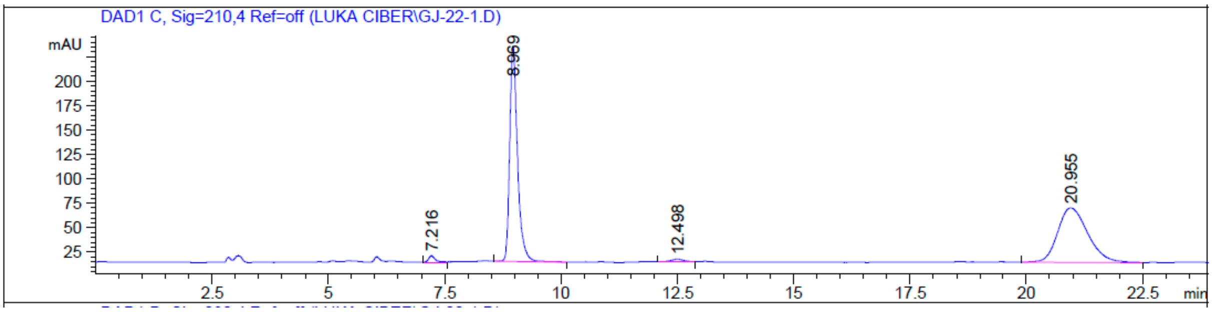

Signal 3: DAD1 C, Sig=210,4 Ref=off

Peak #	RetTime [min]	Type	Width [min]	Area [mAU*s]	Height [mAU]	Area %
1	7.216	BB	0.1366	57.89161	6.45851	1.1188
2	8.969	BB	0.1760	2569.51587	220.34718	49.6574
3	12.498	BB	0.2730	51.26400	2.68168	0.9907
4	20.955	BB	0.6828	2495.81201	55.69257	48.2331
Totals :				5174.48349	285.17995	

Scheme S2, Entry 4

	Product	Catalyst/Solvent	Conversion (%)	*ee* (%)
4	**17b**	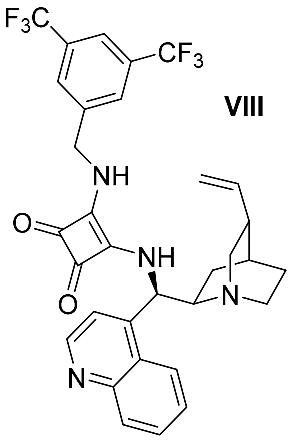 MeCN	100	94 (1*R*)<br>94 (1*R*)

HPLC: Chiralpak IA-3, *n*-Hexane/*i*-PrOH = 80:20, flow rate 1.0 mL/min, λ = 210 nm, T = 20°C.

Diastereomer 1: *t*R = 7.20 minutes (minor); 8.95 minutes (major) – 94% *ee*.

Diastereomer 2: *t*R = 12.47 minutes (minor); 20.94 minutes (major) – 94% *ee*.

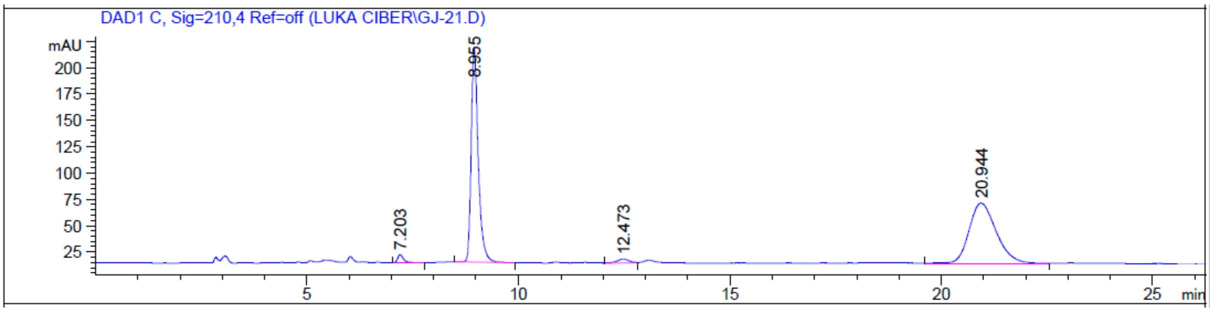

Signal 3: DAD1 C, Sig=210,4 Ref=off

Peak #	RetTime [min]	Type	Width [min]	Area [mAU*s]	Height [mAU]	Area %
1	7.203	BB	0.1424	72.50190	7.66451	1.4384
2	8.955	BB	0.1732	2364.25293	204.03456	46.9052
3	12.473	BV	0.2889	73.30263	3.64048	1.4543
4	20.944	BB	0.6813	2530.43237	57.07153	50.2021

Totals : 5040.48983 272.41107

Scheme S2, Entry 5

	Product	Catalyst/Solvent	Conversion (%)	*ee* (%)
5	**17b**	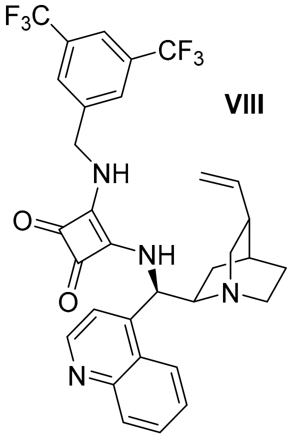 **VIII** MeOH	100	83 (1*R*)<br>81 (1*R*)

HPLC: Chiralpak IA-3, *n*-Hexane/*i*-PrOH = 80:20, flow rate 1.0 mL/min, λ = 210 nm, T = 20°C.

Diastereomer 1: *t*R = 7.21 minutes (minor); 8.97 minutes (major) – 83% *ee*.

Diastereomer 2: *t*R = 12.48 minutes (minor); 20.95 minutes (major) – 81% *ee*.

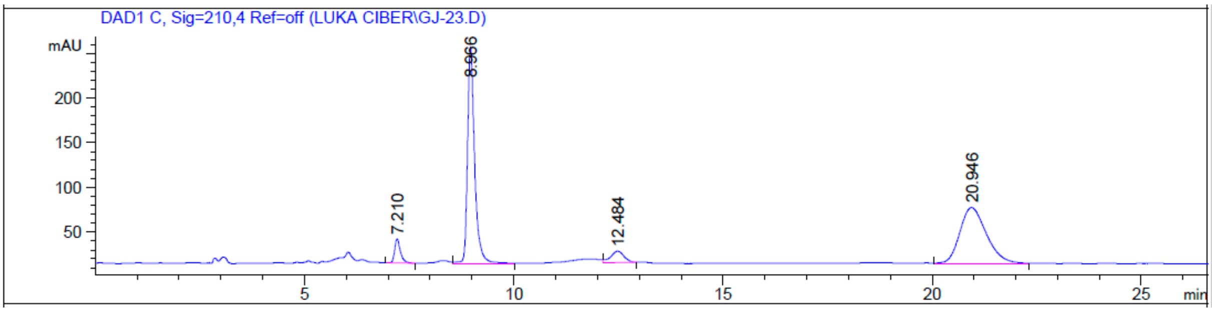

Signal 3: DAD1 C, Sig=210,4 Ref=off

Peak #	RetTime [min]	Type	Width [min]	Area [mAU*s]	Height [mAU]	Area %
1	7.210	BB	0.1429	253.75653	26.68423	4.1582
2	8.966	VB	0.1751	2837.87549	241.56335	46.5032
3	12.484	VB	0.3237	282.07779	12.67422	4.6223
4	20.946	BB	0.6475	2728.82202	62.23940	44.7162

Totals : 6102.53183 343.16121

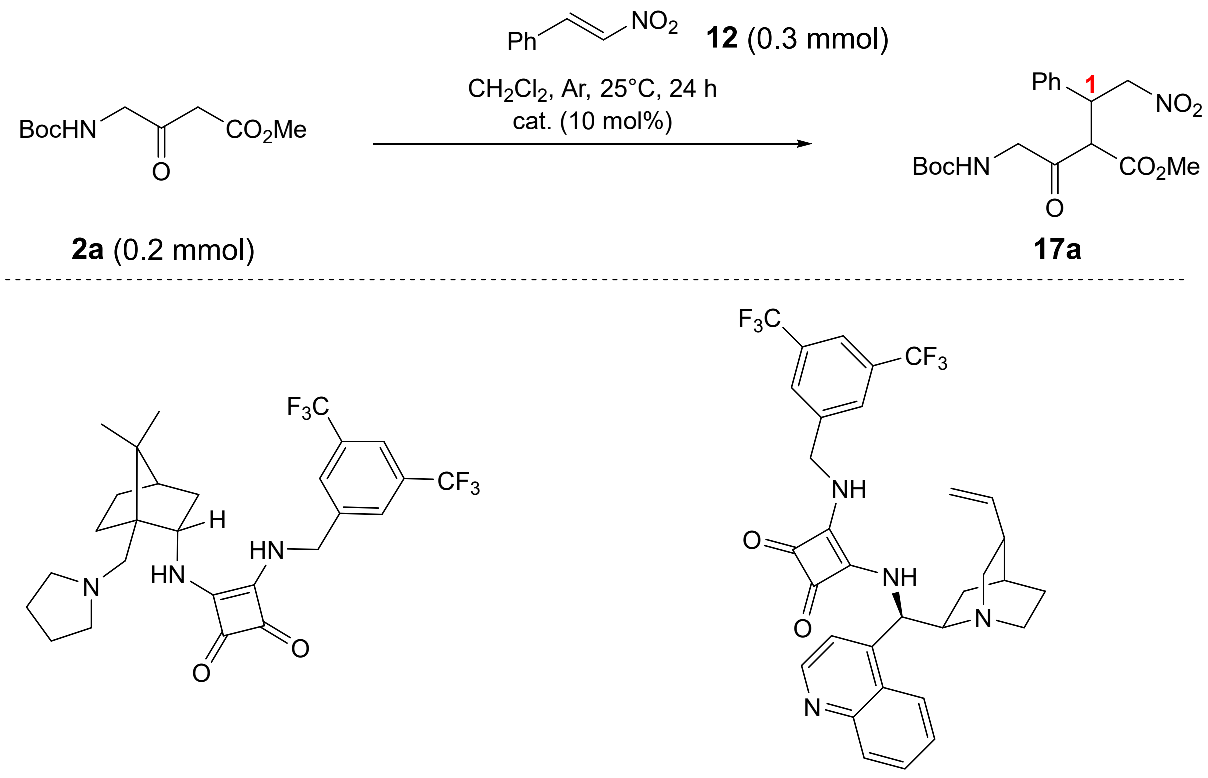

I; 82% yield; **X-Ray**
1. 95% *ee* (1*S*)
2. 94% *ee* (1*S*)

VIII; 84% yield
1. 91% *ee* (1*R*)
2. 93% *ee* (1*R*)

Scheme S3. Evaluation of organocatalysts **I** and **VIII**.

HPLC: Chiralpak AS-H, *n*-Hexane/*i*-PrOH = 80:20, flow rate 1.0 mL/min, λ = 210 nm, T = 20°C.

Diastereomer 1: *t*R = 9.26 minutes; 17.72 minutes.

Diastereomer 2: *t*R = 14.21 minutes; 23.62 minutes.

Racemate:

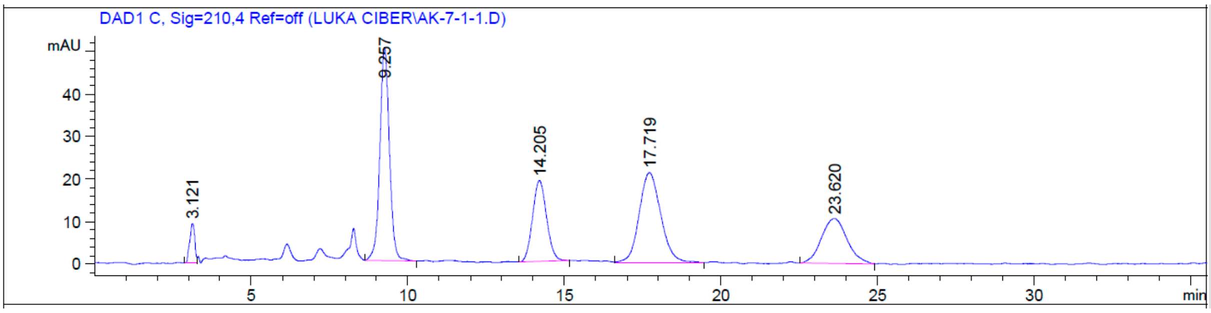

Signal 3: DAD1 C, Sig=210,4 Ref=off

Peak #	RetTime [min]	Type	Width [min]	Area [mAU*s]	Height [mAU]	Area %
1	3.121	BV	0.1682	103.92841	9.24039	3.0862
2	9.257	BB	0.3237	1063.60010	50.10559	31.5842
3	14.205	BB	0.4439	589.74133	19.01176	17.5127
4	17.719	BB	0.6160	1006.64203	21.12216	29.8928
5	23.620	BB	0.6935	603.59320	10.54948	17.9240
Totals :				3367.50507	110.02937	

Scheme S3, Entry 1

	Product	Catalyst	Yield (%)	*ee* (%)
1	**17a**	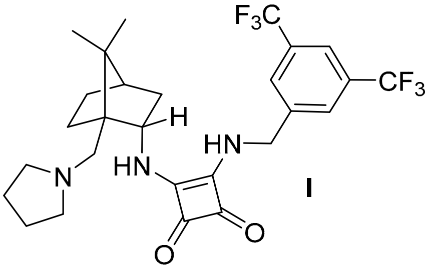 **I**	82<br>Y-Ray	95 (1*S*)<br>94 (1*S*)

HPLC: Chiralpak AS-H, *n*-Hexane/*i*-PrOH = 80:20, flow rate 1.0 mL/min, λ = 210 nm, T = 20°C.

Diastereomer 1: *t*R = 9.27 minutes (major); 17.63 minutes (minor) – 95% *ee*.

Diastereomer 2: *t*R = 14.24 minutes (minor); 23.56 minutes (major) – 94% *ee*.

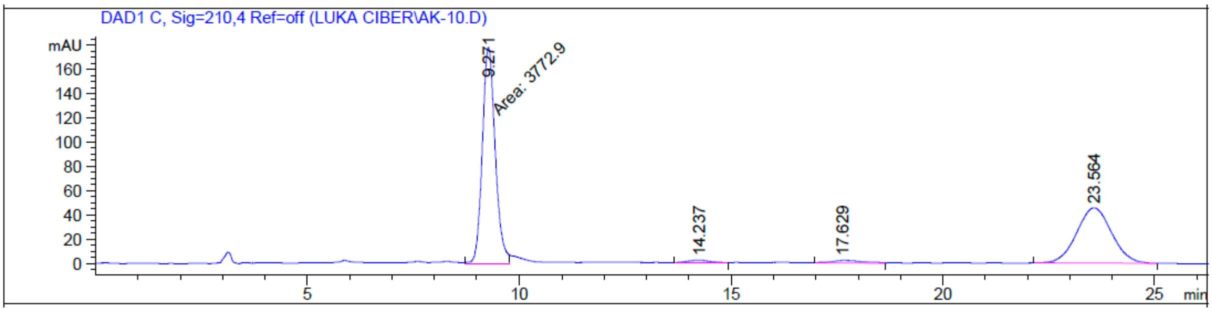

Signal 3: DAD1 C, Sig=210,4 Ref=off

Peak #	RetTime [min]	Type	Width [min]	Area [mAU*s]	Height [mAU]	Area %
1	9.271	MM	0.3524	3772.89551	178.44685	57.5702
2	14.237	BB	0.4179	75.63523	2.17402	1.1541
3	17.629	BB	0.5830	97.76601	1.98380	1.4918
4	23.564	BB	0.8074	2607.25513	45.57800	39.7838

Totals : 6553.55188 228.18268

Scheme S3, Entry 2

	Product	Catalyst	Yield (%)	*ee* (%)
2	**17a**	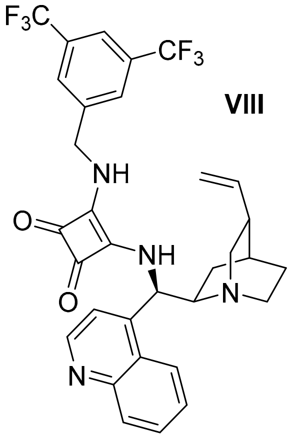	84	91 (1*R*)<br>93 (1*R*)

HPLC: Chiralpak IA-3, *n*-Hexane/*i*-PrOH = 80:20, flow rate 1.0 mL/min, λ = 210 nm, T = 20°C.

Diastereomer 1: *t*R = 9.24 minutes (minor); 17.62 minutes (major) – 91% *ee*.

Diastereomer 2: *t*R = 14.12 minutes (major); 23.56 minutes (minor) – 93% *ee*.

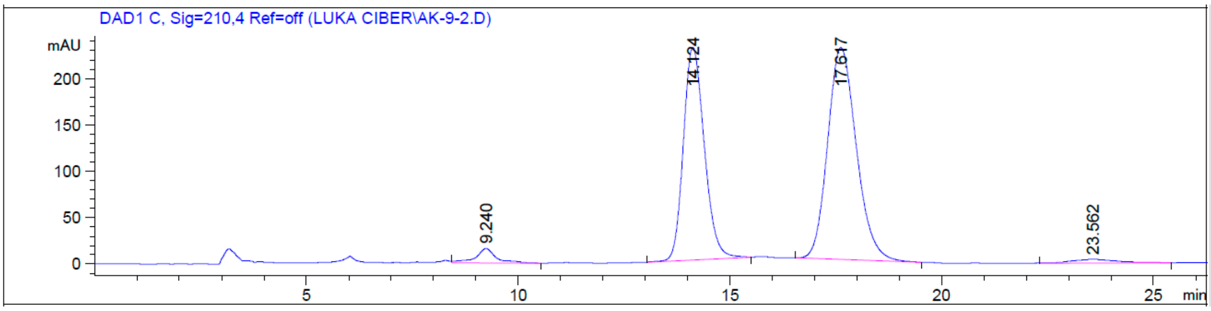

Signal 3: DAD1 C, Sig=210,4 Ref=off

Peak #	RetTime [min]	Type	Width [min]	Area [mAU*s]	Height [mAU]	Area %
1	9.240	VB	0.4635	507.83667	15.36711	2.6143
2	14.124	BB	0.5327	7865.40479	229.02592	40.4900
3	17.617	BB	0.7274	1.07720e4	228.33435	55.4528
4	23.562	BB	0.8649	280.29608	3.81196	1.4429

Totals : 1.94256e4 476.53934

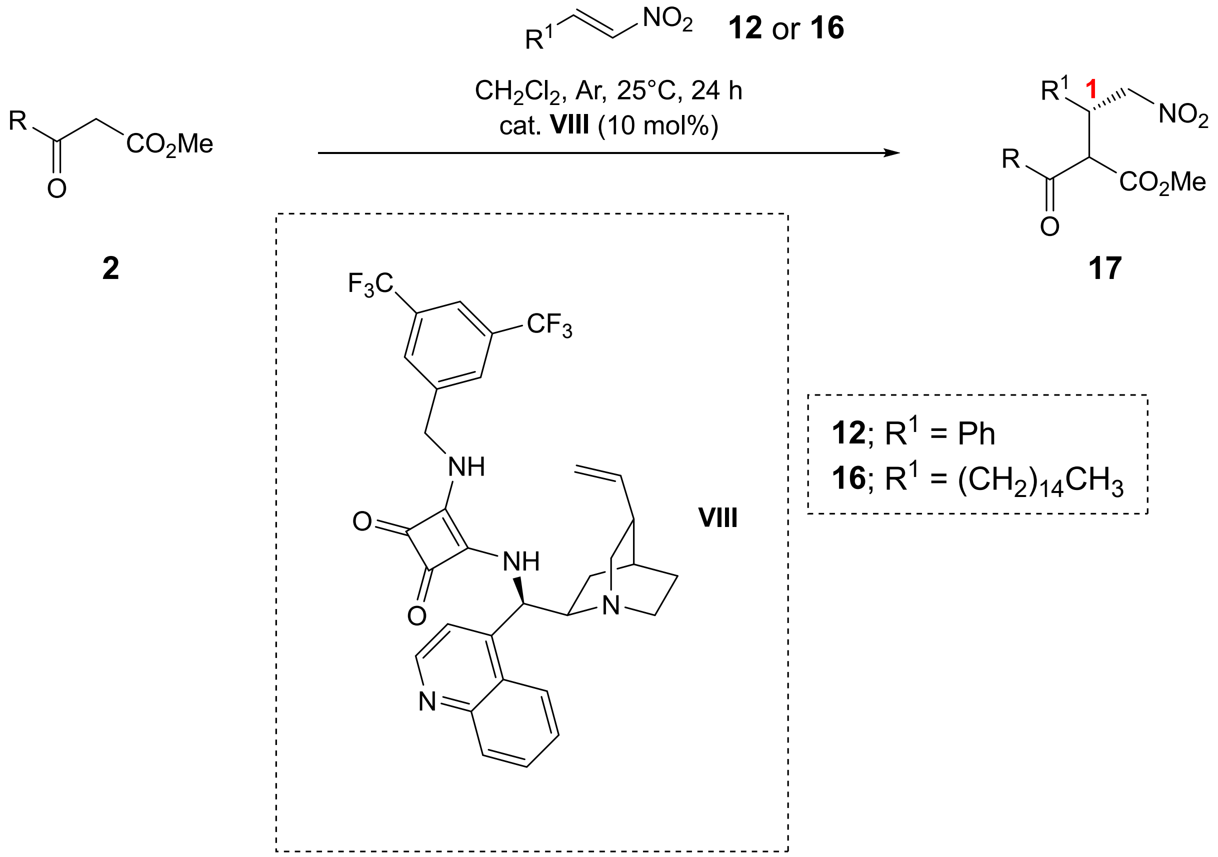

Scheme S4. Scope of the organocatalyzed addition of β-keto esters **2** to *trans*-β-nitrostyrene (**12**) and fatty acid-derived nitroalkene **16**.

Scheme S4, Entry 1

	Product	Yield (%)	*ee* (%)
1	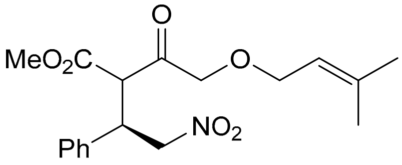 **17d**	89	94 (1*R*)<br>91 (1*R*)

HPLC: Chiralpak AS-H, *n*-Hexane/EtOH = 90:10, flow rate 1.0 mL/min, λ = 210 nm, T = 20 °C.

Racemate:

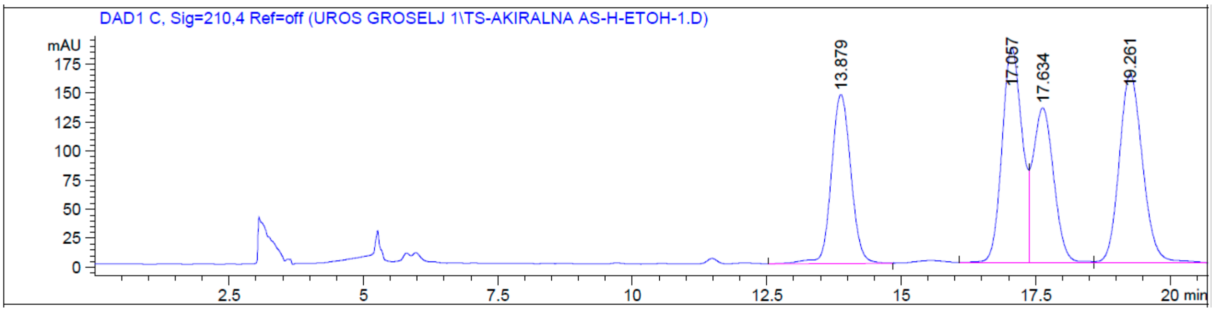

Signal 3: DAD1 C, Sig=210,4 Ref=off

Peak #	RetTime [min]	Type	Width [min]	Area [mAU*s]	Height [mAU]	Area %
1	13.879	BB	0.3992	3676.67236	145.28801	21.6276
2	17.057	VV	0.3937	4794.20410	185.36975	28.2013
3	17.634	VV	0.4126	3620.07520	133.41176	21.2946
4	19.261	VBA	0.4686	4908.97803	162.44025	28.8765

Organocatalyzed asymmetric reaction: Minor diastereomer: enantiomers: *t*R = 14.066 minutes (minor); 17.773 minutes (major) – 94% *ee*. Major diastereomer: enantiomers: *t*R = 17.164 minutes (major); 19.406 minutes (minor) – 91% *ee*.

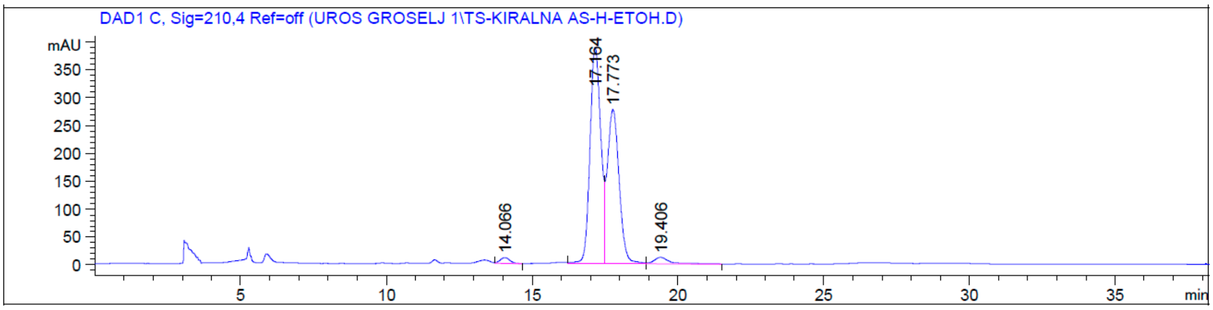

Signal 3: DAD1 C, Sig=210,4 Ref=off

Peak #	RetTime [min]	Type	Width [min]	Area [mAU*s]	Height [mAU]	Area %
1	14.066	VB	0.3637	248.93478	10.68889	1.3459
2	17.164	VV	0.3983	1.00548e4	388.07782	54.3639
3	17.773	VV	0.4255	7725.95313	276.89282	41.7724
4	19.406	VB	0.5685	465.68393	11.90033	2.5178

Totals : 1.84954e4 687.55986

Scheme S4, Entry 2

	Product	Yield (%)	*ee* (%)
2	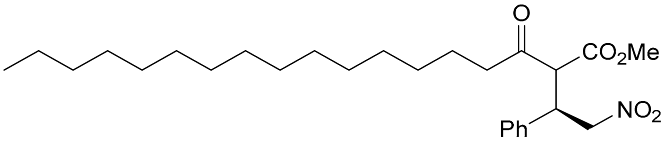 **17f**	60	95 (1*R*)<br>96 (1*R*)

HPLC: Chiralpak IA-3, *n*-Hexane/EtOH = 95:5, flow rate 1.0 mL/min, λ = 210 nm, T = 25 °C.

Racemate:

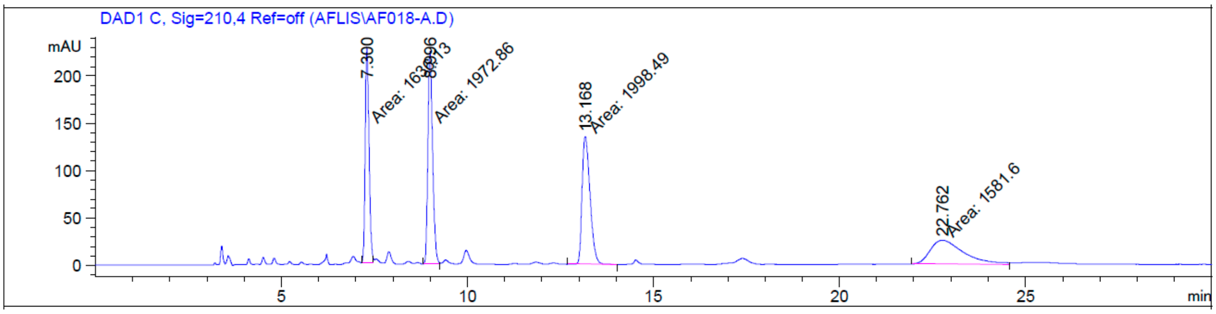

Signal 3: DAD1 C, Sig=210,4 Ref=off

Peak #	RetTime [min]	Type	Width [min]	Area [mAU*s]	Height [mAU]	Area %
1	7.300	MM	0.1196	1636.12561	228.02846	22.7585
2	8.996	MM	0.1461	1972.85608	225.03249	27.4424
3	13.168	MM	0.2470	1998.48853	134.87402	27.7990
4	22.762	MM	1.0535	1581.60327	25.02245	22.0001

Organocatalyzed asymmetric reaction: Minor diastereomer: enantiomers: *t*R = 7.287 minutes (minor); 22.511 minutes (major) – 95% *ee*. Major diastereomer: enantiomers: *t*R = 8.963 minutes (major); 13.215 minutes (minor) – 96% *ee*.

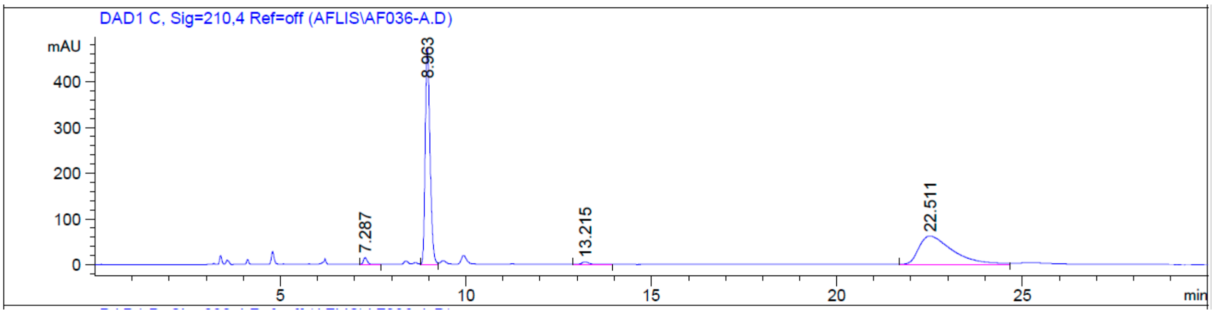

Signal 3: DAD1 C, Sig=210,4 Ref=off

Peak #	RetTime [min]	Type	Width [min]	Area [mAU*s]	Height [mAU]	Area %
1	7.287	VB	0.1160	109.42399	14.50895	1.3175
2	8.963	BV	0.1406	4240.29150	472.81989	51.0547
3	13.215	BB	0.2264	75.77318	5.18918	0.9123
4	22.511	BV	0.9109	3879.90015	61.59085	46.7155

Scheme S4, Entry 3

	**Product**		**Yield (%)**	***ee* (%)**
3	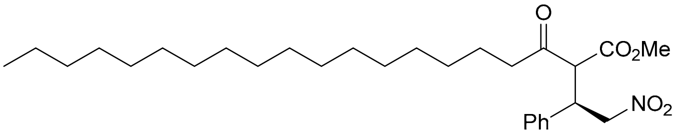	**17h**	54	96 (1*R*)<br>96 (1*R*)

HPLC: Chiralpak IA-3, *n*-Hexane/EtOH = 95:5, flow rate 1.0 mL/min, λ = 210 nm, T = 25 °C.

Racemate:

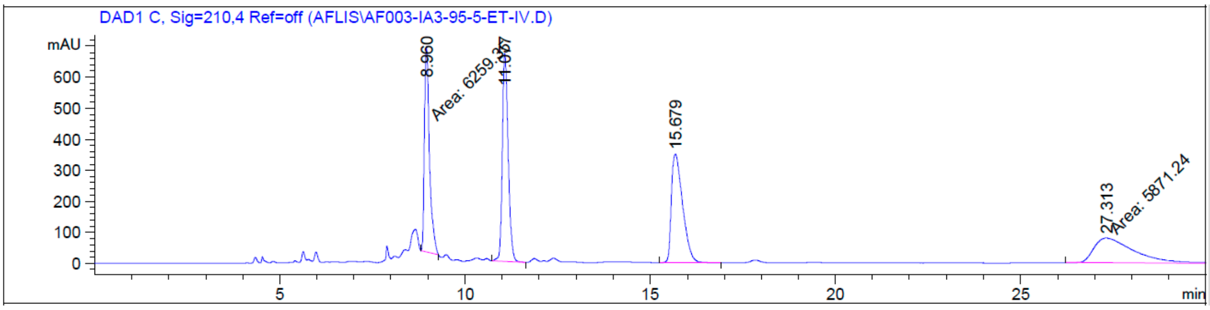

Signal 3: DAD1 C, Sig=210,4 Ref=off

Peak #	RetTime [min]	Type	Width [min]	Area [mAU*s]	Height [mAU]	Area %
1	8.960	MM	0.1574	6259.34912	662.62158	23.2644
2	11.077	BB	0.1703	7359.99902	669.15198	27.3553
3	15.679	BB	0.3272	7414.62158	349.98291	27.5583
4	27.313	MM	1.2371	5871.24072	79.09811	21.8219

Organocatalyzed asymmetric reaction: Major diastereomer: enantiomers: *t*R = 8.838 minutes (minor); 26.885 minutes (major) – 96% *ee*. Minor diastereomer: enantiomers: *t*R = 10.913 minutes (major); 15.371 minutes (minor) – 96% *ee*.

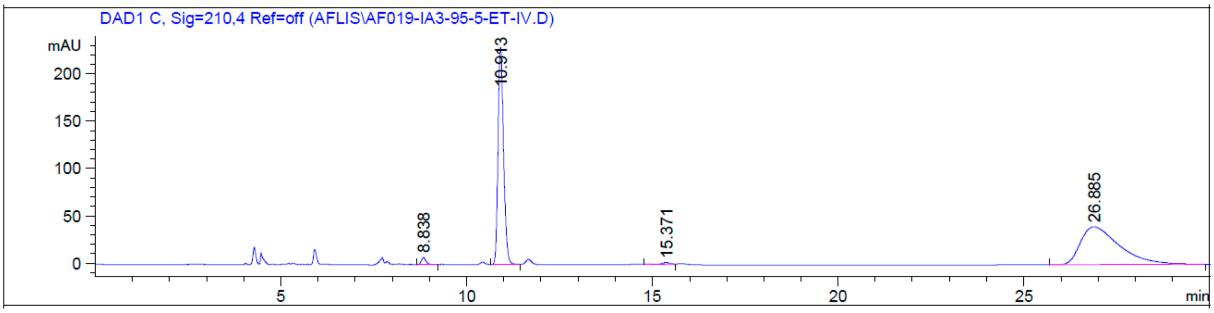

Signal 3: DAD1 C, Sig=210,4 Ref=off

Peak #	RetTime [min]	Type	Width [min]	Area [mAU*s]	Height [mAU]	Area %
1	8.838	BV	0.1358	66.95542	7.52462	1.2324
2	10.913	VV	0.1590	2378.42383	229.32558	43.7769
3	15.371	BV	0.2734	39.89277	2.04711	0.7343
4	26.885	BBA	1.0811	2947.77979	40.03289	54.2564

Scheme S4, Entry 4

	**Product**	**Yield (%)**	***ee* (%)**
4	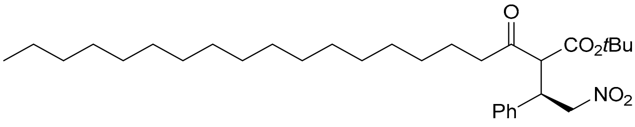 **17j**	47	93 (1*R*)<br>98 (1*R*)

HPLC: Chiralpak IA-3, *n*-Hexane/EtOH = 95:5, flow rate 1.0 mL/min, λ = 210 nm, T = 25 °C.

Racemate:

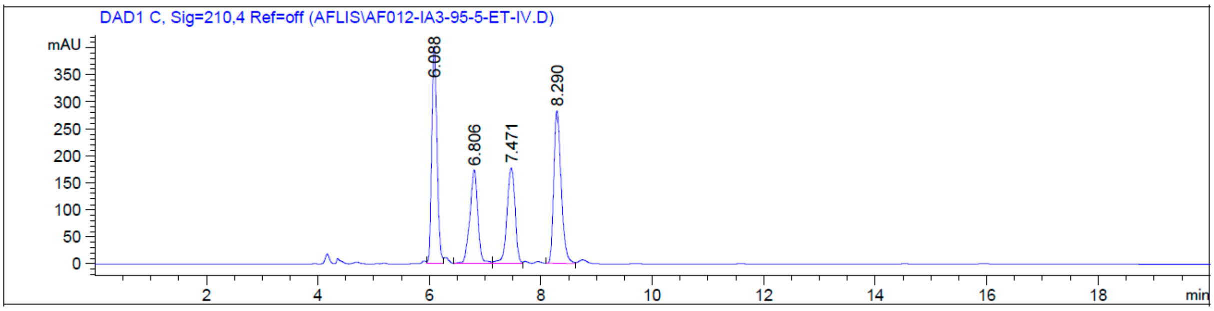

Signal 3: DAD1 C, Sig=210,4 Ref=off

Peak #	RetTime [min]	Type	Width [min]	Area [mAU*s]	Height [mAU]	Area %
1	6.088	VV	0.1032	2655.45020	401.14902	30.3023
2	6.806	BV	0.1458	1713.56238	172.66795	19.5541
3	7.471	VV	0.1639	1786.19922	176.76180	20.3830
4	8.290	BV	0.1418	2607.97266	282.26428	29.7606

Totals : 8763.18445 1032.84305

Organocatalyzed asymmetric reaction: Minor diastereomer: enantiomers: *t*R = 6.051 minutes (minor); 8.241 minutes (major) – 93% *ee*. Major diastereomer: enantiomers: *t*R = 6.768 minutes (minor); 7.403 minutes (major) – 98% *ee*.

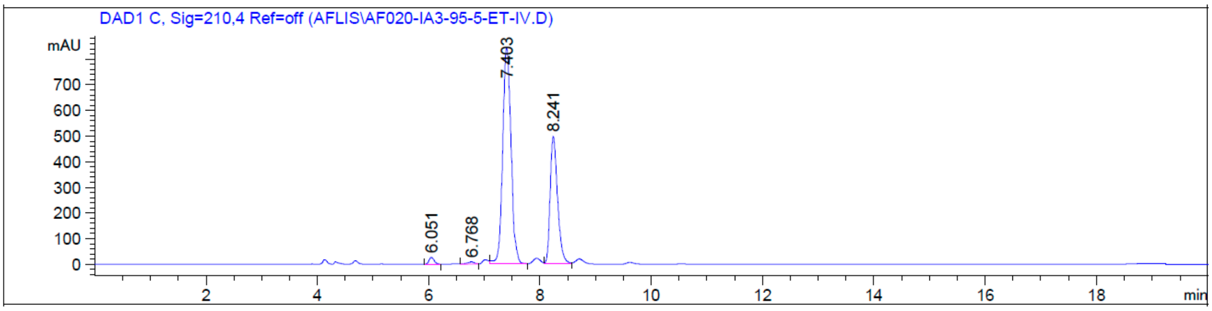

Signal 3: DAD1 C, Sig=210,4 Ref=off

Peak #	RetTime [min]	Type	Width [min]	Area [mAU*s]	Height [mAU]	Area %
1	6.051	VV	0.1011	174.75386	27.13556	1.2785
2	6.768	VB	0.1336	71.98055	8.26838	0.5266
3	7.403	VB	0.1617	8800.66406	843.37244	64.3868
4	8.241	VV	0.1427	4621.03906	495.96112	33.8081

Totals : 1.36684e4 1374.73750

Scheme S4, Entry 5

	Product	Yield (%)	*ee* (%)
5	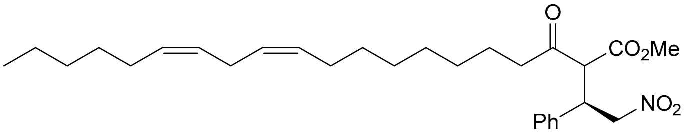 **17l**	62	96 (1*R*)<br>96 (1*R*)

HPLC: Chiralpak IA-3, *n*-Hexane/EtOH = 95:5, flow rate 1.0 mL/min, λ = 210 nm, T = 25 °C.

Racemate:

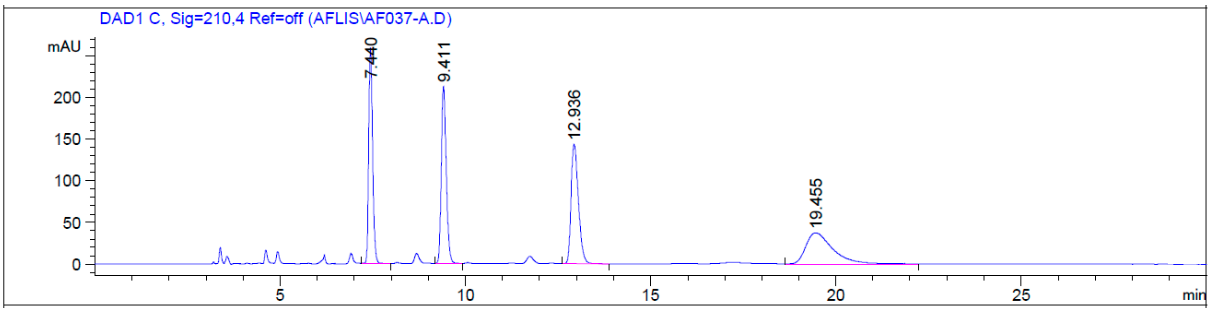

Signal 3: DAD1 C, Sig=210,4 Ref=off

Peak #	RetTime [min]	Type	Width [min]	Area [mAU*s]	Height [mAU]	Area %
1	7.440	VB	0.1141	1902.13721	257.65854	24.6543
2	9.411	VV	0.1405	1974.02234	212.20923	25.5861
3	12.936	VB	0.2133	1976.79773	142.97377	25.6220
4	19.455	BB	0.7472	1862.26575	37.25659	24.1375

Organocatalyzed asymmetric reaction: Minor diastereomer: enantiomers: *t*R = 7.439 minutes (minor); 19.360 minutes (major) – 96% *ee*. Major diastereomer: enantiomers: *t*R = 9.402 minutes (major); 12.992 minutes (minor) – 96% *ee*.

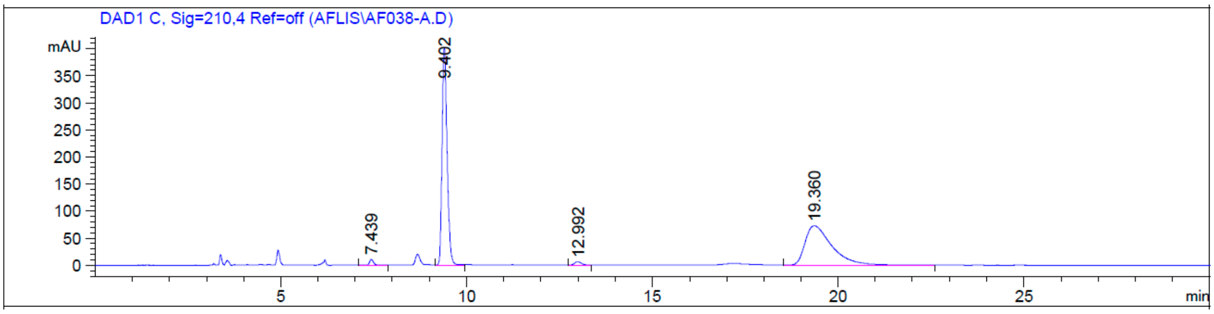

Signal 3: DAD1 C, Sig=210,4 Ref=off

Peak #	RetTime [min]	Type	Width [min]	Area [mAU*s]	Height [mAU]	Area %
1	7.439	BB	0.1156	80.83439	10.76048	1.0679
2	9.402	BV	0.1460	3776.07324	400.33188	49.8870
3	12.992	BB	0.1972	80.25330	6.27429	1.0603
4	19.360	BB	0.7507	3632.09839	72.72542	47.9849

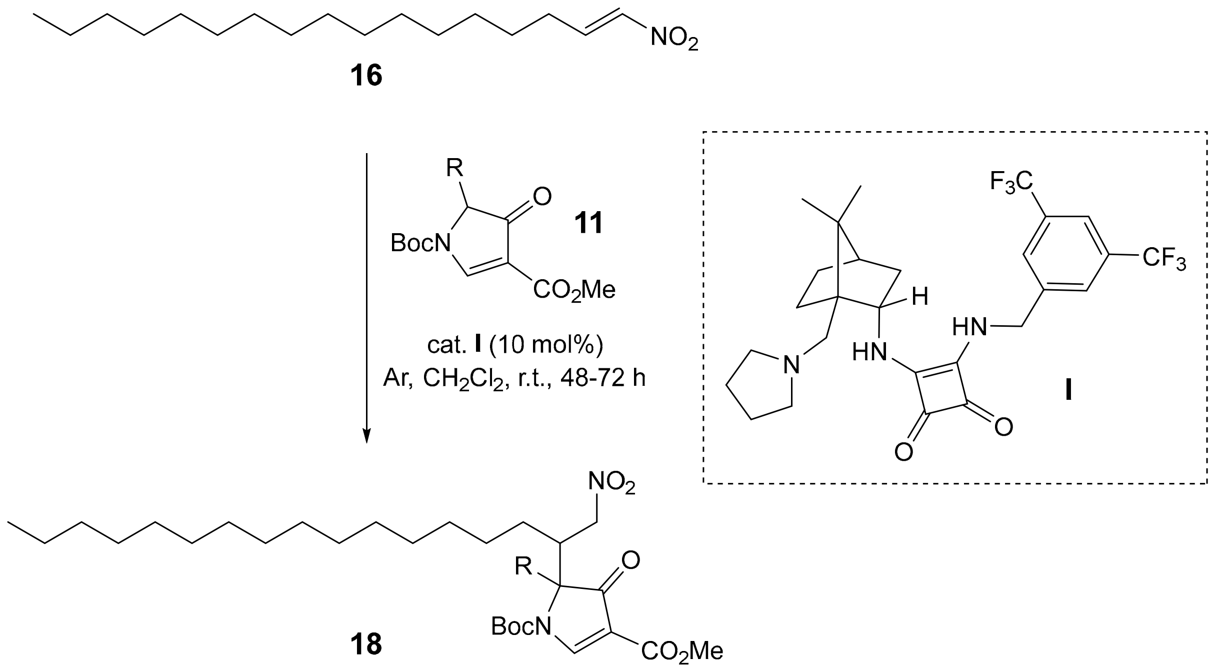

Scheme S5. Scope of the organocatalyzed addition of pyrrolones **11** to fatty acid-derived nitroalkene **16**.

Scheme S5, Entry 1

	Product	Yield (%)	*ee* (%)
1	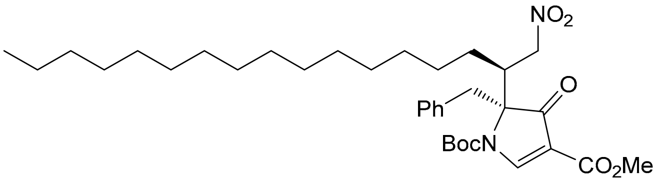 **18a**	69	82

HPLC: Chiralpak IA-3, *n*-Hexane/*i*PrOH = 95:5, flow rate 1.0 mL/min, λ = 210 nm, T = 25 °C.

Racemate:

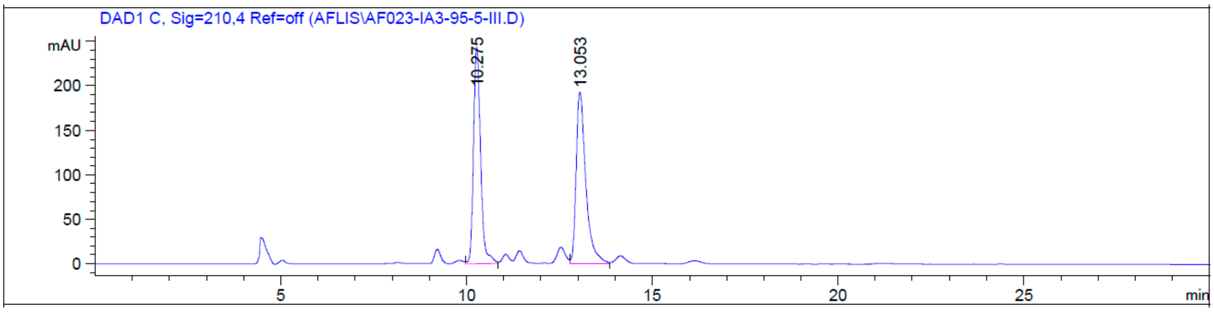

Signal 3: DAD1 C, Sig=210,4 Ref=off

Peak #	RetTime [min]	Type	Width [min]	Area [mAU*s]	Height [mAU]	Area %
1	10.275	VV	0.2049	3228.73438	243.15233	48.0784
2	13.053	VV	0.2734	3486.83252	192.46977	51.9216

Organocatalyzed asymmetric reaction: Major diastereomer: enantiomers: *t*R = 9.350 minutes (major); 13.217 minutes (minor) – 82% *ee*.

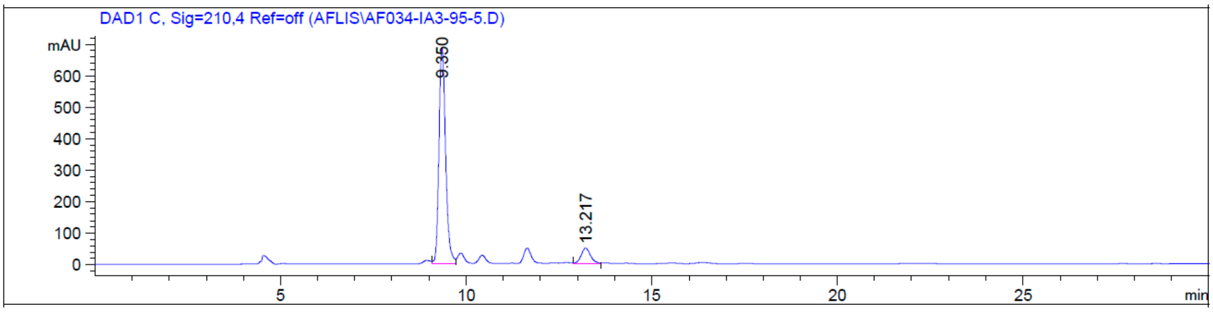

Signal 3: DAD1 C, Sig=210,4 Ref=off

Peak #	RetTime [min]	Type	Width [min]	Area [mAU*s]	Height [mAU]	Area %
1	9.350	VV	0.1929	8526.79883	686.70966	90.7664
2	13.217	VV	0.2708	867.42902	48.94731	9.2336

Scheme S5, Entry 2

	Product	Yield (%)	*ee* (%)
2	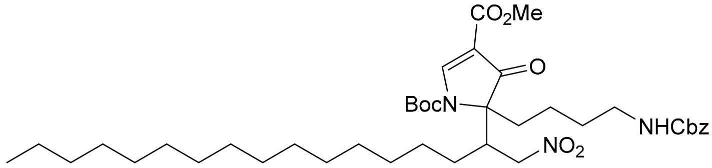 **18b**	18	57<br>11

HPLC: Chiralpak IA-3, *n*-Hexane/*i*PrOH = 90:10, flow rate 1.0 mL/min, λ = 210 nm, T = 25 °C.

Racemate:

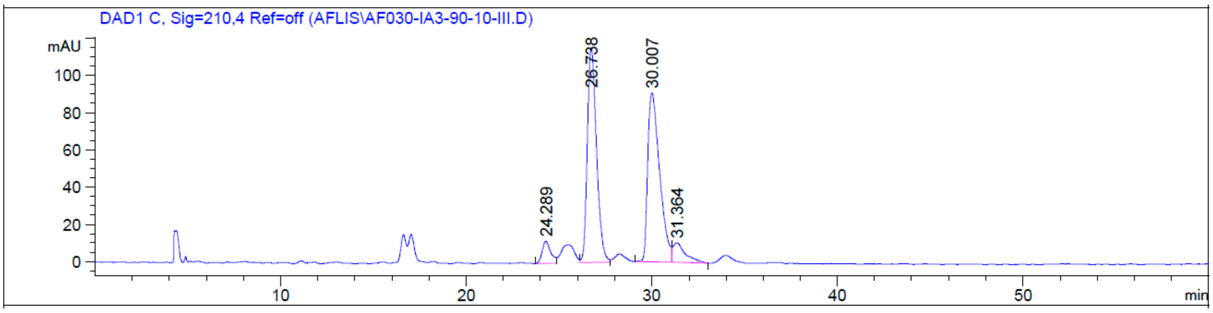

Signal 3: DAD1 C, Sig=210,4 Ref=off

Peak #	RetTime [min]	Type	Width [min]	Area [mAU*s]	Height [mAU]	Area %
1	24.289	BV	0.4889	382.68256	11.84530	4.3130
2	26.738	VV	0.5276	3944.49072	115.17294	44.4558
3	30.007	BV	0.6801	4043.98096	90.71446	45.5771
4	31.364	VB	0.6598	501.68494	10.53015	5.6542

Organocatalyzed asymmetric reaction: Major diastereomer: enantiomers: *t*R = 24.088 minutes (minor); 31.095 minutes (major) – 57% *ee*. Minor diastereomer: enantiomers: *t*R = 26.634 minutes (minor); 30.099 minutes (major) – 11% *ee*.

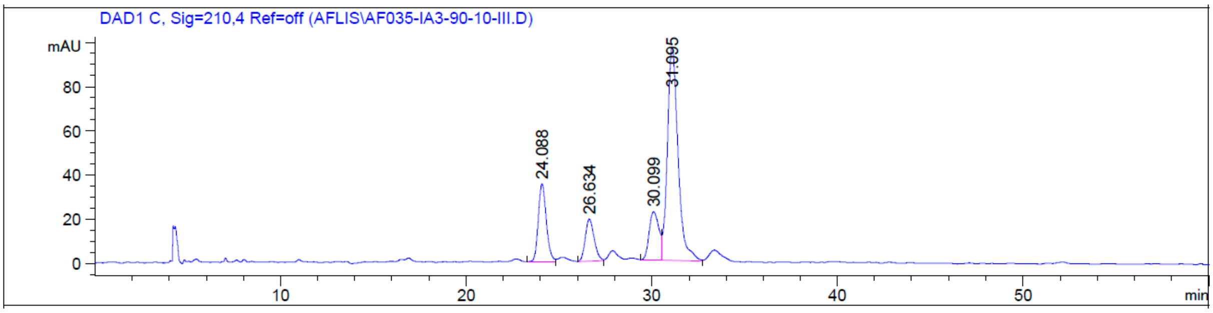

Signal 3: DAD1 C, Sig=210,4 Ref=off

Peak #	RetTime [min]	Type	Width [min]	Area [mAU*s]	Height [mAU]	Area %
1	24.088	BV	0.4658	1057.09045	35.25305	16.6475
2	26.634	BV	0.5062	635.07788	18.98726	10.0015
3	30.099	BV	0.5688	797.31628	21.89840	12.5565
4	31.095	VV	0.6078	3860.34375	96.27203	60.7945

Scheme S5, Entry 3

	Product	Yield (%)	*ee* (%)
3	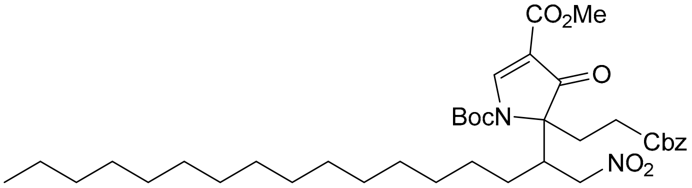 **18c**	31	68<br>38

HPLC: Chiralpak IA-3, *n*-Hexane/*i*PrOH = 90:10, flow rate 1.0 mL/min, λ = 280 nm, T = 25 °C.

Racemate:

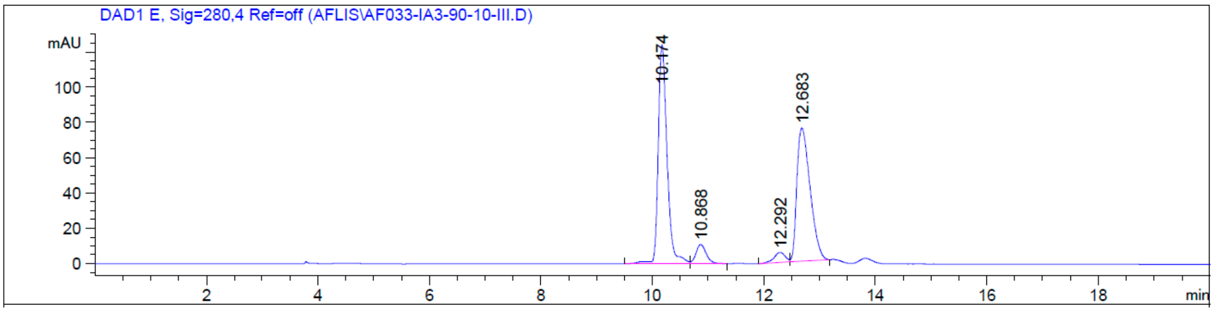

Signal 5: DAD1 E, Sig=280,4 Ref=off

Peak #	RetTime [min]	Type	Width [min]	Area [mAU*s]	Height [mAU]	Area %
1	10.174	BV	0.1682	1373.85547	124.04331	48.3214
2	10.868	VB	0.1940	142.48633	11.00582	5.0115
3	12.292	BV	0.2139	81.42694	5.75929	2.8640
4	12.683	VB	0.2596	1245.39087	75.45386	43.8031

Organocatalyzed asymmetric reaction: Major diastereomer: enantiomers: *t*R = 10.649 minutes (major); 12.074 minutes (minor) – 68% *ee*. Minor diastereomer: enantiomers: *t*R = 9.991 minutes (minor); 12.514 minutes (major) – 38% *ee*.

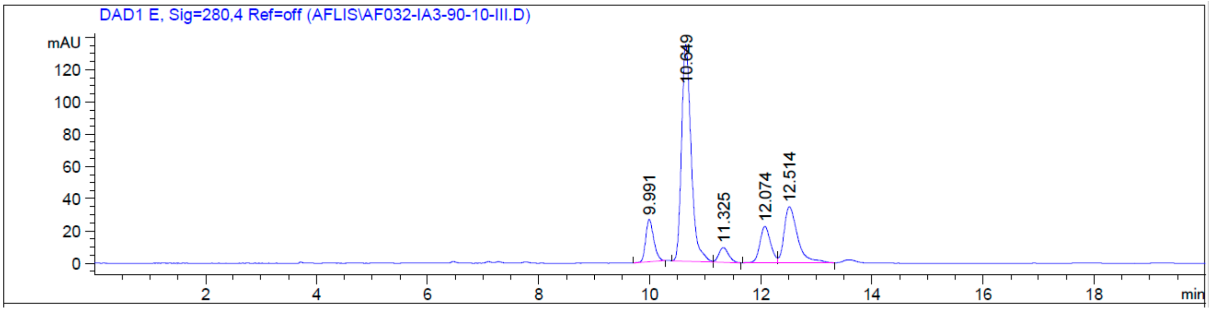

Signal 5: DAD1 E, Sig=280,4 Ref=off

Peak #	RetTime [min]	Type	Width [min]	Area [mAU*s]	Height [mAU]	Area %
1	9.991	BB	0.1544	263.83704	26.21147	9.1831
2	10.649	BV	0.1838	1607.82678	134.20007	55.9618
3	11.325	VB	0.1835	111.00471	9.15522	3.8636
4	12.074	BV	0.2042	302.94135	22.62899	10.5441
5	12.514	VB	0.2555	587.47137	34.71169	20.4474

3. Copies of ^{1}H- and ^{13}C-NMR spectra

Methyl 4-((*tert*-butoxycarbonyl)amino)-3-oxobutanoate (2a)

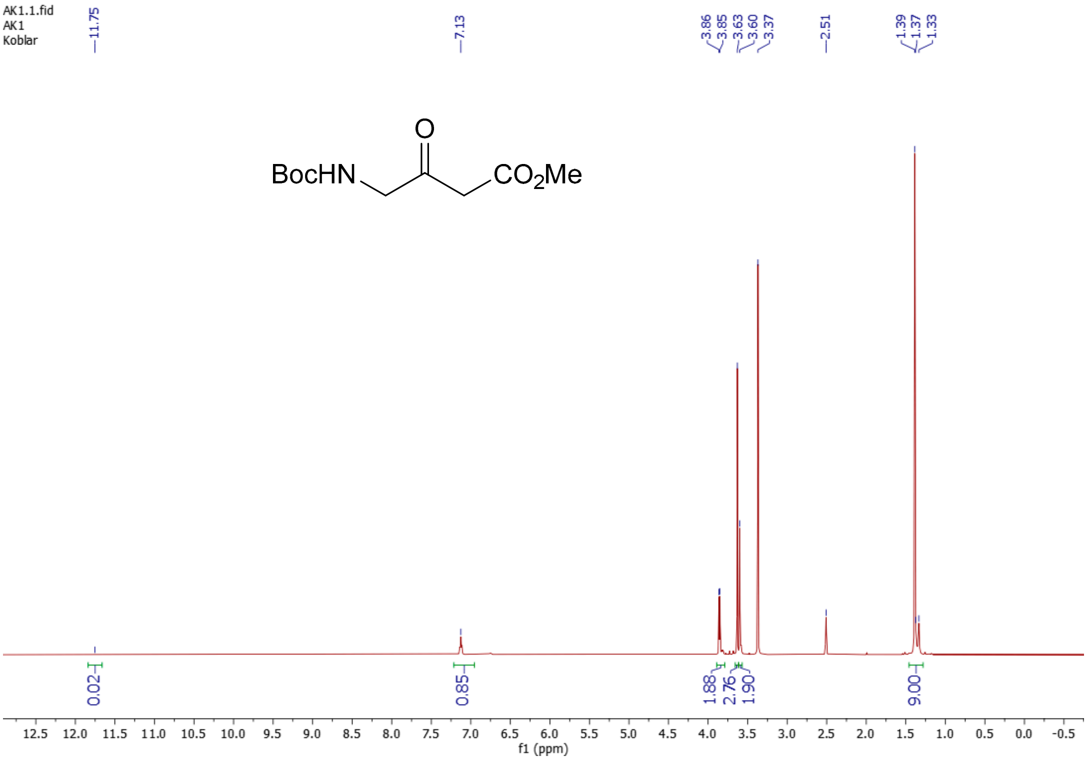

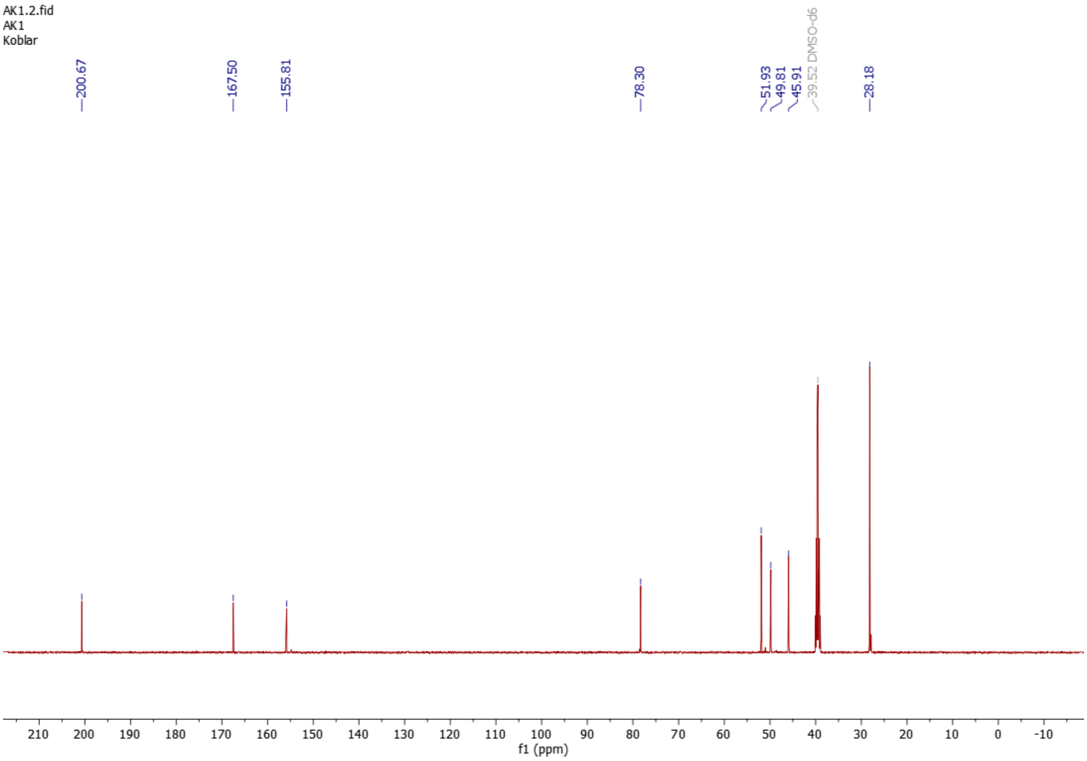

Methyl 5-((*tert*-butoxycarbonyl)amino)-3-oxopentanoate (2b)

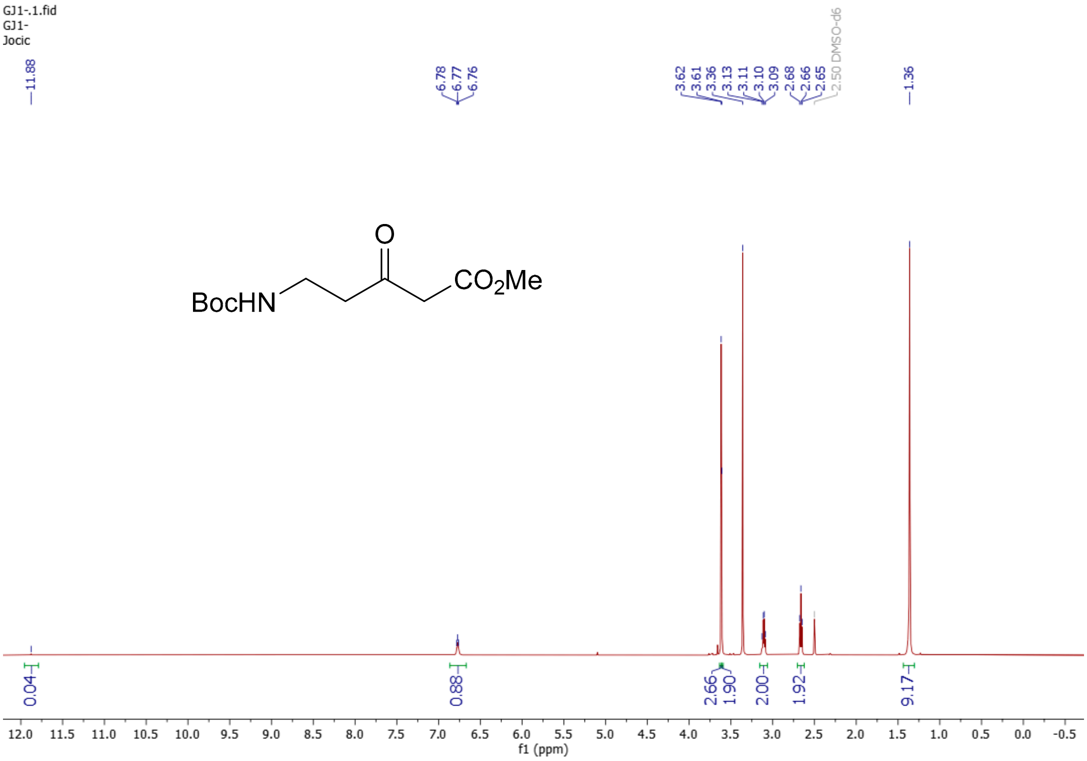

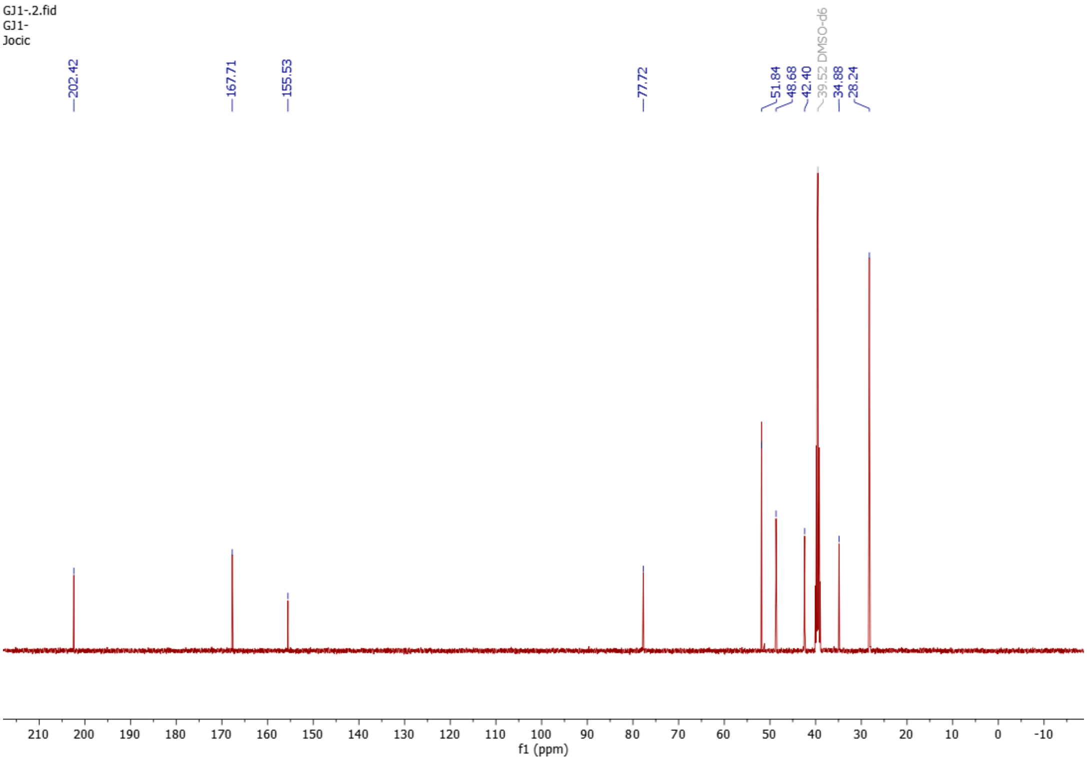

Methyl 4-((3-methylbut-2-en-1-yl)oxy)-3-oxobutanoate (2c)

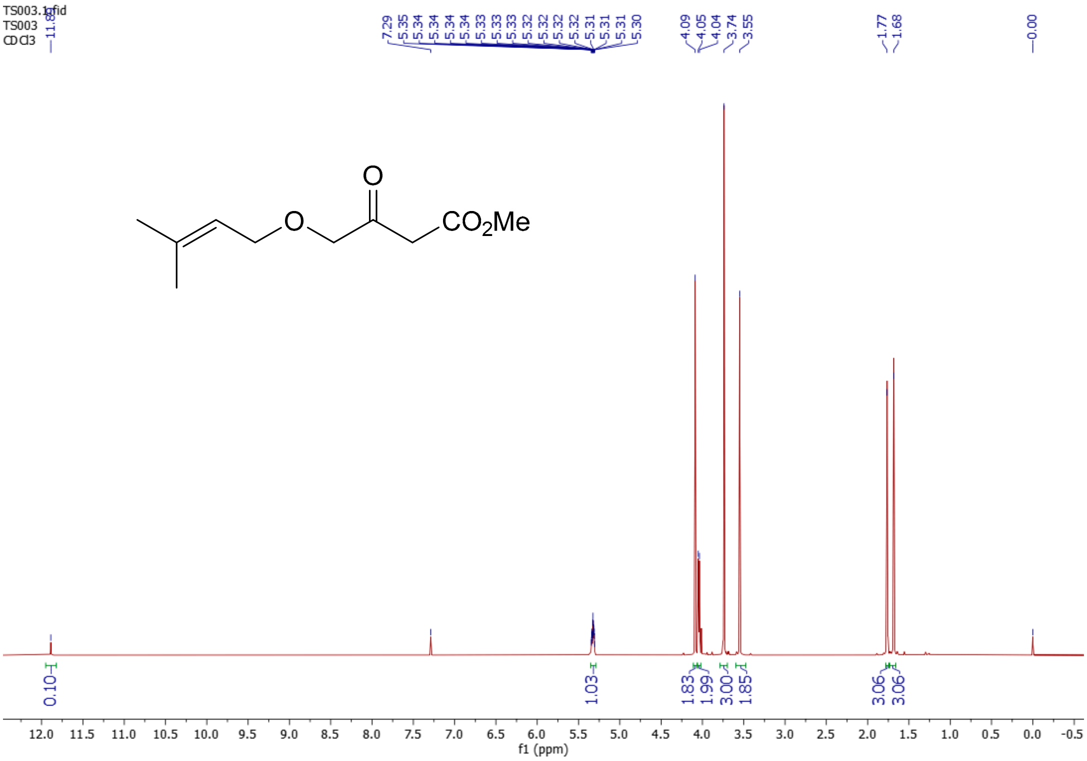

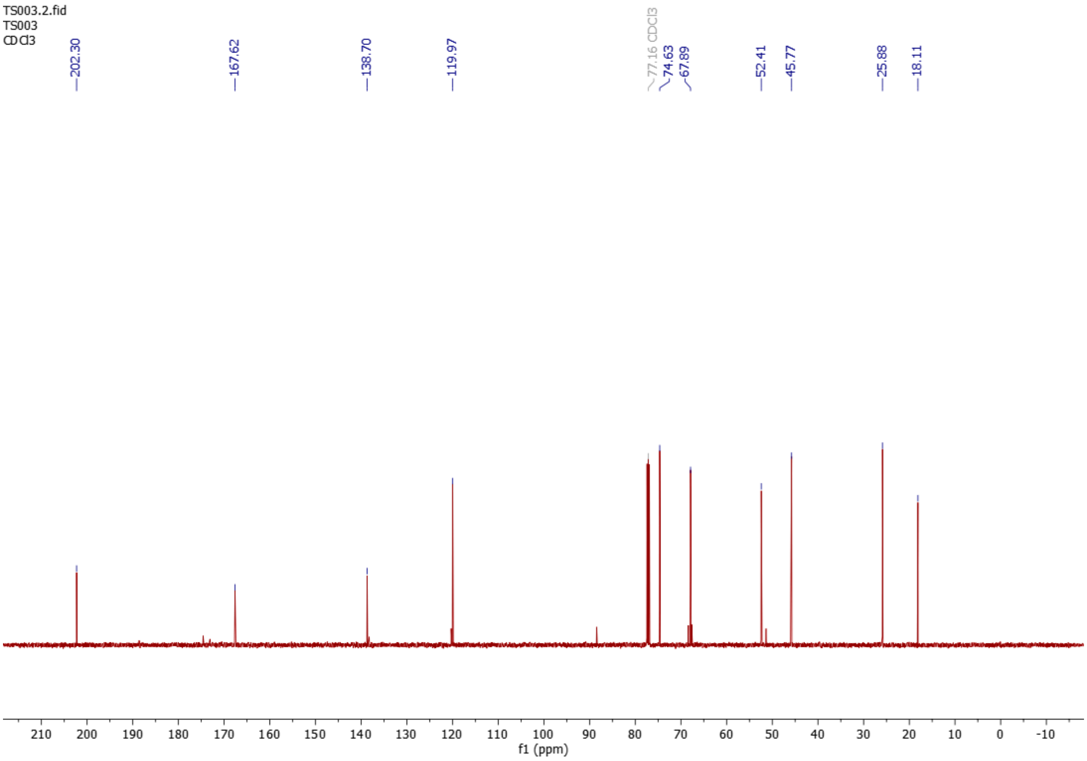

Methyl 3-oxooctadecanoate (2d)

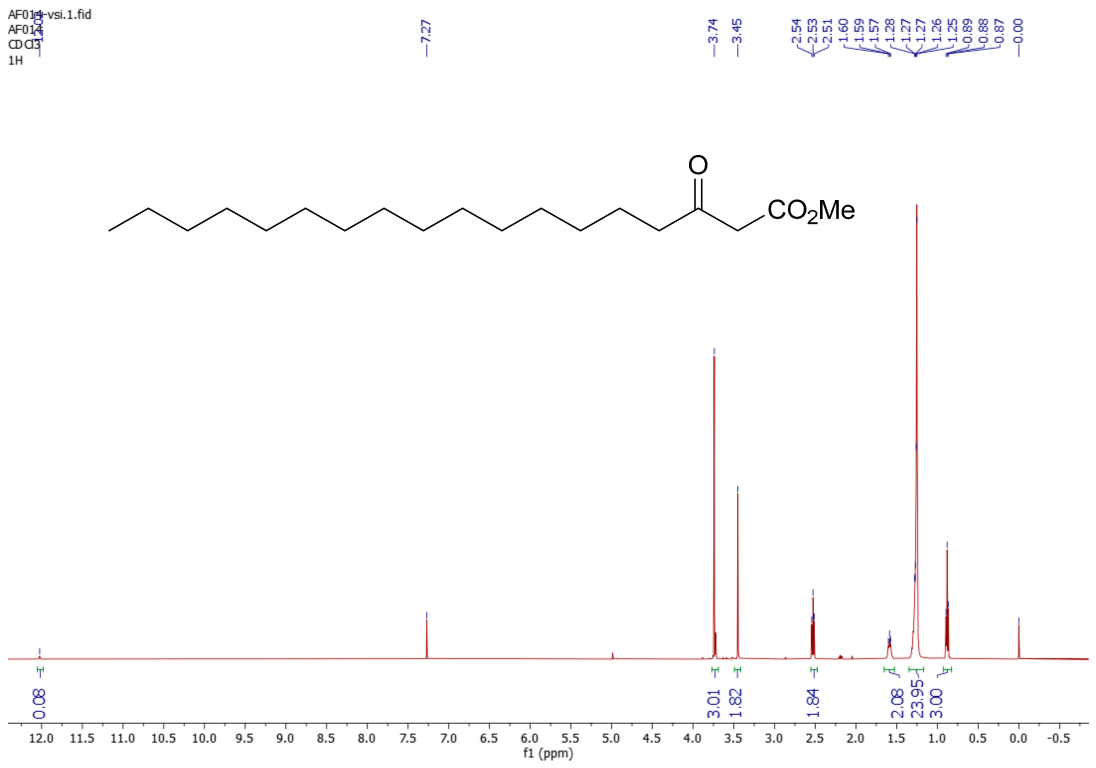

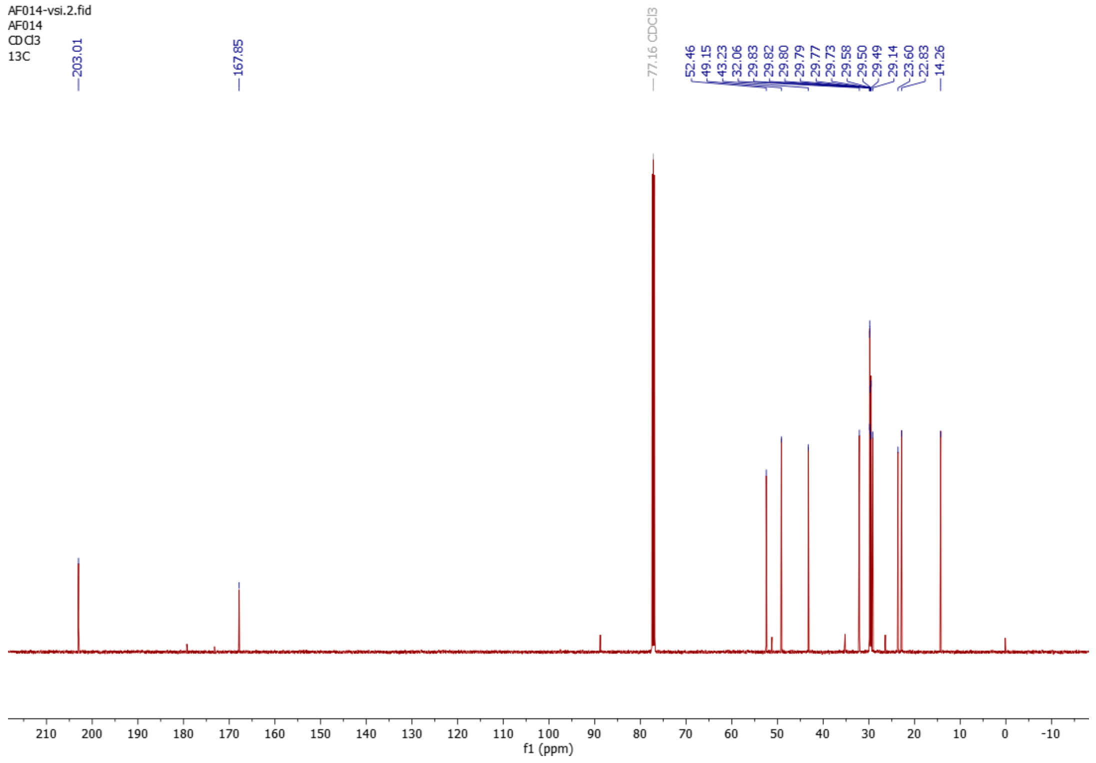

Methyl 3-oxoicosanoate (2e)

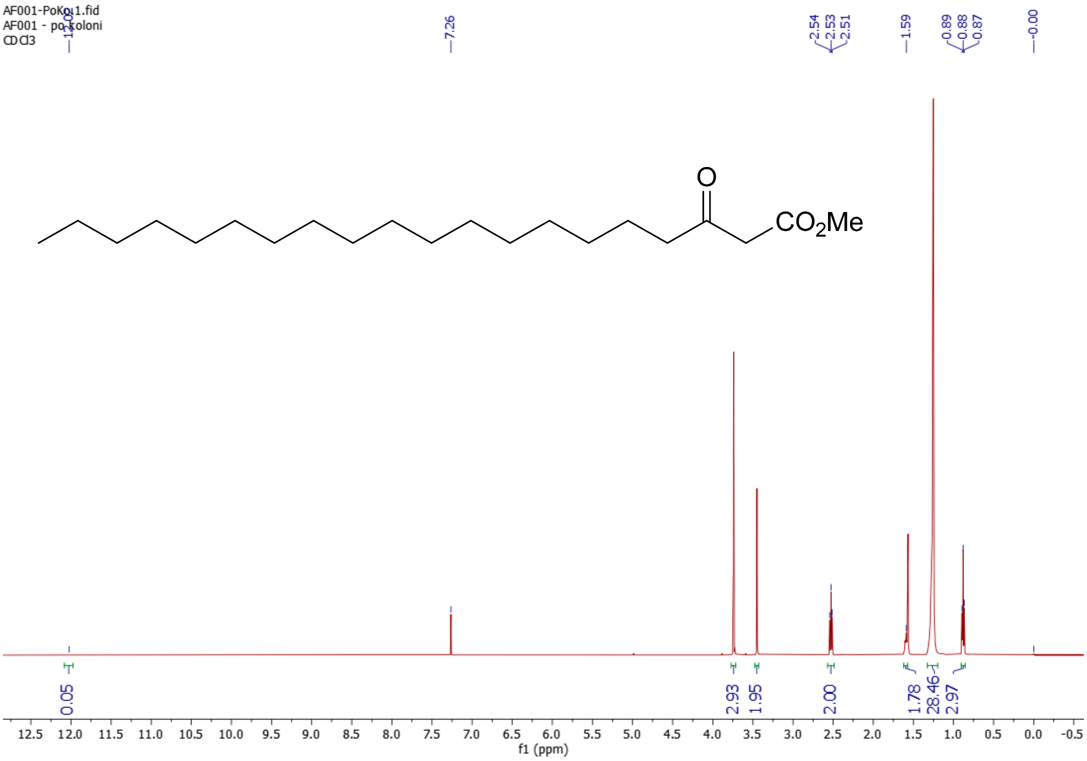

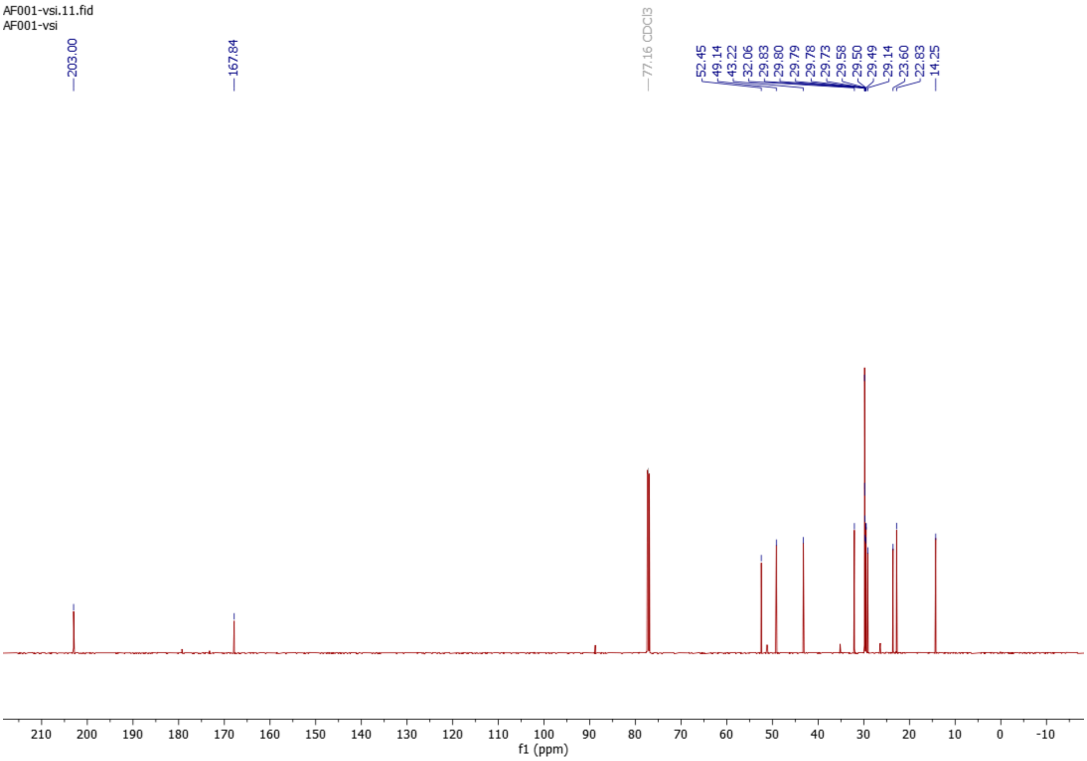

tert-Butyl 3-oxoicosanoate (2f)

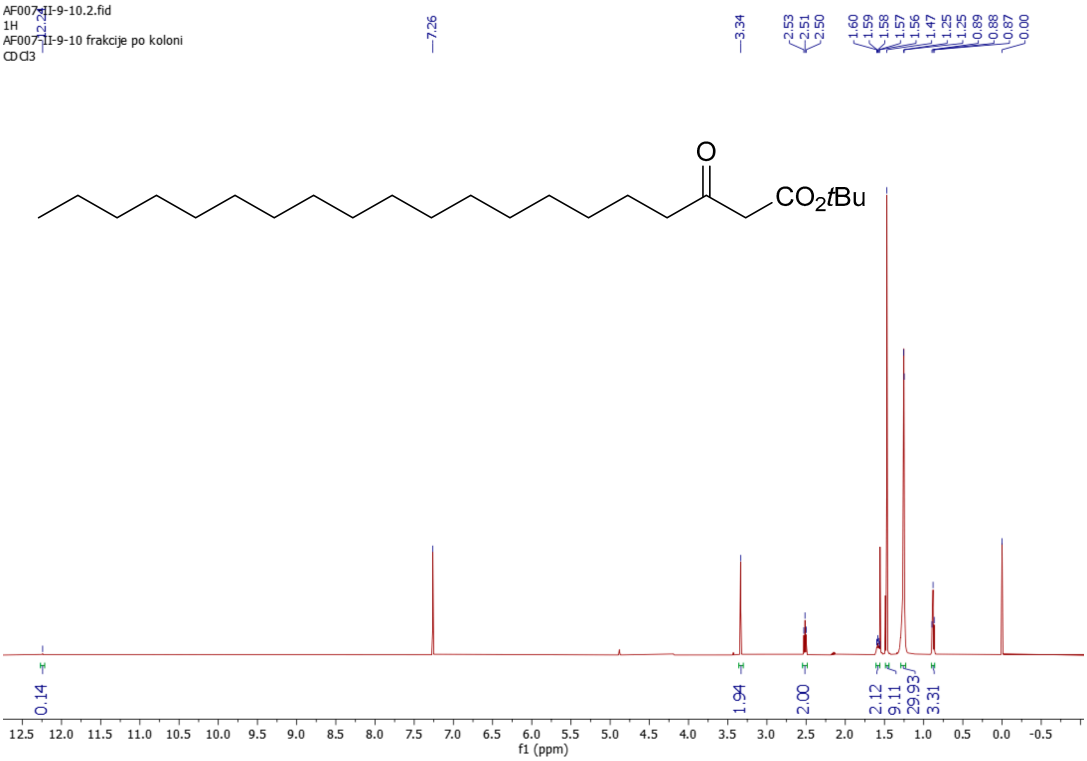

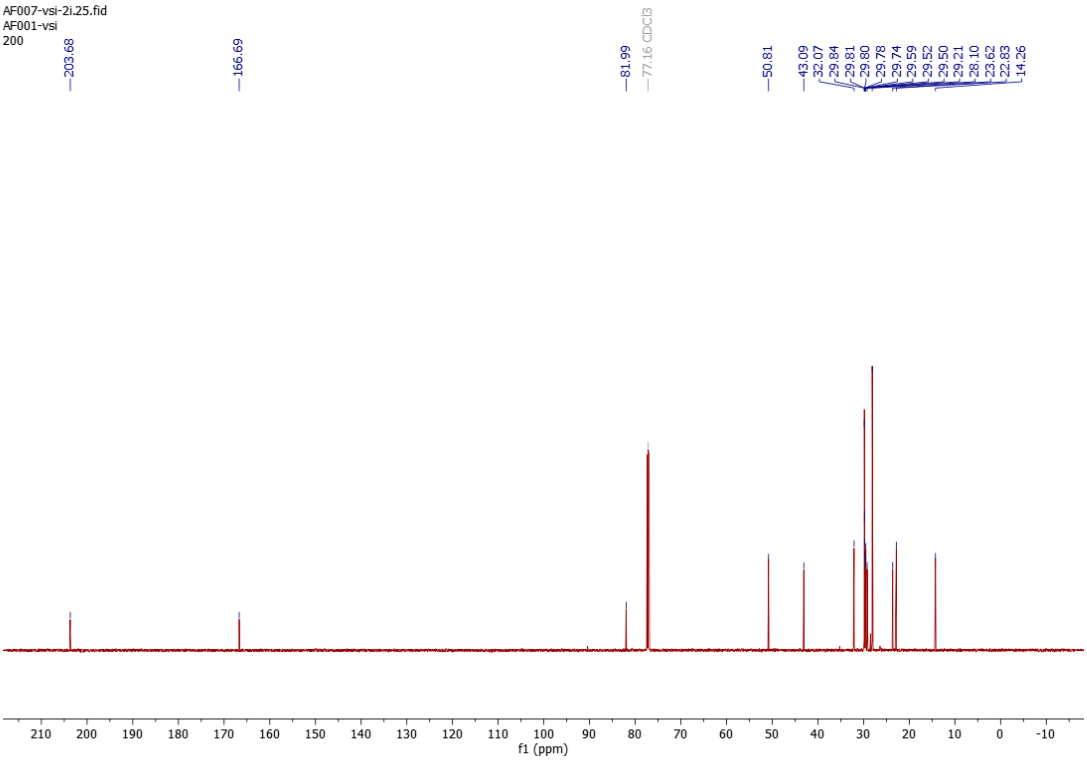

Methyl (11*Z*,14*Z*)-3-oxoicosa-11,14-dienoate (2g)

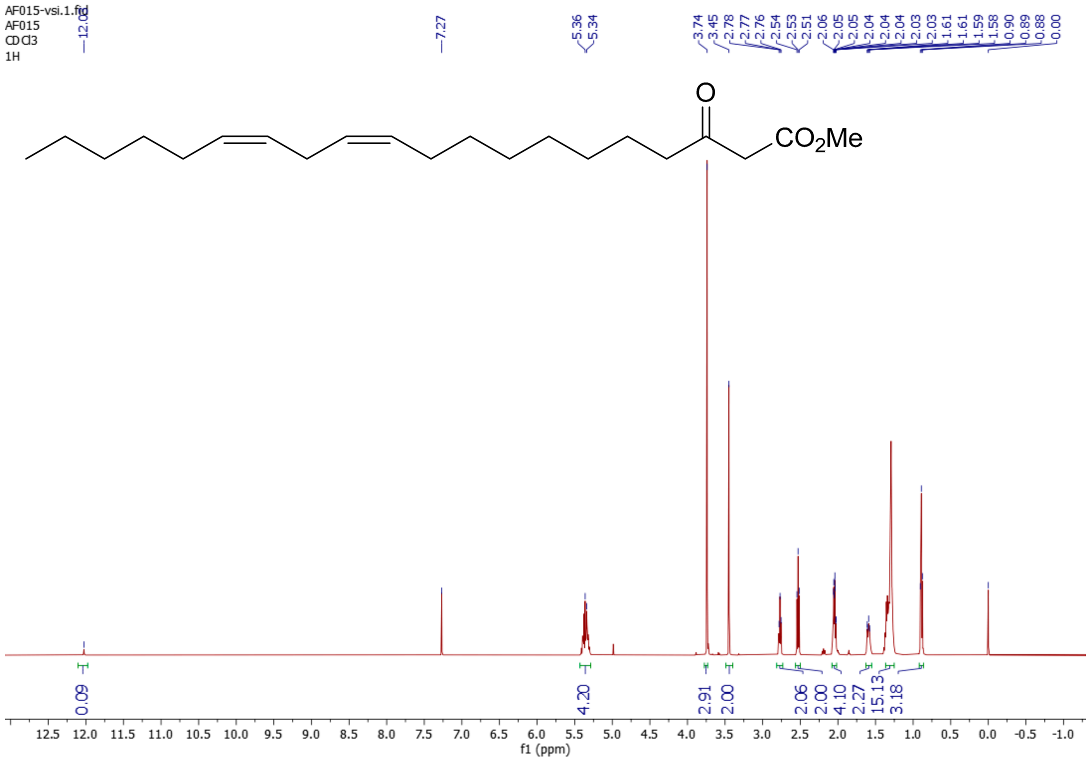

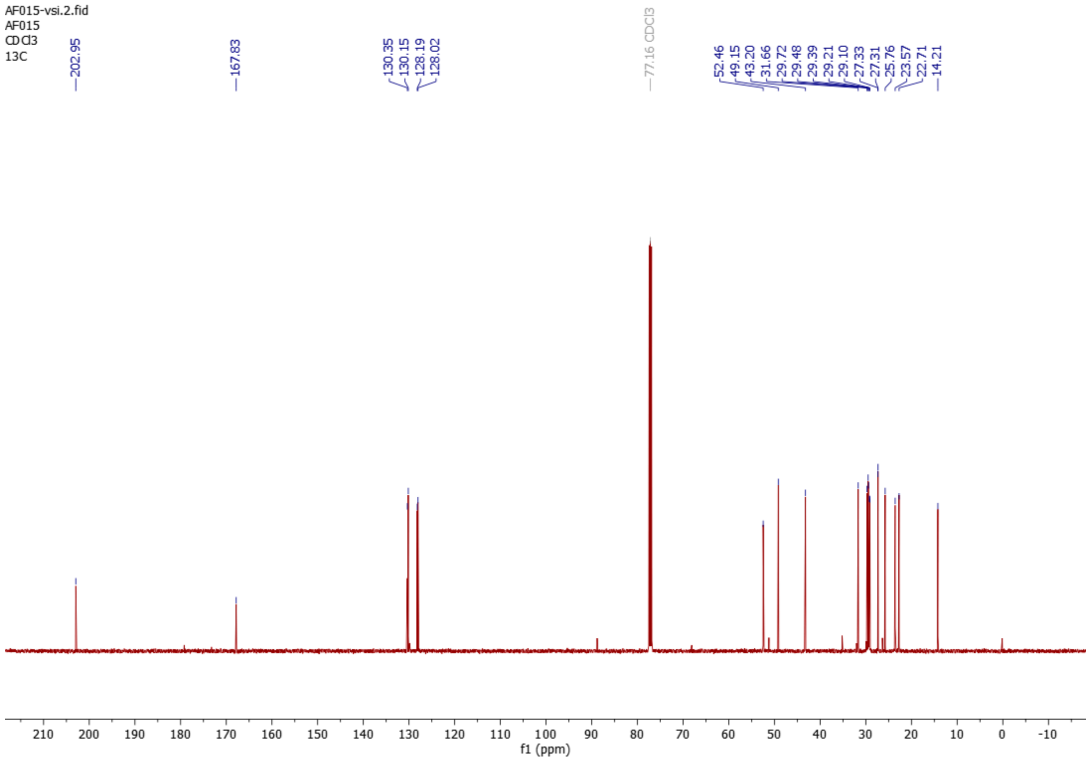

Methyl (*S*)-4-((*tert*-butoxycarbonyl)amino)-3-oxo-5-phenylpentanoate (10a)

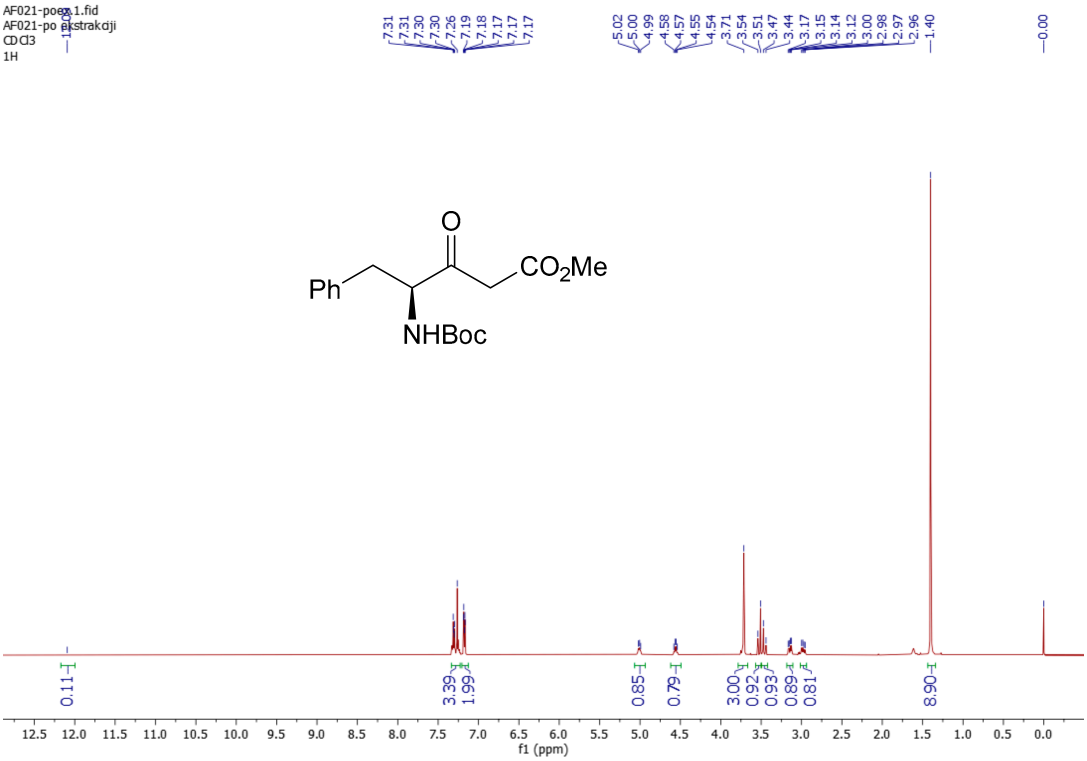

Methyl (*S*)-8-(((benzyloxy)carbonyl)amino)-4-((*tert*-butoxycarbonyl)amino)-3-oxooctanoate (10b)

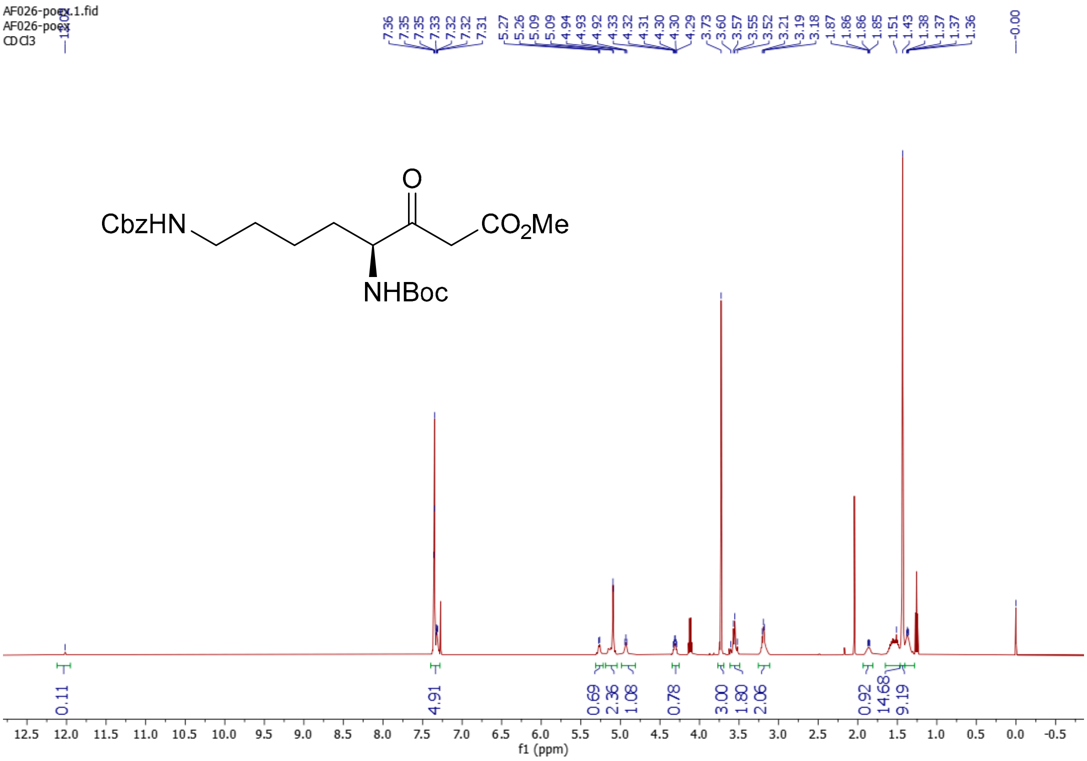

7-Benzyl 1-methyl (*S*)-4-((*tert*-butoxycarbonyl)amino)-3-oxoheptanedioate (10c)

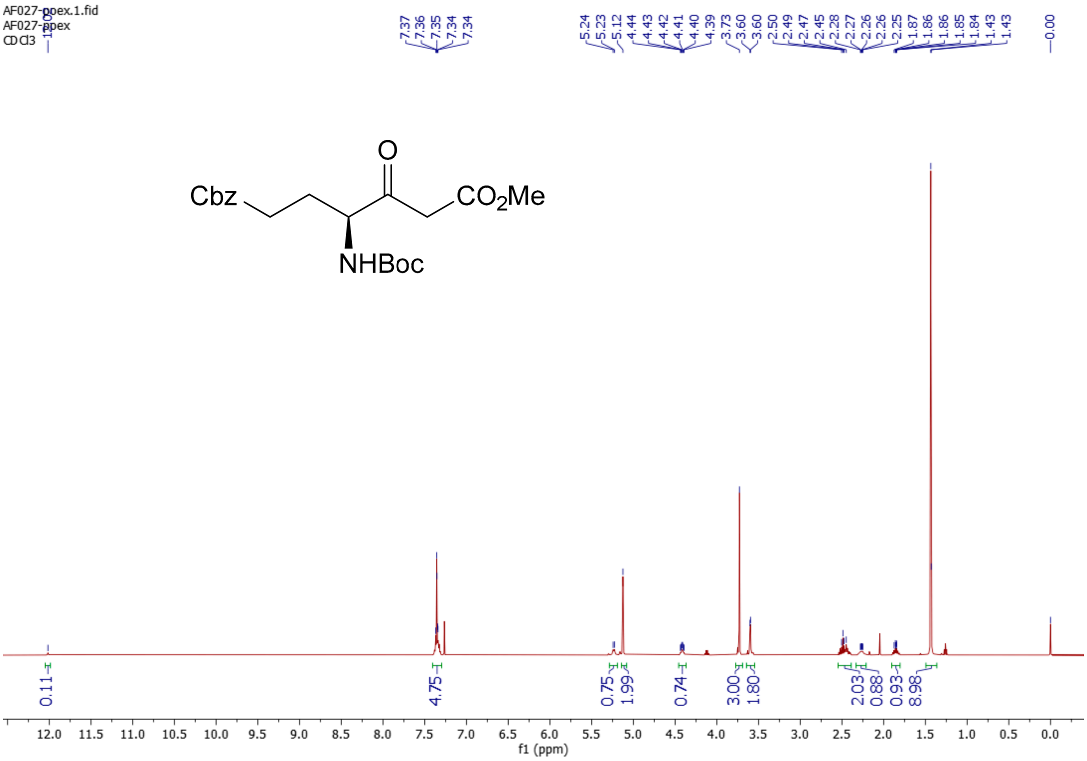

Methyl (*S*)-5-((*tert*-butoxycarbonyl)amino)-2-stearamidopentanoate (4)

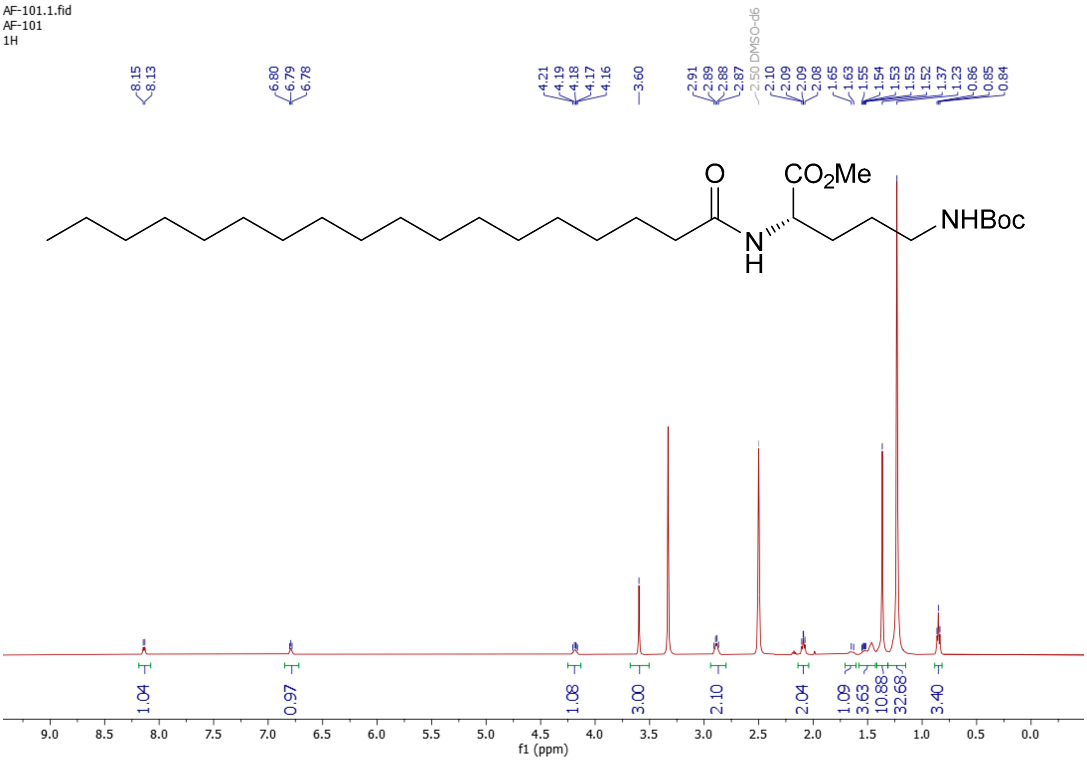

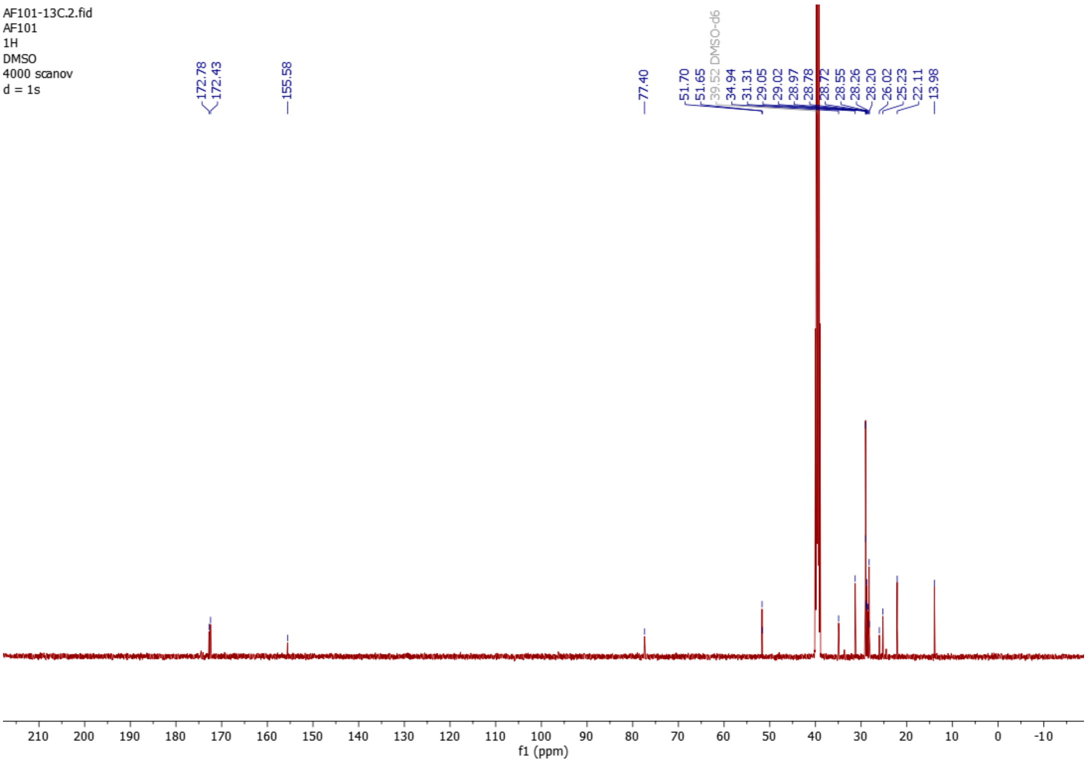

(*S*)-5-((*tert*-Butoxycarbonyl)amino)-2-stearamidopentanoic acid (5)

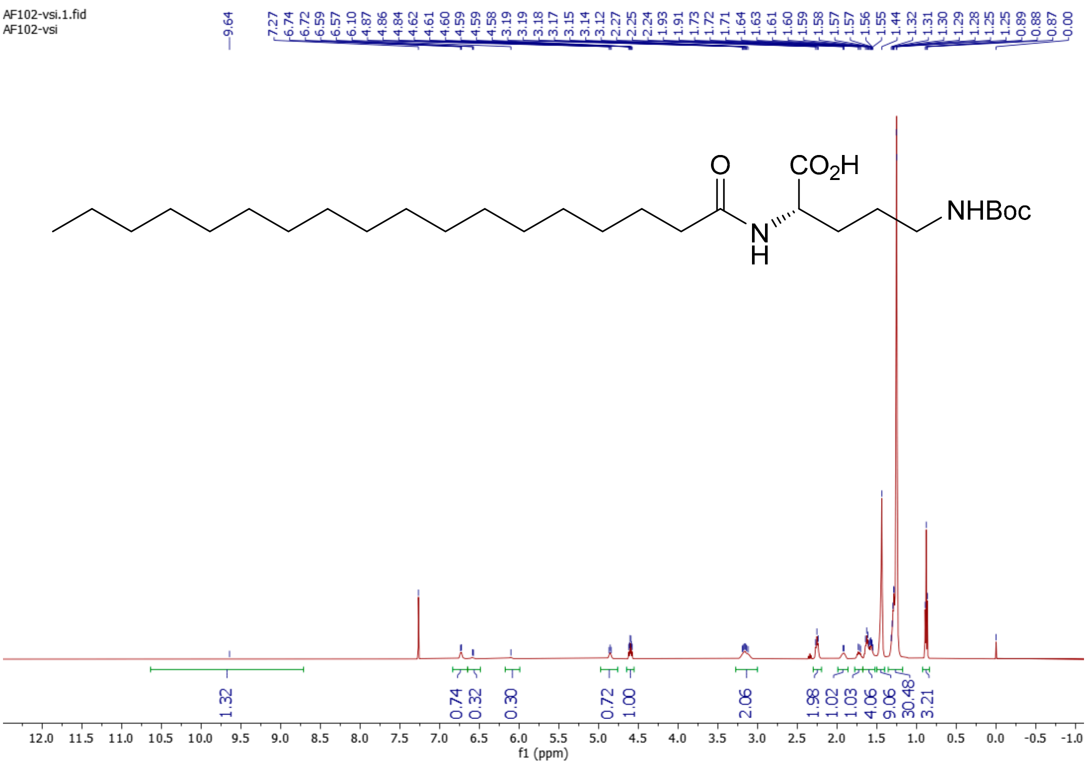

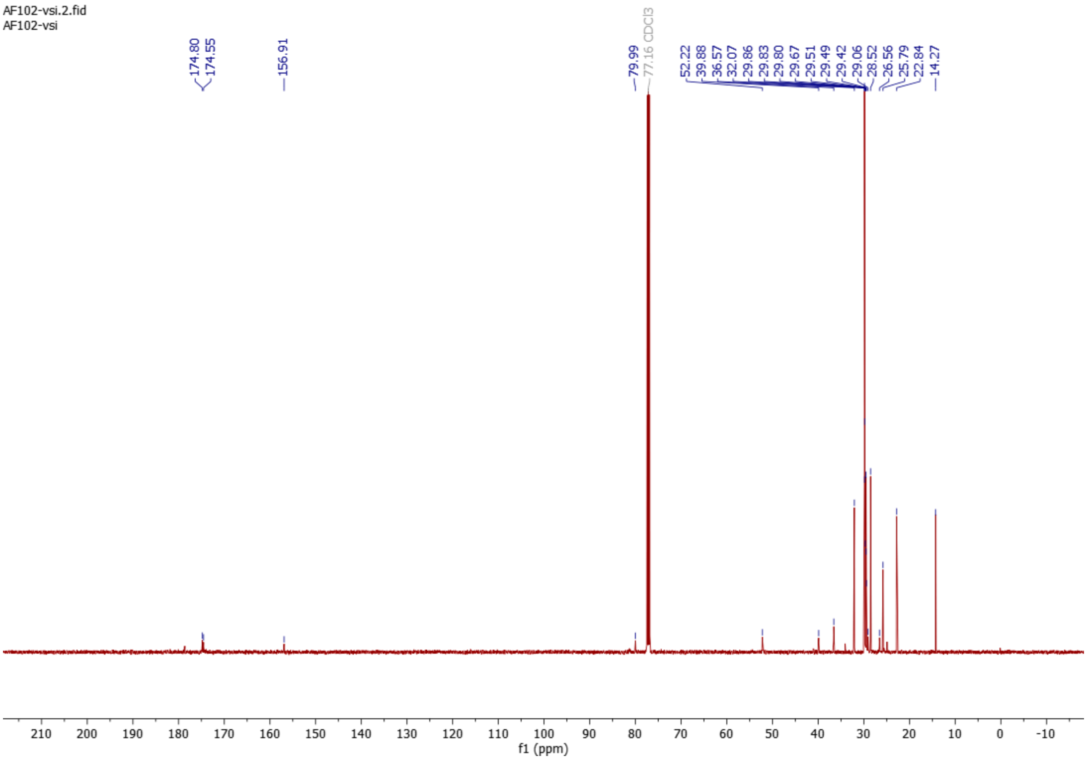

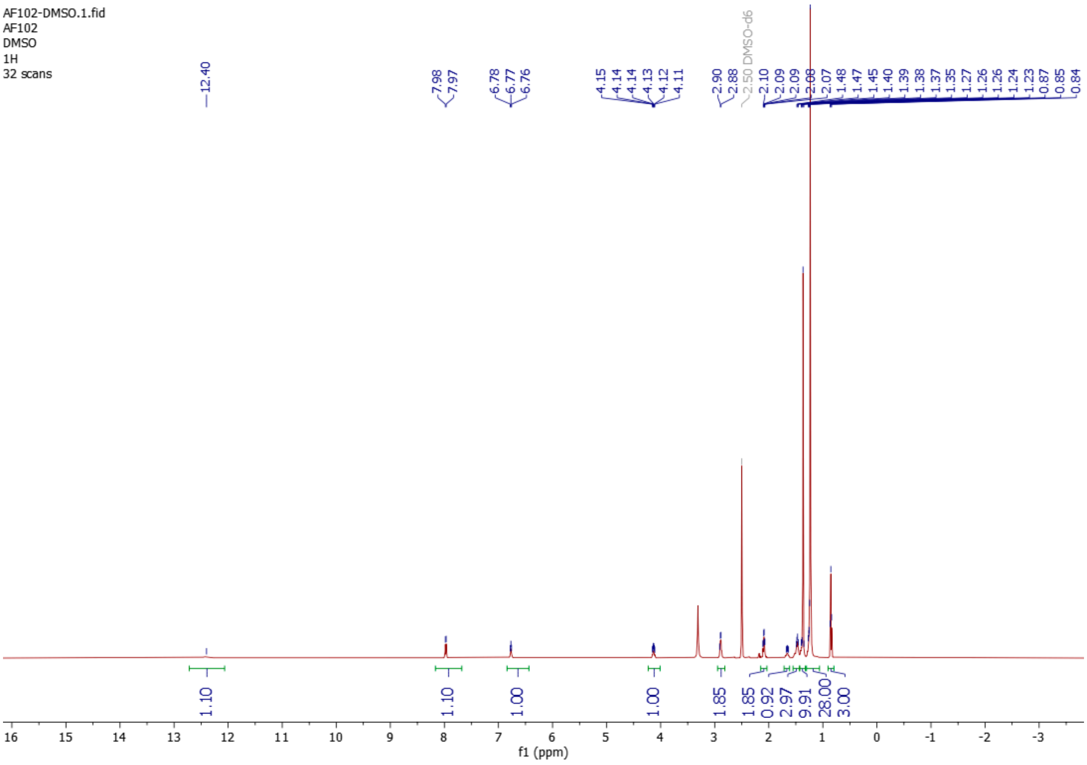
AF102-DMSO.1.fid
AF102
DMSO
1H
32 scans
12.40
7.98
7.97
6.78
6.77
6.76
4.15
4.14
4.14
4.13
4.12
4.11
2.90
2.88
2.50 DMSO-d6
2.10
2.09
2.09
2.08
2.07
1.48
1.47
1.45
1.40
1.39
1.38
1.37
1.35
1.27
1.26
1.26
1.24
1.23
0.87
0.85
0.84
1.10
1.10
1.00
1.00
1.85
1.85
0.92
2.97
9.91
28.00
3.00
f1 (ppm)

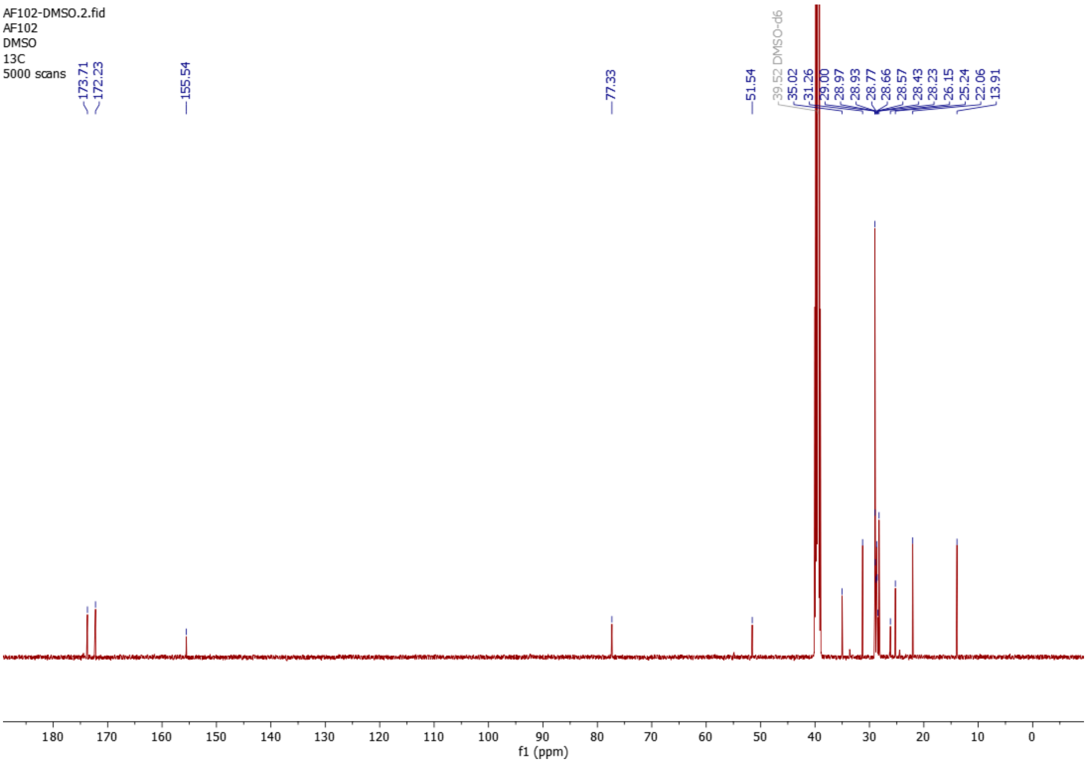
AF102-DMSO.2.fid
AF102
DMSO
13C
5000 scans
173.71
172.23
155.54
77.33
51.54
39.52 DMSO-d6
35.02
31.26
29.00
28.97
28.93
28.77
28.66
28.57
28.43
28.23
26.15
25.24
22.06
13.91
f1 (ppm)

Methyl (*S*)-7-((*tert*-butoxycarbonyl)amino)-3-oxo-4-stearamidoheptanoate (2h)

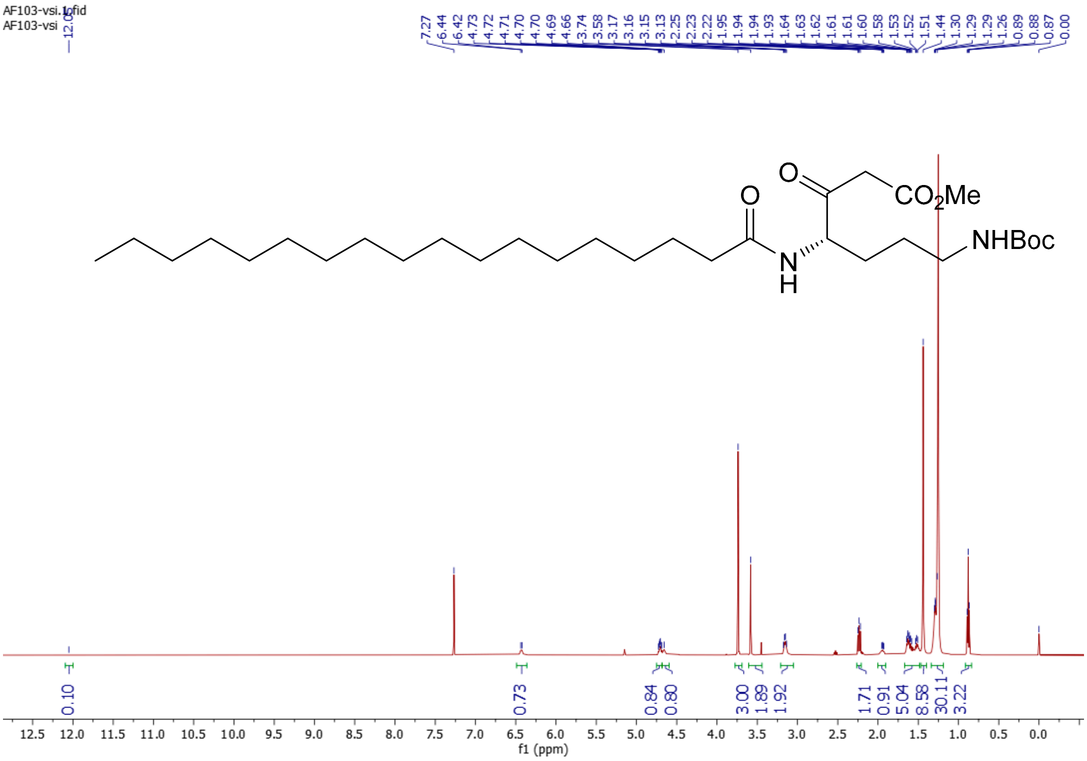

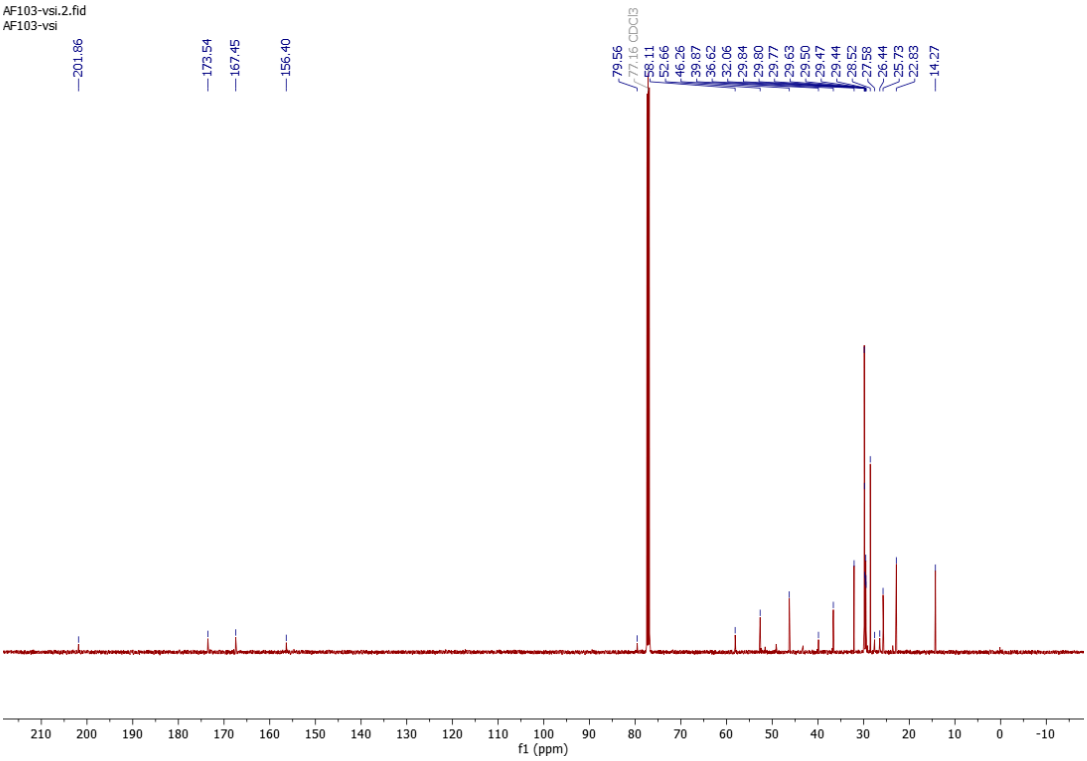

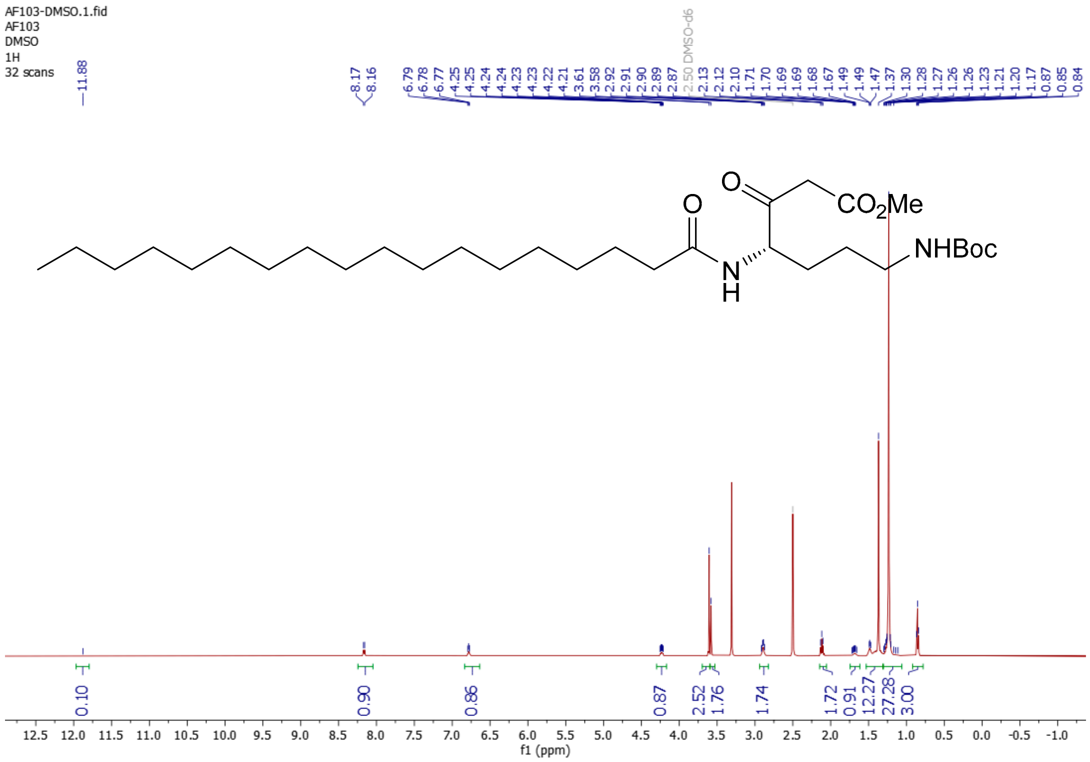
AF103-DMSO.1.fid
AF103
DMSO
1H
32 scans
11.88
8.17
8.16
6.79
6.78
6.77
4.25
4.25
4.24
4.24
4.23
4.23
4.22
4.21
3.61
3.58
2.92
2.91
2.90
2.89
2.87
2.50 DMSO-d6
2.13
2.12
2.10
1.71
1.70
1.69
1.69
1.68
1.67
1.49
1.49
1.47
1.37
1.30
1.28
1.27
1.26
1.26
1.23
1.21
1.20
1.17
0.87
0.85
0.84
O
O
CO2Me
NHBoc
N
H
0.10
0.90
0.86
0.87
2.52
1.76
1.74
1.72
0.91
12.27
27.28
3.00
f1 (ppm)

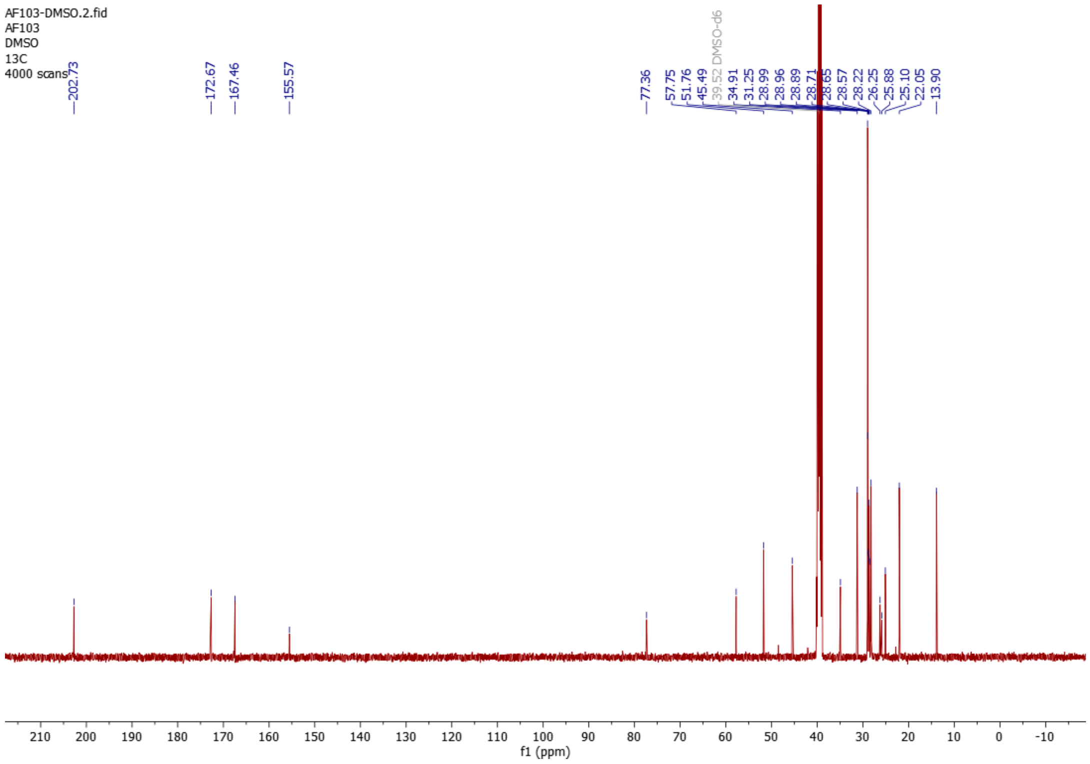
AF103-DMSO.2.fid
AF103
DMSO
13C
4000 scans
202.73
172.67
167.46
155.57
77.36
57.75
51.76
45.49
39.52 DMSO-d6
34.91
31.25
28.99
28.96
28.89
28.71
28.65
28.57
28.22
26.25
25.88
25.10
22.05
13.90
f1 (ppm)

(*S*)-5-Methoxy-5-oxo-4-stearamidopentan-1-aminium 2,2,2-trifluoroacetate (6)

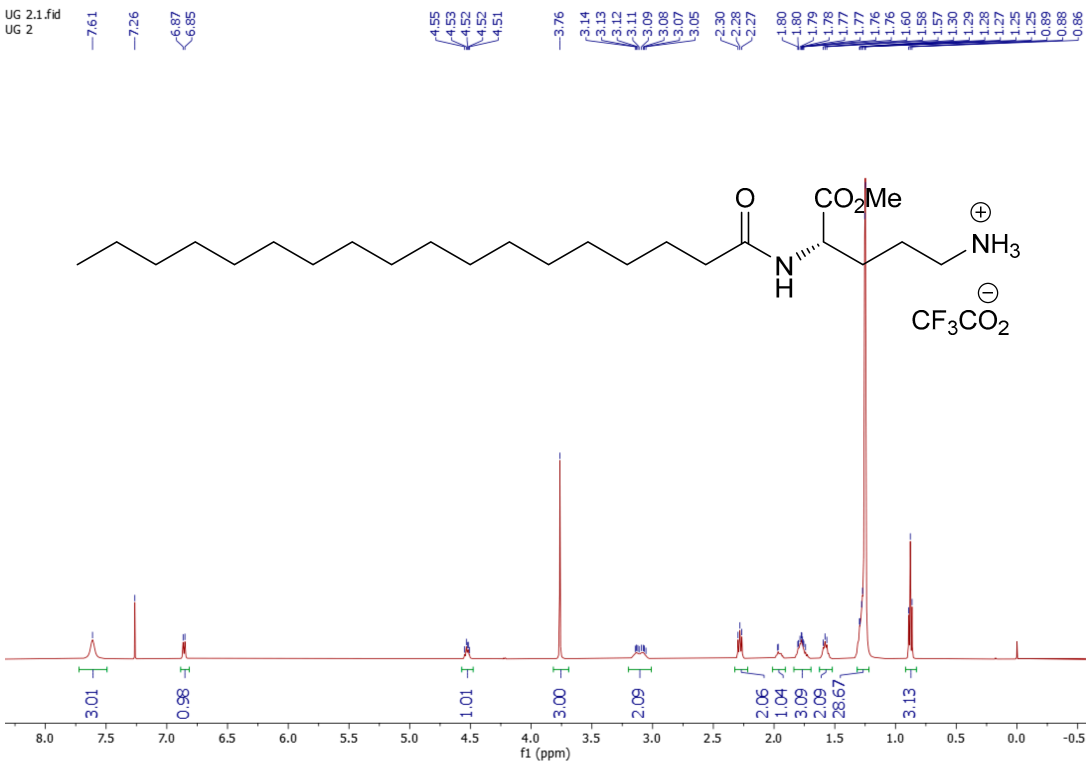

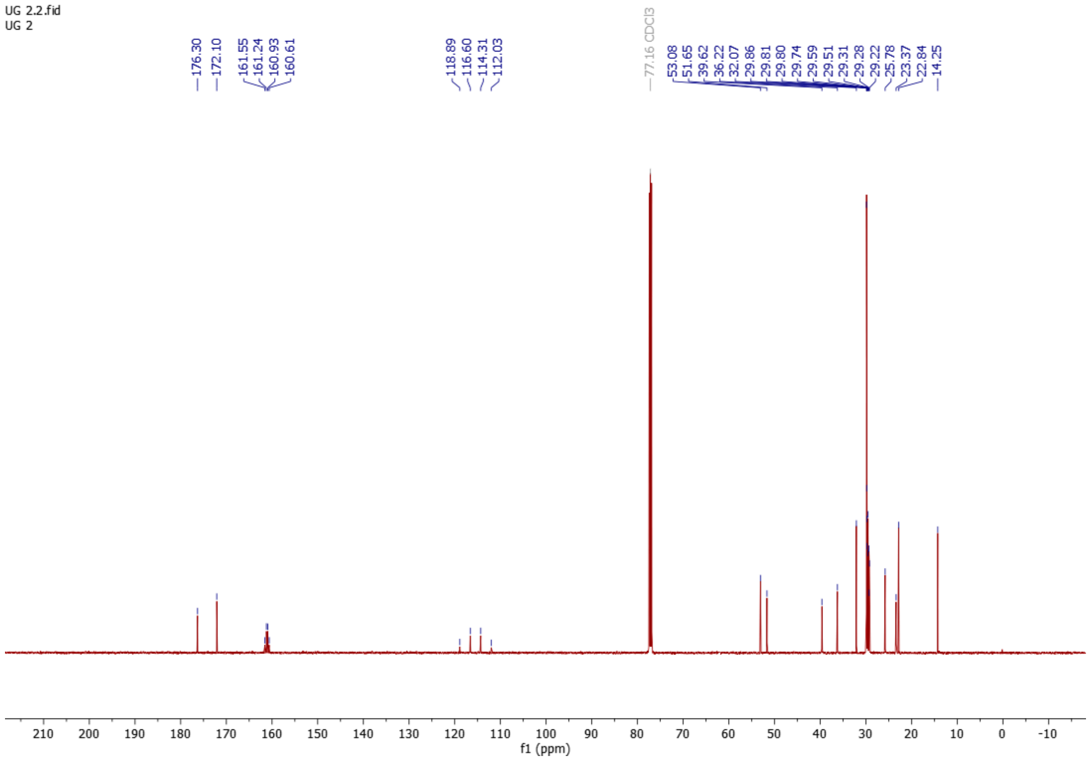

Methyl (*S*)-2,5-distearamidopentanoate (7)

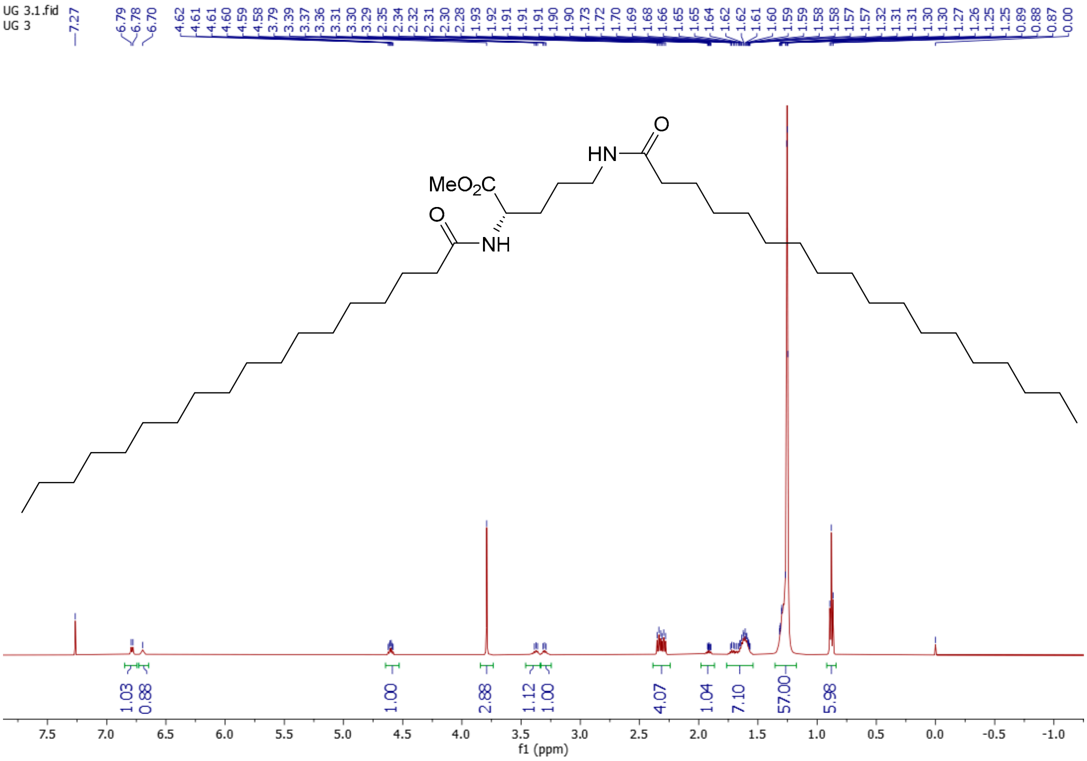

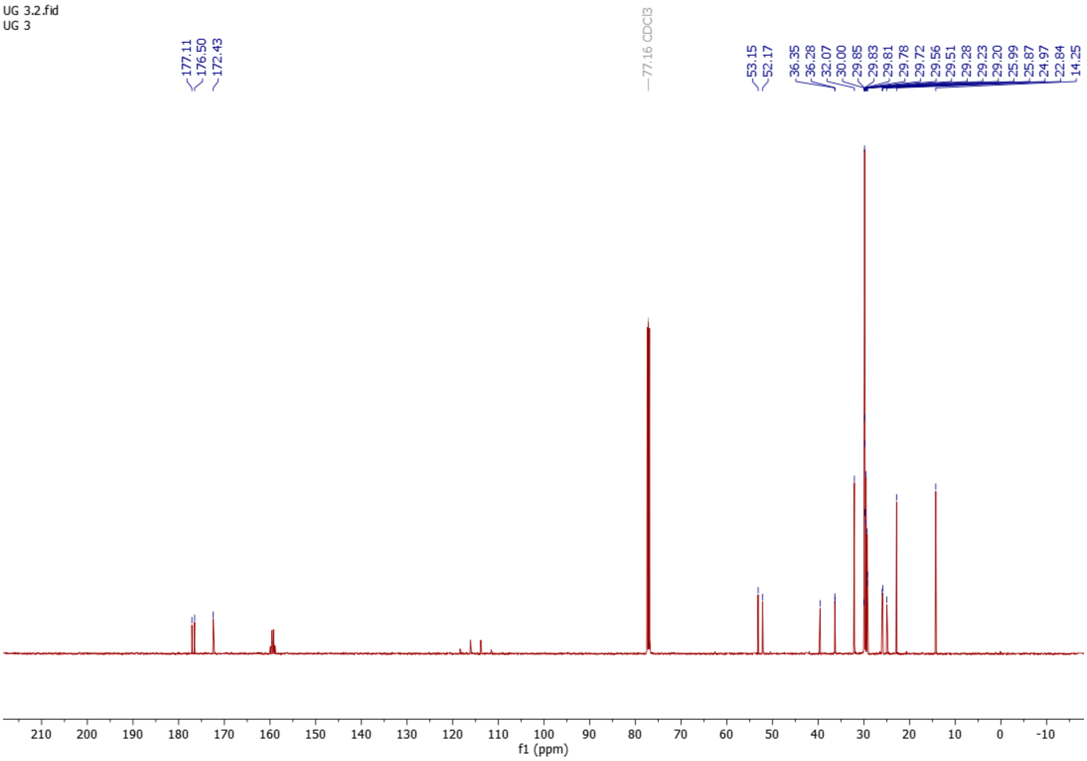

(*S*)-2,5-Distearamidopentanoic acid (8)

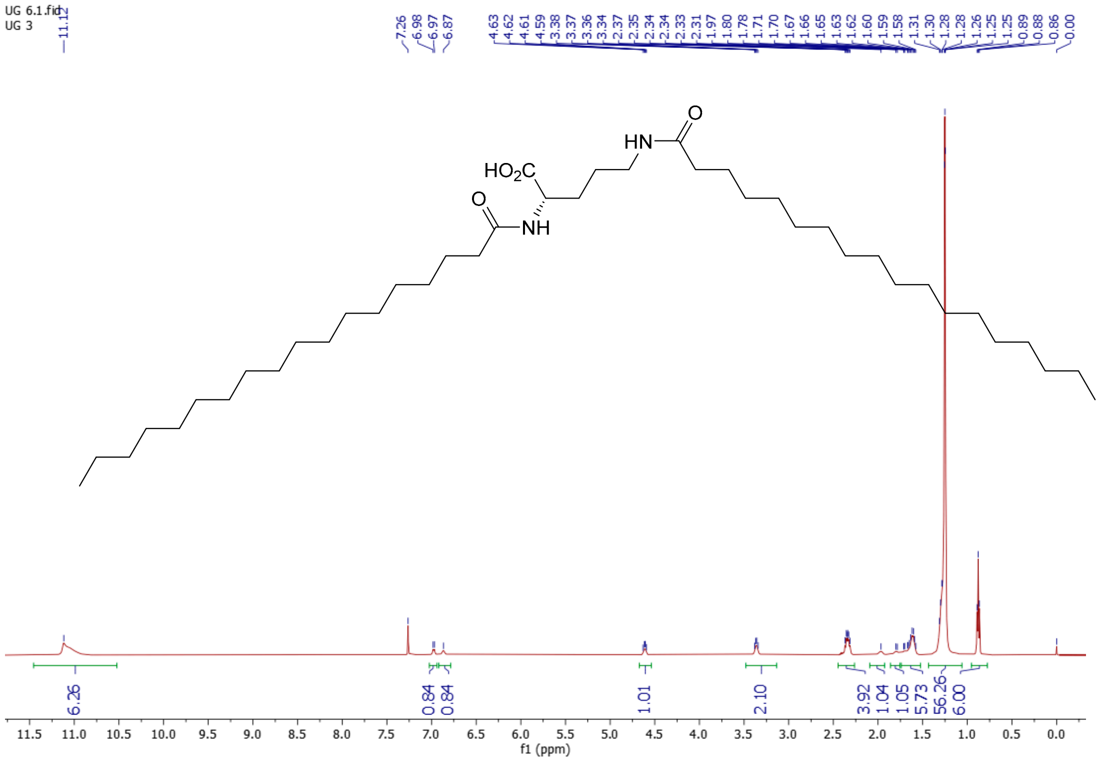

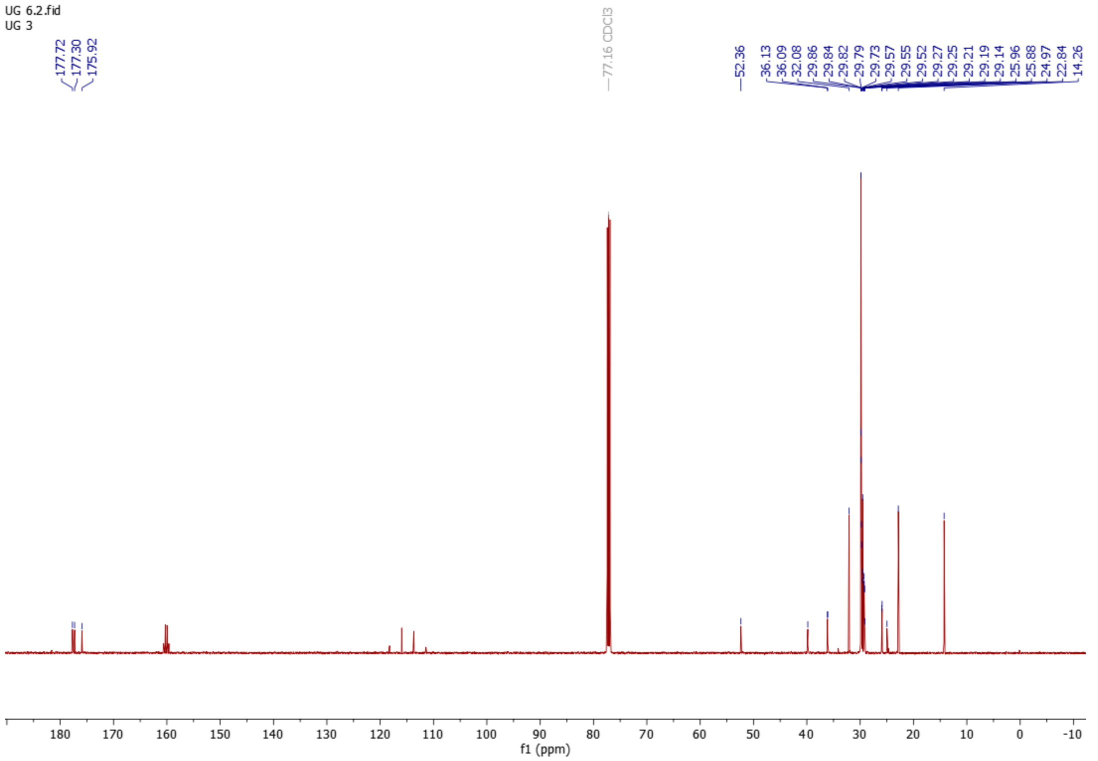

Methyl (*S*)-3-oxo-4,7-distearamidoheptanoate (2i)

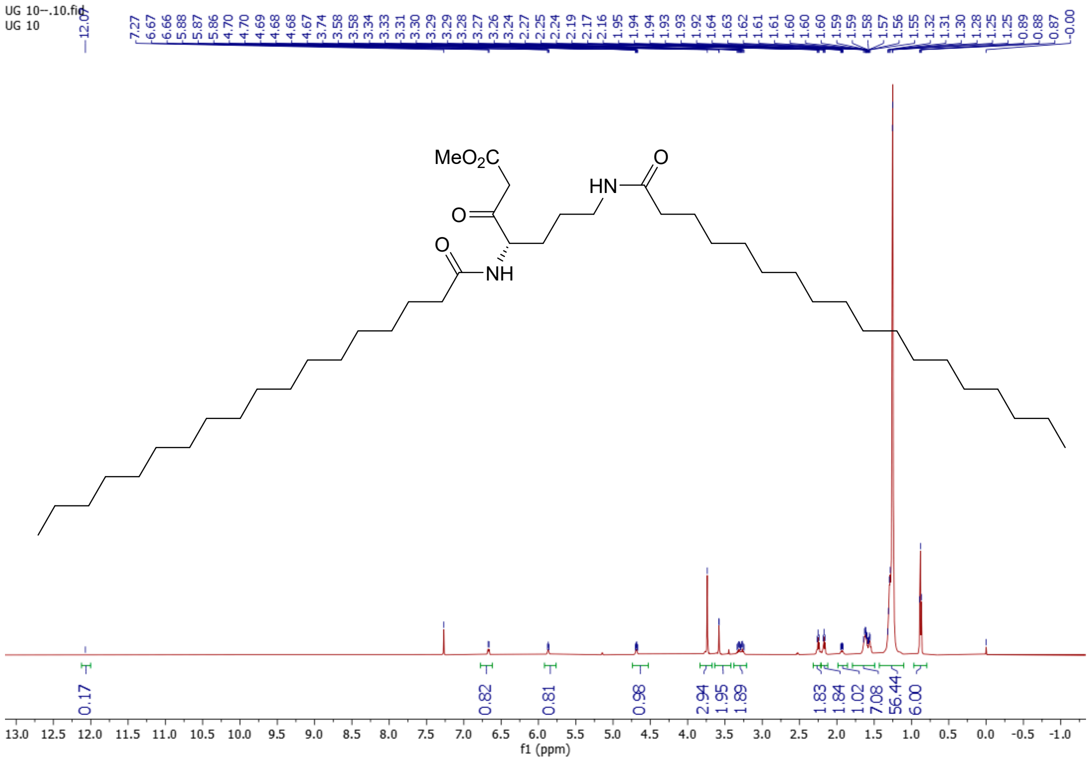

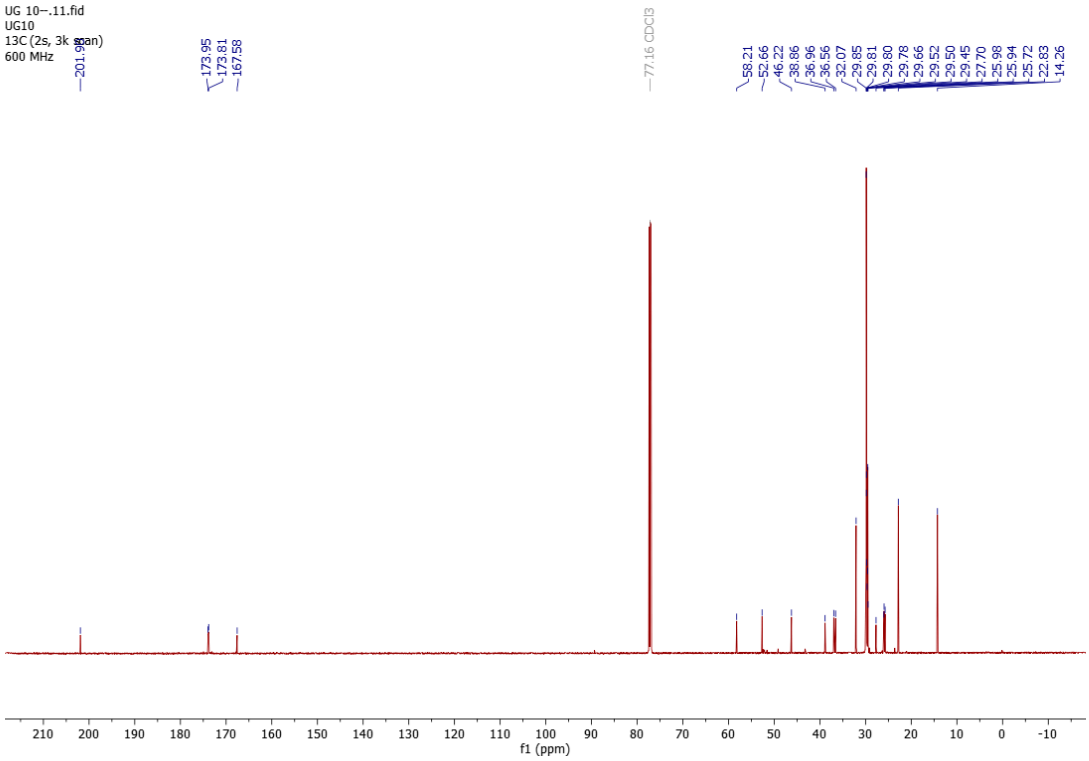

1-(*tert*-Butyl) 3-methyl 5-benzyl-4-oxo-4,5-dihydro-1*H*-pyrrole-1,3-dicarboxylate (11a) and 1-(*tert*-butyl) 3-methyl 5-benzyl-4-hydroxy-1*H*-pyrrole-1,3-dicarboxylate (11a')

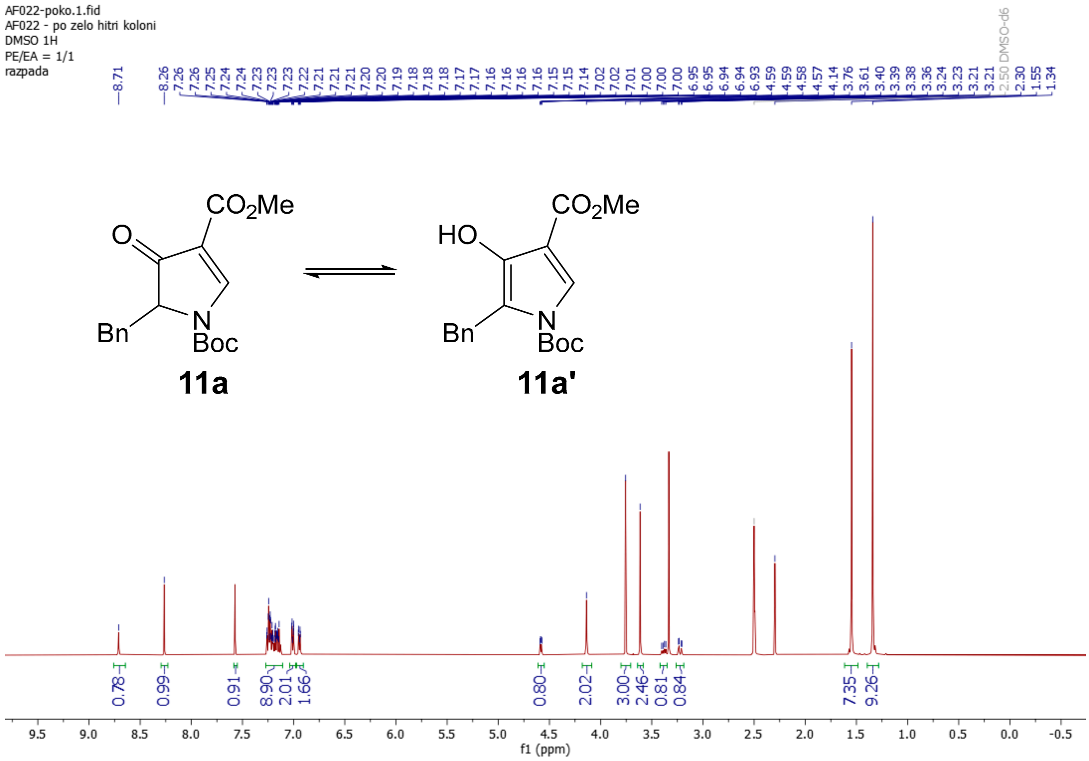

The sample contains toluene.

1-(*tert*-Butyl) 3-methyl 5-(4-(((benzyloxy)carbonyl)amino)butyl)-4-oxo-4,5-dihydro-1*H*-pyrrole-1,3-dicarboxylate (11b) and 1-(*tert*-butyl) 3-methyl 5-(4-(((benzyloxy)carbonyl)amino)butyl)-4-hydroxy-1*H*-pyrrole-1,3-dicarboxylate (11b')

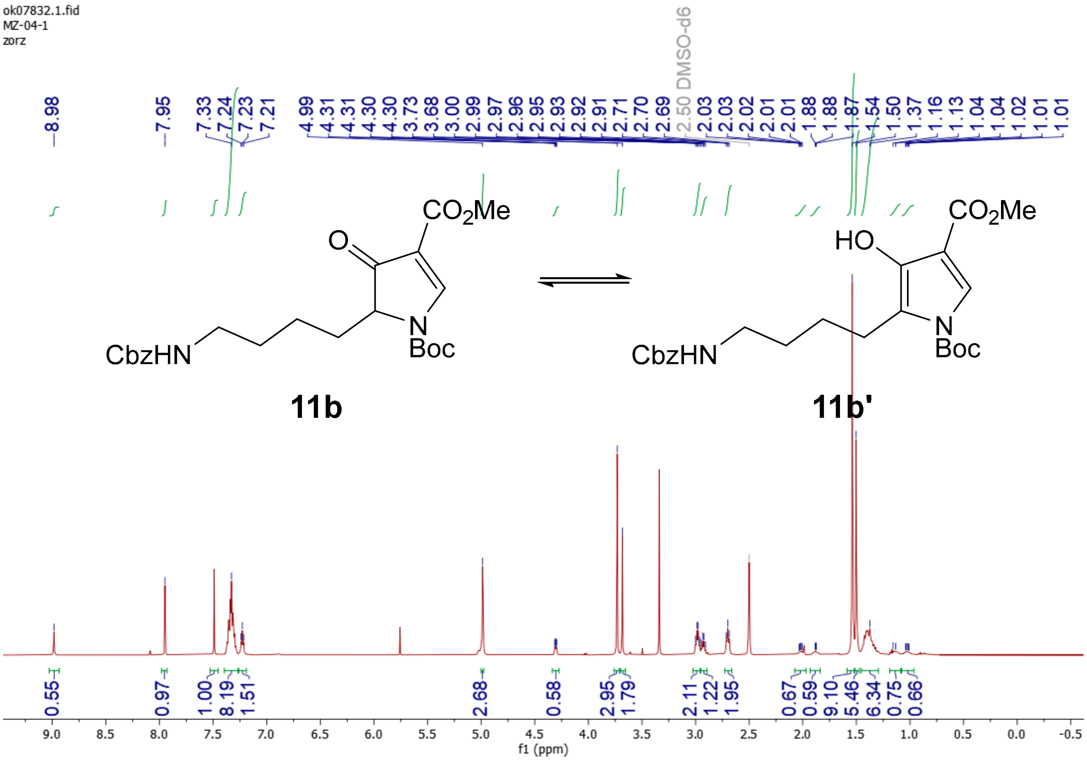

1-(*tert*-Butyl) 3-methyl 5-(3-(benzyloxy)-3-oxopropyl)-4-oxo-4,5-dihydro-1*H*-pyrrole-1,3-dicarboxylate (11c) and 1-(*tert*-butyl) 3-methyl 5-(3-(benzyloxy)-3-oxopropyl)-4-hydroxy-1*H*-pyrrole-1,3-dicarboxylate (11c')

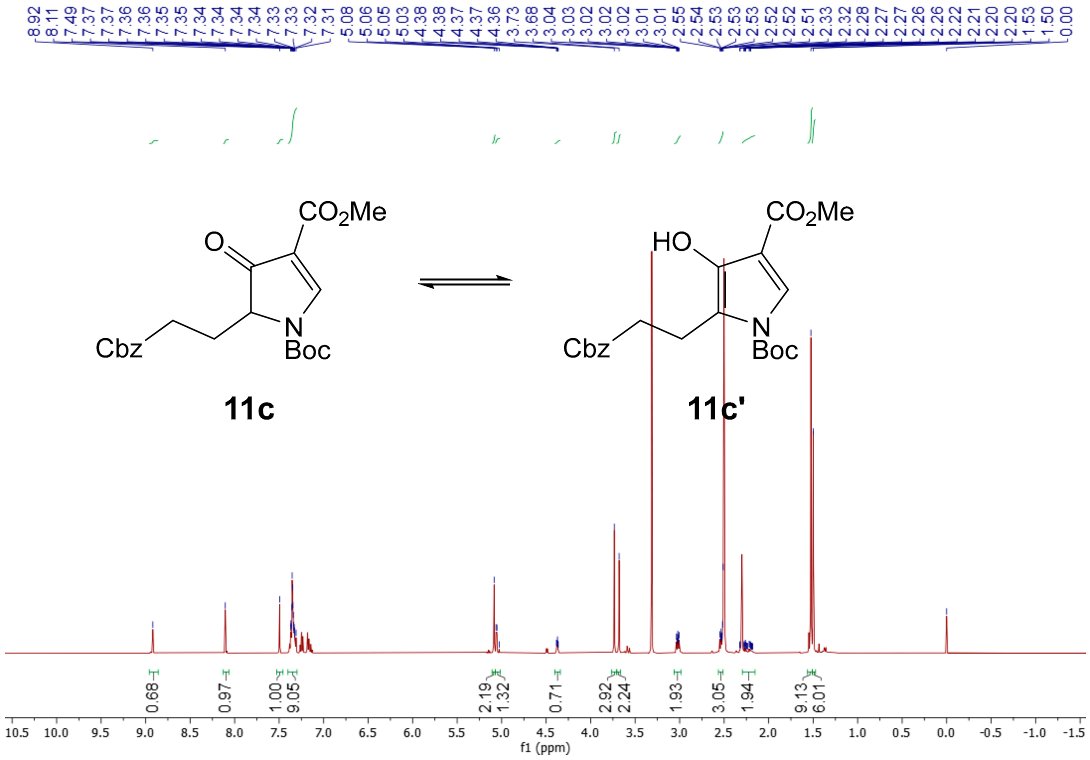

The sample contains toluene.

Hexadecan-1-ol (13)

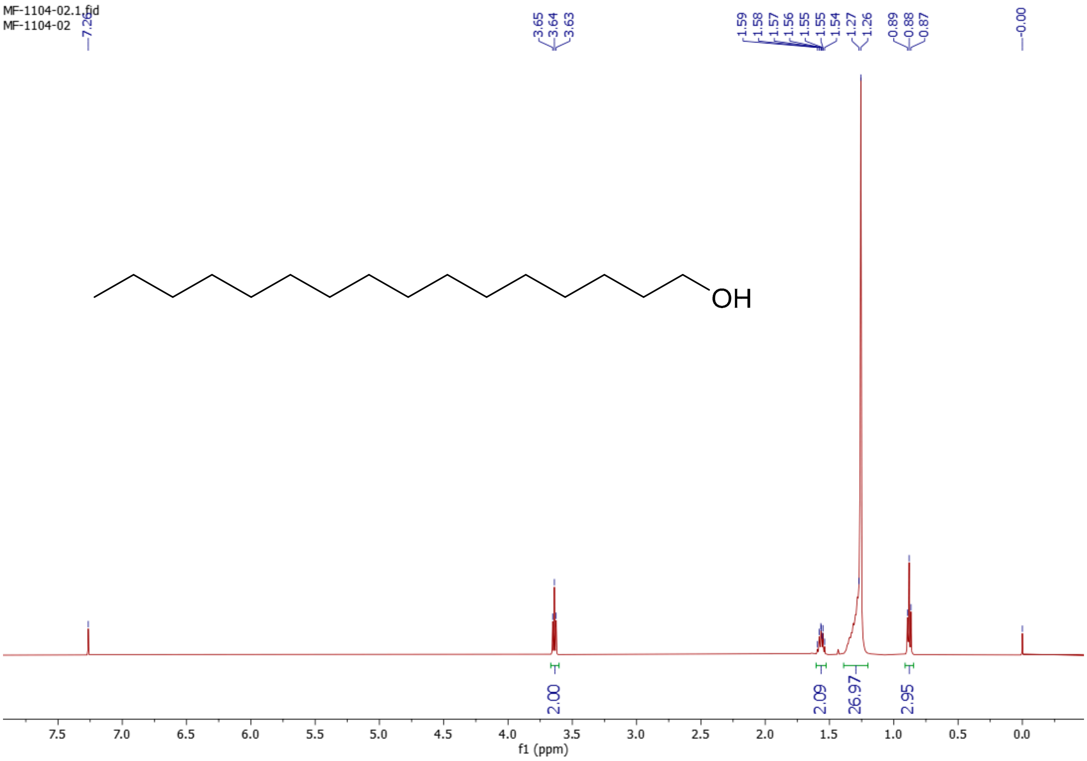

Palmitaldehyde (14)

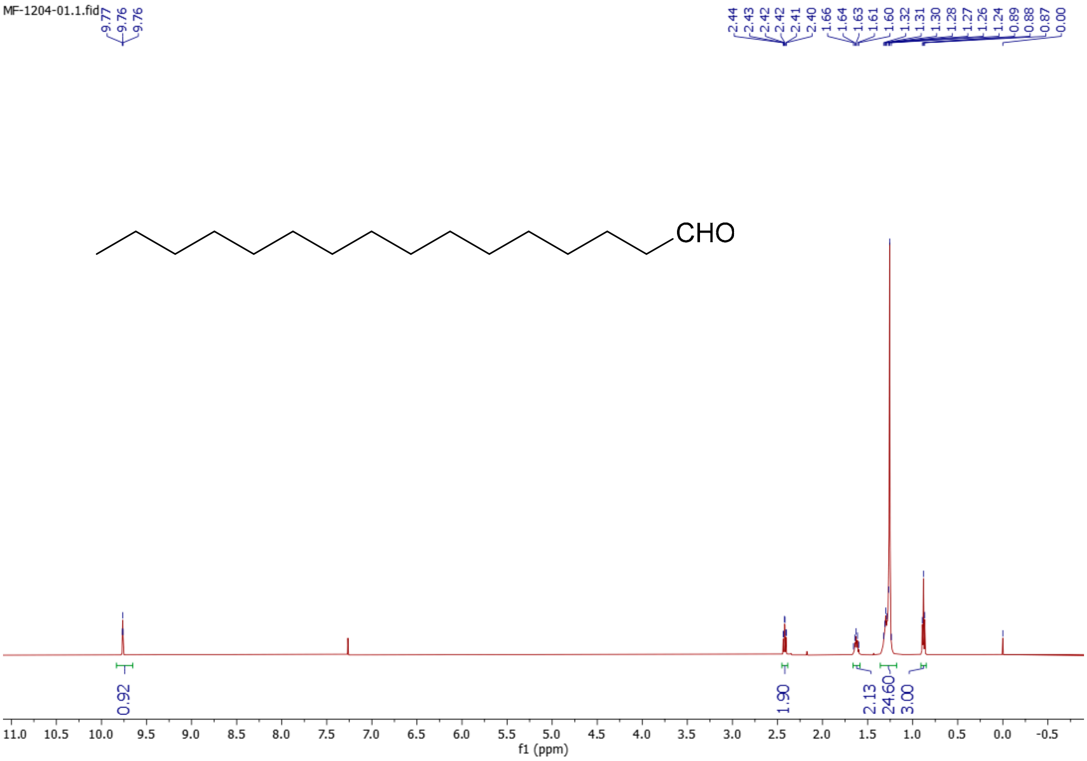

1-Nitroheptadecan-2-ol (15)

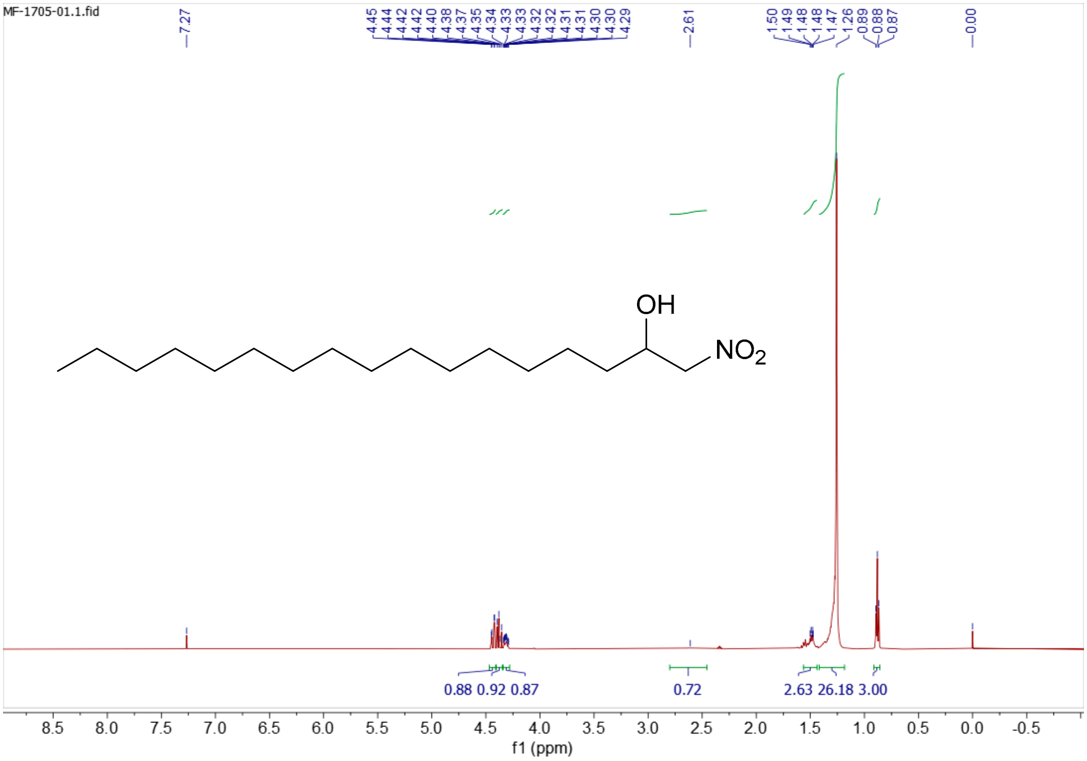

(*E*)-1-Nitroheptadec-1-ene (16)

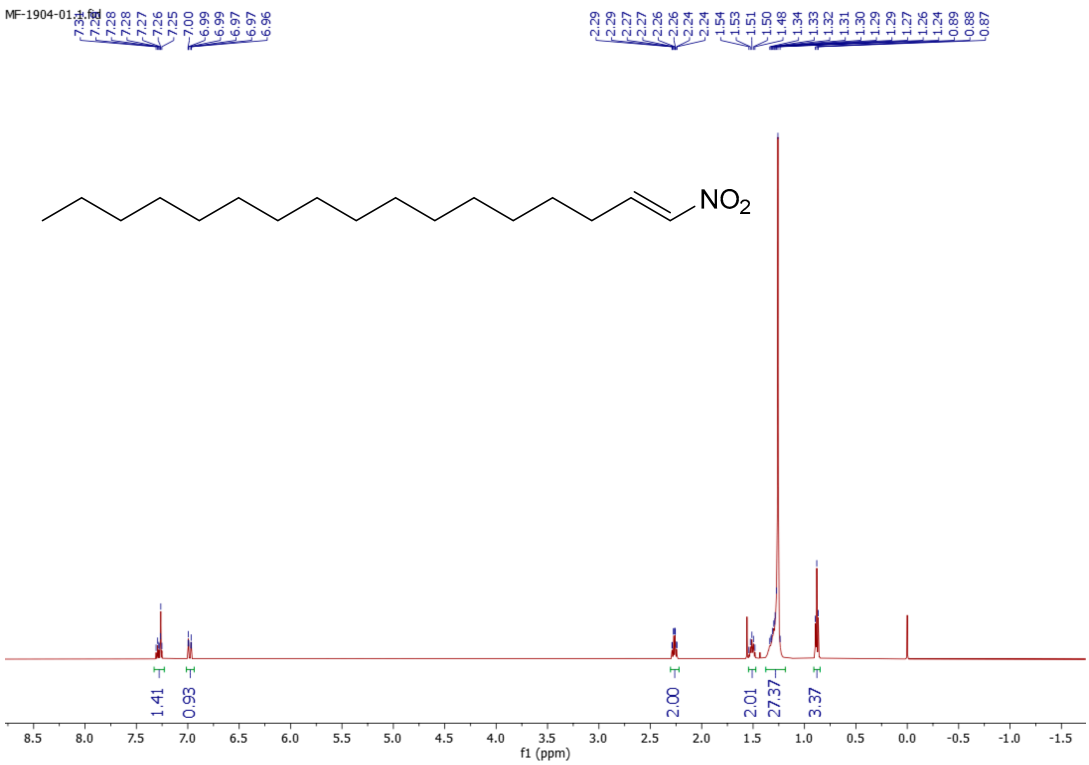

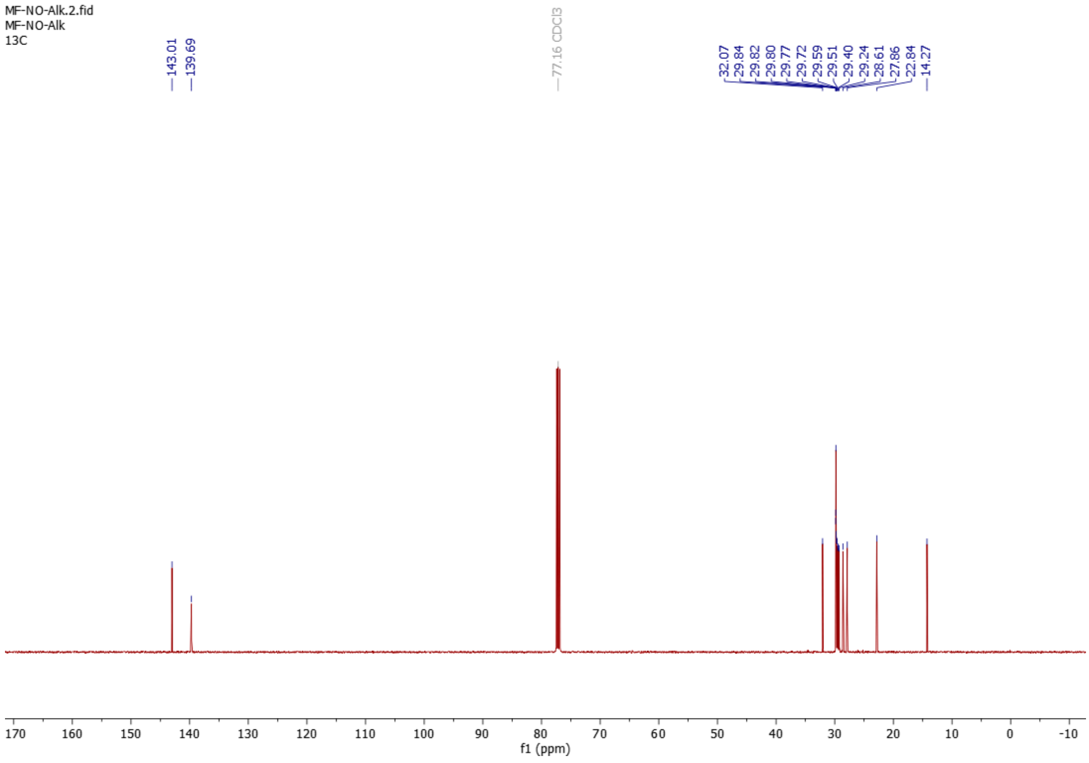

Methyl 4-((*tert*-butoxycarbonyl)amino)-2-((*R*)-2-nitro-1-phenylethyl)-3-oxobutanoate (17a)

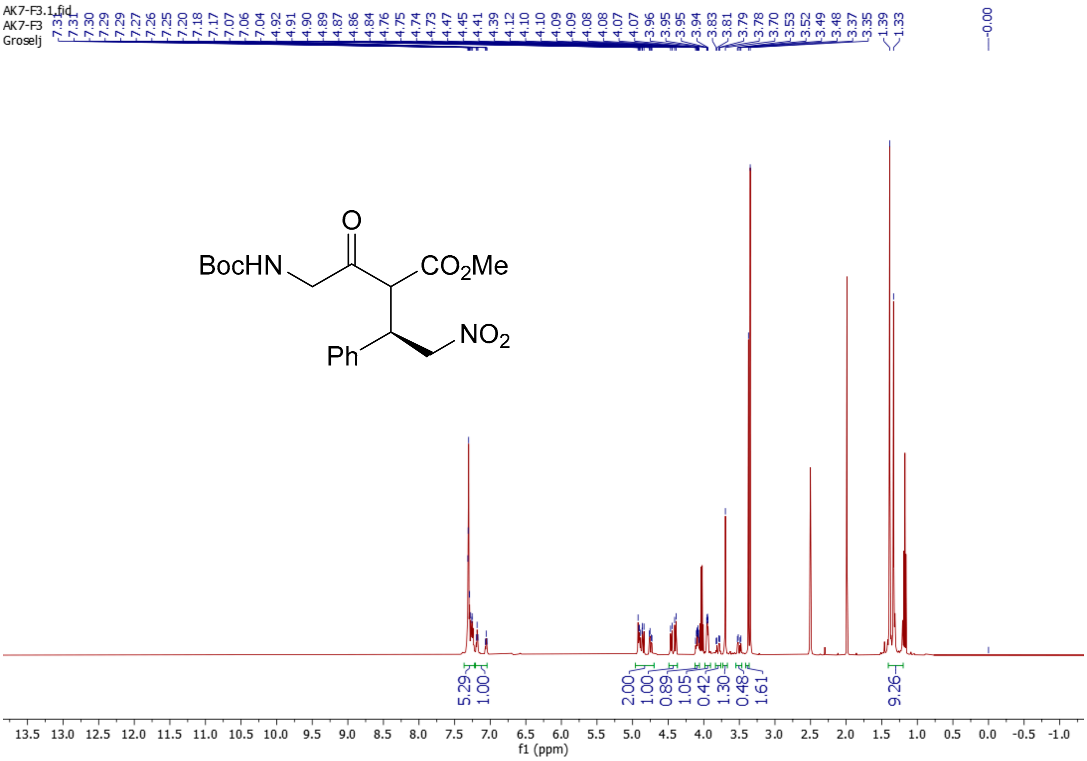

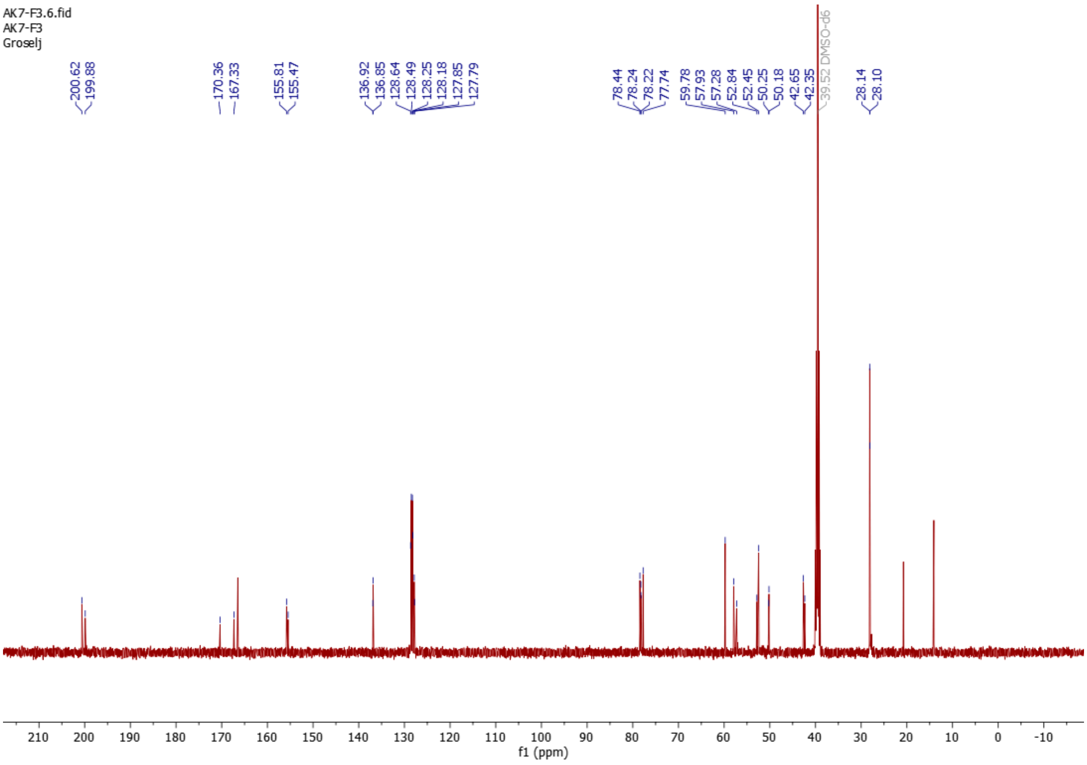

Methyl 5-((*tert*-butoxycarbonyl)amino)-2-((*R*)-2-nitro-1-phenylethyl)-3-oxopentanoate (17b)

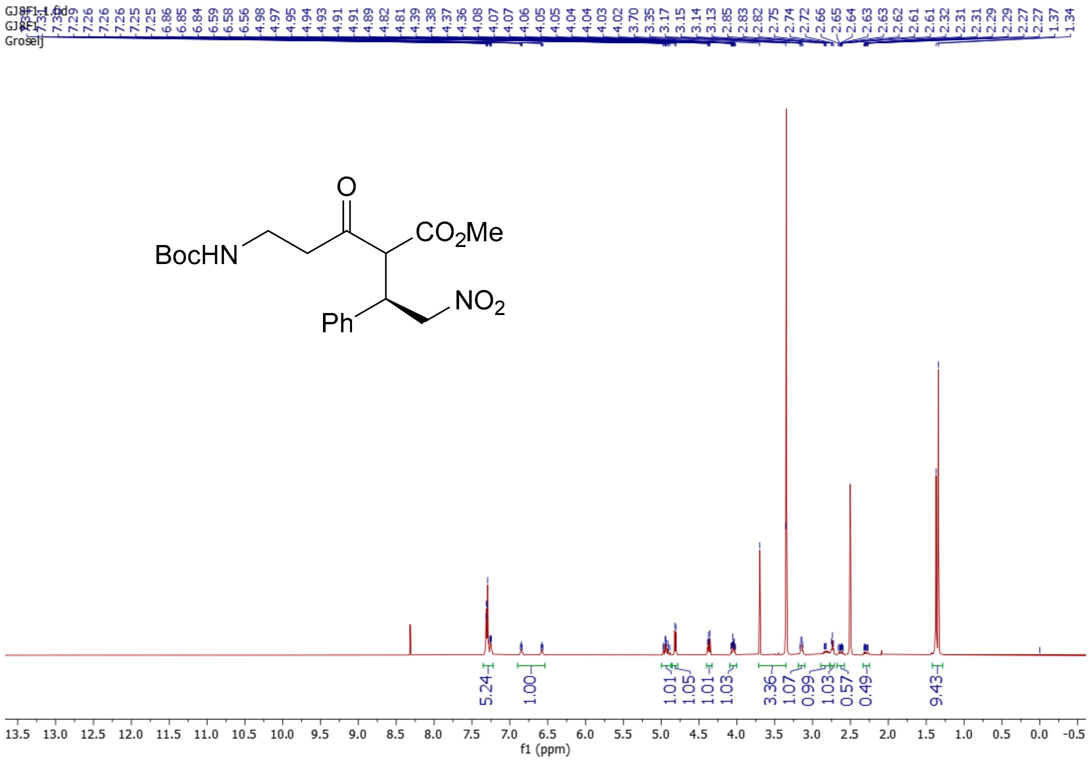

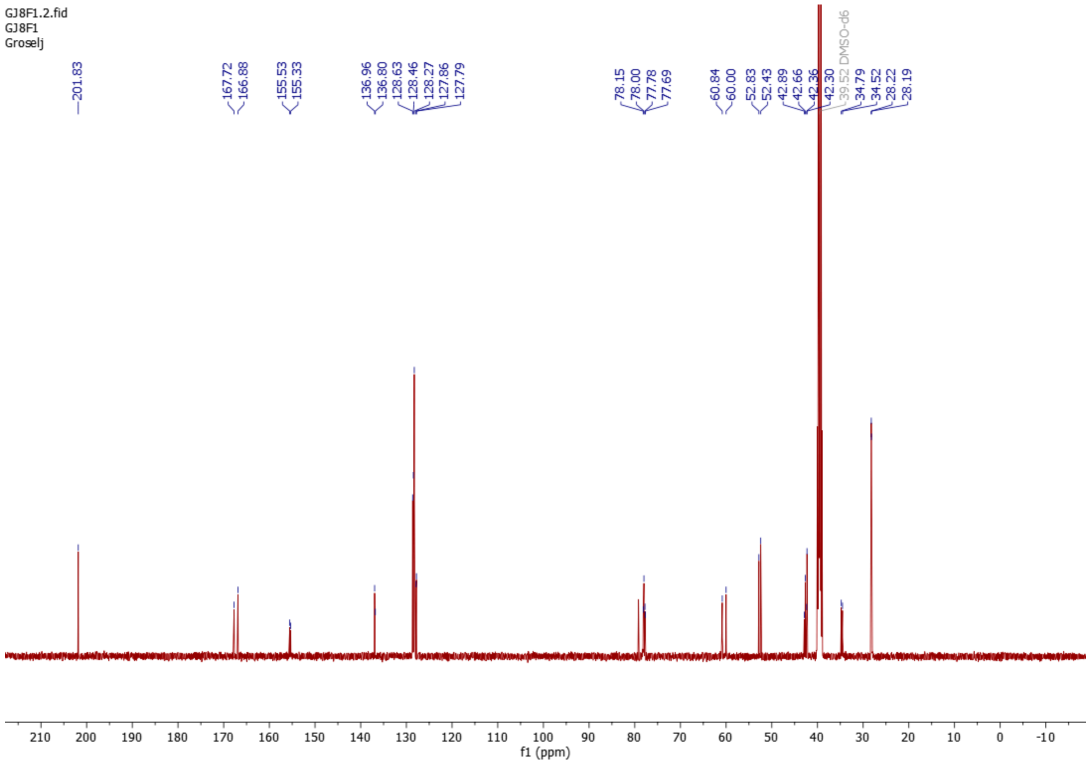

Methyl 2-(3-((*tert*-butoxycarbonyl)amino)propanoyl)-3-(nitromethyl)octadecanoate (*rac*-17c)

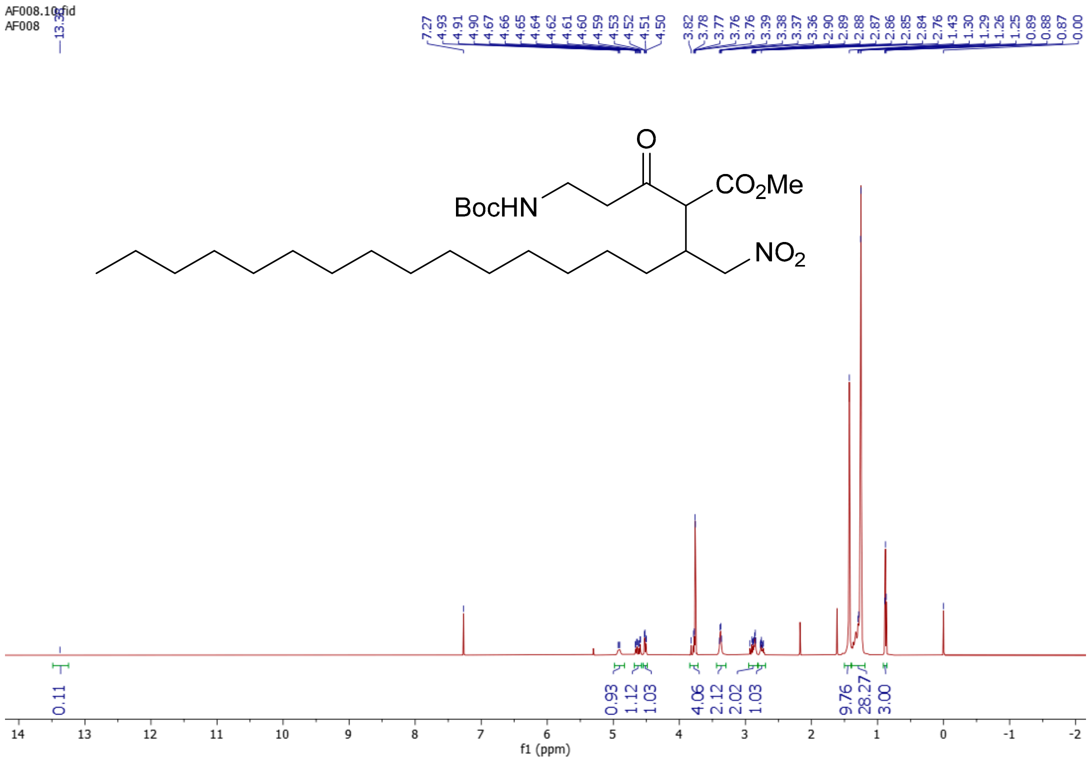

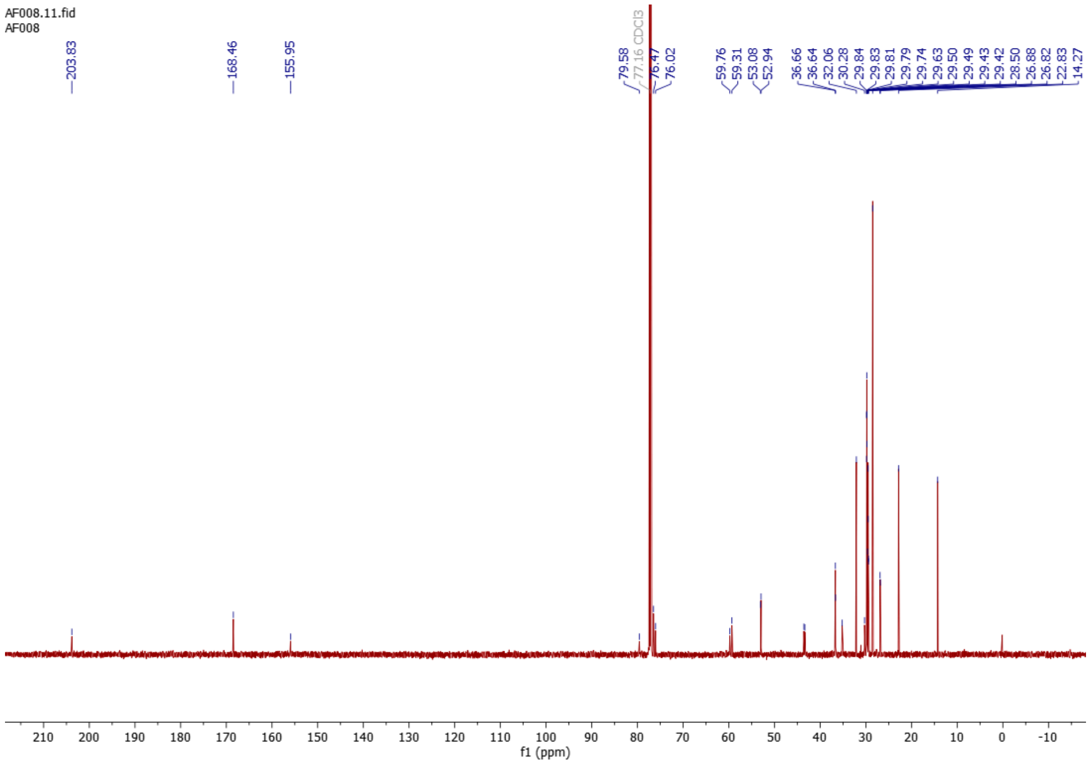

Methyl 4-((3-methylbut-2-en-1-yl)oxy)-2-((*R*)-2-nitro-1-phenylethyl)-3-oxobutanoate (17d)

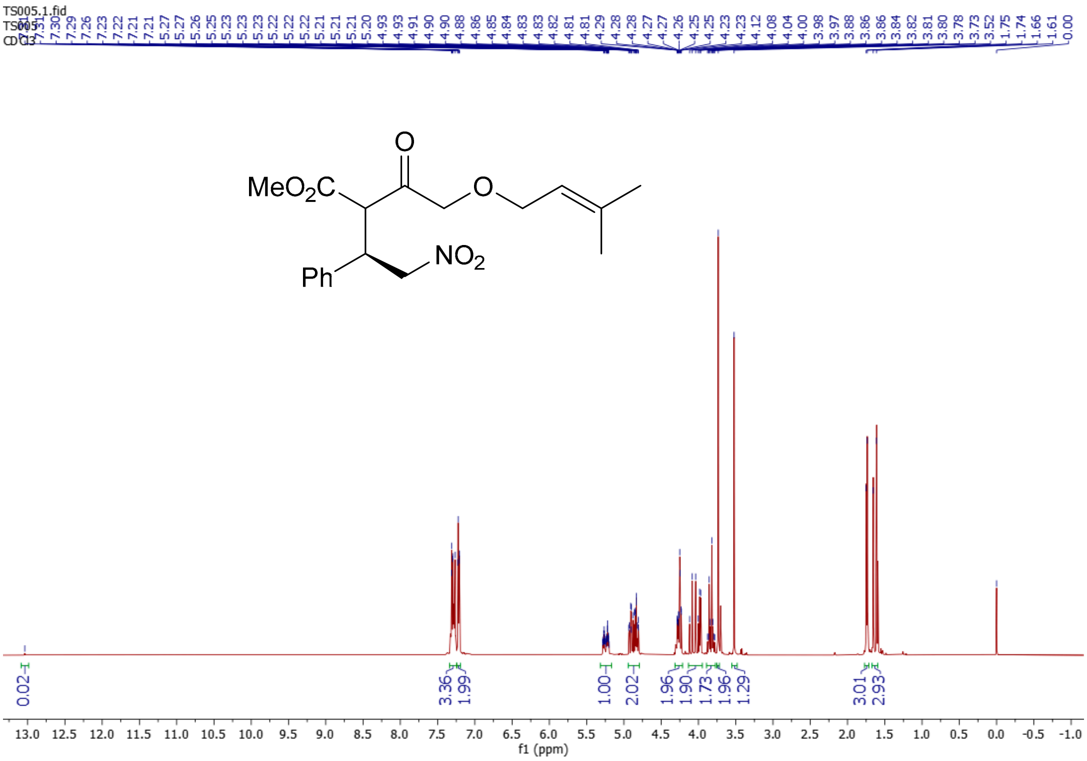

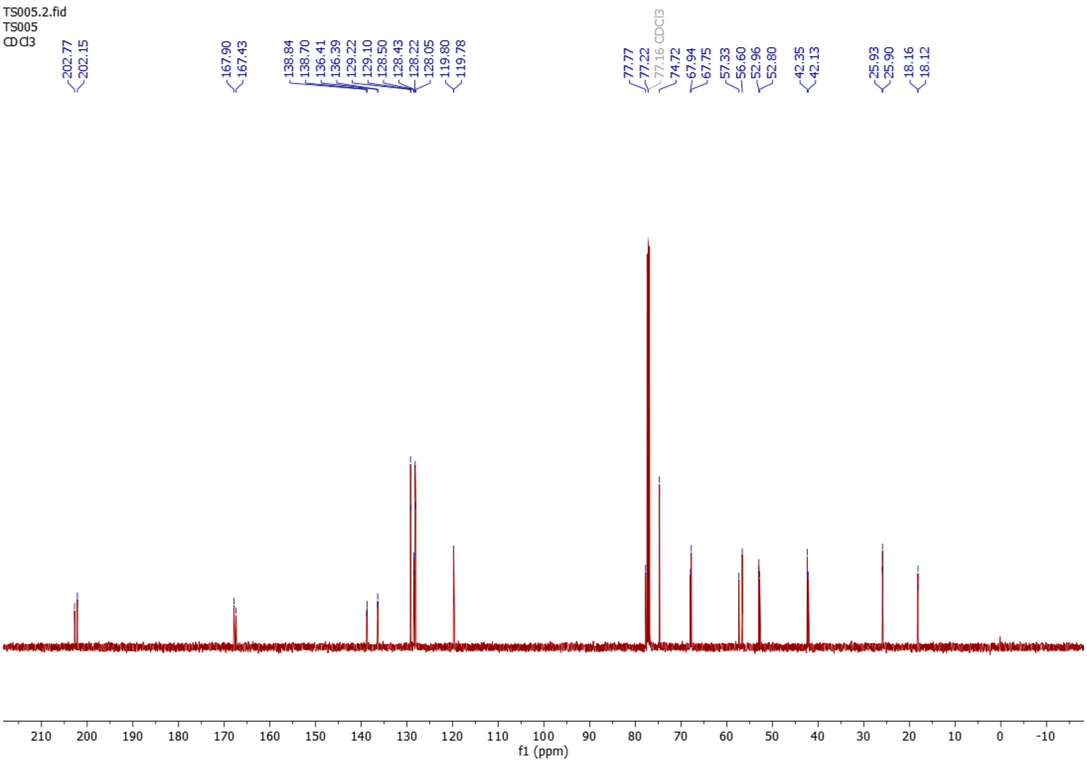

Methyl 2-(2-((3-methylbut-2-en-1-yl)oxy)acetyl)-3-(nitromethyl)octadecanoate (*rac*-17e)

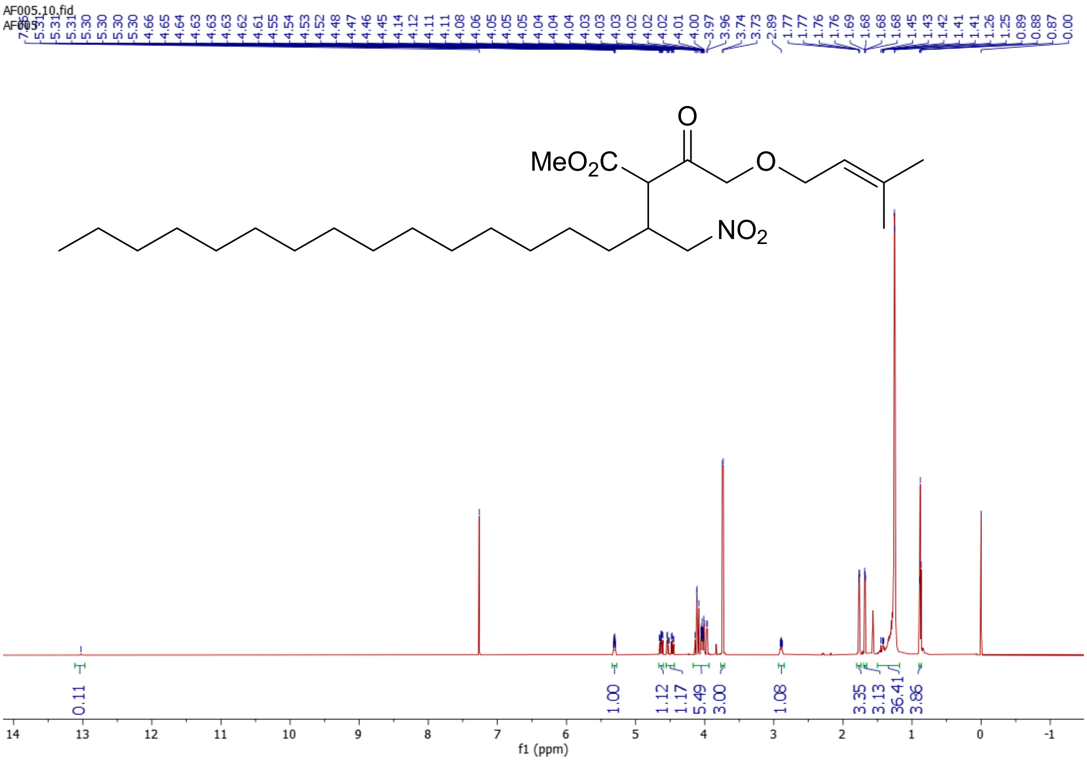

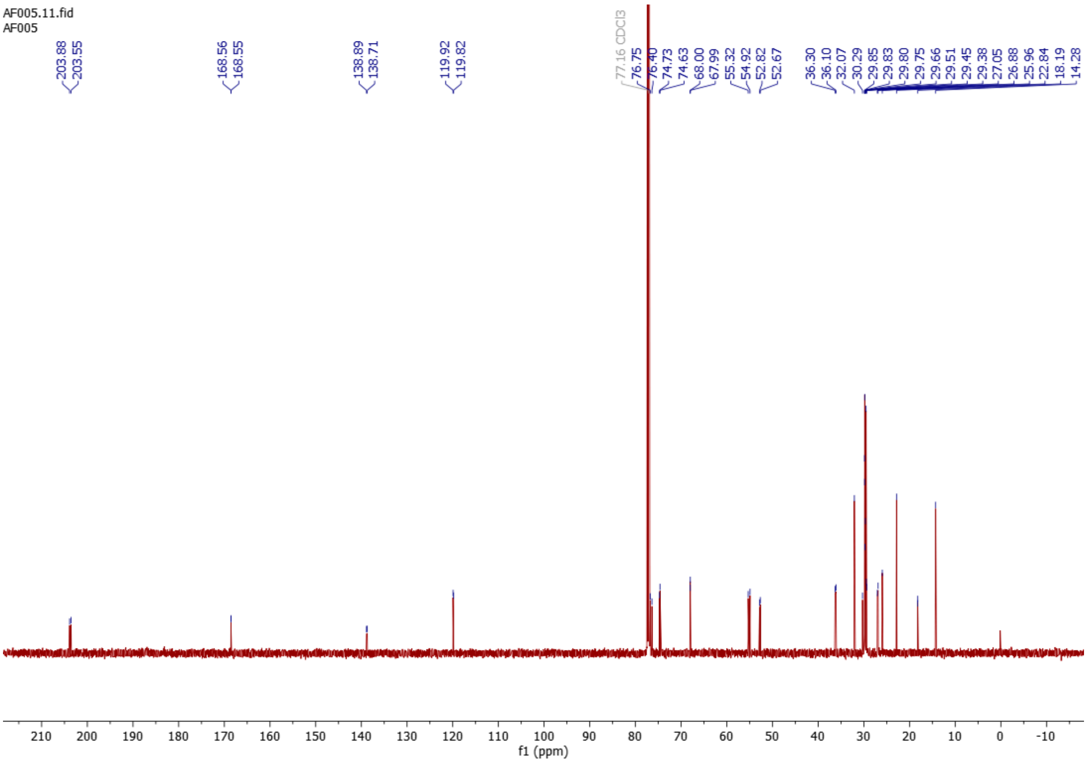

Methyl 2-((*R*)-2-nitro-1-phenylethyl)-3-oxooctadecanoate (17f)

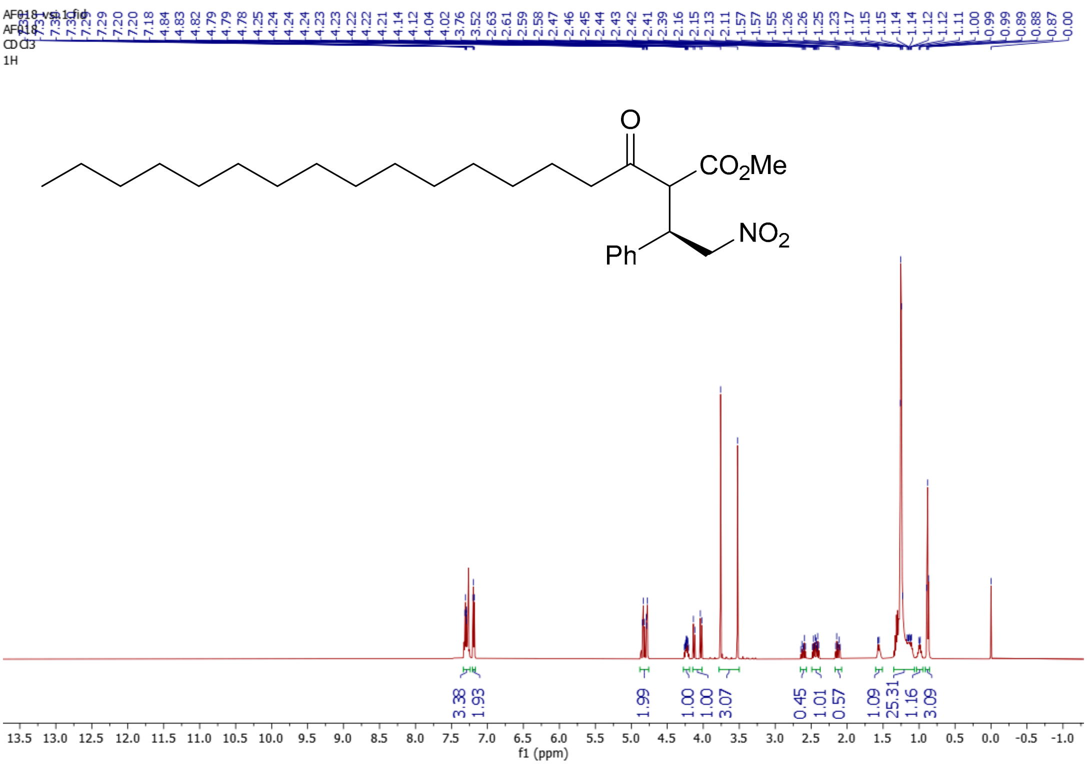

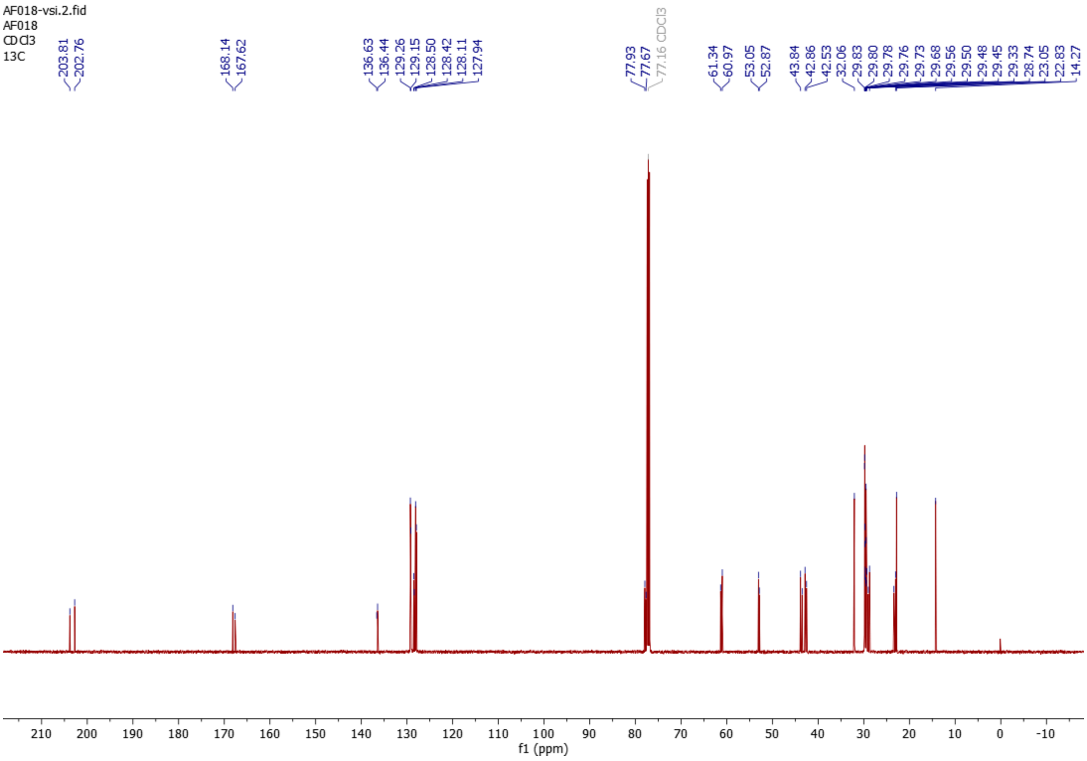

Methyl 2-(1-nitroheptadecan-2-yl)-3-oxooctadecanoate (*rac*-17g)

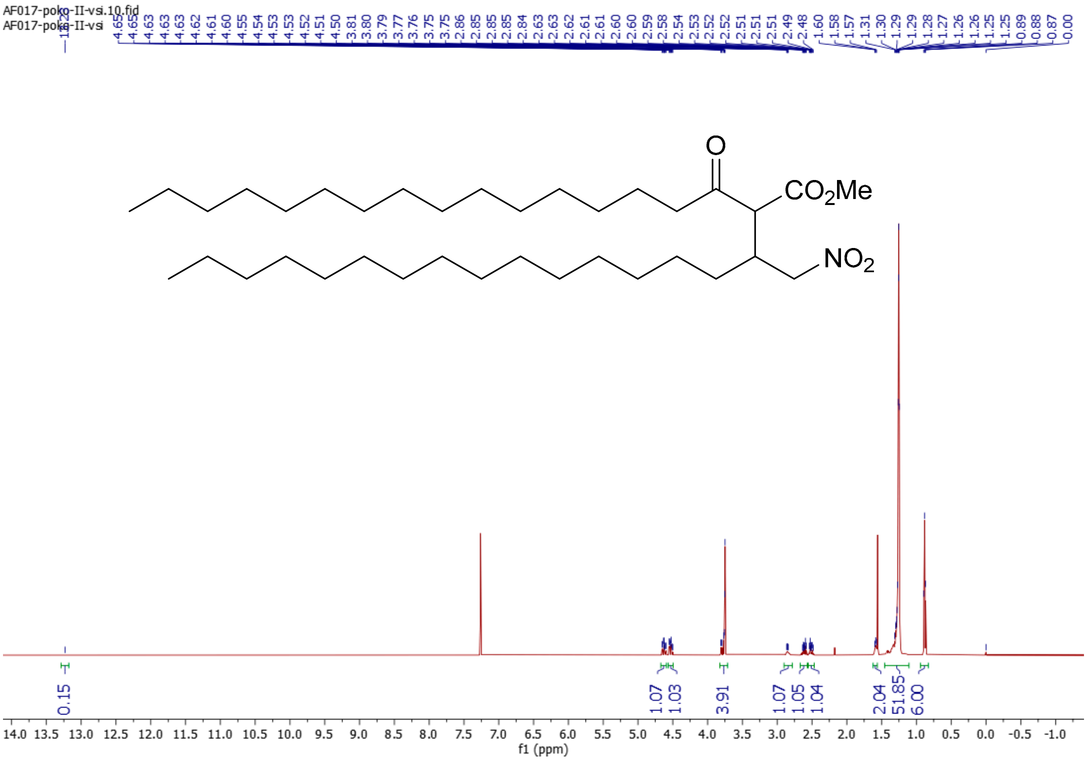

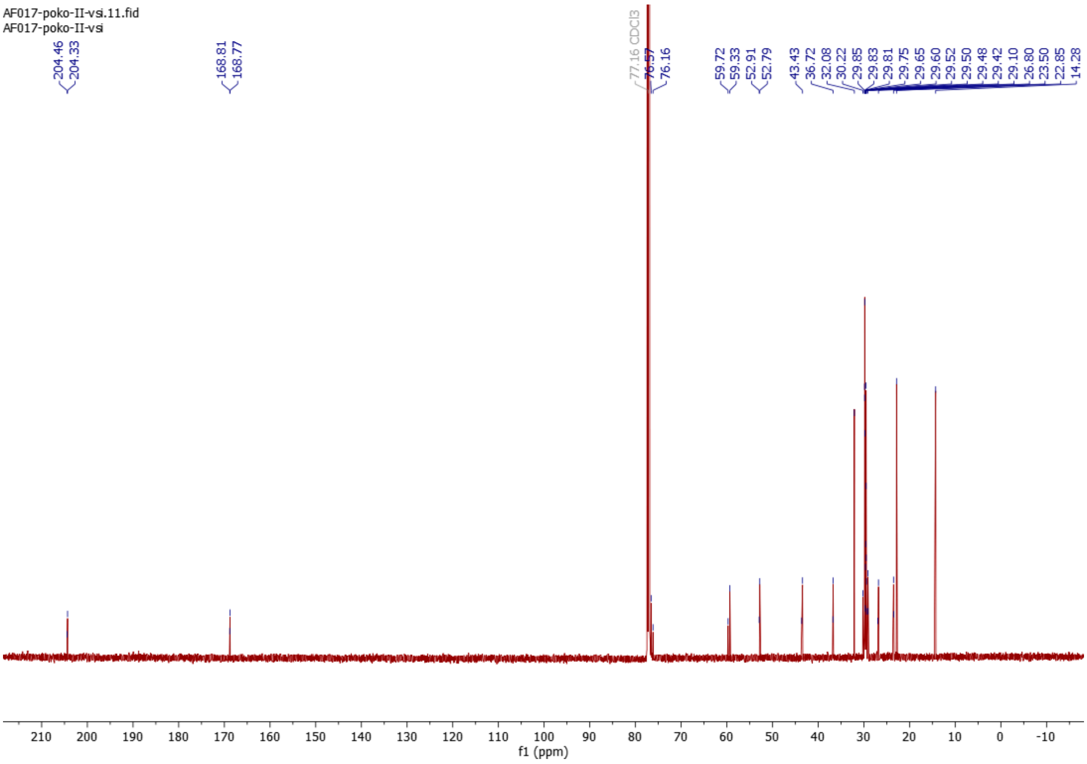

Methyl 2-((*R*)-2-nitro-1-phenylethyl)-3-oxoicosanoate (17h)

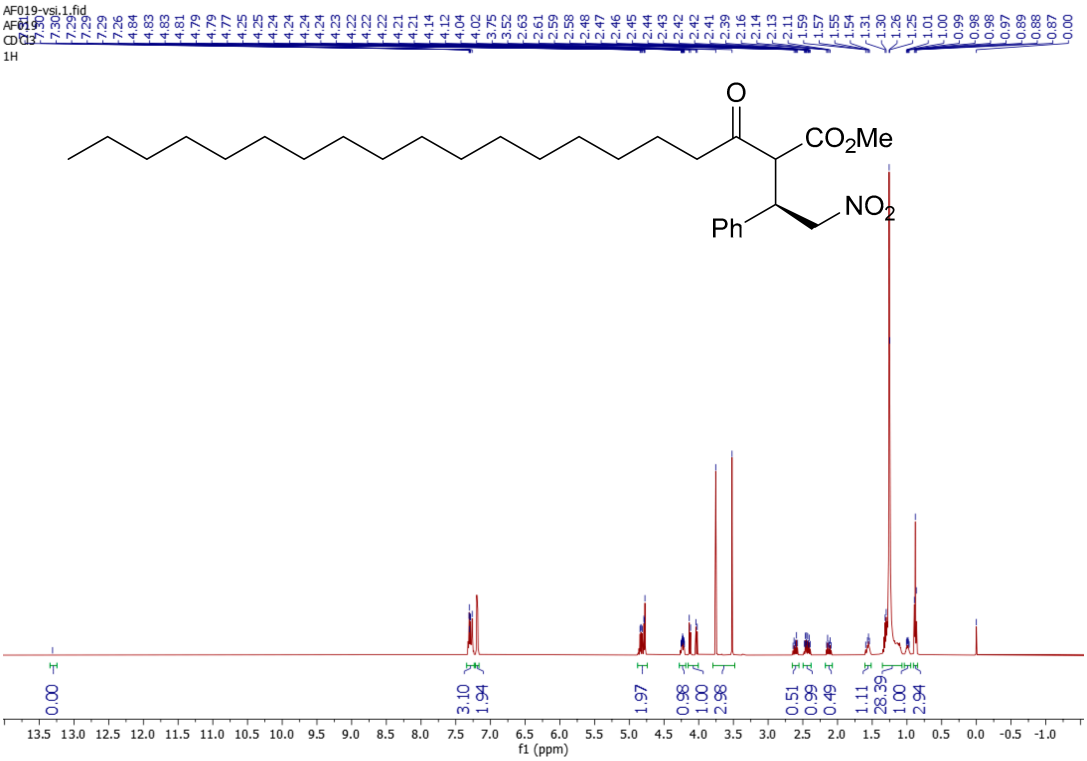

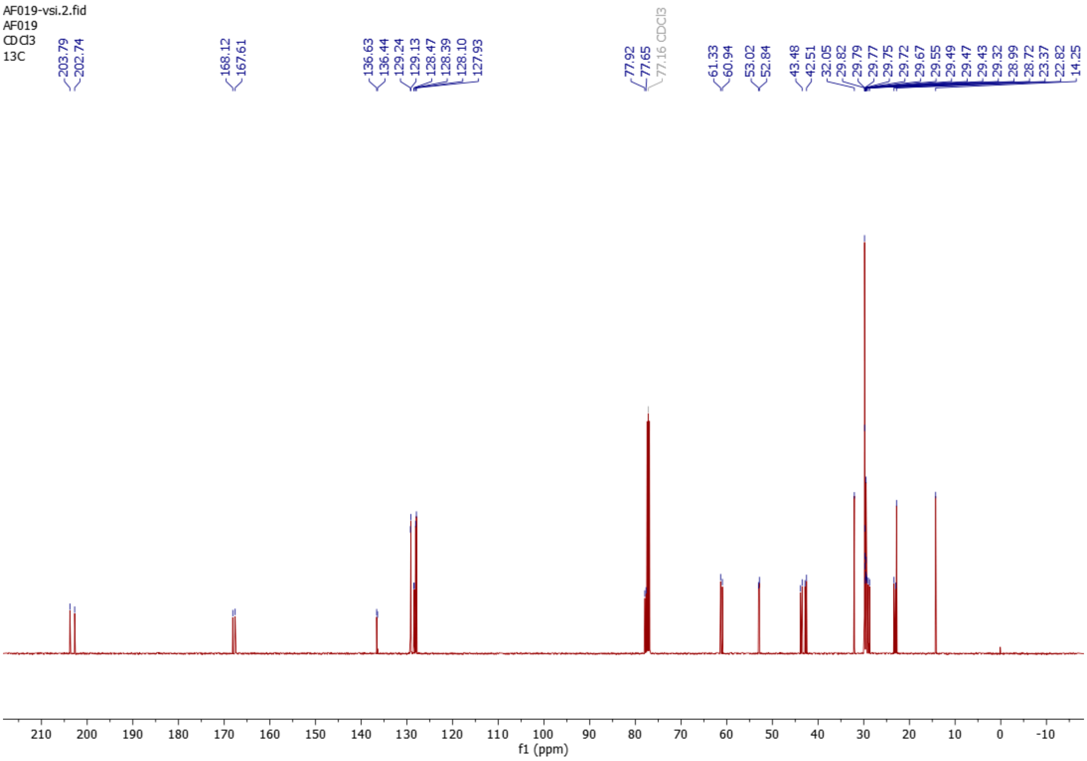

Methyl 2-(1-nitroheptadecan-2-yl)-3-oxoicosanoate (*rac*-17i)

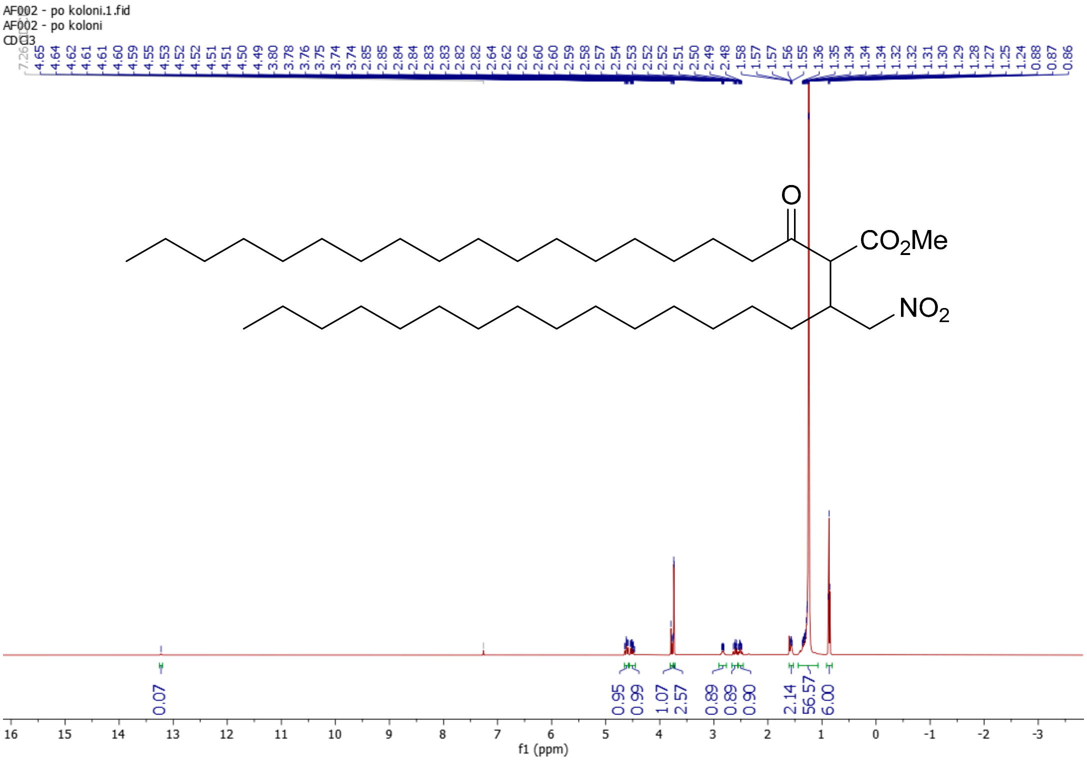

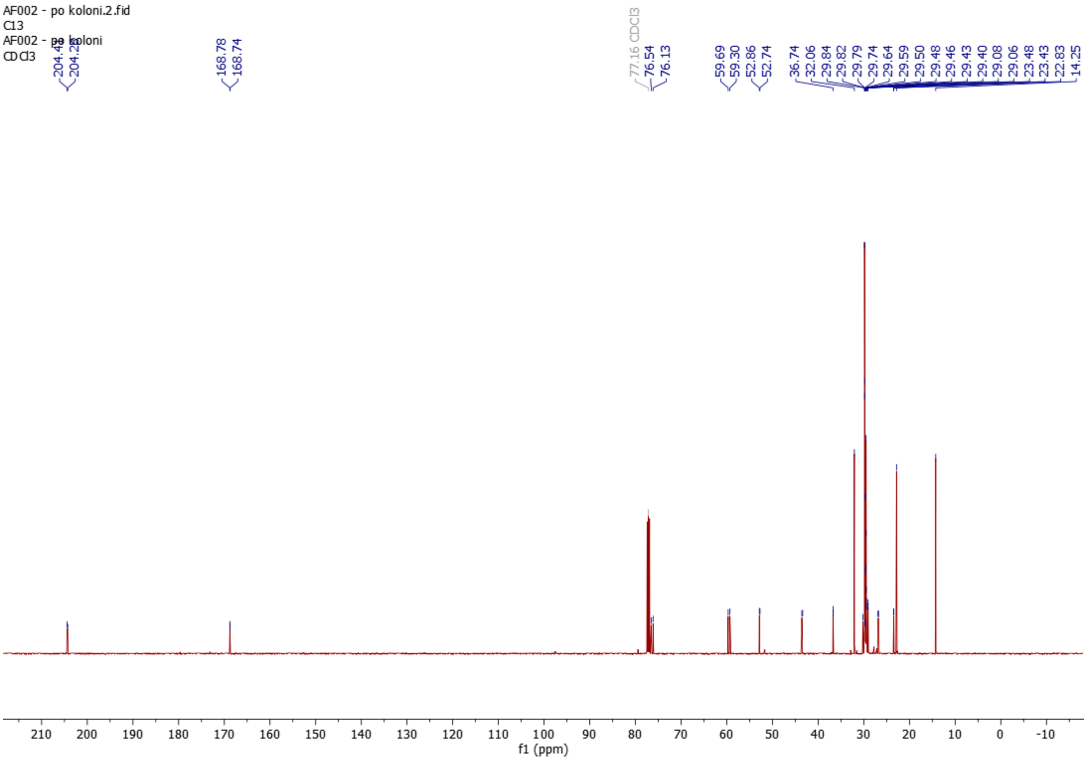

***tert*-Butyl 2-((*R*)-2-nitro-1-phenylethyl)-3-oxoicosanoate (17j)**

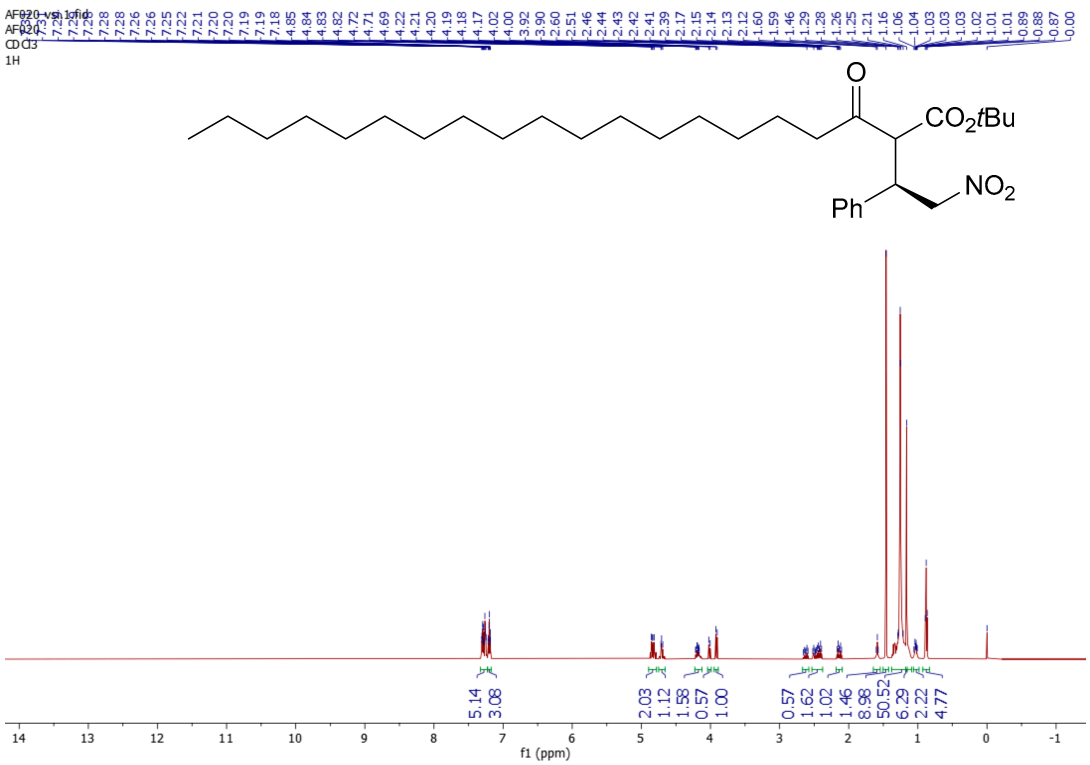

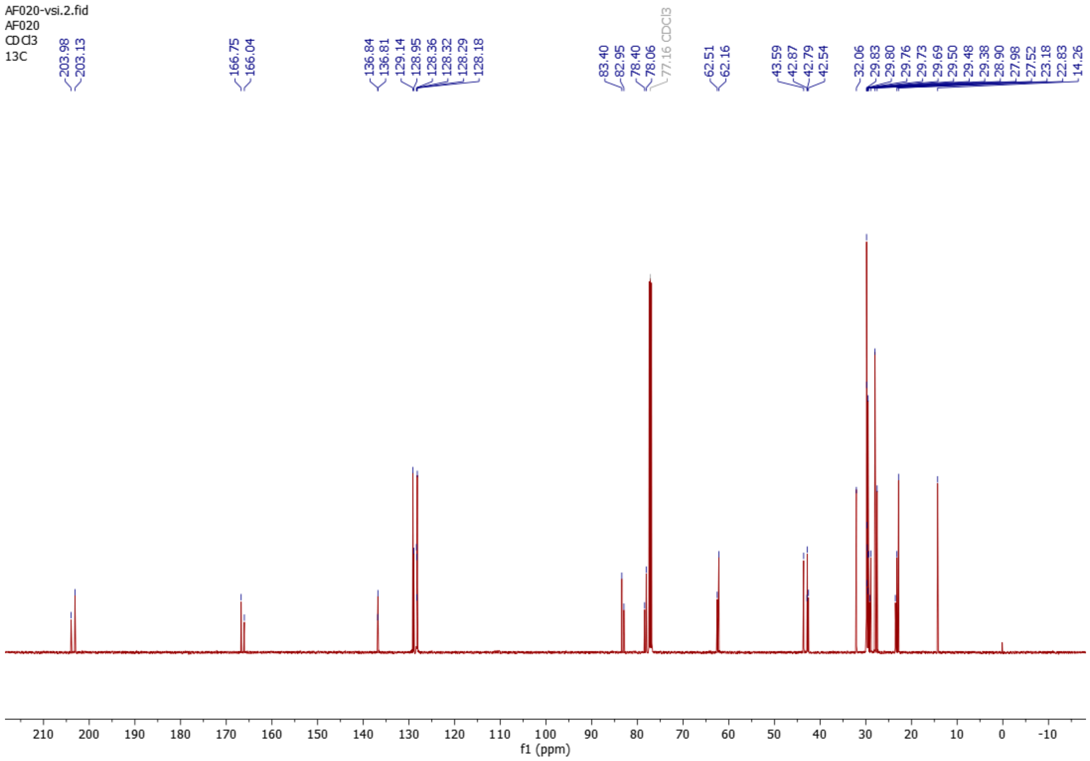

tert-Butyl 2-(1-nitroheptadecan-2-yl)-3-oxoicosanoate (*rac*-17k)

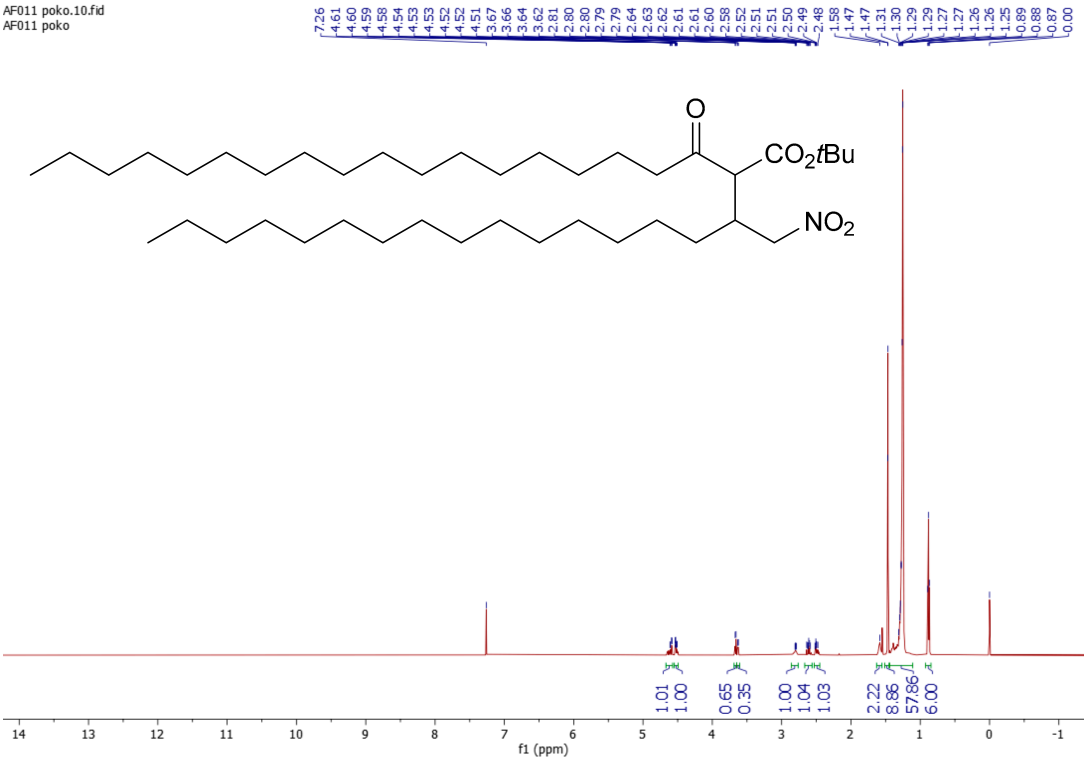

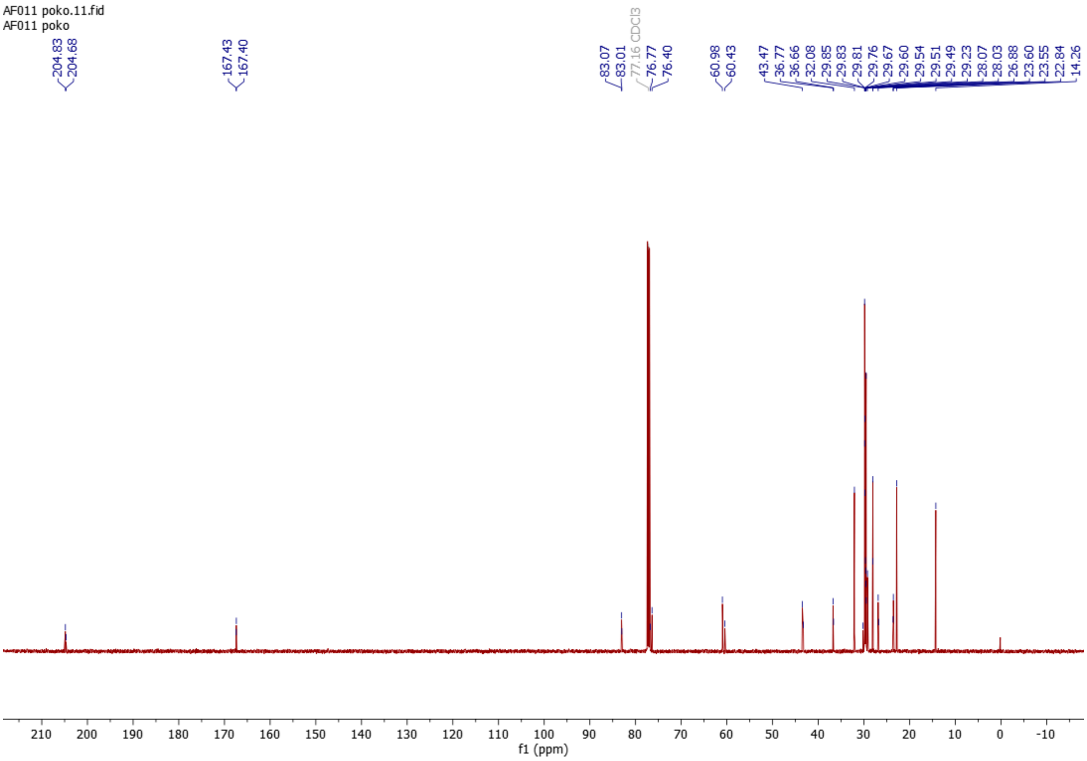

Methyl (11*Z*,14*Z*)-2-((*R*)-2-nitro-1-phenylethyl)-3-oxoicosa-11,14-dienoate (17l)

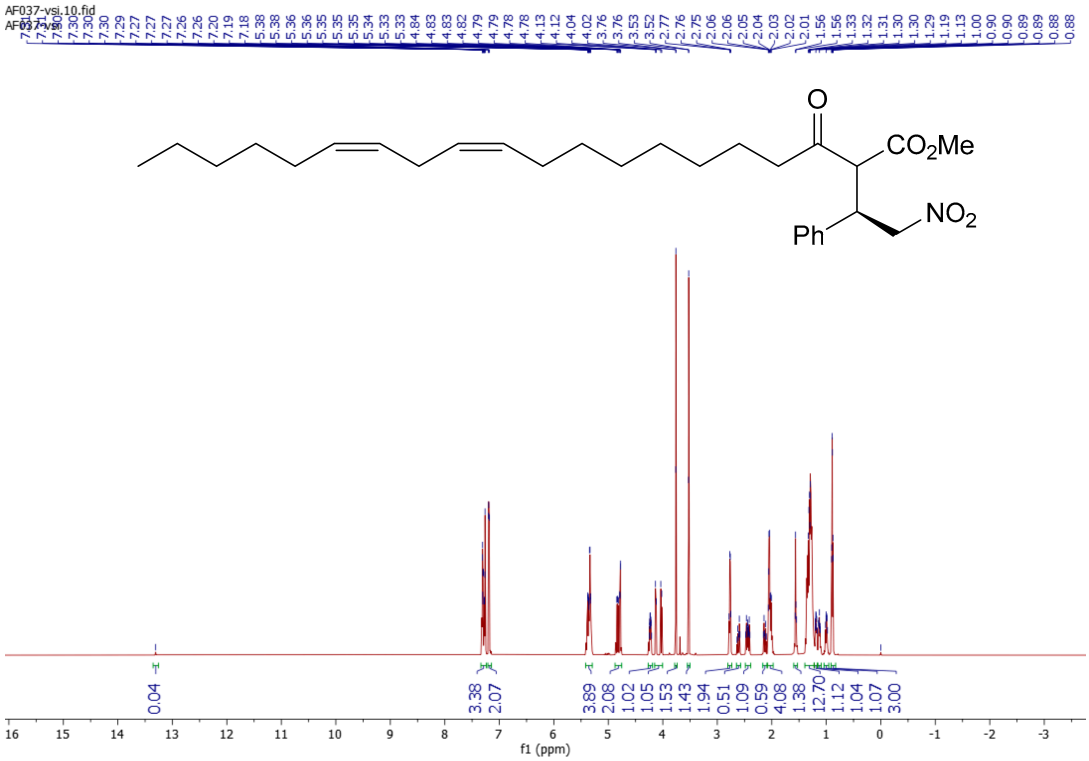

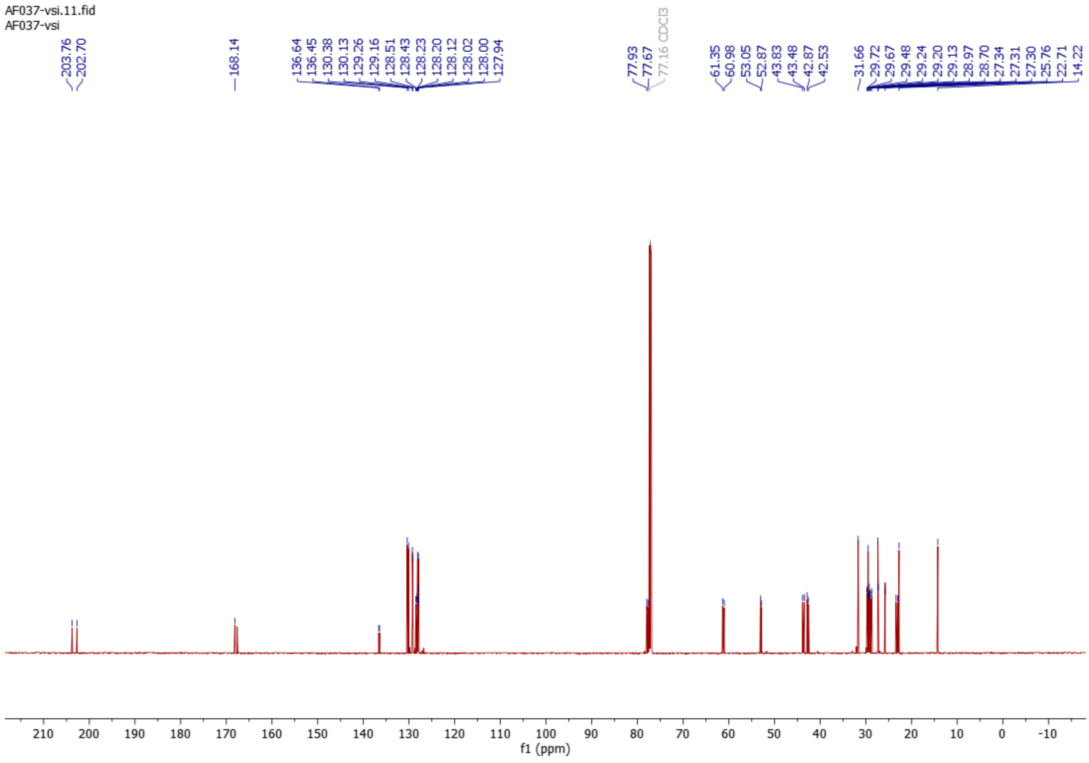

Methyl (4*S*)-7-((*tert*-butoxycarbonyl)amino)-2-(2-nitro-1-phenylethyl)-3-oxo-4-stearamidoheptanoate (17m)

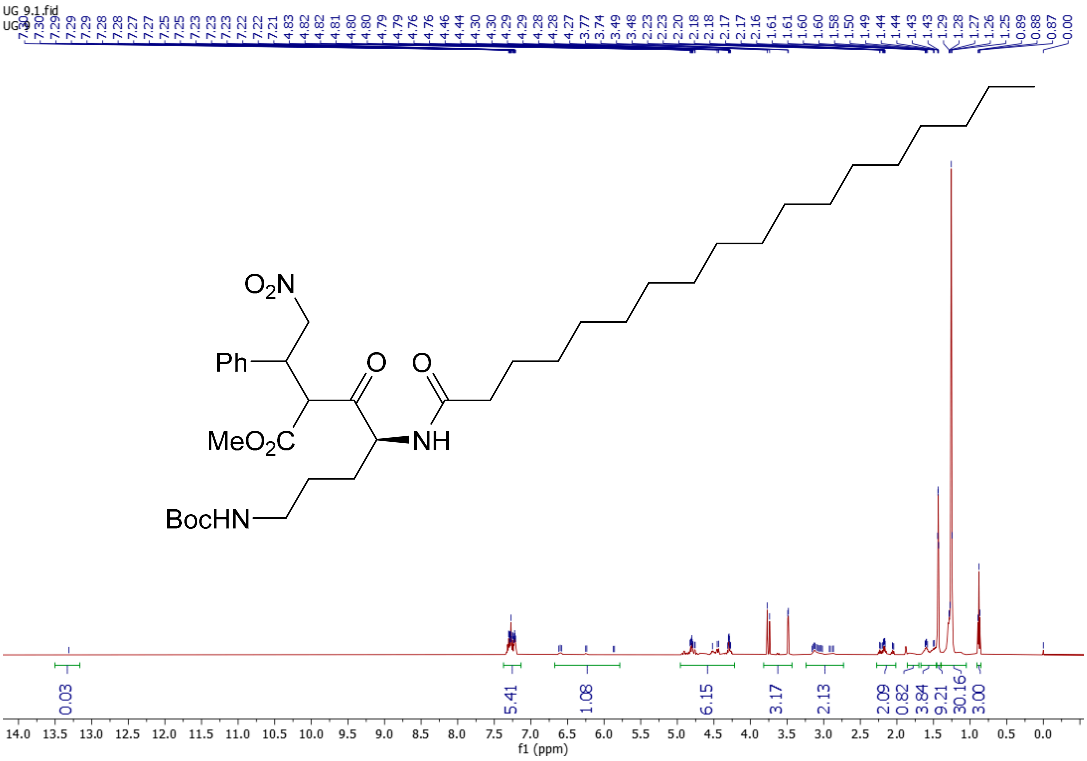

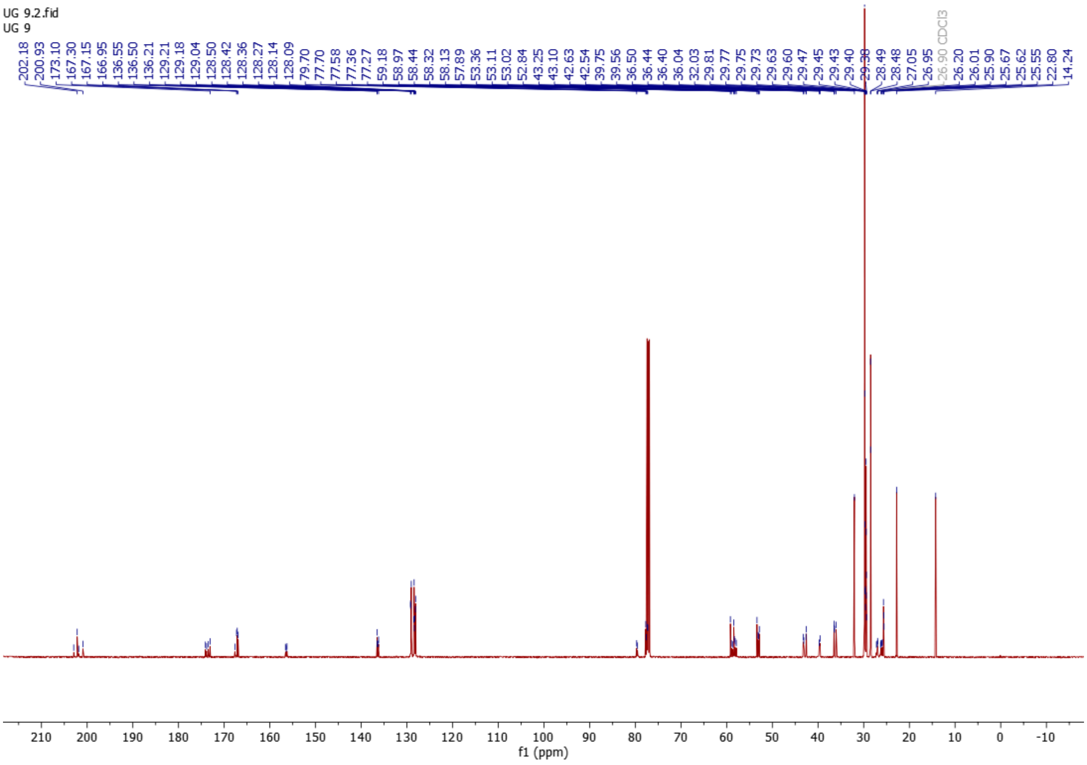

Methyl 2-((*S*)-5-((*tert*-butoxycarbonyl)amino)-2-stearamidopentanoyl)-3-(nitromethyl)octadecanoate (17n)

With catalyst **X**.

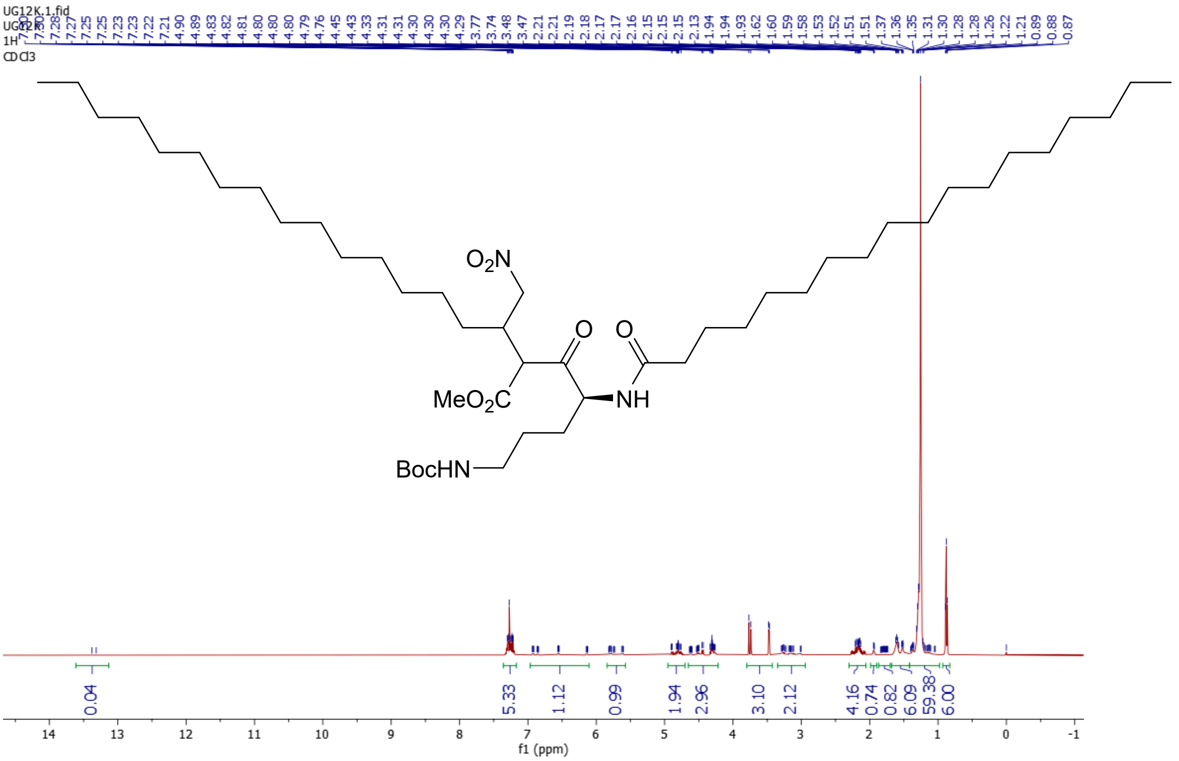

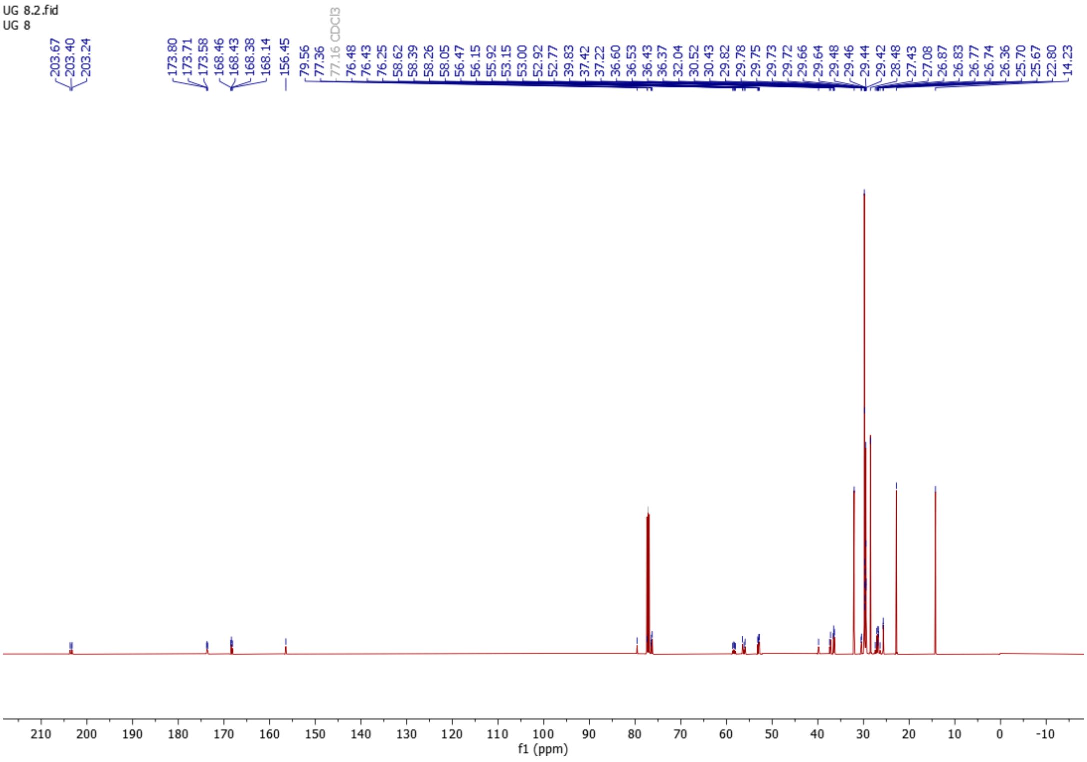

With catalyst **VIII**.

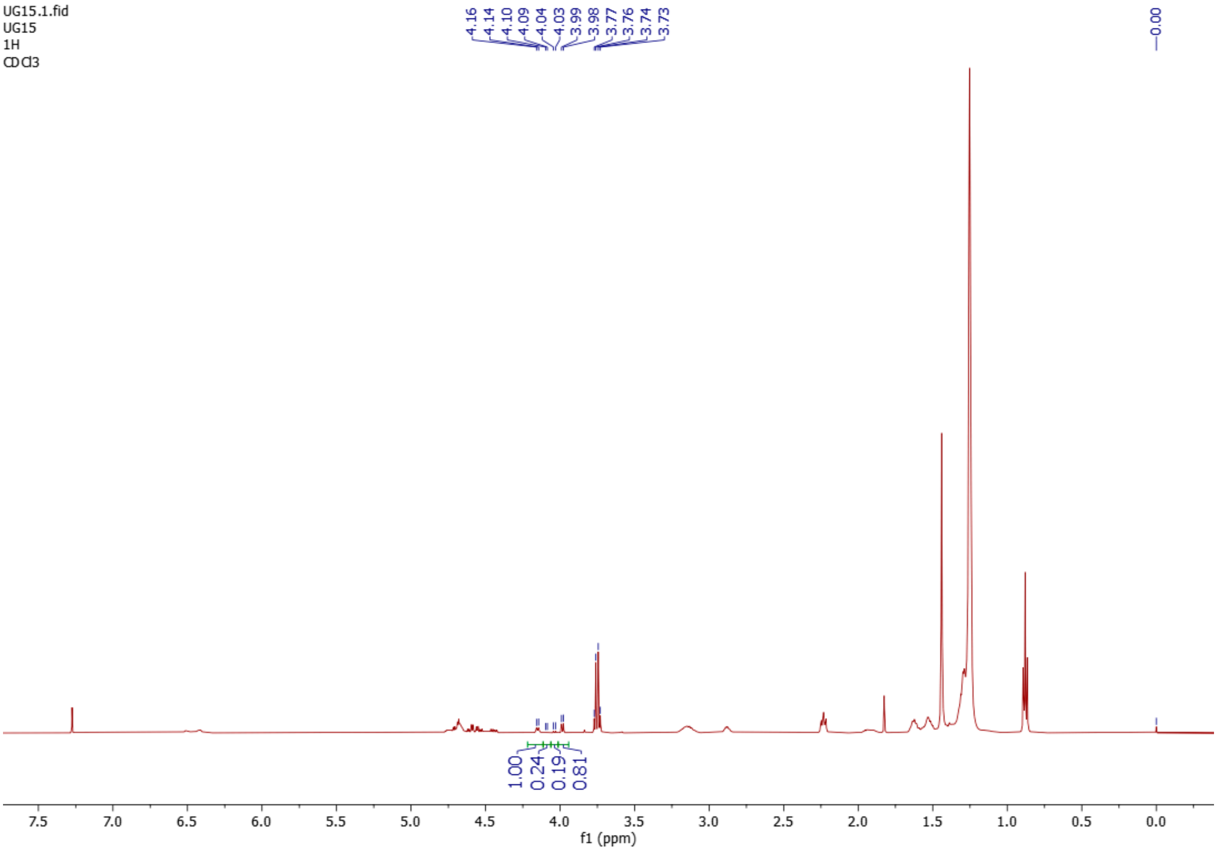

Methyl (4*S*)-2-(2-nitro-1-phenylethyl)-3-oxo-4,7-distearamidoheptanoate (17o)

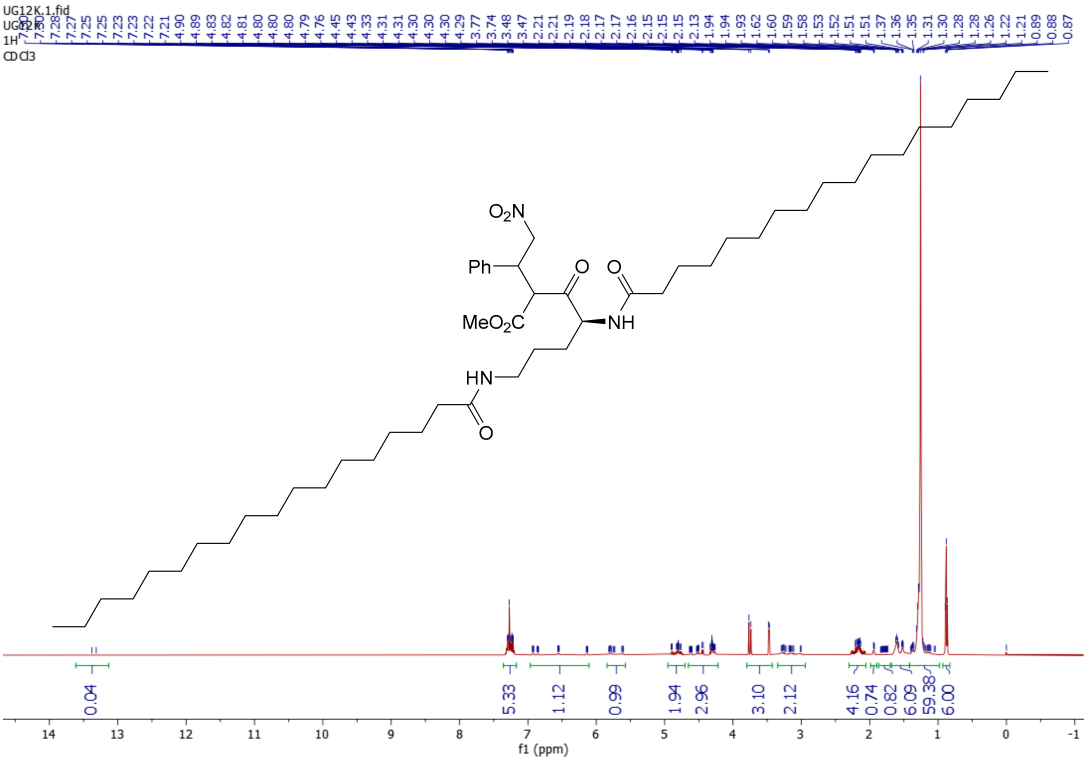

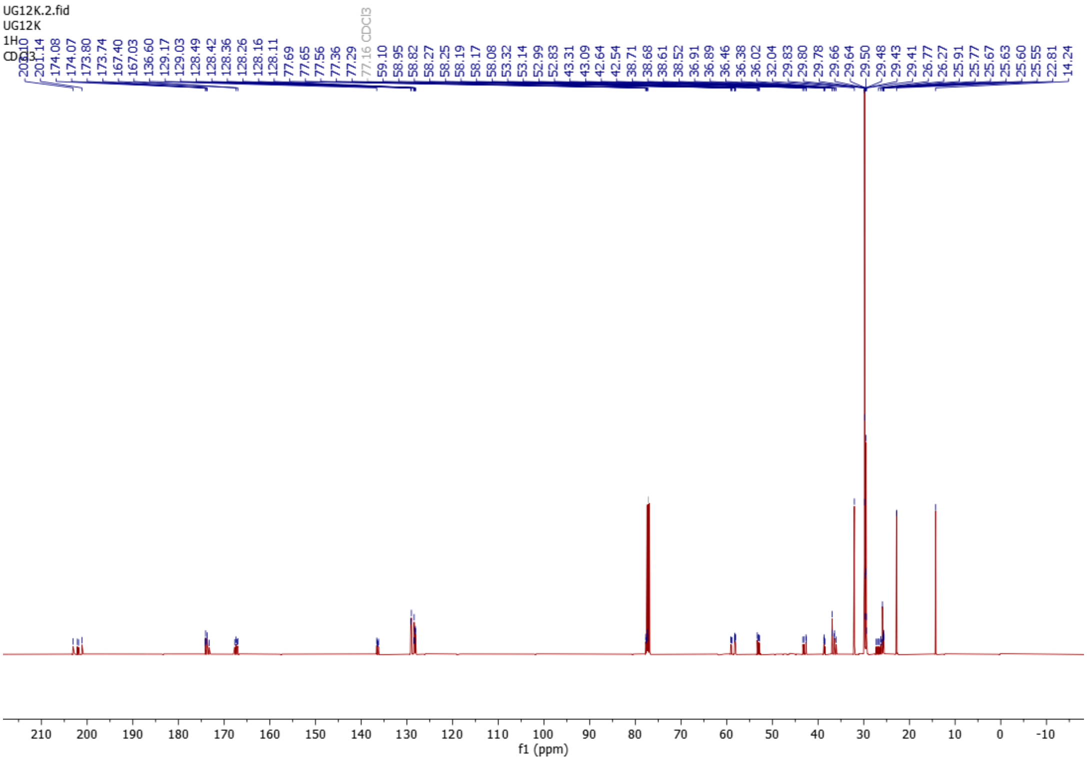

Synthesis of methyl 2-((*S*)-2,5-distearamidopentanoyl)-3-(nitromethyl)octadecanoate (17p)

With catalyst **X**.

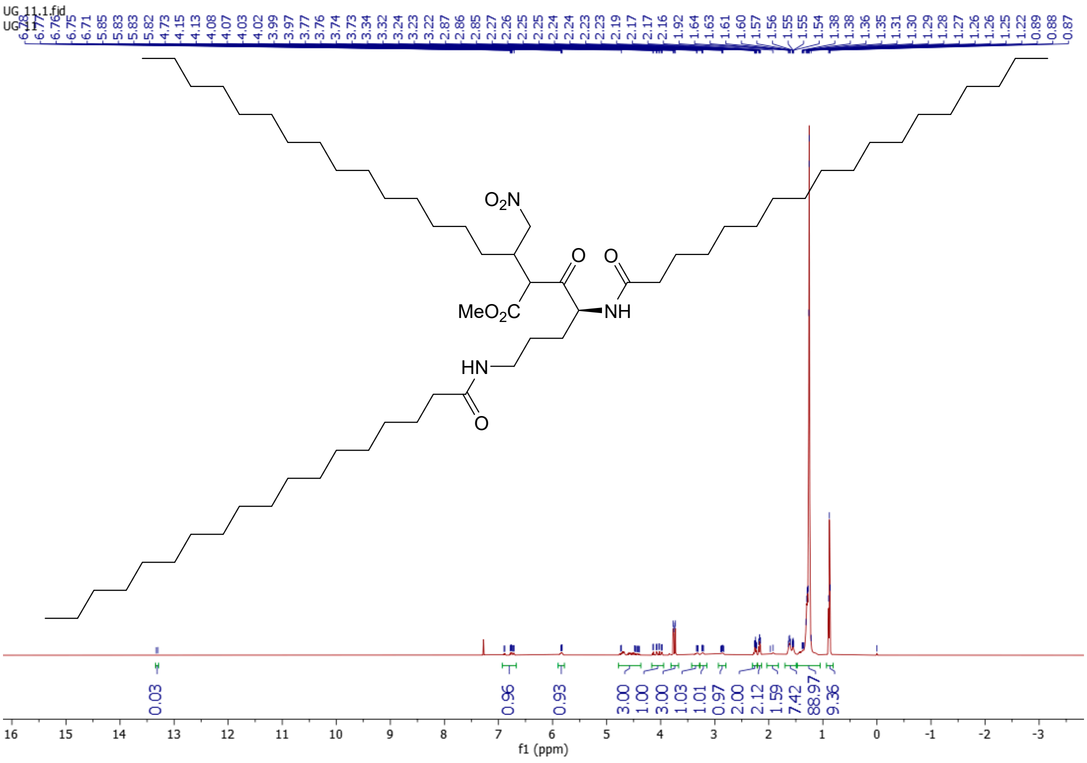

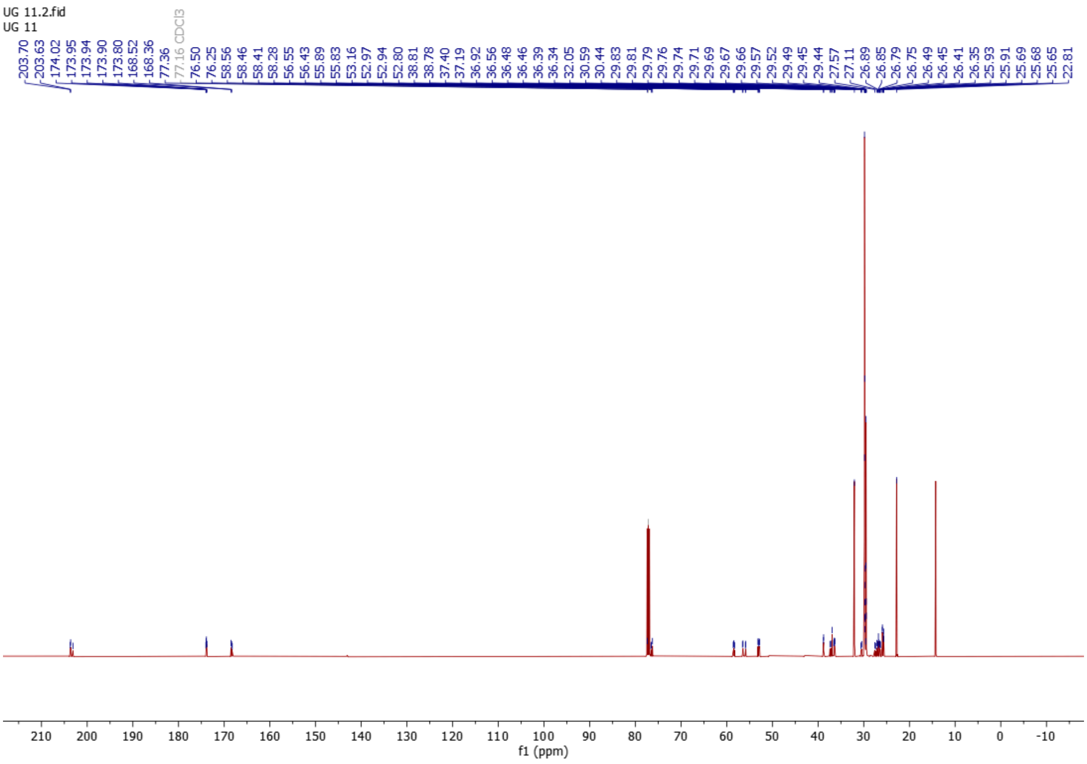

With catalyst **VIII**.

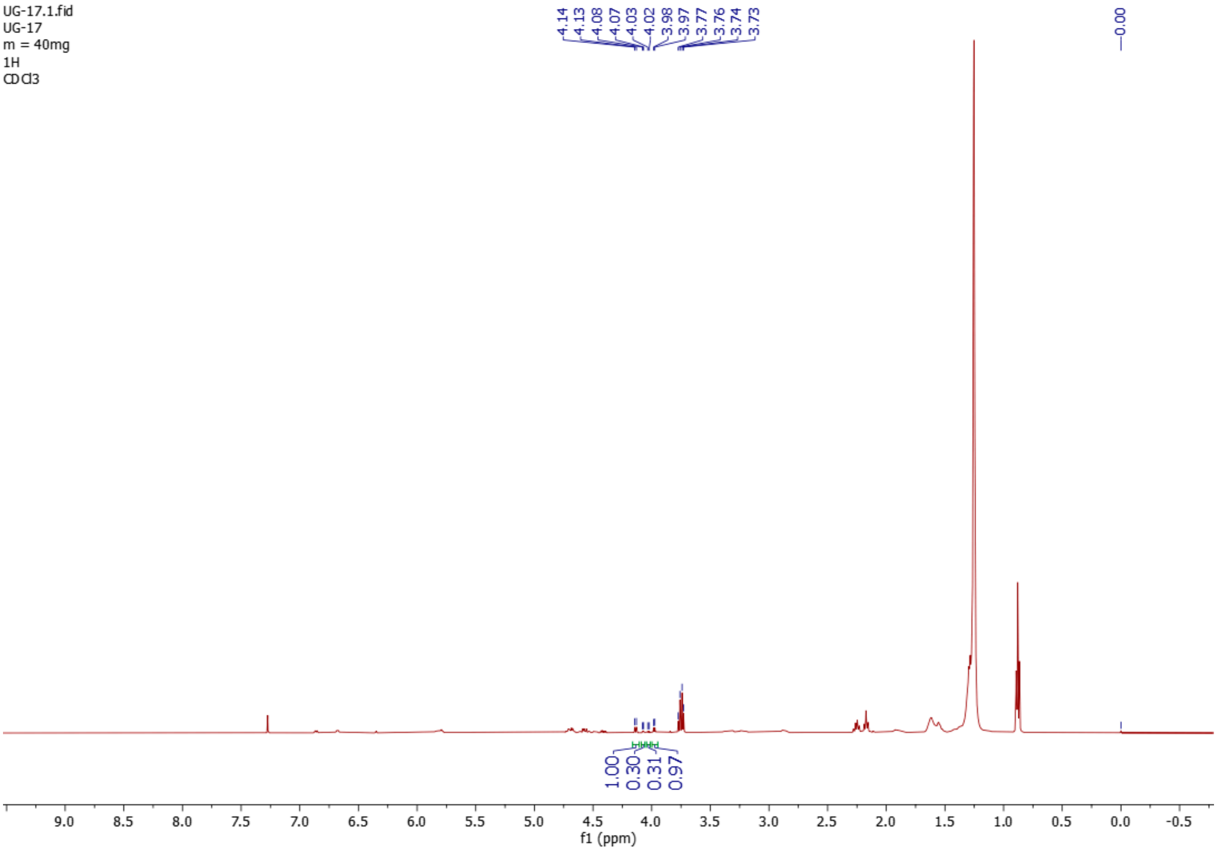

1-(*tert*-Butyl) 3-methyl (*S*)-5-benzyl-5-((*S*)-1-nitroheptadecan-2-yl)-4-oxo-4,5-dihydro-1*H*-pyrrole-1,3-dicarboxylate (18a)

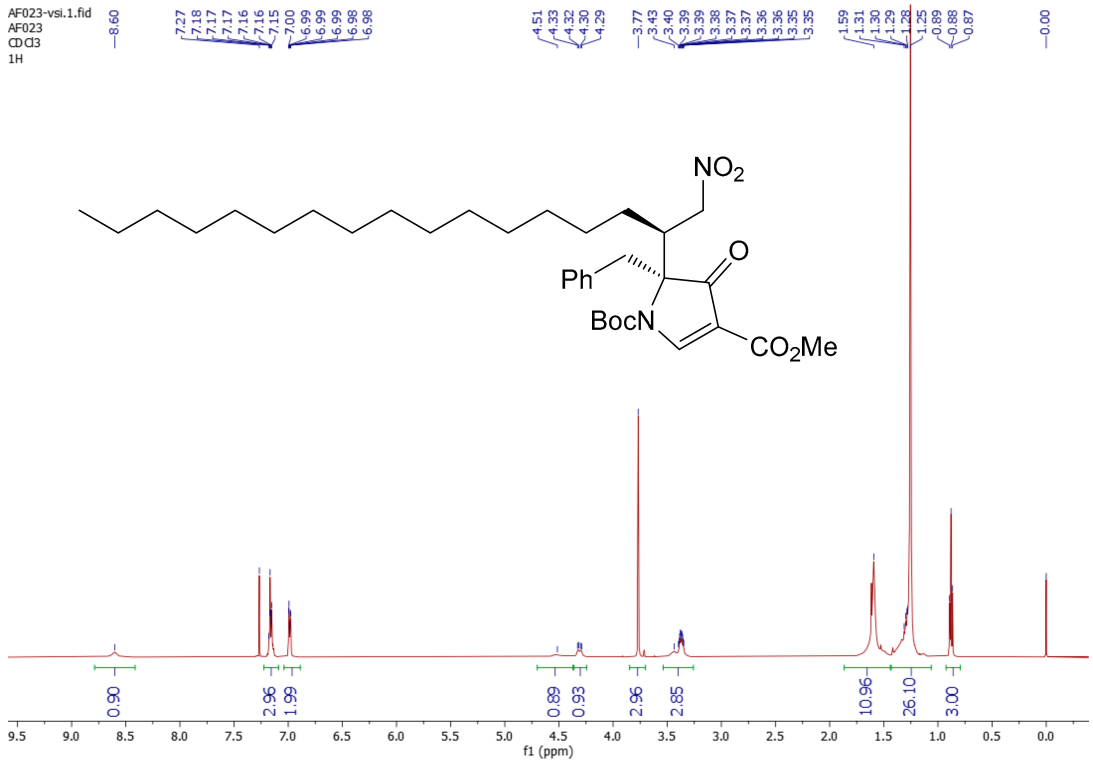

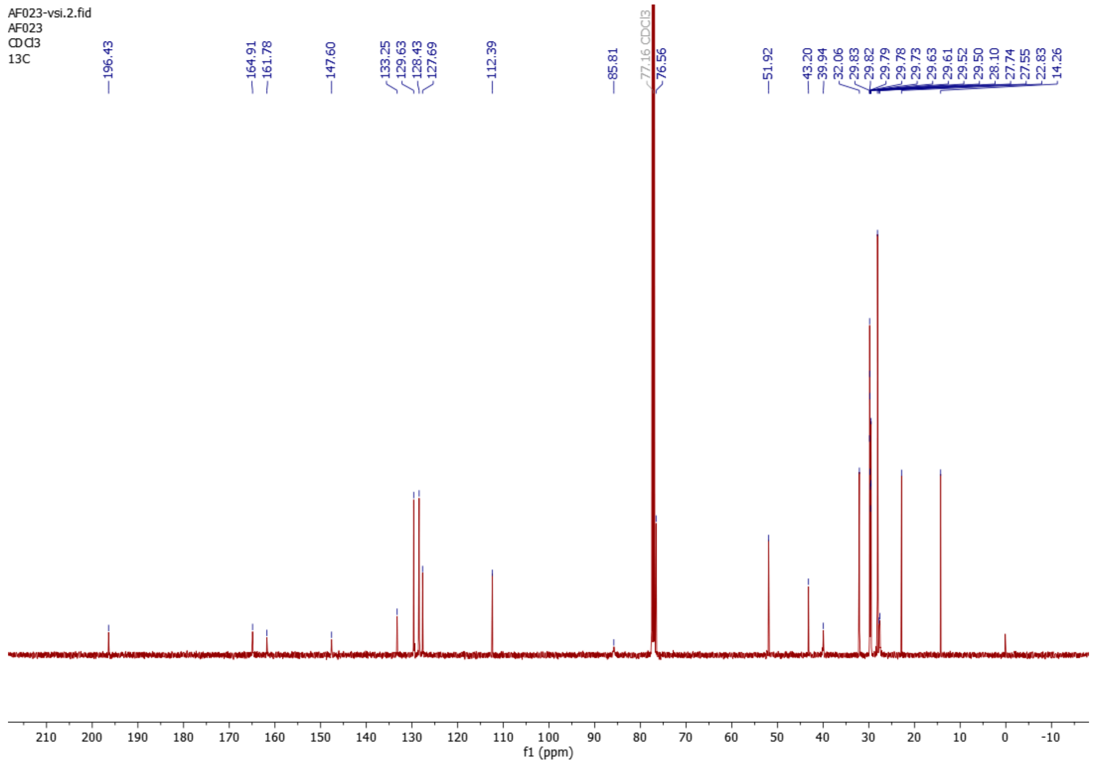

1-(*tert*-Butyl) 3-methyl 5-(4-(((benzyloxy)carbonyl)amino)butyl)-5-(1-nitroheptadecan-2-yl)-4-oxo-4,5-dihydro-1*H*-pyrrole-1,3-dicarboxylate (18b)

Racemic: diastereomer 1/diastereomer 2 = 90:10

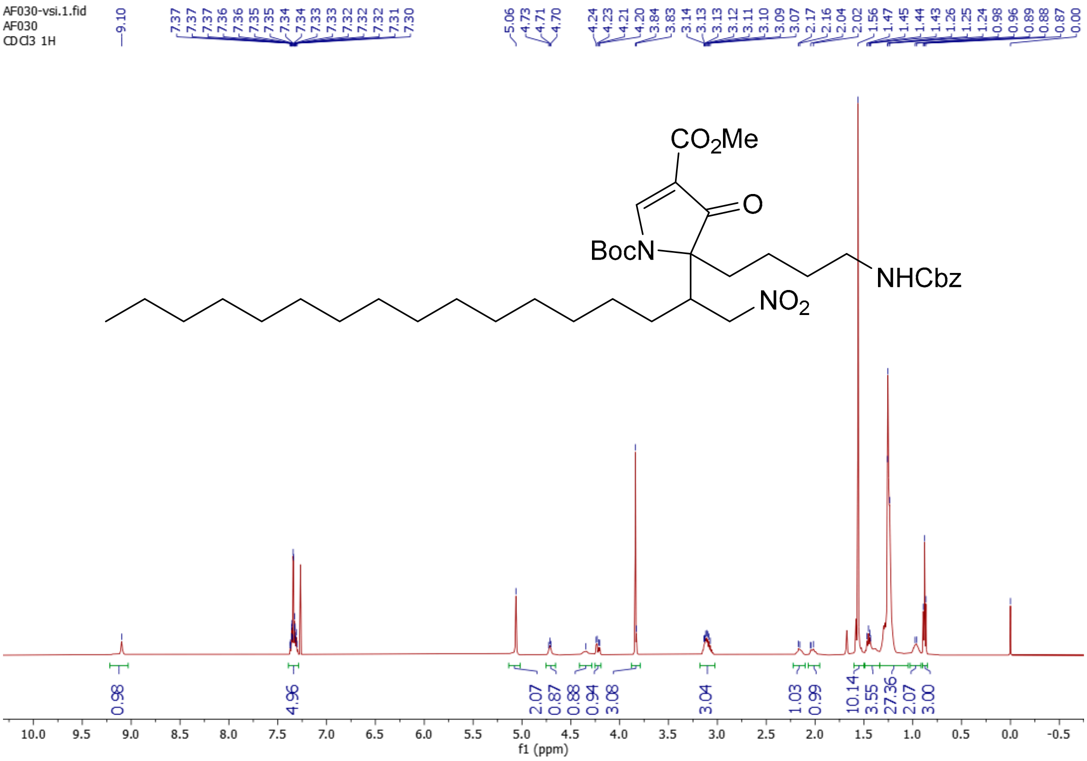

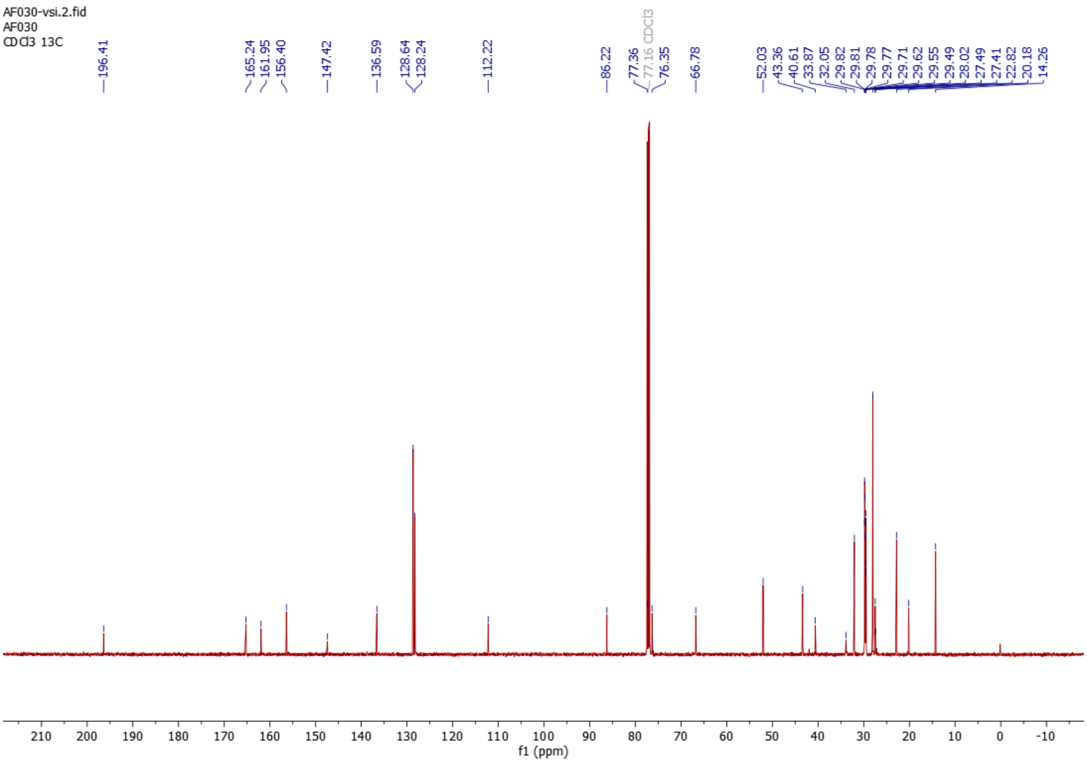

Nonracemic: diastereomer 1/diastereomer 2 = 26:74

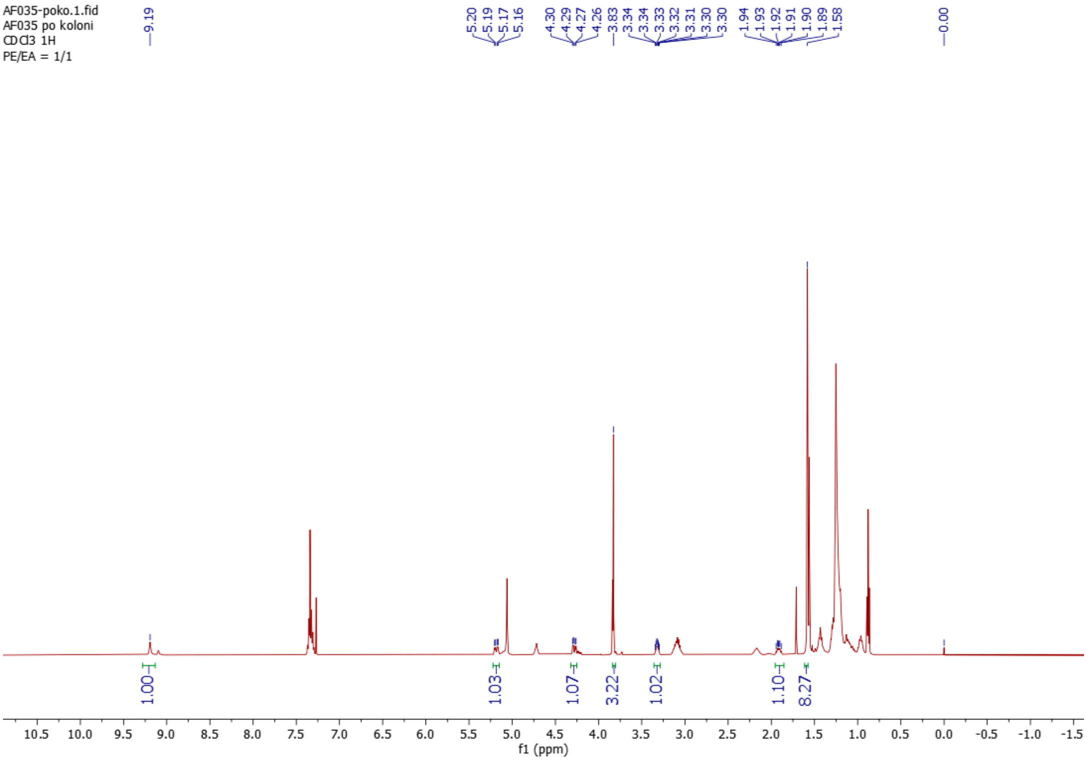

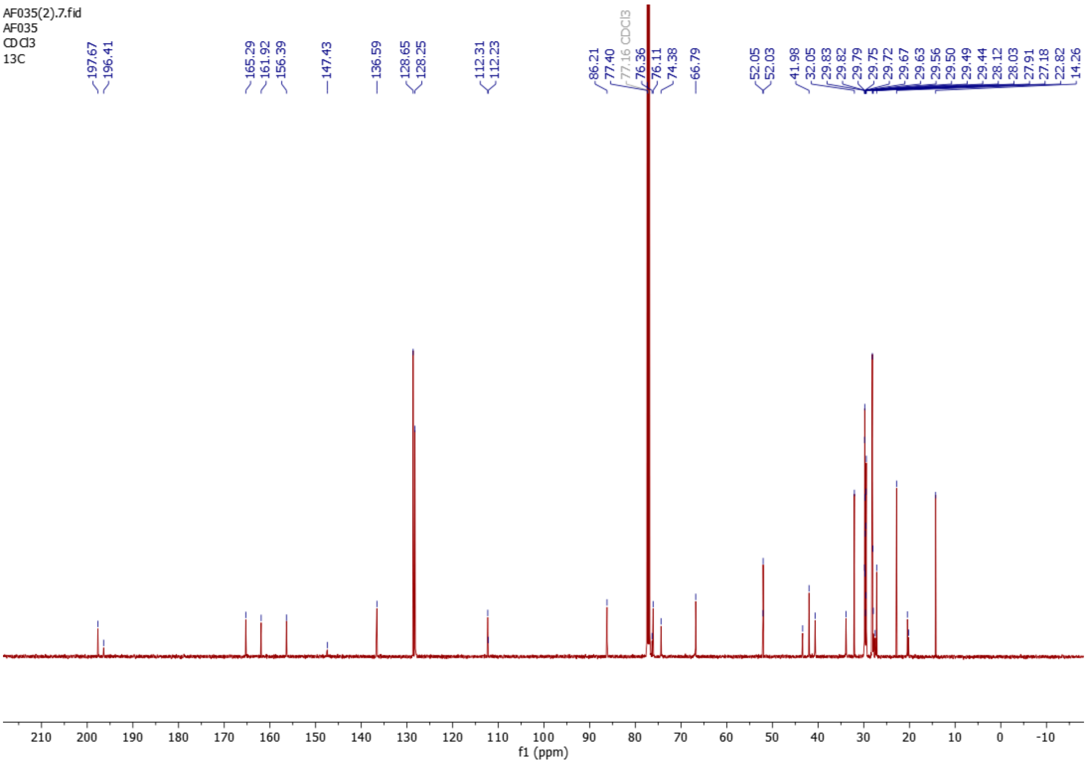

1-(*tert*-Butyl) 3-methyl 5-(3-(benzyloxy)-3-oxopropyl)-5-(1-nitroheptadecan-2-yl)-4-oxo-4,5-dihydro-1*H*-pyrrole-1,3-dicarboxylate (18c)

Racemic: diastereomer 1/diastereomer 2 = 93:7

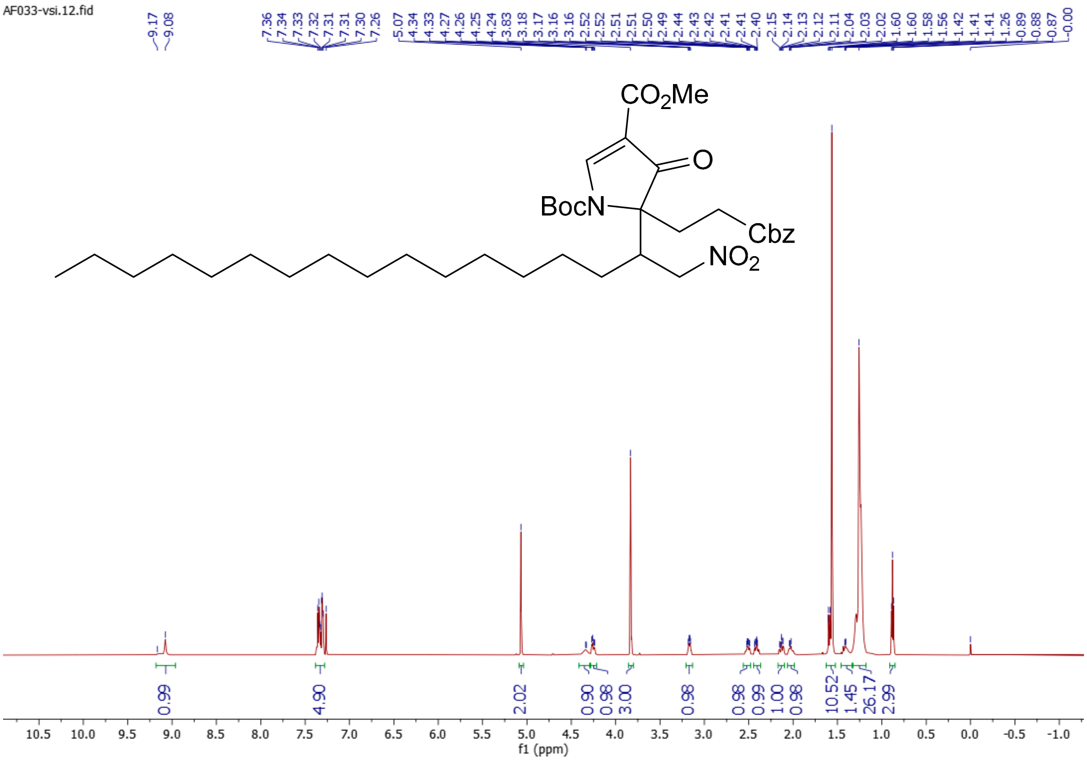

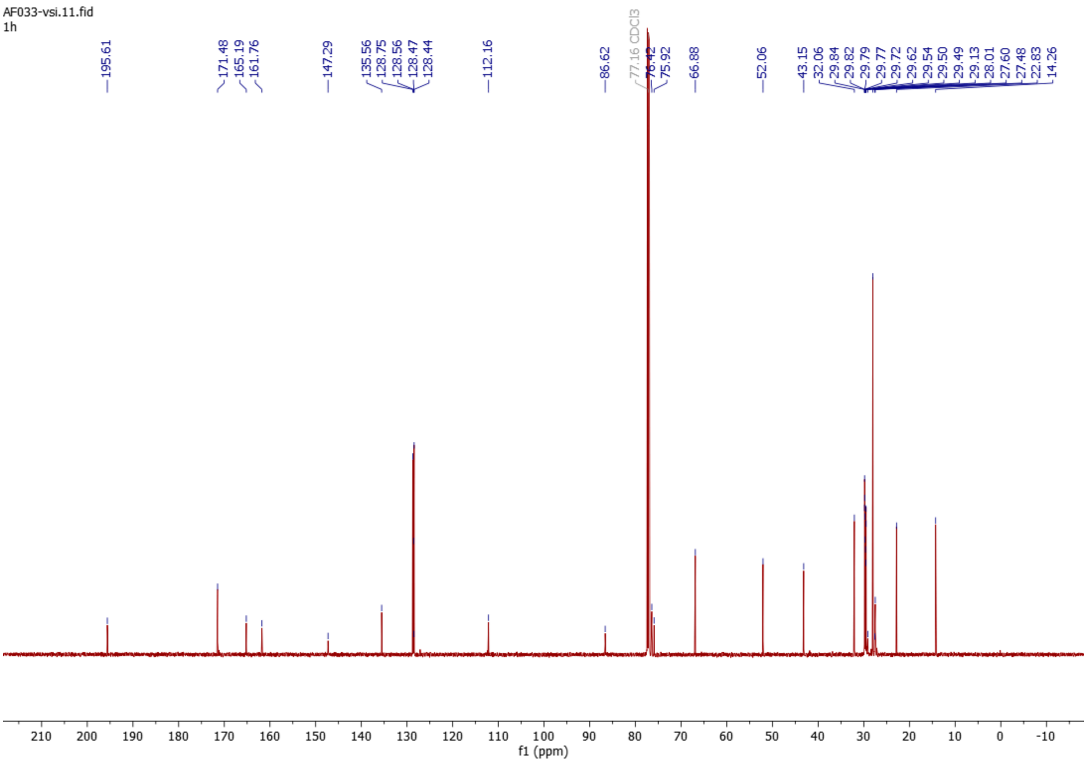

Nonracemic: diastereomer 1/diastereomer 2 = 31:69

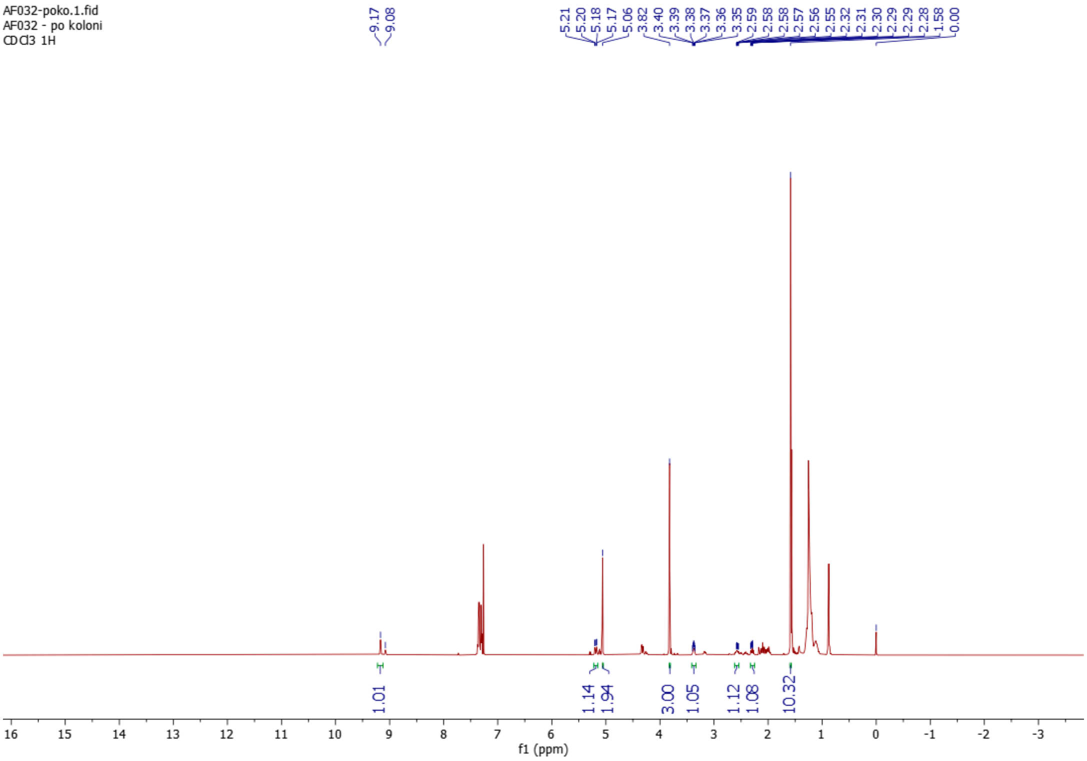

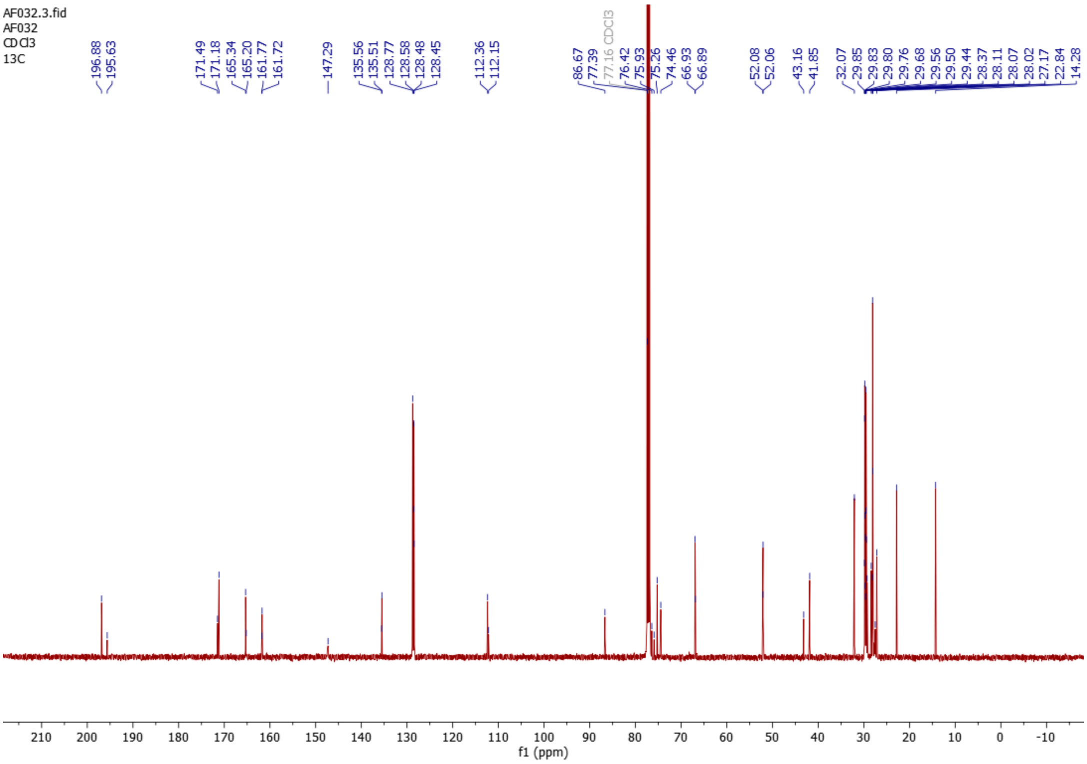

4-Hydroxy-3-(1-nitroheptadecan-2-yl)furan-2(5*H*)-one (20)

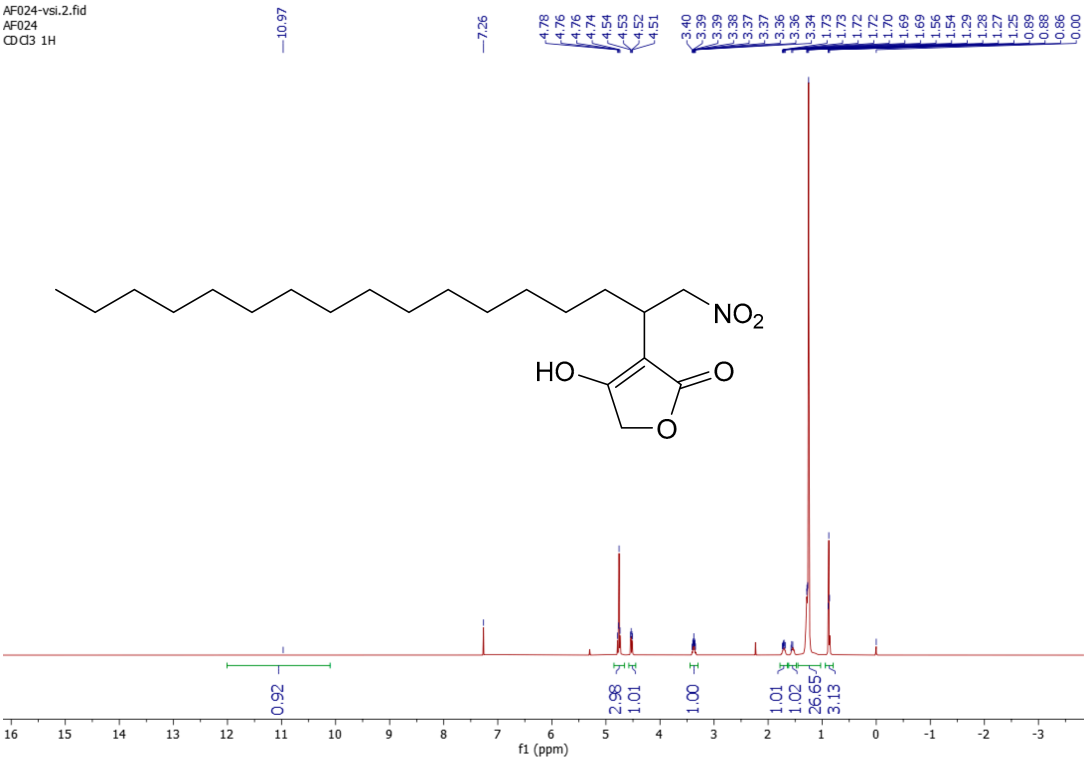

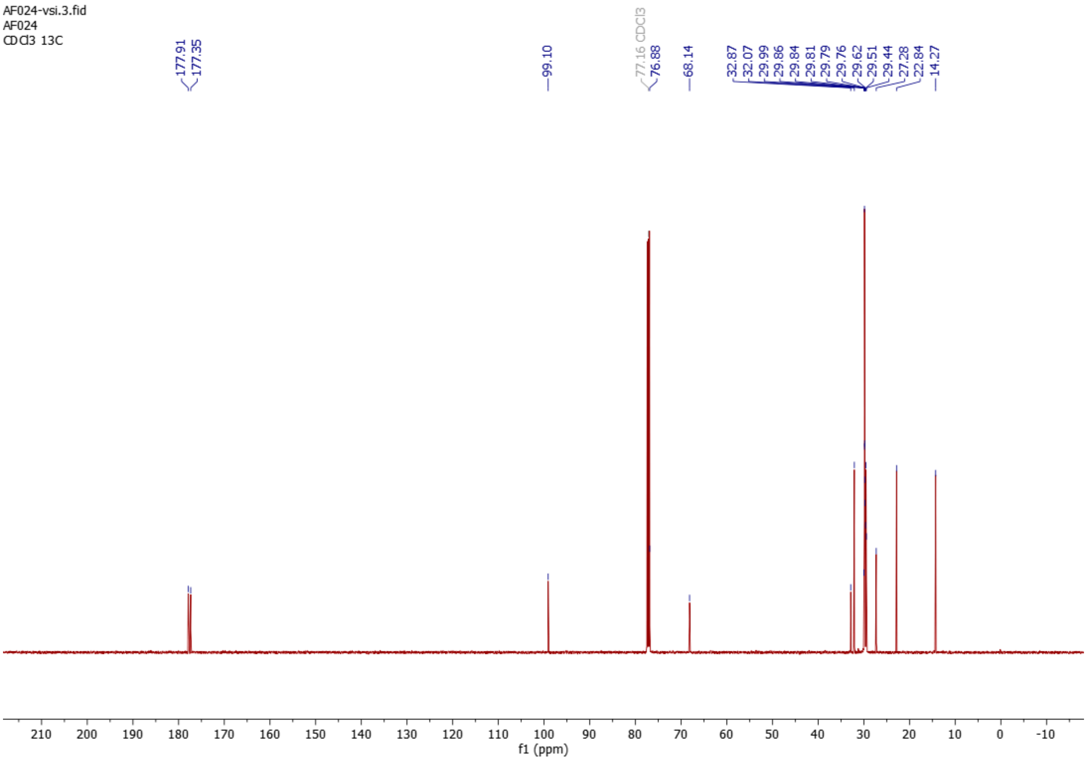

4. Structure determination by X-ray diffraction analysis

Table S1. Crystal data and structure refinement for compound **17a**.

Empirical formula	$C_{18}H_{24}N_2O_7$
Formula weight	380.39
Temperature/K	152(4)
Crystal system	Triclinic
Space group	P1
a [Å^3]	6.0296(3)
b [Å^3]	8.1461(4)
c [Å^3]	10.3061(5)
α [°]	103.129(4)
β [°]	95.329(4)
γ [°]	102.783(4)
V [Å^3]	475.27(4)
Z	1
ρ_{calc} [g/cm^3]	1.329
μ [mm^{-1}]	0.103
F(000)	202.0
Crystal size/mm^3	0.3 × 0.3 × 0.2
Radiation	MoKα (λ = 0.71073)
Reflections collected	10087
Independent reflections	4954
R_{int}	0.0307
Data/restraints/parameters	4954/3/248
GOF	1.039
R_1, wR_2 [I>=2σ (I)]	0.0449, 0.1050
R_1, wR_2 (all data)	0.0554, 0.1142
$(\Delta\rho)_{max}$ [e Å^{-3}]	0.25
$(\Delta\rho)_{min}$ [e Å^{-3}]	–0.23
Flack parameter	–0.3(5)

General Information (for SI)

Single-crystal X-ray diffraction data was collected on Agilent Technologies SuperNova Dual diffractometer with an Atlas detector using monochromated Mo-K_α radiation (λ = 0.71073 Å) at 150 K. The data was processed using CrysAlis PRO [17]. Using Olex2.1.2. [18], the structure was solved by direct methods implemented in SHELXS [19] or SHELXT [20] and refined by a full-matrix least-squares procedure based on F^2 with SHELXT-2014/7 [21]. All nonhydrogen atoms were refined anisotropicallly. Hydrogen atoms were placed in geometrically calculated positions and were refined using a riding model. The drawing and the analysis of bond lengths, angles and intermolecular interactions were carried out using Mercury [22] and Platon [23]. Structural and other crystallographic details on data collection and refinement for compound **17a** have been deposited with the Cambridge Crystallographic Data Centre as supplementary publication number CCDC Deposition Number **2513139**. These data can be obtained free of charge via www.ccdc.cam.ac.uk/conts/retrieving.html (or from the CCDC, 12 Union Road, Cambridge CB2 1EZ, UK; fax: +44 1223 336033; e-mail: deposit@ccdc.cam.ac.uk).

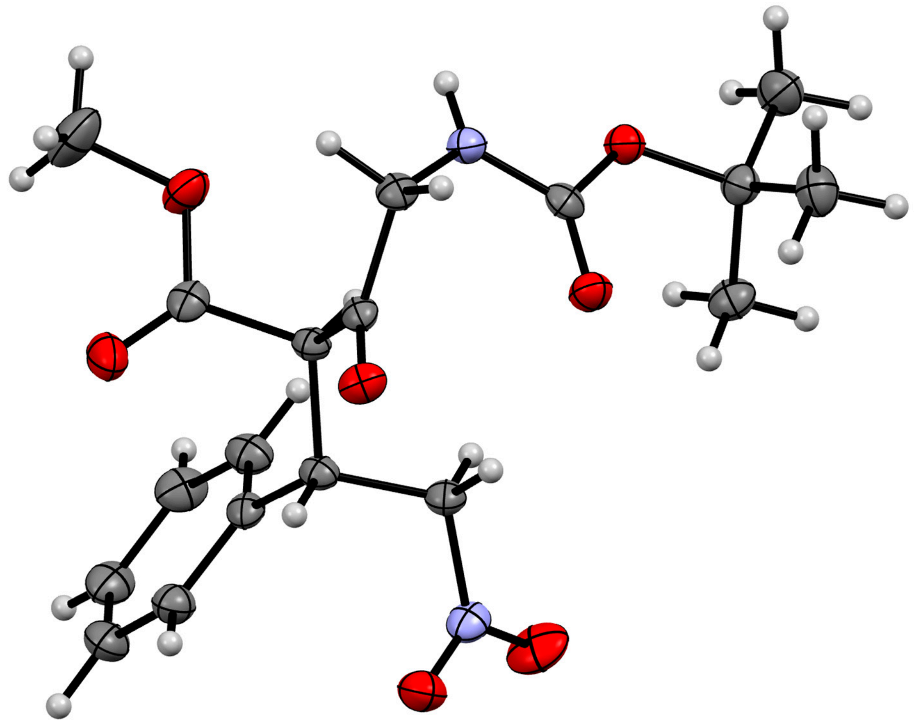

Figure S1. Molecular structure of product **17a**. Thermal ellipsoids are shown at 50% probability.

5. References

[1] Ričko, S.; Svete, J.; Štefane, B.; Perdih, A.; Golobič, A.; Meden, A.; Grošelj, U., 1,3-Diamine-Derived Bifunctional Organocatalyst Prepared from Camphor. *Adv. Synth. Catal.* **2016**, *358*, 3786–3796. https://doi.org/10.1002/adsc.201600498

[2] Mailhol, D.; Duque, M. d. M. S.; Raimondi, W.; Bonne, D.; Constantieux, T.; Coquerel, Y.; Rodriguez, J., Enantioselective Organocatalytic Michael Addition of Cyclobutanones to Nitroalkenes. *Adv. Synth. Catal.* **2012**, *354*, 3523–3532. https://doi.org/10.1002/adsc.201200658

[3] Konishi, H.; Lam, T. Y.; Malerich, J. P.; Rawal, V. H., Enantioselective α-Amination of 1,3-Dicarbonyl Compounds Using Squaramide Derivatives as Hydrogen Bonding Catalysts. *Org. Lett.* **2010**, *12*, 2028–2031. https://doi.org/10.1021/ol1005104

[4] Badiola, E.; Fiser, B.; Gómez-Bengoa, E.; Mielgo, A.; Olaizola, I.; Urruzuno, I.; García, J. M.; Odriozola, J. M.; Razkin, J.; Oiarbide, M.; Palomo, C., Enantioselective Construction of Tetrasubstituted Stereogenic Carbons through Brønsted Base Catalyzed Michael Reactions: α′-Hydroxy Enones as Key Enoate Equivalent. *J. Am. Chem. Soc.* **2014**, *136*, 17869–17881. https://doi.org/10.1021/ja510603w

[5] Vakulya, B.; Varga, S.; Csámpai, A.; Soós, T., Highly Enantioselective Conjugate Addition of Nitromethane to Chalcones Using Bifunctional Cinchona Organocatalysts. *Org. Lett.* **2005**, *7*, 1967–1969. https://doi.org/10.1021/ol050431s

[6] Malerich, J. P.; Hagihara, K.; Rawal, V. H., Chiral Squaramide Derivatives are Excellent Hydrogen Bond Donor Catalysts. *J. Am. Chem. Soc.* **2008**, *130*, 14416–14417. https://doi.org/10.1021/ja805693p

[7] Yang, W.; Du, D.-M., Highly Enantioselective Michael Addition of Nitroalkanes to Chalcones Using Chiral Squaramides as Hydrogen Bonding Organocatalysts. *Org. Lett.* **2010**, *12*, 5450–5453. https://doi.org/10.1021/ol102294g

[8] Grošelj, U.; Žorž, M.; Golobič, A.; Stanovnik, B.; Svete, J., α-Amino acid derived enaminones and their application in the synthesis of *N*-protected methyl 5-substituted-4-hydroxypyrrole-3-carboxylates and other heterocycles. *Tetrahedron* **2013**, *69*, 11092–11108. https://doi.org/10.1016/j.tet.2013.11.008

[9] Li, Y.-J.; Lee, P.-T.; Yang, C.-M.; Chang, Y.-K.; Weng, Y.-C.; Liu, Y.-H., [2,3]-Wittig rearrangement of methyl β-pyrrolidinyl-γ-allyloxy-(E)-2-butenoate. Expeditious synthesis of 5-alkenyl-4-pyrrolidin-1-yl-5H-furan-2-ones. *Tetrahedron Lett.* **2004**, *45*, 1865–1868. https://doi.org/10.1016/j.tetlet.2004.01.013

[10] Yatsuzuka, K.; Kawasaki, M.; Shirai, R., Enantioselective [2,3]-Wittig Rearrangement of Carboxylic Acid Derived Enolates by Tetradentate Chiral Lithium Amide. *Synlett* **2023**, *34*, 1727–1731. https://doi.org/10.1055/a-2039-6352

[11] Brinkerhoff, R. C.; Tarazona, H. F.; de Oliveira, P. M.; Flores, D. C.; Montes D'Oca, C. D. R.; Russowsky, D.; Montes D'Oca, M. G., Synthesis of β-ketoesters from renewable resources and Meldrum's acid. *RSC Advances* **2014**, *4*, 49556–49559. https://doi.org/10.1039/C4RA08986C

[12] Ričko, S.; Meden, A.; Ivančič, A.; Perdih, A.; Štefane, B.; Svete, J.; Grošelj, U., Organocatalyzed Deracemization of Δ^2-Pyrrolin-4-ones. *Adv. Synth. Catal.* **2017**, *359*, 2288–2296. https://doi.org/10.1002/adsc.201700539

[13] Franchini, L.; Panza, L.; Kongmanas, K.; Tanphaichitr, N.; Faull, K. F.; Ronchetti, F., An efficient and convenient synthesis of deuterium-labelled seminolipid isotopomers and their ESI-MS characterization. *Chem. Phys. Lipids* **2008**, *152*, 78–85. https://doi.org/10.1016/j.chemphyslip.2008.02.002

[14] Bevan, T. W.; Francis-Taylor, J.; Wong, H.; Northcote, P. T.; Harvey, J. E., A colourful azulene-based protecting group for carboxylic acids. *Tetrahedron* **2018**, *74*, 2942–2955. https://doi.org/10.1016/j.tet.2018.04.066

[15] Jirošová, A.; Majer, P.; Jančařík, A.; Dolejšová, K.; Tykva, R.; Šobotník, J.; Jiroš, P.; Hanus, R., Sphinganine-Like Biogenesis of (*E*)-1-Nitropentadec-1-ene in Termite Soldiers of the Genus Prorhinotermes. *ChemBioChem* **2014**, *15*, 533–536. https://doi.org/10.1002/cbic.201300665

[16] Kuldová, J.; Hrdý, I.; Svatos, A., Defense Secretion of Prorhinotermes simplex: Toxicity to Insecticide Susceptible and Resistant House Fly. *J. Chem. Ecol.* **1999**, *25*, 657–662. https://doi.org/10.1023/A:1020918323678

[17] CrysAlis PRO; Agilent Technologies UK Ltd.: Yarnton, UK, **2011**.

[18] Dolomanov, O.V.; Bourhis, L.J.; Gildea, R.J.; Howard, J.A.K.; Puschmann, H. OLEX2: A complete structure solution, refinement and analysis program. *J. Appl. Cristallogr.* **2009**, *42*, 339–341. https://doi.org/10.1107/S0021889808042726

[19] Sheldrick, G.M. A short history of SHELX. *Acta Crystallogr. A* **2008**, *64*, 112–122. https://doi.org/10.1107/S0108767307043930

[20] Sheldrick, G.M. SHELXT-Integrated space-group and crystal-structure determination. *Acta Crystallogr. Sect. Found. Adv.* **2015**, *71*, 3–8. https://doi: 10.1107/S2053273314026370

[21] Sheldrick, G.M. Crystal structure refinement with SHELXL. *Acta Crystallogr. Sect. C Struct. Chem.* **2015**, *71*, 3–8. https://doi.org/10.1107/S2053229614024218

[22] Macrae, C.F.; Edgington, P.R.; McCabe, P.; Pidcock, E.; Shields, G.P.; Taylor, R.; Towler, M.; van de Streek, J. *Mercury*: visualization and analysis of crystal structures. *J. Appl. Crystallogr.* **2006**, *39*, 453–457. https://doi.org/10.1107/S002188980600731X

[23] Spek, A.L. Single-crystal structure validation with the program PLATON. *J. Appl. Crystallogr.* **2003**, *3*6, 7–13. https://doi.org/10.1107/S0021889802022112
